# Tirzepatide on ingestive behavior in adults with overweight or obesity: a randomized 6-week phase 1 trial

**DOI:** 10.1038/s41591-025-03774-9

**Published:** 2025-06-24

**Authors:** Corby K. Martin, Owen T. Carmichael, Susan Carnell, Robert V. Considine, David A. Kareken, Ulrike Dydak, Richard D. Mattes, David Scott, Sergey Shcherbinin, Hiroshi Nishiyama, Alastair Knights, Shweta Urva, Lukasz Biernat, Edward Pratt, Axel Haupt, Mark Mintun, Diana Otero Svaldi, Zvonko Milicevic, Tamer Coskun

**Affiliations:** 1 Pennington Biomedical Research Center, Louisiana State University System, Baton Rouge, LA USA; 2 Department of Psychiatry and Behavioral Sciences, Johns Hopkins University School of Medicine, Baltimore, MD USA; 3 Department of Medicine, Indiana University School of Medicine, Indianapolis, IN USA; 4 School of Health Sciences, Purdue University, West Lafayette, IN USA; 5 Department of Nutrition Science, Purdue University, West Lafayette, IN USA; 6 Clario, Inc., San Mateo, CA USA; 7 Eli Lilly and Company, Indianapolis, IN USA

**Keywords:** Obesity, Obesity

## Abstract

Tirzepatide induces weight reduction but the underlying mechanisms are unknown. This 6-week phase 1 study investigated early effects of tirzepatide on energy intake. Male and female adults without diabetes (*n* = 114) and a body mass index from 27 to 50 kg per m^2^ were randomized 1:1:1 to blinded once-weekly tirzepatide or placebo, or open-label once-daily liraglutide. The primary outcome was change from baseline to week 3 in energy intake during an ad libitum lunch with tirzepatide versus placebo. Secondary outcomes assessed self-reported ingestive behavior and blood-oxygenation-level-dependent functional magnetic resonance imaging with food photos. Tirzepatide reduced energy intake versus placebo at week 3 (estimated treatment difference −524.6 kcal (95% confidence interval −648.1 to −401.0), *P* < 0.0001). With regard to secondary outcomes versus placebo, tirzepatide decreased overall appetite, food cravings, tendency to overeat, perceived hunger and reactivity to foods in the environment but did not impact volitional restriction of dietary intake. At week 3 versus placebo, tirzepatide did not statistically significantly impact blood-oxygenation-level-dependent activation to highly palatable food photos (aggregated category of high-fat, high-sugar foods and high-fat, high-carbohydrate foods) but decreased activation to high-fat, high-sugar food photos in the medial frontal and cingulate gyri, orbitofrontal cortex and hippocampus. Our results suggest tirzepatide reduces food intake, potentially by impacting ingestive behavior. ClinicalTrials.gov registration: NCT04311411.

## Abstract (web summary)

In a 6-week randomized phase 1 trial, tirzepatide led to reduced caloric intake during a meal test at week 3 compared with placebo and liraglutide in people with overweight or obesity.

## Main

Obesity is a chronic progressive disease characterized by excessive body fat^1–3^. New incretin-based pharmacotherapies produce weight reduction^4^. However, the behavioral and neurobiological mechanisms underlying their effects on body weight in humans are not well understood^5^.

Previous studies of selective glucagon-like peptide-1 (GLP-1) receptor agonists (RAs) using self-report inventories suggest these therapies affect ingestive behaviors^6,7^, while functional magnetic resonance imaging (fMRI) studies using food cues show involvement of central nervous system (CNS) mechanisms^8–13^. These findings suggest that GLP-1 RAs may have early effects on energy intake by modifying appetite and brain activity in regions implicated in appetite and food reward.

Tirzepatide is a glucose-dependent insulinotropic polypeptide (GIP) and GLP-1 RA^14^. A 72-week phase 3 study in adults with obesity demonstrated that once-weekly tirzepatide produced weight reduction of up to 20.9%, compared to 3.1% with placebo^15^. A phase 1 mechanistic study in individuals with obesity undergoing energy restriction or weight reduction found that tirzepatide reduced energy intake and food cravings^16^, with no effect on metabolic adaptation^17^, indicating that tirzepatide’s effect on body weight may primarily be related to its effect on energy intake and appetite. Tirzepatide has similarly been shown to reduce appetite and energy intake in individuals with diabetes^18^. However, these studies used limited measures of ingestive behavior and did not collect brain functioning measurements that could inform CNS mechanisms.

Early adaptive changes in ingestive behavior and involvement of CNS mechanisms producing energy intake reductions with tirzepatide are yet to be fully elucidated in humans. We conducted a 6-week phase 1 clinical trial in adults with obesity or overweight to better understand the effect of short-term tirzepatide administration on appetite, ingestive behaviors and blood-oxygen-level-dependent (BOLD) fMRI activation in response to food cues relative to placebo and liraglutide. We hypothesized that tirzepatide would decrease energy intake and appetite, and modulate activation of brain regions associated with appetite and food reward.

## Results

**Fig. 1:**
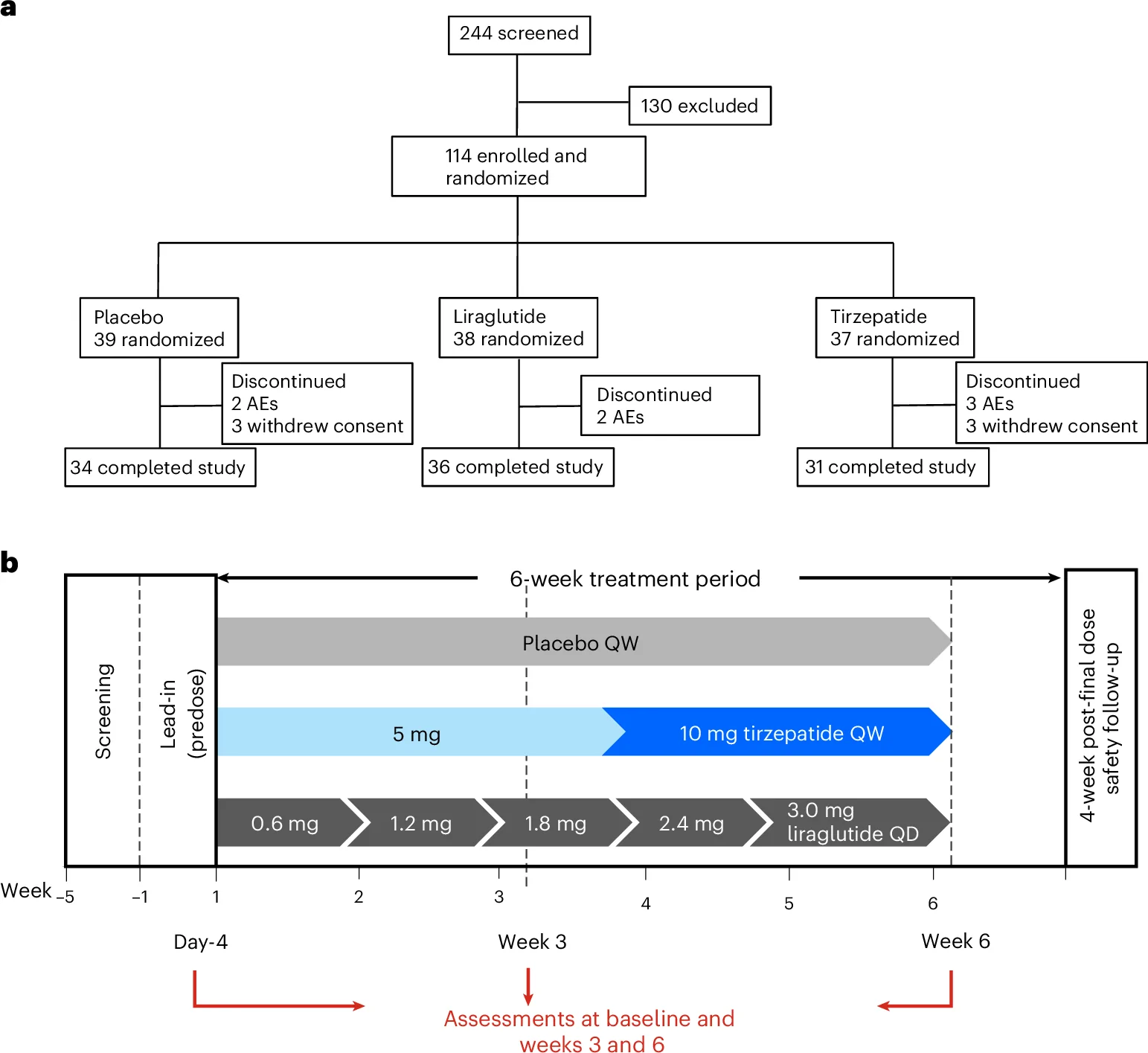
Trial profile and study design. **a**, Trial profile of the number of participants who underwent screening, enrollment and randomization and the number of participants who completed the study. **b**, Study design. The assessments carried out included an ad libitum food intake test (primary objective at week 3); questionnaires assessing appetite (VAS), food cravings (FCI and FCQ-S), disinhibition, hunger and dietary restraint (Eating Inventory), susceptibility to the food environment (PFS) and impulsiveness (BIS); and BOLD fMRI (fasted). QD, once-daily; QW, once-weekly.

A total of 244 participants were screened and 114 were randomly assigned to placebo (*n* = 39), tirzepatide (*n* = 37) or liraglutide (*n* = 38). The first participant was enrolled on 9 November 2020 and the last participant was enrolled on 14 October 2022. All received at least one dose of study drug and 101 participants (89%) completed the study (Fig. 1a). Of 13 participants who did not complete the study, 7 discontinued due to adverse events (AEs) and 6 due to withdrawal of consent. Baseline demographics were well balanced across treatment groups except for sex, with fewer female participants in the liraglutide group (25 (66%)) than in the placebo (36 (92%)) and tirzepatide (36 (97%)) groups (Table 1).

**Table 1:** Baseline characteristics

	Placebo (*n* = 39)	Liraglutide (*n* = 38)	Tirzepatide (*n* = 37)	Overall (*N* = 114)
**Age (yr)**	46.2 (9.5)	43.7 (11.9)	44.8 (10.2)	44.9 (10.5)
**Sex**				
Female	36 (92%)	25 (66%)	36 (97%)	97 (85%)
Male	3 (8%)	13 (34%)	1 (3%)	17 (15%)
**Race**				
White	28 (72%)	28 (74%)	27 (73%)	83 (73%)
Black or African American	11 (28%)	10 (26%)	9 (24%)	30 (26%)
**Ethnicity**				
Hispanic or Latino	2 (5%)	3 (8%)	0	5 (4%)
Not Hispanic or Latino	37 (95%)	35 (92%)	36 (97%)	108 (95%)
**Weight (kg)**	98.2 (20.8)	101.1 (19.5)	97.6 (16.0)	99.0 (18.8)
**BMI (kg per** **m**^**2**^**)**	36.2 (5.9)	36.2 (5.4)	36.1 (5.7)	36.2 (5.6)
27 to <30 kg per m^2^	6 (15%)	4 (11%)	5 (14%)	15 (13%)
30 to <35 kg per m^2^	12 (31%)	13 (34%)	12 (32%)	37 (33%)
35 to 50 kg per m^2^	21 (54%)	21 (55%)	20 (54%)	62 (54%)
**Waist circumference (cm)**	110.3 (14.3)	110.7 (14.5)	109.8 (12.2)	110.3 (13.6)

Data are mean (standard deviation) or *n* (%). Sex was self-reported by participants. *n*, number of randomized participants in each treatment group; *N*, total number of randomized participants.

### Primary outcome

**Fig. 2:**
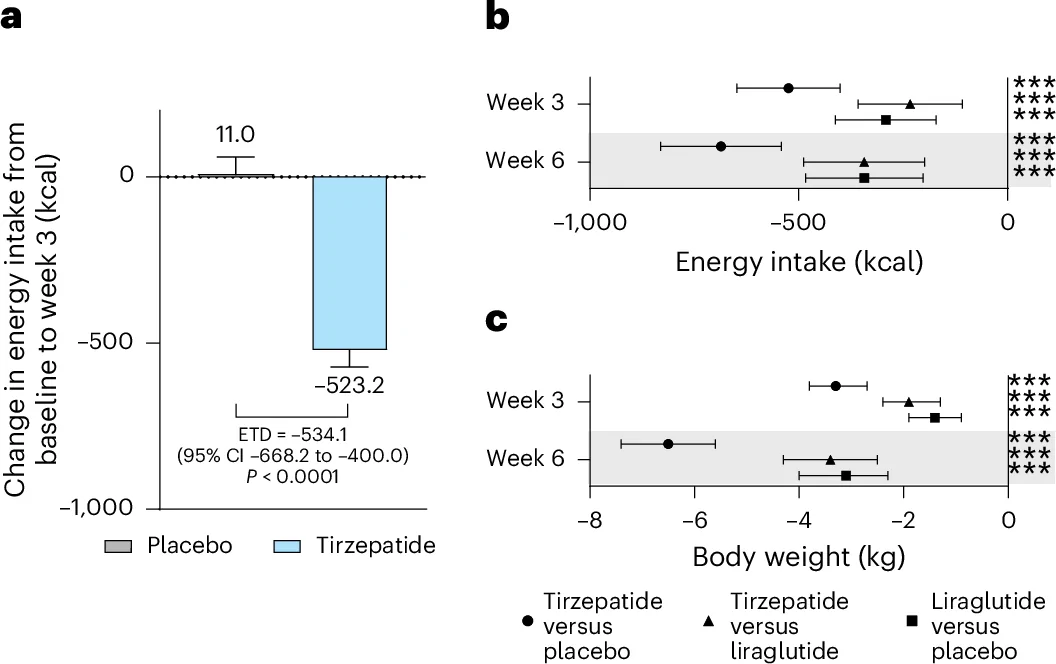
Differences between treatment groups in changes in energy intake and body weight. **a**, Least squares mean change from baseline (standard error), mean ETD and associated 95% CI from analysis of covariance for change in energy intake at week 3 in all randomized participants (placebo, *n* = 39; tirzepatide, *n* = 37). Statistical tests were two-sided at a significance level of 0.05 with adjustment for multiplicity. **b**, Mean ETD (center) and associated 95% CIs (whiskers) for change in energy intake at weeks 3 and 6. **c**, Mean ETD (center) and associated 95% CIs (whiskers) for change in body weight at weeks 3 and 6. For **b** and **c**, ETD was estimated using a MMRMs in all randomized participants (placebo, *n* = 39; tirzepatide, *n* = 37; liraglutide, *n* = 38). Statistical tests were two-sided at a significance level of 0.05 and adjustment was not made for multiplicity. ***P < 0.001 for comparisons between treatment groups. Statistical comparisons including exact *P* values are provided in Table 2 and Supplementary Table 1. ETD, estimated treatment difference. Source data

**Extended Data Fig. 1:**
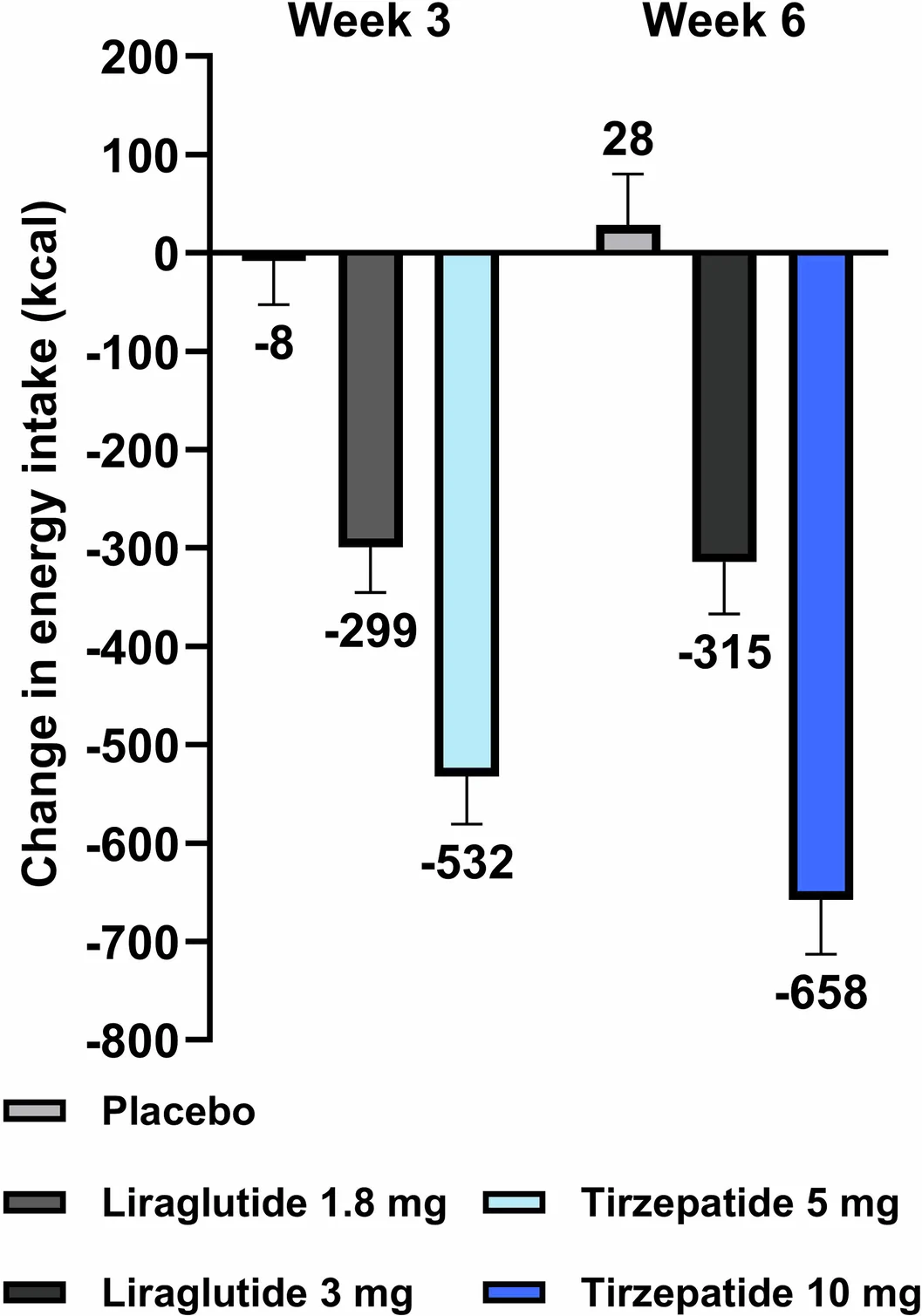
Changes in energy intake. Data are least squares mean (standard error) from mixed-model repeated measures for change in energy intake at week 3 and 6 in all randomized participants (placebo, n = 39; tirzepatide, n = 37; liraglutide, n = 38). Source data

The prespecified primary analysis using an analysis of covariance found a statistically significantly greater mean change from baseline to week 3 in energy intake during an ad libitum lunch meal test with tirzepatide (−523.2 kcal) versus placebo (11.0 kcal); treatment difference −534.1 kcal (95% confidence interval (CI) −668.2 to −400.0, *P* < 0.0001) (Fig. 2a). Results were similar when analyzed using a mixed model for repeated measures (MMRMs) (Fig. 2b and Extended Data Fig. 1).

### Secondary outcomes

**Fig. 3:**
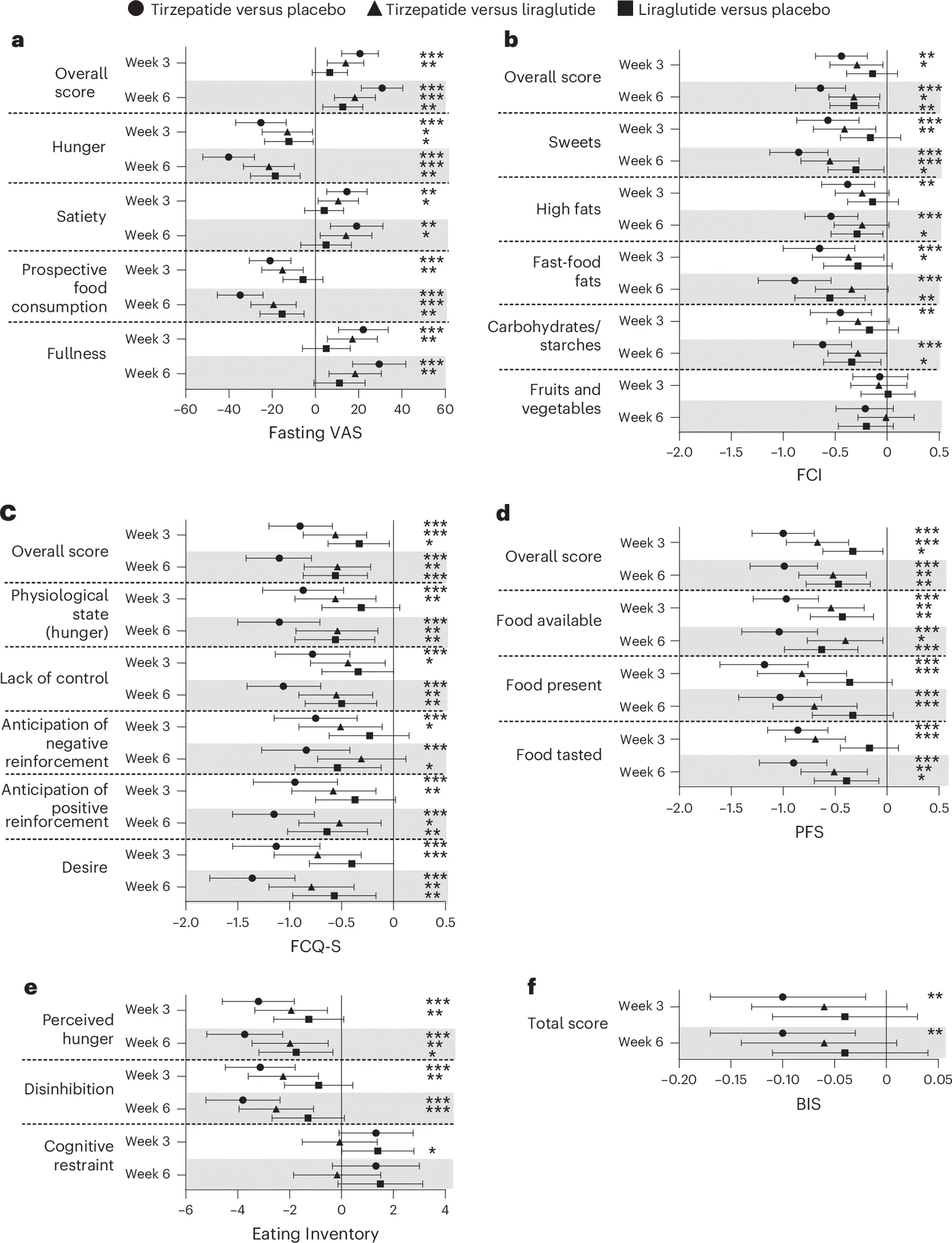
Differences between treatment groups in changes in ingestive behavior and impulsiveness. **a**–**f**, Data are presented as mean ETD (center) and associated 95% CIs (whiskers) for change in fasting VAS (**a**), FCI (**b**), FCQ-S (**c**), PFS (**d**), Eating Inventory (**e**) and BIS (**f**). ETD was estimated using a MMRMs in all randomized participants (placebo, *n* = 39; tirzepatide, *n* = 37; liraglutide, *n* = 38). Statistical tests were two-sided at a significance level of 0.05 and no adjustments were made for multiplicity. ^*^P < 0.05, ^**^P < 0.01, ^***^P < 0.001 for comparisons between treatment groups. Statistical comparisons including exact *P* values are provided in Table 2 and Supplementary Tables 4–7. Source data

**Extended Data Fig. 2:**
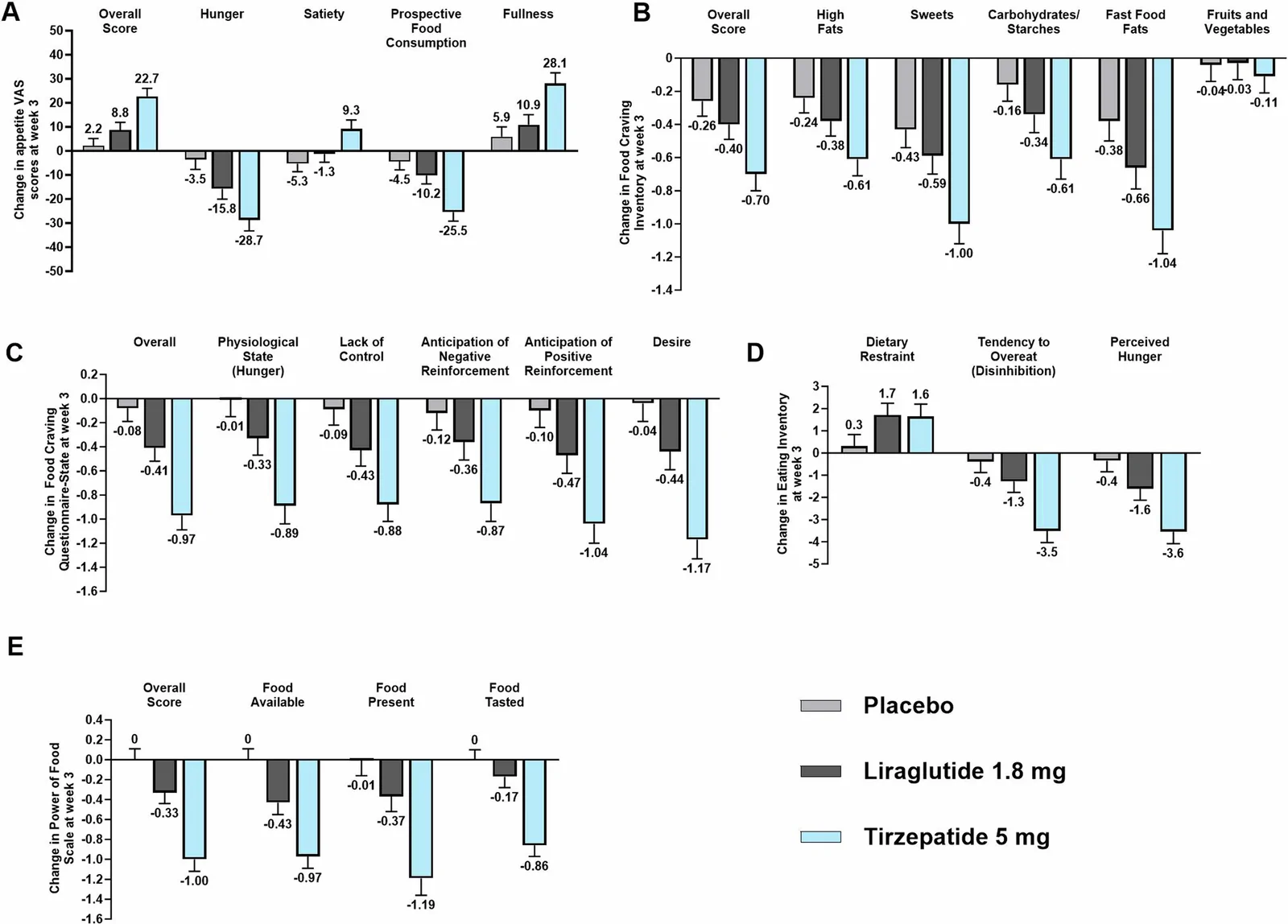
Changes in ingestive behaviour at week 3. Data are least squares mean (standard error) from mixed-model repeated measures for change in **A**) pre-lunch appetite VAS scores, **B**) Food Craving Inventory, **C**) Food Craving Questionnaire-State, **D**) Eating Inventory, and **E**) Power of Food Scale, at week 3 in all randomized participants (placebo, n = 39; tirzepatide, n = 37; liraglutide, n = 38). VAS=visual analogue scale. Source data

Tirzepatide decreased fasting overall appetite as assessed by visual analog scale (VAS) at week 3 versus placebo (Table 2, Fig. 3a and Extended Data Fig. 2). Tirzepatide decreased fasting hunger and prospective food consumption, and increased fasting satiety and fullness versus placebo at week 3 (Supplementary Table 1). Tirzepatide decreased fasting desire to eat sweet, salty and fatty foods as assessed by VAS versus placebo at week 3. Tirzepatide and placebo did not differ in any postprandial appetite changes at week 3 (Supplementary Table 2).

**Table 2:** Energy intake, body weight and eating behavior findings

	Placebo (*n* = 39)	Liraglutide (*n* = 38)	Tirzepatide (*n* = 37)
**Energy intake at lunch (kcal)**			
Baseline	893.1 (384.2)	1,044.8 (562.3)	926.4 (371.2)
Change at week 3 (primary analysis of covariance)	11.0 (49.1)	–	−523.2 (52.8)
Versus placebo	–	–	−534.11 (−668.20 to −400.02), *P* < 0.0001
Change at week 3	−7.9 (44.7) (1.9%)	−299.3 (46.2) (−31.5%)	−532.4 (48.3) (−59.1%)
Versus placebo	–	−291.4 (−412.1 to −170.8), *P* < 0.0001	−524.6 (−648.1 to −401.0), *P* < 0.0001
Versus liraglutide	–	–	−233.1 (−358.0 to −108.3), *P* = 0.0004
Change at week 6	28.3 (52.0) (−0.6%)	−314.5 (52.7) (−28.8%)	−657.8 (55.4) (−72.4%)
Versus placebo	–	−342.8 (−483.6 to −202.0), *P* < 0.0001	−686.0 (−830.7 to −541.4), *P* < 0.0001
Versus liraglutide	–	–	−343.3 (−488.2 to −198.3), *P* < 0.0001
Change from week 3 to week 6 within treatment group	36.1 (−26.1 to 98.4), *P* = 0.2522 (2.7%)	−15.2 (−75.7 to 45.3), *P* = 0.6188 (2.7%)	−125.3 (−188.8 to −61.9), *P* = 0.0002 (−35.5%)
**Body weight (kg)**			
Baseline	98.7 (20.5)	101.8 (19.3)	98.2 (16.2)
Change at week 3	−0.5 (0.2)	−1.9 (0.2)	−3.7 (0.2)
Versus placebo	–	−1.4 (−1.9 to −0.9), *P* < 0.0001	−3.3 (−3.8 to −2.7), *P* < 0.0001
Versus liraglutide	–	–	−1.9 (−2.4 to −1.3), *P* < 0.0001
Change at week 6	−0.6 (0.3)	−3.7 (0.3)	−7.0 (0.4)
Versus placebo	–	−3.1 (−4.0 to −2.3), *P* < 0.0001	−6.5 (−7.4 to −5.6), *P* < 0.0001
Versus liraglutide	–	–	−3.4 (−4.3 to −2.5), *P* < 0.0001
Change from week 3 to week 6 within treatment group	−0.1 (−0.5 to 0.3), *P* = 0.6720	−1.8 (−2.3 to −1.4) *P* < 0.0001	−3.3 (−3.8 to −2.9) *P* < 0.0001
**Fasting overall appetite VAS score**^**a**^			
Baseline	26.3 (11.3)	25.6 (18.7)	25.3 (14.0)
Change at week 3	2.2 (3.0)	8.8 (3.1)	22.7 (3.3)
Versus placebo	–	6.6 (−1.5 to 14.8), *P* = 0.1097	20.6 (12.1 to 29.0), *P* < 0.0001
Versus liraglutide	–	–	13.9 (5.5 to 22.4), *P* = 0.0015
Change at week 6	−2.4 (3.5)	10.2 (3.4)	28.4 (3.6)
Versus placebo	–	12.6 (3.4 to 21.8), *P* = 0.0080	30.8 (21.3 to 40.4), *P* < 0.0001
Versus liraglutide	–	–	18.2 (8.8 to 27.7), *P* = 0.0002
Change from week 3 to week 6 within treatment group	−4.6 (−11.2 to 2.0), *P* = 0.1708	1.37 (−5.1 to 7.8), *P* = 0.6717	5.7 (−1.2 to 12.5), *P* = 0.1024
**Eating Inventory cognitive restraint**			
Baseline	9.6 (4.0)	9.4 (3.9)	9.3 (4.6)
Change at week 3	0.3 (0.5)	1.7 (0.5)	1.6 (0.6)
Versus placebo	–	1.4 (0 to 2.8), *P* = 0.0487	1.3 (−0.1 to 2.8), *P* = 0.0682
Versus liraglutide	–	–	−0.1 (−1.5 to 1.4), *P* = 0.9273
Change at week 6	1.1 (0.6)	2.6 (0.6)	2.4 (0.6)
Versus placebo	–	1.5 (−0.1 to 3.1), *P* = 0.0723	1.3 (−0.4 to 3.0), *P* = 0.1192
Versus liraglutide	–	–	−0.2 (−1.9 to 1.5), *P* = 0.8428
Change from week 3 to week 6 within treatment group	0.8 (−0.2 to 1.8), *P* = 0.1135	0.9 (−0.1 to 1.9), *P* = 0.0736	0.8 (−0.2 to 1.8), *P* = 0.1297
**Eating Inventory disinhibition (tendency to overeat)**			
Baseline	10.2 (3.5)	9.2 (3.5)	9.5 (3.5)
Change at week 3	−0.4 (0.5)	−1.3 (0.5)	−3.5 (0.5)
Versus placebo	–	−0.9 (−2.2 to 0.4), *P* = 0.1850	−3.1 (−4.5 to −1.8), *P* < 0.0001
Versus liraglutide	–	–	−2.3 (−3.6 to −0.9), *P* = 0.0014
Change at week 6	−0.6 (0.52)	−1.9 (0.5)	−4.4 (0.6)
Versus placebo	–	−1.3 (−2.7 to 0.1), *P* = 0.0711	−3.8 (−5.2 to −2.4), *P* < 0.0001
Versus liraglutide	–	–	−2.5 (−4.0 to −1.1), *P* = 0.0007
Change from week 3 to week 6 within treatment group	−0.2 (−0.9 to 0.5), *P* = 0.5687	−0.6 (−1.3 to 0.1), *P* = 0.0862	−0.9 (−1.6 to −0.2), *P* = 0.0182
**Eating Inventory perceived hunger**			
Baseline	6.8 (3.4)	6.5 (3.3)	7.1 (4.3)
Change at week 3	−0.4 (0.5)	−1.6 (0.5)	−3.6 (0.5)
Versus placebo	–	−1.3 (−2.6 to 0.1), *P* = 0.0664	−3.2 (−4.6 to −1.8), *P* < 0.0001
Versus liraglutide	–	–	−1.9 (−3.3 to −0.5), *P* = 0.0071
Change at week 6	−0.5 (0.5)	−2.2 (0.5)	−4.2 (0.6)
Versus placebo	–	−1.8 (−3.2 to −0.3), *P* = 0.0163	−3.7 (−5.2 to −2.3), *P* < 0.0001
Versus liraglutide	–	–	−2.0 (−3.5 to −0.5), *P* = 0.0090
Change from week 3 to week 6 within treatment group	−0.1 (−0.8 to 0.6), *P* = 0.7842	−0.6 (−1.3 to 0.1), *P* = 0.0916	−0.6 (−1.3 to 0.1), *P* = 0.0872
**FCI overall score**			
Baseline	2.5 (0.4)	2.7 (0.5)	2.4 (0.6)
Change at week 3	−0.3 (0.1)	−0.4 (0.1)	−0.7 (0.1)
Versus placebo	–	−0.1 (−0.4 to 0.1), *P* = 0.2376	−0.4 (−0.7 to −0.2), *P* = 0.0008
Versus liraglutide	–	–	−0.3 (−0.6 to 0), *P* = 0.0249
Change at week 6	−0.3 (0.1)	−0.6 (0.1)	−0.9 (0.1)
Versus placebo	–	−0.3 (−0.6 to −0.1), *P* = 0.0082	−0.6 (−0.9 to −0.4), *P* < 0.0001
Versus liraglutide	–	–	−0.3 (−0.6 to −0.1), *P* = 0.0113
Change from week 3 to week 6 within treatment group	0 (−0.1 to 0.2), *P* = 0.9303	−0.2 (−0.3 to 0), *P* = 0.0175	−0.2 (−0.3 to −0.1), *P* = 0.0099
**FCQ-S overall score**			
Baseline	2.9 (0.6)	3.0 (0.7)	2.9 (0.8)
Change at week 3	−0.1 (0.1)	−0.4 (0.1)	−1.0 (0.1)
Versus placebo	–	−0.3 (−0.6 to 0), *P* = 0.0282	−0.9 (−1.2 to −0.6), *P* < 0.0001
Versus liraglutide	–	–	−0.6 (−0.9 to −0.3), *P* = 0.0005
Change at week 6	−0.1 (0.1)	−0.7 (0.1)	−1.2 (0.1)
Versus placebo	–	−0.6 (−0.9 to −0.3), *P* = 0.0005	−1.1 (−1.4 to −0.8), *P* < 0.0001
Versus liraglutide	–	–	−0.5 (−0.9 to −0.2), *P* = 0.0011
Change from week 3 to week 6 within treatment group	0 (−0.2 to 0.2), *P* = 0.7864	−0.3 (−0.4 to −0.1), *P* = 0.0049	−0.2 (−0.4 to −0.1), *P* = 0.0150
**PFS overall score**			
Baseline	3.0 (0.8)	2.9 (0.8)	3.2 (0.9)
Change at week 3	0 (0.1)	−0.3 (0.1)	−1.0 (0.1)
Versus placebo	–	−0.3 (−0.6 to 0), *P* = 0.0273	−1.0 (−1.3 to −0.7), *P* < 0.0001
Versus liraglutide	–	–	−0.7 (−1.0 to −0.4), *P* < 0.0001
Change at week 6	−0.2 (0.1)	−0.6 (0.1)	−1.2 (0.1)
Versus placebo	–	−0.5 (−0.8 to −0.2), *P* = 0.0036	−1.0 (−1.3 to −0.7), *P* < 0.0001
Versus liraglutide	–	–	−0.5 (−0.9 to −0.2), *P* = 0.0019
Change from week 3 to week 6 within treatment group	−0.2 (−0.3 to 0), *P* = 0.0429	−0.3 (−0.5 to −0.2), *P* = 0.0001	−0.2 (−0.3 to 0), *P* = 0.0545
**BIS total score**			
Baseline	2.3 (0.2)	2.3 (0.2)	2.3 (0.2)
Change at week 3	0.08 (0.03)	0.04 (0.03)	−0.02 (0.03)
Versus placebo	–	−0.04 (−0.11 to 0.03), *P* = 0.2414	−0.10 (−0.17 to −0.02), *P* = 0.0098
Versus liraglutide	–	–	−0.06 (−0.13 to 0.02), *P* = 0.1352
Change at week 6	0.06 (0.03)	0.03 (0.03)	−0.04 (0.03)
Versus placebo	–	−0.04 (−0.11 to 0.04), *P* = 0.3167	−0.10 (−0.17 to −0.03), *P* = 0.0084
Versus liraglutide	–	–	−0.06 (−0.14 to 0.01), *P* = 0.0868

Data are mean (standard deviation) at baseline, least squares mean (standard error) change from baseline, difference in least squares mean (95% CI) versus placebo and liraglutide, change from week 3 to week 6 (95% CI) and median percentage change for energy intake. Statistical tests were conducted using a MMRMs unless stated otherwise. *n*, number of participants who were randomized and received at least one dose of study treatment.

^a^A higher overall appetite score indicates less appetite and a lower score indicates more appetite.

Appetite over the previous week as assessed by retrospective VAS showed similar results to fasting appetite (Supplementary Table 3). Tirzepatide decreased retrospective appetite versus placebo at week 3.

Tirzepatide decreased Food Craving Inventory (FCI) overall score and cravings for high-fat foods, sweets, carbohydrates or starches and fast-food fats (high in fat and calories and easily accessible; for example, pizza, hamburgers and French fries), but not fruits and vegetables, versus placebo at week 3 (Fig. 3b, Table 2 and Supplementary Table 4).

Tirzepatide decreased Food Craving Questionnaire-State (FCQ-S) overall score versus placebo at week 3, as well as all subscale scores (physiological state: hunger, lack of control over eating, anticipation of negative reinforcement from eating, anticipation of positive reinforcement and intense desire to eat; Fig. 3c, Table 2 and Supplementary Table 5), reflecting a greater reduction in state-dependent food craving.

Tirzepatide decreased Power of Food Scale (PFS) overall score and all subscale scores (food available, food present and food tasted; Fig. 3d, Table 2 and Supplementary Table 6) versus placebo at week 3 suggesting less responsiveness to proximal foods and greater control of appetite.

Tirzepatide decreased Eating Inventory perceived hunger versus placebo at week 3 (Fig. 3e and Table 2). Tirzepatide also decreased disinhibition versus placebo at week 3, reflecting a greater reduction in the tendency to overeat in response to external (for example, the sight and smell of food) and internal (for example, stress and anxiety) cues. There was no statistically significant difference for change in cognitive restraint at week 3 between tirzepatide and placebo.

### Safety

Treatment-emergent AEs were reported by 30 (81%) participants in the tirzepatide group, 25 (66%) in the liraglutide group and 17 (44%) in the placebo group (Extended Data Table 1). There were seven discontinuations due to AEs: three (8%) in the tirzepatide group because of nausea, pancreatitis and vomiting; two (5%) in the liraglutide group because of COVID-19 pneumonia and vomiting and two (5%) in the placebo group because of urticaria and increased appetite. There was one serious AE in the liraglutide group (COVID-19 pneumonia). No deaths were reported during the study. There were no confirmed hypoglycemic events during the study.

**Extended Data Fig. 3:**
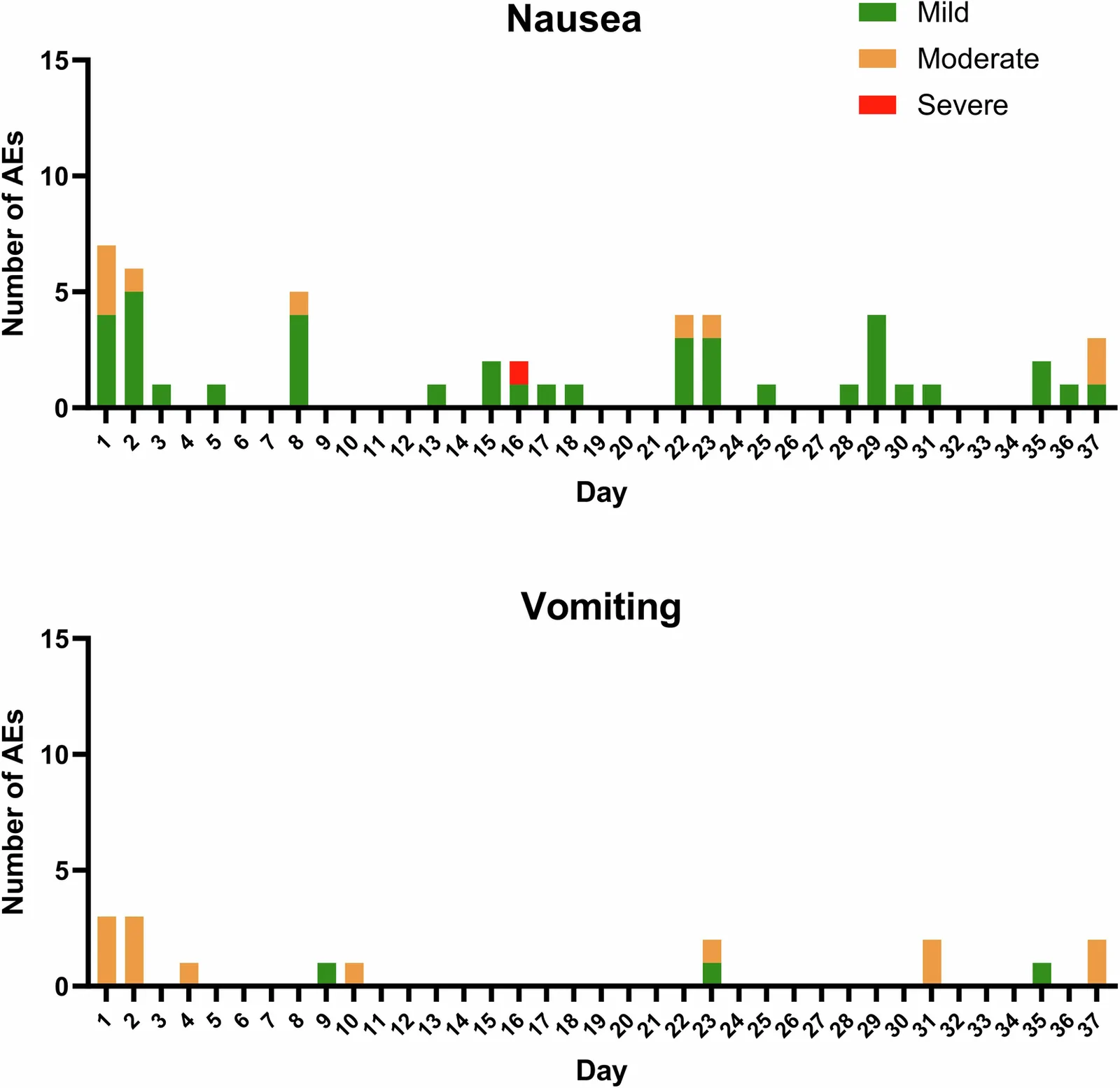
Number of nausea and vomiting AEs reported by participants receiving tirzepatide over time. AEs were classed as mild (shown in green), moderate (shown in orange), or severe (shown in red) in all participants randomized to tirzepatide (n = 37). AE=adverse events. Source data

The most common treatment-emergent AEs with tirzepatide were gastrointestinal (nausea, vomiting, dyspepsia and constipation). Most nausea and vomiting AEs were mild to moderate in severity and a higher proportion happened after the first injection compared to each subsequent injection (nausea: 15 events of 49 total throughout the study; vomiting: 7 events of 16 total) (Extended Data Fig. 3). On the days of the procedures, day 16 and day 37, we observed 2 (1 mild, 1 severe) and 3 (1 mild, 2 moderate) nausea AEs, respectively, and 0 and 2 (2 moderate) vomiting AEs, respectively, suggesting that any effects of nausea and vomiting in the tirzepatide group on assessments were limited.

Nausea, malaise and gastrointestinal distress over the previous week was monitored using retrospective VAS. For tirzepatide versus placebo, there were larger increases in nausea and gastrointestinal distress at week 3 and week 6, and in malaise at week 6 (Supplementary Table 3). For liraglutide versus placebo, there was a larger increase in nausea at week 3 and week 6, and gastrointestinal distress at week 6. For tirzepatide versus liraglutide, there was a larger increase in nausea at week 6.

With tirzepatide, liraglutide and placebo, mean body weight changes from baseline to week 3 were −3.7 kg, −1.9 kg and −0.5 kg, respectively, and changes to week 6 were −7.0 kg, −3.7 kg and −0.6 kg, respectively (Table 2). There was a greater decrease from baseline to week 3 and to week 6 in body weight with tirzepatide versus placebo and liraglutide, and with liraglutide versus placebo (Fig. 2c).

### Exploratory outcomes

Tirzepatide reduced energy intake during the lunch meal test at week 3 versus liraglutide. The reduction in energy intake with tirzepatide was sustained at week 6 versus both placebo and liraglutide. Liraglutide reduced energy intake at week 3 and week 6 versus placebo.

**Extended Data Fig. 4:**
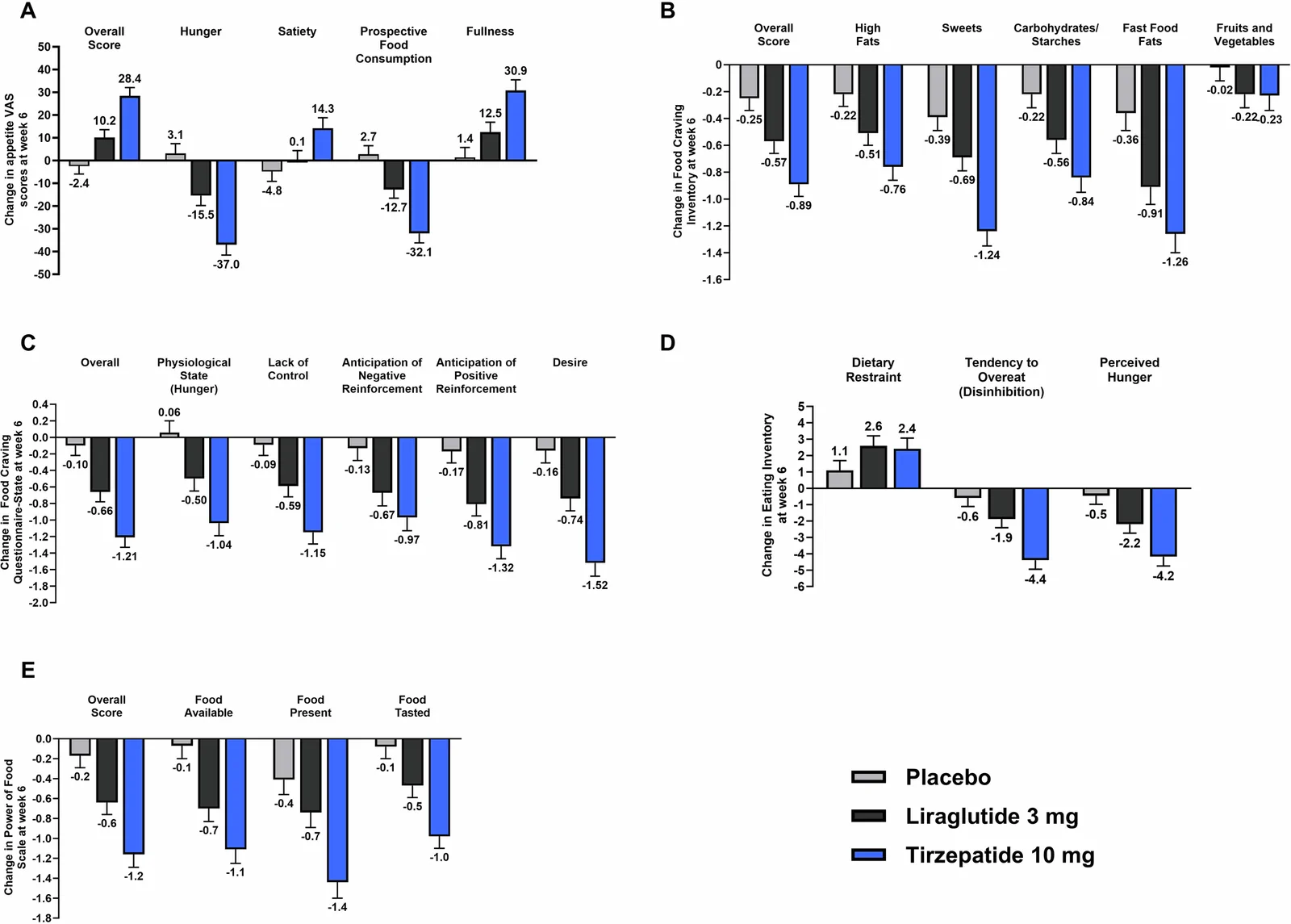
Changes in ingestive behaviour at week 6. Data are least squares mean (standard error) from mixed-model repeated measures for change in **A**) pre-lunch appetite VAS scores, **B**) Food Craving Inventory, **C**) Food Craving Questionnaire-State, **D**) Eating Inventory, and **E**) Power of Food Scale, at week 6 in all randomized participants (placebo, n = 39; tirzepatide, n = 37; liraglutide, n = 38). VAS=visual analogue scale. Source data

Tirzepatide decreased fasting VAS overall appetite at week 3 versus liraglutide. Tirzepatide decreased fasting hunger and prospective food consumption, and increased fasting satiety and fullness, versus liraglutide at week 3. Differences between tirzepatide and both placebo and liraglutide at week 3 were sustained at week 6 (Extended Data Fig. 4). With liraglutide, fasting overall appetite change did not differ from placebo at week 3, but decreased versus placebo at week 6. Tirzepatide decreased fasting desire to eat sweet, salty and savory foods at week 3 versus liraglutide. Differences between tirzepatide and placebo or liraglutide generally persisted at week 6. There were no differences between liraglutide and placebo for any food group. Treatment groups did not differ in postprandial appetite changes at either week 3 or week 6.

Tirzepatide decreased retrospective appetite at week 3 versus liraglutide. Differences between tirzepatide and both placebo and liraglutide at week 3 were sustained at week 6. With liraglutide, retrospective appetite decreased at week 3 and week 6 versus placebo.

The differences between tirzepatide and placebo in cravings for high-fat foods, sweets, carbohydrates or starches and fast-food fats, as well as overall cravings at week 3, were sustained at week 6. Tirzepatide decreased craving overall and for sweets and fast-food fats at week 3 versus liraglutide but at week 6 only sweet cravings differed from liraglutide. With liraglutide, there were no differences in food craving versus placebo at week 3, but at week 6 cravings for all categories except for fruits and vegetables decreased versus placebo.

Tirzepatide decreased FCQ-S overall score at week 3 versus liraglutide, as well as all subscale scores. Differences between tirzepatide and both placebo and liraglutide at week 3 were sustained at week 6 except for negative reinforcement from eating versus liraglutide. With liraglutide, overall score, but not subscale scores, decreased versus placebo at week 3 and all scores decreased versus placebo at week 6.

Tirzepatide decreased PFS overall score and all subscale scores versus liraglutide at week 3, suggesting less responsiveness to proximal foods and greater control of appetite. These differences between tirzepatide and both placebo and liraglutide at week 3 were sustained at week 6. With liraglutide, overall score and food available subscale scores decreased versus placebo at week 3 and week 6, when liraglutide also reduced the food tasted score.

On the Eating Inventory, tirzepatide decreased perceived hunger and disinhibition versus liraglutide at week 3. These differences between tirzepatide and both placebo and liraglutide at week 3 were sustained at week 6. With liraglutide, perceived hunger and disinhibition decreased versus placebo at week 6, but not at week 3. Only liraglutide increased reported cognitive restraint versus placebo at week 3 and there were no statistically significant treatment group differences for change in cognitive restraint at week 6.

**Extended Data Fig. 5:**
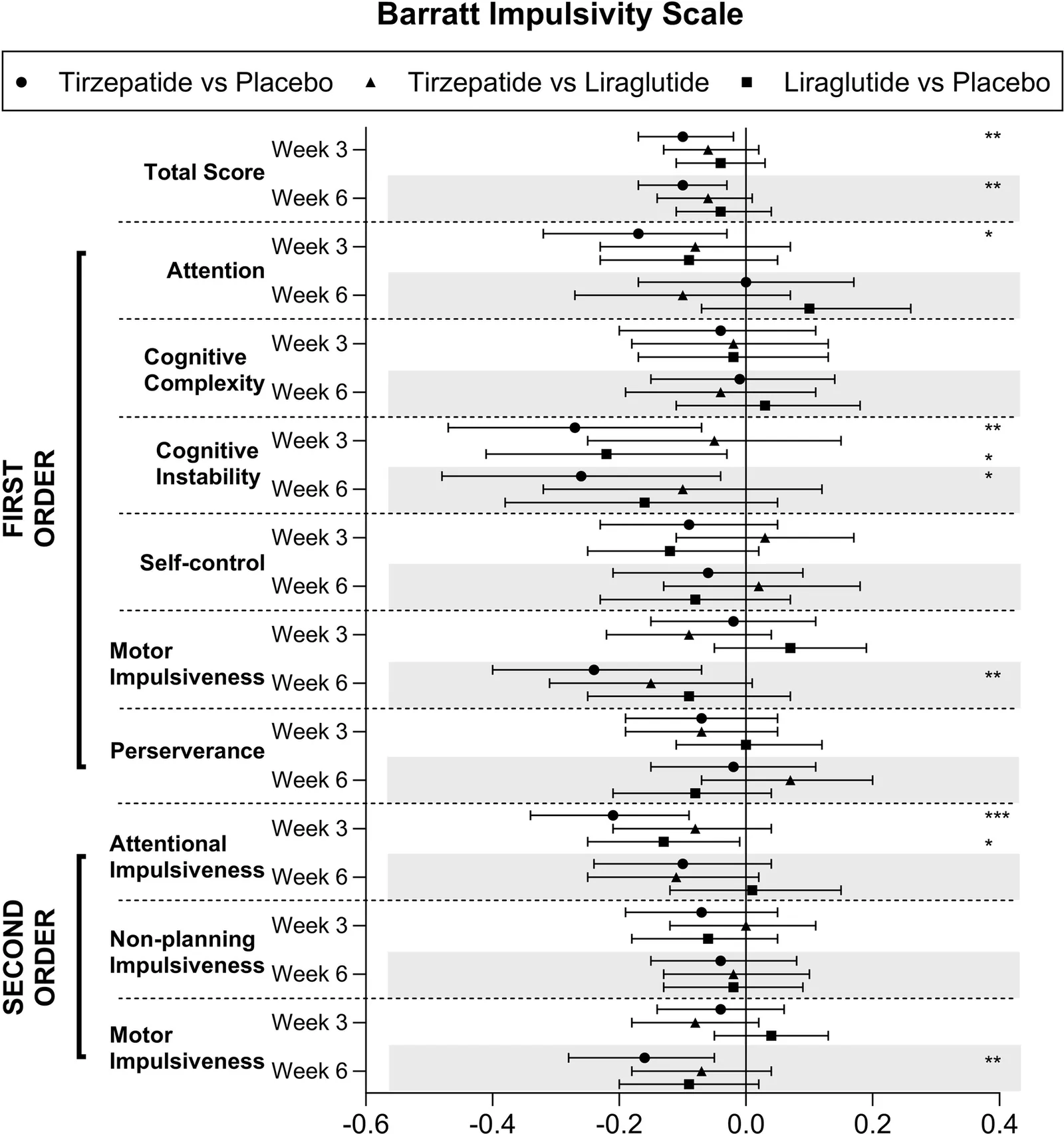
Differences between treatment groups in changes in Barratt Impulsiveness Scale scores. Data are presented as mean ETD (centre) and associated 95% CIs (whiskers) for change in Barratt Impulsiveness Scale scores. ETD was estimated using an MMRM in all randomized participants (placebo, n = 39; tirzepatide, n = 37; liraglutide, n = 38). Statistical tests were two-sided at a significance level of 0.05, and no adjustments were made for multiplicity. *p < 0.05, **p < 0.01, ***p < 0.001 for comparisons between treatment groups. Statistical comparisons including exact p-values are provided in Table S7. ETD = estimated treatment difference. Source data

Tirzepatide decreased Barratt Impulsiveness Scale (BIS) total impulsiveness score versus placebo at week 3 and week 6 (Table 2 and Fig. 3f). Tirzepatide decreased cognitive instability and attentional impulsiveness versus placebo at week 3, and cognitive instability and motor impulsiveness versus placebo at week 6 (Supplementary Table 7 and Extended Data Fig. 5). There was an increase in attention with tirzepatide versus placebo at week 3 but change in attention was similar at week 6. Tirzepatide and liraglutide groups did not differ on changes in any scores at either week 3 or week 6. Liraglutide decreased cognitive instability and attentional impulsiveness versus placebo at week 3 but did not differ from placebo on any scores at week 6.

**Fig. 4:**
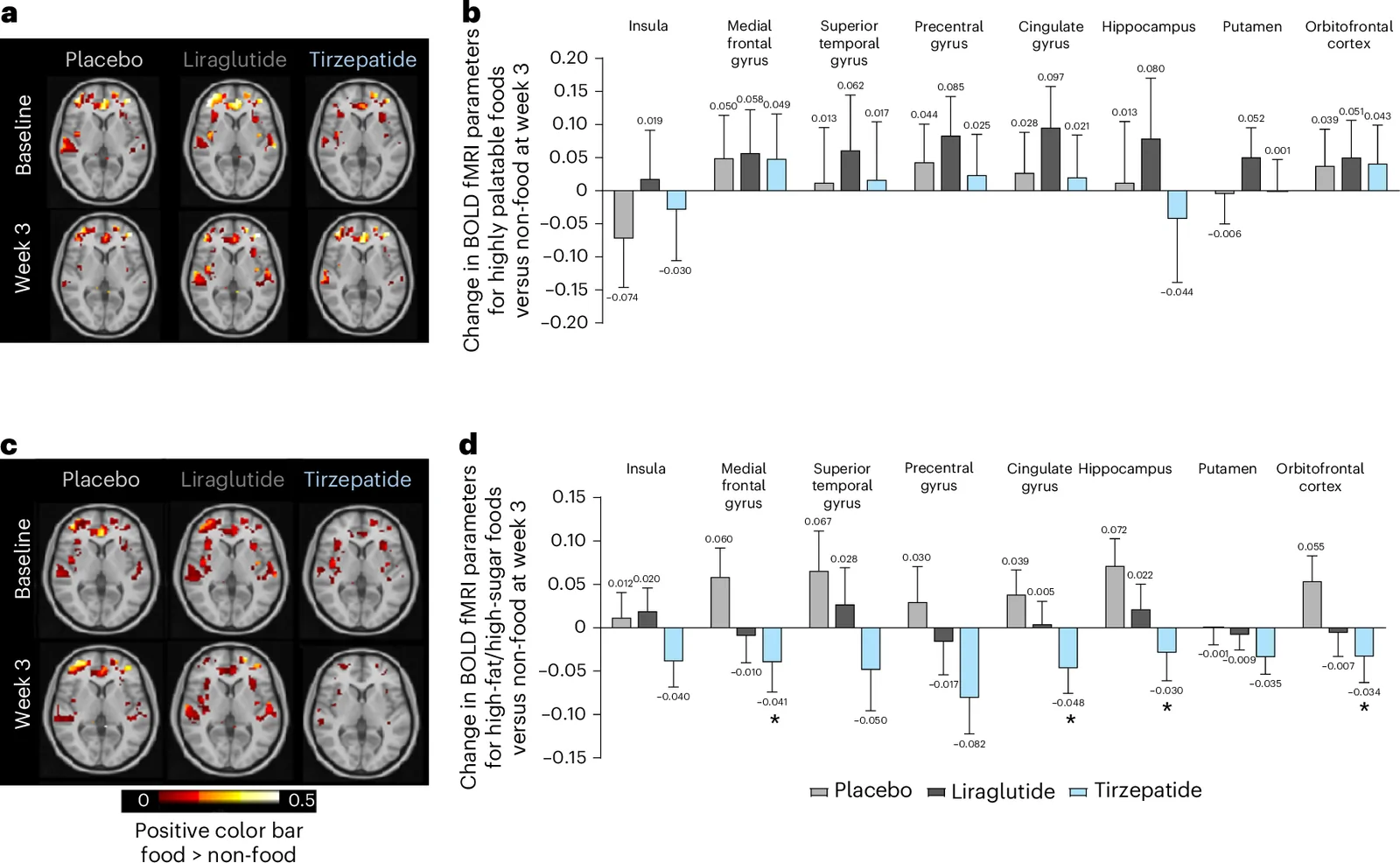
Effects on brain activation in response to food cues at week 3 as assessed by BOLD fMRI. **a**,**b**, Mean images of brain activation at baseline and week 3 (**a**) and least squares mean (standard error) change in BOLD fMRI parameters for highly palatable foods versus non-food objects at week 3 (**b**). **c**,**d**, Mean images of brain activation at baseline and week 3 (**c**) and least squares mean (standard error) change in BOLD fMRI parameters for high-fat, high-sugar foods versus non-food objects at week 3 (**d**). For each scan and each region, the mean of positive voxels was taken within each of the regions separately. The least squares mean was estimated using a MMRMs in all randomized participants who had available data (placebo, *n* = 33; tirzepatide, *n* = 31; liraglutide, *n* = 34). Statistical tests were two-sided at a significance level of 0.05 and no adjustments were made for multiplicity. No positive voxels (food > non-food) were identified for the ventral striatum; therefore, statistical analysis was not done on this region. ^*^P < 0.05 versus placebo (high-fat, high-sugar foods versus non-food objects: medial frontal gyrus, *P* = 0.0335; cingulate gyrus, *P* = 0.0306; hippocampus, *P* = 0.0221; orbitofrontal cortex, *P* = 0.0321). Source data

**Extended Data Fig. 6:**
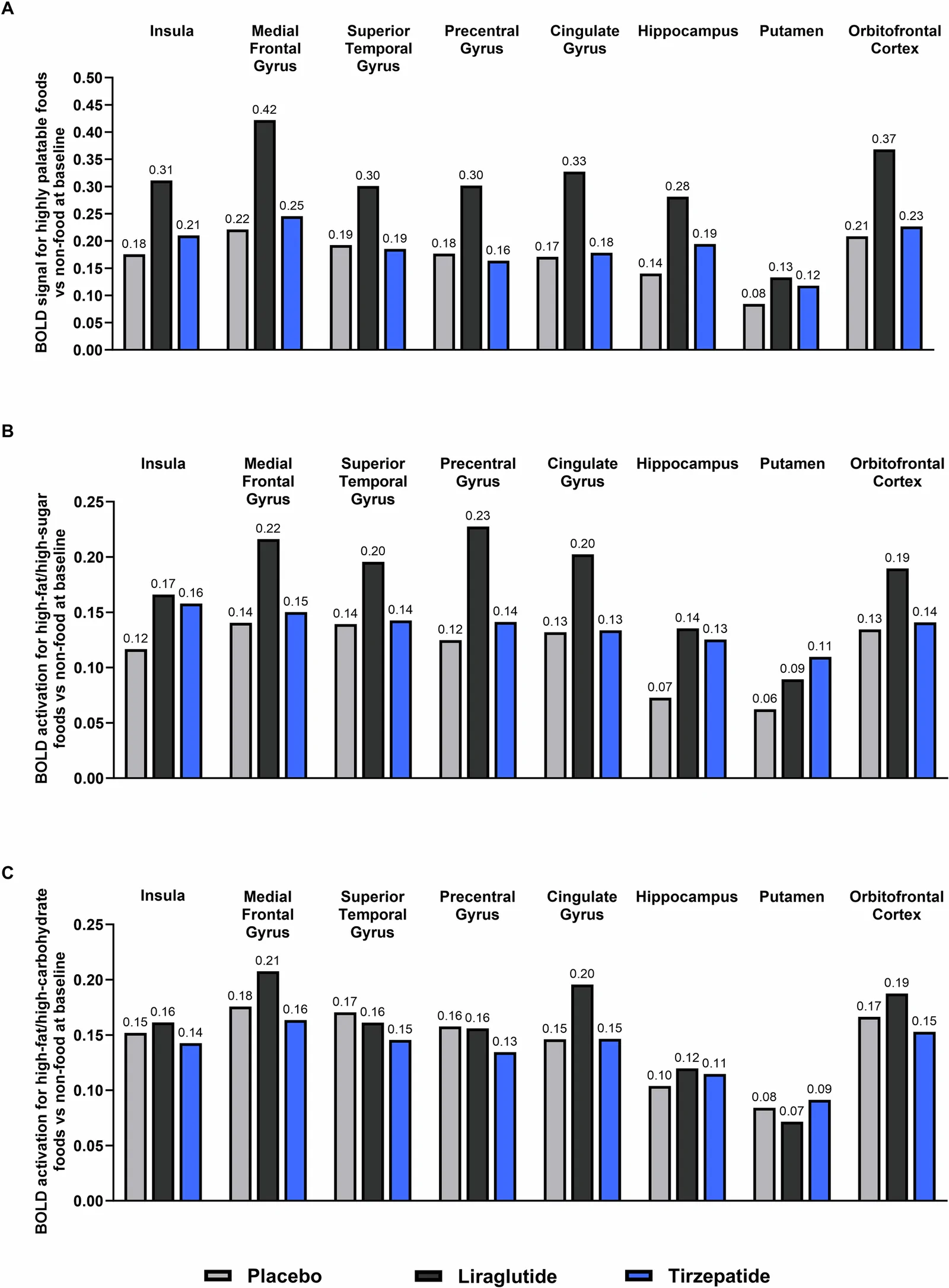
Baseline brain activation as assessed by BOLD fMRI. Mean BOLD fMRI parameter activation at baseline for **A**) highly palatable foods vs non-food objects, **B**) high-fat/high-sugar foods vs non-food objects, and **C**) high-fat/high-carbohydrate foods vs non-food objects in all randomized participants who had available data (placebo, n = 33; tirzepatide, n = 31; liraglutide, n = 34). For each scan and each region, the mean of positive voxels was taken within each of the regions separately. No positive voxels (Food > Non-food) were identified for the ventral striatum; therefore, statistical analysis was not done on this region BOLD=blood oxygenation level dependent; fMRI=functional magnetic resonance imaging. Source data

**Extended Data Fig. 7:**
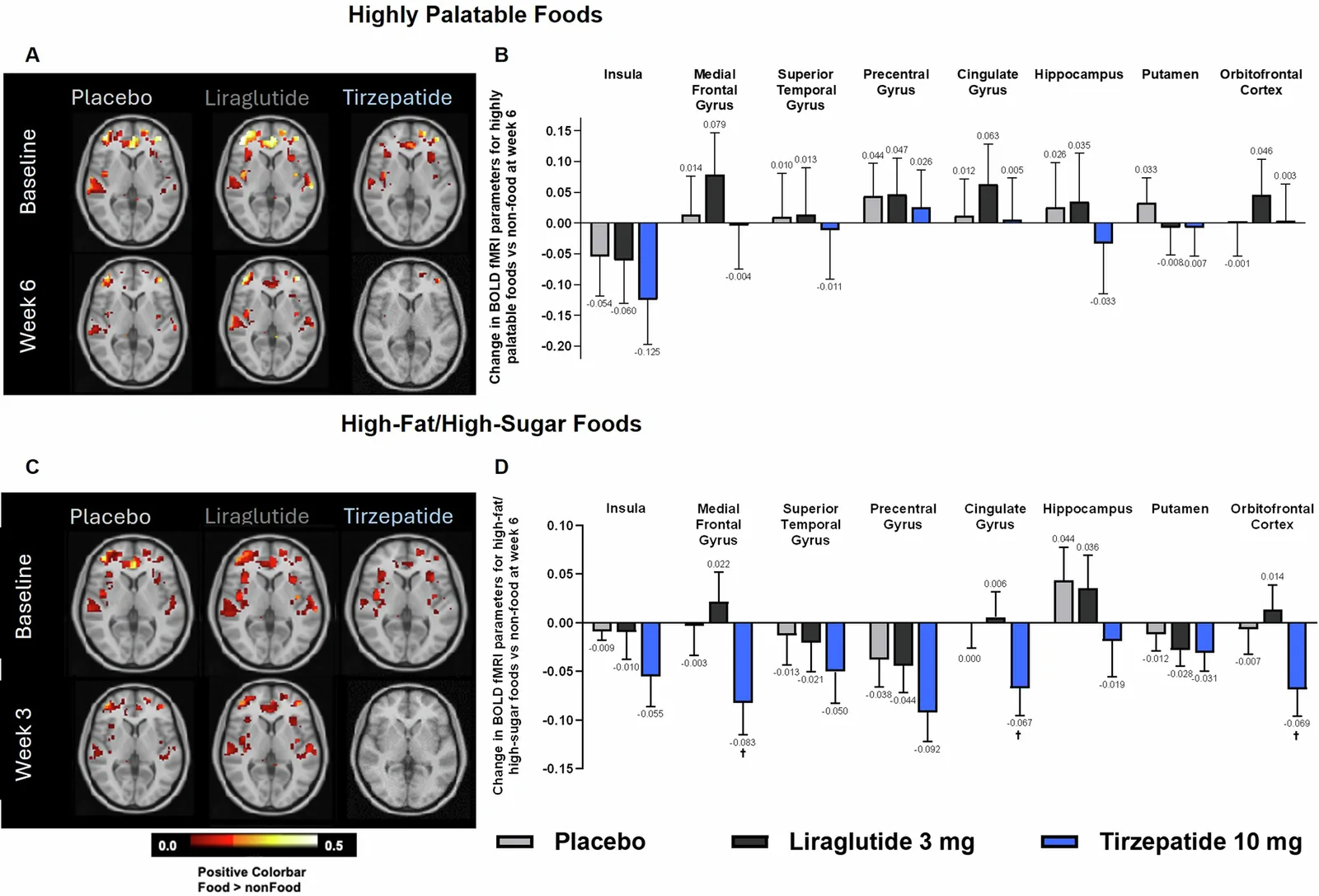
Effects of highly palatable and high-fat/high-sugar foods versus non-food objects on brain activation at week 6 as assessed by BOLD fMRI. **A**) Mean images of brain activation at baseline and week 6 and **B**) least squares mean (standard error) change in BOLD fMRI parameters for highly palatable foods vs non-food objects at week 6. **C**) mean images of brain activation at baseline and week 6 and **D**) least squares mean (standard error) change in BOLD fMRI parameters for high-fat/high-sugar foods vs non-food objects at week 6. For each scan and each region, the mean of positive voxels was taken within each of the regions separately. The least squares mean was estimated using an MMRM in all randomized participants who had available data (placebo, n = 33; tirzepatide, n = 31; liraglutide, n = 34). Statistical tests were two-sided at a significance level of 0.05, and no adjustments were made for multiplicity. No positive voxels (Food > Non-food) were identified for the ventral striatum; therefore, statistical analysis was not done on this region. †p < 0.05 vs liraglutide (medial frontal gyrus, p = 0.0156; cingulate gyrus, p = 0.0486; orbitofrontal cortex, p = 0.0219). BOLD=blood oxygenation level dependent; fMRI=functional magnetic resonance imaging. Source data

**Extended Data Fig. 8:**
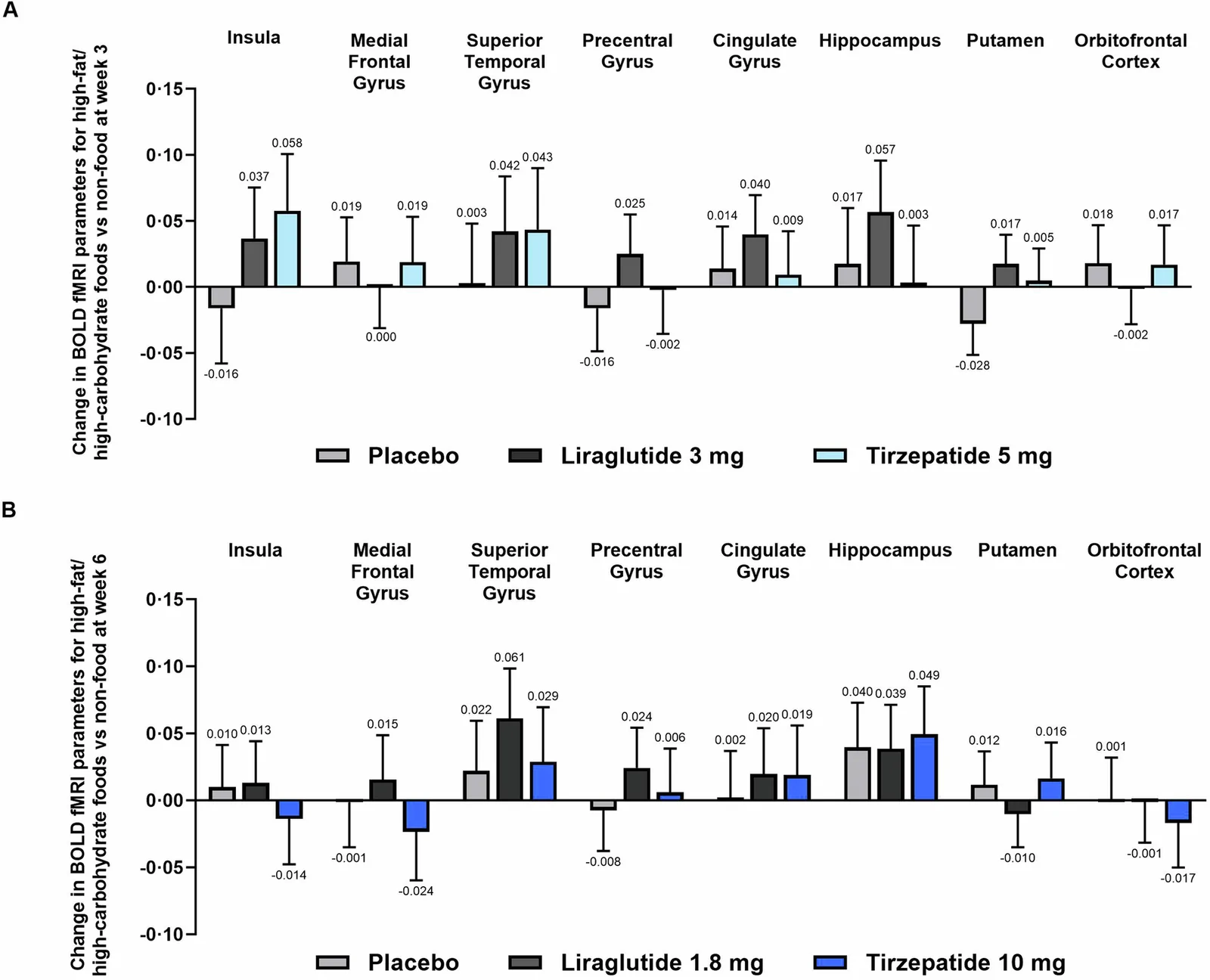
Effects of high-fat/high-carbohydrate food versus non-food objects on brain activation at week 3 and week 6 as assessed by BOLD fMRI. Least squares mean (standard error) change in BOLD fMRI parameters for high-fat/high-carbohydrate food vs non-food objects **A**) at week 3 and **B**) at week 6. For each scan and each region, the mean of positive voxels was taken within each of the regions separately. The least squares mean was estimated using an MMRM in all randomized participants who had available data (placebo, n = 33; tirzepatide, n = 31; liraglutide, n = 34). Statistical tests were two-sided at a significance level of 0.05, and no adjustments were made for multiplicity. No positive voxels (Food > Non-food) were identified for the ventral striatum; therefore, statistical analysis was not done on this region. BOLD=blood oxygenation level dependent; fMRI=functional magnetic resonance imaging. Source data

Mean BOLD fMRI activation while viewing photographs of food versus non-food objects at baseline is shown in Extended Data Fig. 6. Change in highly palatable food (Food_HiPal_) BOLD fMRI activation from baseline to week 3 or week 6 did not differ between treatment groups within any region of interest (ROI) (Fig. 4a, Extended Data Fig. 7A and Supplementary Table 8). At week 3, with tirzepatide versus placebo, high-fat, high-sugar food (Food_HiF/HiS_) activation decreased within medial frontal gyrus, cingulate gyrus, hippocampus and orbitofrontal cortex (Fig. 4b and Supplementary Table 9). At week 6, with tirzepatide versus liraglutide (but not placebo), Food_HiF/HiS_ activation decreased within medial frontal gyrus, cingulate gyrus and orbitofrontal cortex (Extended Data Fig. 7B). For liraglutide versus placebo, there were no differences in activation to Food_HiF/HiS_. Change in high-fat, high-carbohydrate (Food_HiF/HiC_) activation from baseline to week 3 or week 6 did not differ between treatment groups within any ROI (Extended Data Fig. 8 and Supplementary Table 10). Change in activation to other food group categories did not differ between treatment groups within any ROI (Supplementary Tables 11–14).

### Sensitivity analyses

Tirzepatide decreased energy intake at the ad libitum lunch to a numerically greater extent in both participants who experienced and those who did not experience any nausea or vomiting AEs compared to placebo and liraglutide (Supplementary Table 15). A trend toward a slightly higher reduction in energy intake was observed for those who experienced nausea or vomiting in the tirzepatide group but the opposite was observed in the liraglutide group. Only two participants reported nausea or vomiting in the placebo group. The overall study conclusions were similar when participants who experienced nausea or vomiting were excluded.

### Post hoc analyses

Tirzepatide was associated with decreased intake of all macronutrients (fat, carbohydrate and protein) at the ad libitum lunch versus placebo and liraglutide at both week 3 and week 6 (Supplementary Table 16). Liraglutide was associated with decreased intake of all macronutrients compared to placebo at both week 3 and week 6.

## Discussion

We investigated early adaptative changes in appetite, ingestive behavior and brain appetite circuits during tirzepatide treatment. Tirzepatide reduced energy intake and was associated with reduced intake of all three macronutrients at the ad libitum lunch at week 3 compared with placebo and liraglutide. At week 6, participants in the tirzepatide group had a 72% reduction in energy intake at lunch compared to baseline. BOLD activation to high-fat, high-sugar foods in CNS regions modulating appetite decreased with tirzepatide compared to placebo at week 3 and compared to liraglutide at week 6. In the fasting state, when the drive to eat is enhanced, tirzepatide decreased overall appetite and affected multiple domains of appetite and ingestive behavior, with the exception of cognitive restraint. No selective changes in food preferences were detected, as food intake reductions were similar across macronutrients.

Previous studies using self-report inventories with selective GLP-1 RAs, liraglutide and semaglutide have reported effects on ingestive behavior^7,19,20^. One study in adults with obesity found that 16 weeks of liraglutide treatment reduced prospective food consumption and desire for sweet, salty, savory or fatty foods, and increased fullness, relative to placebo^20^. Another reported that 12 weeks of semaglutide treatment decreased overall fasting appetite, fullness, hunger and prospective food consumption, but not fasting satiety, relative to placebo^7^. Semaglutide also lowered food cravings, particularly for savory foods, and explicit liking and implicit wanting for ‘high-fat and non-sweet’ foods but not ‘high-fat and sweet’ foods^7^. In the present study, tirzepatide decreased energy intake, appetite, food cravings, disinhibition and reactivity to the food environment in the fasting state, and increased satiety and fullness as early as week 3 compared to placebo and liraglutide. Tirzepatide was also associated with reduced intake of all three macronutrients. There were no differences in VAS changes postprandially between groups. This, combined with differences in the energy intake between groups during the ad libitum lunch, suggests that participants in all groups stopped their meal when they felt comfortably full rather than for other reasons while still being hungry. These findings are consistent with previous research that found decreases in food intake and fasting or premeal appetite ratings, while postprandial ratings remained largely the same^21^.

Tirzepatide reduced food cravings for all food groups (high-fats, sweets, carbohydrates and starches, and fast-food fats) except fruits and vegetables relative to placebo, and cravings for sweets relative to liraglutide. We observed in a separate study that tirzepatide reduced overall energy intake during lunch and dinner without selectively decreasing intake of certain macronutrients, and these findings were supported by the reduction in cravings for foods across all but one food group (fruits and vegetables)^16^. These findings, which are consistent with the current study, demonstrate how tirzepatide reduces appetite and the drive to eat foods from almost all food categories.

Taken together, our findings indicate that tirzepatide effectively reduced drive to eat, as well as food-cue-driven and other-cue-driven eating behavior. It also seems that the impact of tirzepatide on modulating appetite and cravings may be greater and potentially more sustained than that of selective GLP-1 RAs. This may be due to the GIP receptor activity of tirzepatide, as shown in preclinical studies^22^. Although there are no approved selective GIP RAs in clinical use, studies comparing tirzepatide to selective GIP RAs may provide additional insights into the contribution of GIP receptor activation in humans. Importantly, tirzepatide was not found to affect cognitive restraint, which may distinguish it from other weight reduction interventions where increased volitional cognitive restraint appears to be an important component in restricting energy intake to lose weight^23–25^.

Evidence suggests that some individuals seeking weight loss, for example, those with binge eating behaviors^26,27^, experience behavioral symptoms (cravings and continued use despite harm) and neurological mechanisms (disruptions of inhibitory control and reward sensitivity) that parallel substance use disorders^28^. Selective GLP-1 RAs have been shown to reduce impulsive and addictive behaviors in preclinical models, including intake of alcohol and drugs of abuse^29,30^. In human retrospective cohort studies, semaglutide reduced alcohol, tobacco^31^ and cannabis^32^ use. Here we report that tirzepatide reduced self-reported impulsiveness, as did liraglutide for some factors. This suggests that these compounds may reduce impulsiveness more generally, though further data on GLP-1 RA effects for impulsive choices based on craving for other substances must be evaluated.

CNS responses to food following tirzepatide treatment are critical for understanding the effects of tirzepatide on ingestive behavior. Evidence suggests that activation within brain reward and appetite regions in response to food cues, measured using BOLD fMRI, may influence eating behavior following weight reduction leading to weight regain^33–35^. Earlier GLP-1 RA fMRI studies have reported short-term reductions in brain responses to food cues localized to the amygdala and insula with exenatide^8,9^, and to the insula and putamen with liraglutide^10^. Conversely, longer-term use of liraglutide has been associated with increases in orbitofrontal activation^13^, or attenuation of short-term treatment effects on brain responses^11,12^. The 3- and 6-week assessments in the present study align closely to the assessment time points in two of these previous liraglutide studies, which showed differential impacts of liraglutide on brain responses to food cues after 17 days^10^ and 5 weeks of treatment^13^.

In this study, tirzepatide reduced brain activation in response to Food_HiF/HiS_ cues in the orbitofrontal cortex, cingulate gyrus, hippocampus and medial frontal gyrus. The orbitofrontal cortex plays a role encoding satiety and food reward value^36–39^, and functional connectivity of the orbitofrontal cortex to other regions is associated with differential preferences for sweet foods and positively correlated with body mass index (BMI)^28^. Sweet-taste-related activation of the anterior cingulate gyrus has been associated with sweet food intake at a subsequent ad libitum test meal^40,41^. The parahippocampal gyrus is involved in hedonic feeding and incentive motivation processes driven by emotional memory^28^. The medial frontal gyrus plays a role in voluntary behavior, either self-generated or self-reflective^42–45^. Modulation of activity in these regions, all of which are implicated in the regulation of food intake, could therefore plausibly play a role in the effectiveness of tirzepatide.

Prior studies of weight loss by caloric restriction have identified treatment effects on CNS responses to food cues in the orbitofrontal cortex, medial frontal gyrus and anterior cingulate gyrus^46,47^. Bariatric surgery studies have identified reductions on food cue responses in medial frontal and orbitofrontal regions^48^. Convergence in affected regions between these differing weight loss modalities suggest a common neurobiological pathway for weight loss treatment effects. However, unlike tirzepatide and surgery, caloric restriction led to increases rather than decreases in food cue responses, likely due to the neuroendocrine modulation that is specific to tirzepatide and surgery-related weight loss, but not to caloric restriction. Considering our results in the context of these findings, we speculate that tirzepatide impacts activation within brain regions implicated in the motivating value of foods, particularly highly palatable high-fat, high-sugar foods, contributing to lower energy intake. The findings of tirzepatide fMRI effects in response to Food_HiF/HiS_ cues is also supported by findings of tirzepatide effects on self-reported cravings for sweets, fats and fast-food fats with the FCI. Notably, the Food_HiF/HiS_ category of the Macronutrient Picture System paradigm (for example, cakes, cookies and candy bars)^49^ is very similar to the ‘sweets’ FCI subscale (for example, brownies, cookies, candy and chocolate). These findings align with both preclinical studies using high-fat diets, which are high in both fat and sugar, and human fMRI studies describing neurobehavioral impacts of high-fat, high-sugar diets in implicating reward system responses to high-fat, high-sugar stimuli as a key phenotype contributing to overeating and weight gain^50,51^.

It is important to interpret the robustness of the fMRI findings in the context of the multiple comparisons that were performed across stimulus contrasts, regions and time points. While there were reductions in activation with tirzepatide relative to placebo in response to Food_HiF/HiS_ cues in several ROIs at week 3, there were no differences relative to placebo at week 6. Though numerically larger reductions in activation relative to baseline at week 6 were observed in the tirzepatide group compared to week 3, we speculate that differences between tirzepatide and placebo may have been attenuated due to task habituation, since activation was reduced relative to baseline in the placebo group at week 6 in most ROIs. Given the statistical limitations of the findings due to the nature of multiple comparisons, voxel-wise analyses and further fMRI studies are needed to support, replicate and extend our findings.

Study strengths include the randomized trial design comprising a blinded placebo control and an active GLP-1 RA control, liraglutide. BOLD fMRI harmonization among several sites was successfully implemented, assessed and monitored by an external MRI organization. This study employed non-invasive dynamic recordings of brain activity and examined ingestive behavior and regional brain activation with tirzepatide in humans.

There were several study limitations. First, the absence of a blinded comparison to a long-acting GLP-1 RA. Once-daily liraglutide 1.8 and 3 mg was selected as the GLP-1 RA control instead of once-weekly semaglutide because of prior fMRI data for liraglutide^11–13^ and an approved titration scheme^52^ compatible with our study duration, unlike the 3 to 5 months required for semaglutide 1 mg or 2.4 mg (ref. ^53^). Liraglutide was open-label due to differences in autoinjectors and daily administration schedule. Second, a lack of sex parity in the study, a common occurrence in obesity trials^54^, albeit one that reflects the treatment population. There was also an imbalance in sex across treatment groups, with more males in the liraglutide group (34%) compared to the other groups (<10% in each). This study was not designed to test the effects of sex but future studies into the potential effects of sex on the effect of tirzepatide on energy intake and eating behaviors are warranted. Third, this study was principally designed to detect treatment effect of the primary outcome. Since type 1 error rate adjustments were not made for multiple comparisons, the results of these multiple comparisons were evaluated with consideration of consistency with findings within the study, as well as those from literature, and their interpretation should be taken with caution. Fourth, highly palatable foods were selected based on US food preferences that may not be generalizable to other nations. We also acknowledge individual variation in perceived palatability that our design was unable to capture. Palatability of food was not specifically tested. Fifth, use of the same food cue stimuli at each fMRI visit creates a potential order effect, as participants may habituate with repeated exposure, potentially reducing ability to detect treatment effects at later follow ups, though all groups would experience such habituation. Sixth, this study was of shorter duration than efficacy trials. Studies with longer-term therapy at maximum maintenance doses could elucidate further changes in ingestive behaviors while minimizing contributions of gastrointestinal effects and examine potential habituation and brain plasticity effects with extended therapy. Seventh, although our findings suggest an added contribution from GIP receptor agonism, since neither a selective GIP RA or antagonist are currently available for use in humans, it was not possible to differentiate between neurobiological effects of GIP and GLP-1 receptor activation.

Pharmacotherapies like GLP-1 RAs and tirzepatide will likely be a strong contributor to the management of obesity. Understanding the neurobiological mechanisms that underpin tirzepatide’s impact on energy intake are critical for its effective use and in the development of future therapies. We found that after 3 weeks of treatment, tirzepatide reduced energy intake relative to placebo. One key mechanism underlying tirzepatide’s efficacy may be to modify ingestive behaviors by impacting responsivity of brain appetite circuits in particular toward high-fat and high-sugar foods.

## Methods

### Study design

This phase 1, randomized, parallel design, partially-blinded, placebo-controlled study with a positive control (liraglutide) was conducted at three study centers in the United States. The study consisted of four periods: a screening period of approximately 5 weeks, a 5-day lead-in period, a 6-week treatment period and a 4-week safety follow-up period (Fig. 1b). The study was approved by institutional review boards at each site (Pennington Biomedical Research Center Institutional Review Board, Human Research Protection Program Office of Research Compliance Indiana University and Johns Hopkins Institutional Review Board). The study was conducted in accordance with the Declaration of Helsinki and International Conference on Harmonization Good Clinical Practice guidelines. All participants provided written informed consent before participating in the study. This study is registered with ClinicalTrials.gov, NCT04311411.

Once-daily liraglutide 1.8 and 3 mg was selected as the GLP-1 RA control instead of a newer, once-weekly, selective GLP-1 RA, like semaglutide to investigate the early changes in ingestive behavior for the following reasons: available prior data for food intake, ingestive behavior questionnaires and fMRI^6,11–13^; and an approved titration scheme^52^, compatible with our study duration, unlike the 3 to 5 months required for semaglutide 1 mg or 2.4 mg (ref. ^53^).

### Participants

Male and female adults (18 to 65 years) were eligible for inclusion if they had a BMI of 27 to 50 kg per m^2^ and stable weight in the last month (no weight change of >4 kg). Exclusion criteria included diagnosis of any form of diabetes or glycated hemoglobin ≥6.5% at screening, and contraindications to undergoing MRI. Full inclusion and exclusion criteria are provided in the Supplementary Information. Patients were screened and enrolled irrespective of their sex. Sex was self-reported by participants.

### Randomization and masking

Participants were randomized 1:1:1 to receive tirzepatide, liraglutide or placebo, using a randomization table with treatment codes. Randomization was stratified by baseline BMI (27 to <30, 30 to <35 and 35 to 50 kg per m^2^) within each site. The sponsor, investigators and participants were blinded to tirzepatide and placebo treatment but liraglutide treatment was open-label. Therefore, the study was considered partially blinded.

### Procedures

Tirzepatide and placebo were administered subcutaneously once-weekly at the study centers. Tirzepatide was administered at 5 mg for the first 3 weeks followed by 10 mg for the next 3 weeks. Liraglutide was self-administered once-daily by participants at home for 38 days. Liraglutide was initiated at 0.6 mg and escalated weekly by 0.6 mg until a dose of 3 mg was reached at week 5 and maintained for the remainder of the study.

Assessments, including an energy intake test, ingestive behavior questionnaires and BOLD fMRI, were conducted at baseline (lead-in), week 3 and week 6. Energy intake was assessed by a clinic-based, multi-item, ad libitum lunch test meal. Multiple dimensions of ingestive behavior were assessed by questionnaires. Subjective appetite was assessed via VAS before and after the test meal (participants rated feelings at that moment) and retrospectively over the study (participants rated average feelings over the previous week). The appetite VAS questionnaire measured hunger, satiety, fullness, prospective food consumption (questions 1 to 4) and desire for specific food groups (questions 5 to 8). An overall appetite score was calculated as the average of the four individual scores for questions 1 to 4. A higher overall appetite score indicated lower appetite. We also assessed cravings for specific food types via the FCI; state cravings via the FCQ-S; disinhibition, hunger and cognitive restraint via the Eating Inventory; and susceptibility to food environment via the PFS. Impulsiveness was assessed with the BIS, which includes an overall score and has six first-order factors (attention, cognitive stability, motor, perseverance, self-control and cognitive complexity) and three second-order factors (attentional, motor and non-planning). Attempts were made for questionnaires to be completed in a fasted state shortly after arrival to the study site except for the appetite VAS that was completed immediately before and after the test lunch. Further details on the ad libitum meal test and behavior questionnaires are provided in the supplementary information.

**Extended Data Fig. 9:**
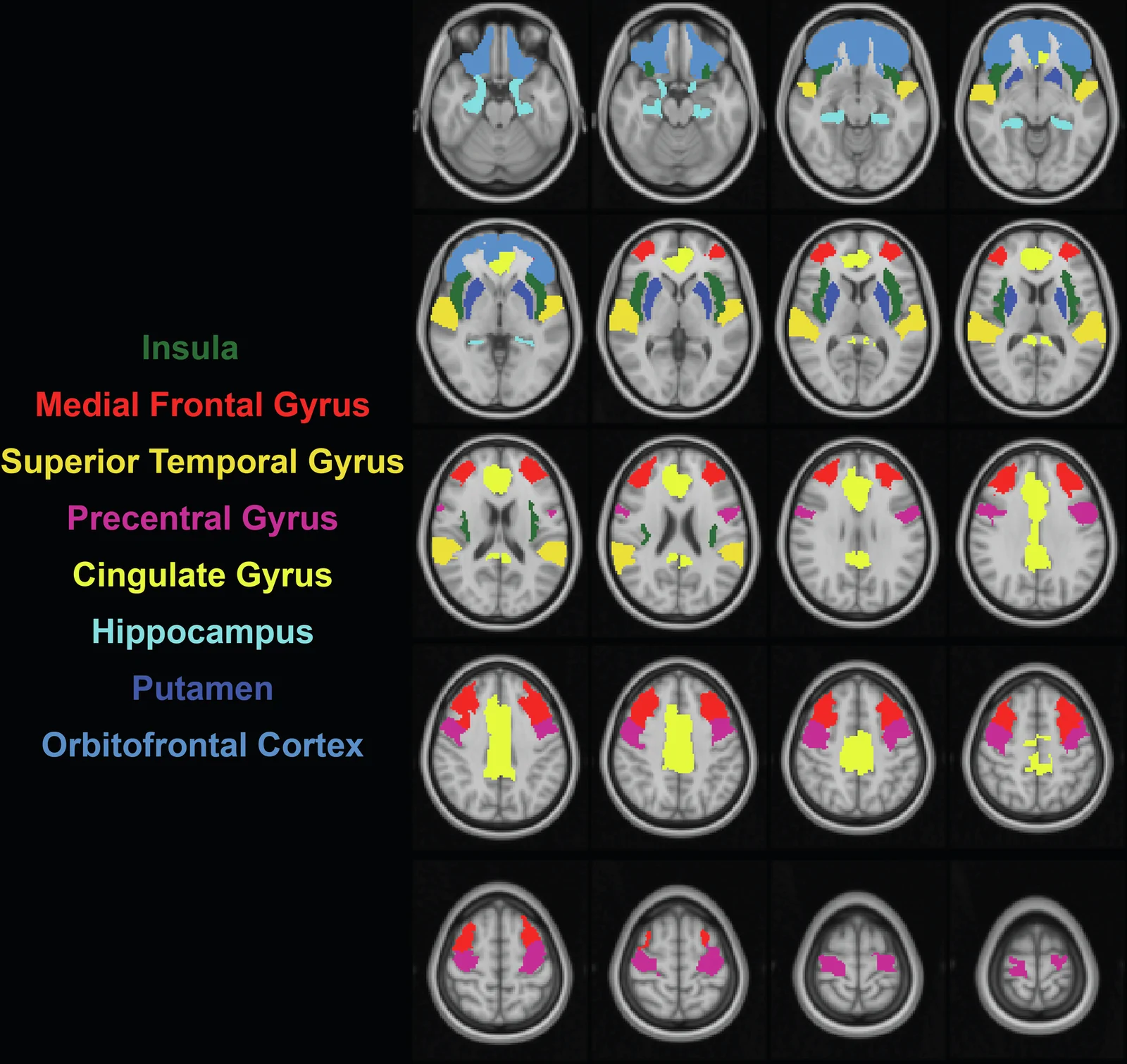
Brain regions of interest. Map of the prespecified brain regions of interest for BOLD fMRI. The dashed box shows the brain slice displayed in the results figures. No difference in signal was detected for the ventral striatum at baseline, week 3, or week 6; therefore, statistical analysis was not conducted for this brain region and this region is not shown. BOLD=blood oxygenation level dependent; fMRI=functional magnetic resonance imaging.

For BOLD fMRI, a Macronutrient Picture System paradigm validated for characterizing activation of brain regions implicated in appetite and food reward in response to food images was used^49^. Participants underwent BOLD fMRI while viewing photographs^55^ of non-food objects and foods from various categories^56^. The same set of photographs was viewed at each scanning visit. Food photographs were grouped into six food categories with 15 photographs in each^56^. High-fat and high-sugar (Food_HiF/HiS_—for example, cakes, cookies and candy bars) and high-fat and high-carbohydrate (Food_HiF/HiC_—for example, French fries, potato chips and cheese pizza) foods were aggregated into a highly palatable food category (Food_HiPal_). Findings presented here focus on responses to Food_HiPal_ and its components with change in brain activation from baseline to week 3 prespecified as a secondary objective. For each food category, BOLD activation was measured in nine a priori-defined brain ROIs based on the automated anatomical labeling atlas, as described in a prior study^49^. The insula, medial frontal gyrus, superior temporal gyrus, precentral gyrus and cingulate gyrus were assessed as the principal brain reward ROIs, and the hippocampus, putamen, orbitofrontal cortex and ventral striatum were assessed as exploratory ROIs (Extended Data Fig. 9). These ROIs were selected based on previous reports of changes in those regions with the use of GLP-1 RAs and/or previous evidence of linkage to intake regulation and food reward^8,13,36–38,40,42–45^. Mean BOLD activation (food > non-food) of voxels within each prespecified ROI served as the dependent variable. BOLD fMRI acquisition and methodology is described in detail below. No voxels were identified as having a positive contrast between food cue images and non-food objects in the ventral striatum; therefore, statistical analysis was not done on this region.

### fMRI

#### Food image task paradigm

Participants were required to complete the food cue image task during the BOLD fMRI scan. The food cue image task was developed using images from the food-pics database^55^. Ninety food images across sweet and savory tastes, high- and low-energy density and varied macronutrient composition were included along with 15 images of non-food objects (for example, everyday household objects). Macronutrient categories^56^ were made by categorizing each food as low-fat versus high-fat (<30% versus >30%); low-sugar versus high-sugar (<30% versus >30%); low-carbohydrate versus high-carbohydrate (<30% versus >30%) and low-protein versus high-protein (<30% versus >30%).

This resulted in six food categories with 15 photos in each: (1) high-fat and high-sugar (Food_HiF/HiS_); (2) high-fat and high-carbohydrate (Food_HiF/HiC_); (3) high-fat and low-carbohydrate and high-protein; (4) high-sugar and low-fat; (5) low-fat and high-carbohydrate; and (6) low-fat and low-carbohydrate and high-protein.

Color images viewed were 600 × 450 pixels in size and pictured one food on a white background. Among the six food categories, Food_HiF/HiS_ and Food_HiF/HiC_ were aggregated to make the highly palatable food category (Food_HiPal_). Low-fat and low-carbohydrate and high-protein foods were referred to as not highly palatable foods. The scan duration was 20.5 min and consisted of seven blocks with six interblock rest intervals. In each trial of the task, one food photo was displayed for 5 s followed by 0.5 s of a fixation crosshair. Next, the same image scaled to 80% of the original size was shown with the words ‘How much do you want to eat this?’ for 2.5 s. A slider bar with ‘Not at all’ and ‘Want very much’ on the left and right was used to rate preference for food. A fixation crosshair was then displayed for a minimum of 1.5 s before the next trial. There were 15 trials in total (that is, 15 photos) per block and 14 intertrial intervals per block.

#### Scanning parameters

Imaging was performed on four different 3T MRI scanners (Siemens Prisma Fit at Indiana University School of Medicine, Siemens Prisma at Purdue University, GE Discovery 750w at Pennington Biomedical Research Center and a Philips Ingenia Elition X at Johns Hopkins University). MRI scans were acquired using a uniform scanning protocol that minimized and accounted for between-site differences in MRI systems. Task-based BOLD fMRI acquisition parameters included repetition time of 3,000 ms, echo time of 30 ms, flip angle of 90°, slice thickness of 3.5 mm and 64 × 64 pixels image matrix. Physiologic (respiratory and cardiac) time courses were acquired simultaneously with imaging data using a lap belt and pulse oximeter. In addition to the fMRI sequence, a three-dimensional T1-weighted sequence (GE, three-dimensional Sagittal T1 IR-prepped fast SPGR; Philips, TFE; Siemens, MP-RAGE) was also acquired to delineate the predefined ROIs as well as to aid in preprocessing of the fMRI images.

#### fMRI processing

Preprocessing of fMRI data in Statistical Parametric Mapping 12 (SPM12) included slice-timing correction, head-motion correction, smoothing and warping to a standard coordinate frame. Data were then entered into a first-level voxel-wise analysis with each trial modeled as a boxcar function that covers the time when the large image is viewed. The boxcar function was convolved with the canonical hemodynamic response function with additional regressors for head motion, artifact detection and physiologic recordings. Functional scans were analyzed in the context of the general linear model. Highly palatable food, non-food objects and individual food categories were modeled separately. To assess brain activation related to viewing food pictures, the contrast between highly palatable foods and non-food objects was computed. Additionally, the contrast between individual food categories and non-food objects was computed. Average BOLD contrast (food > non-food) in each of the nine areas associated to the brain reward was reported (insula, medial frontal gyrus, superior temporal gyrus, precentral gyrus, cingulate gyrus, hippocampus, putamen, orbitofrontal cortex and ventral striatum). The nine ROIs were defined according to the automated anatomical labeling atlas as previously described^49,57^.

### Outcomes

The primary objective was to compare change from baseline to week 3 in energy intake during ad libitum test meals for tirzepatide and placebo. Secondary objectives were change from baseline to week 3 for tirzepatide versus placebo in fasting and postprandial appetite VAS ratings, FCI, FCQ-S, Eating Inventory, PFS questionnaire ratings and BOLD activation to photos of Food_HiPal_ (Food_HiF/HiS_ and Food_HiF/HiC_) relative to non-food during the fasting state in the prespecified principal reward ROIs. Exploratory objectives included changes in the above measures from baseline to week 6 for tirzepatide versus placebo and from baseline to week 3 and to week 6 for tirzepatide versus liraglutide and liraglutide versus placebo. Additional exploratory objectives included change from baseline in the BIS and in BOLD activation to Food_HiPal_ (Food_HiF/HiS_ and Food_HiF/HiC_) relative to non-food in the prespecified exploratory ROIs. Safety endpoints included AEs and safety laboratory parameters.

### Statistical methods

A total of 111 participants were planned to be randomized so that 93 participants (31 per treatment group) would complete the study. This sample size provides at least 80% power for the comparison of tirzepatide versus placebo for the change in energy intake during ad libitum test meals (primary outcome) based on a two-sample *t*-test at an alpha level of 0.05, given an expected treatment difference of 212 kcal and an assumed common standard deviation of 289 kcal for the change in energy intake from baseline. The primary outcome was analyzed using an analysis of covariance with treatment as fixed effect, and baseline BMI stratum and baseline energy intake as covariates.

No multiplicity adjustments were made in assessing secondary parameters. All secondary and exploratory parameters were assessed with reference to a two-sided 0.05 alpha level. Changes in efficacy parameters from baseline to week 3 and week 6 and changes from week 3 to week 6 were analyzed using a MMRMs with treatment, baseline BMI stratum, time point (of measurement) and treatment-by-time-point interaction as fixed effects, baseline value as a covariate and participant as a random effect. Scanner ID was also included in the model for BOLD fMRI parameters. Food cue task fMRI data were quality controlled and processed by an independent imaging core lab as per a previous paper^49^.

Analyses were conducted with data from randomized participants using all available evaluable data. Analyses were carried out using SAS Enterprise Guide v.8, unless stated otherwise. The number of patients who completed each assessment at each time point is shown in Supplementary Table 17.

### Reporting summary

Further information on research design is available in the Nature Portfolio Reporting Summary linked to this article.

## Online content

Any methods, additional references, Nature Portfolio reporting summaries, source data, extended data, supplementary information, acknowledgements, peer review information; details of author contributions and competing interests; and statements of data and code availability are available at https://doi.org/10.1038/s41591-025-03774-9.

## Supplementary information

Supplementary Information Supplementary inclusion and exclusion criteria, methods, Tables 1–17, references, trial protocol and trial statistical analysis plan.

Reporting Summary

## Source data

Source Data Figs. 2–4 All source data in single file with clearly named tabs for Figs. 2–4.

Source Data Extended Data Figs. 1–8 All source data in single file with clearly named tabs for Extended Data Figs. 1–8.

## Figures and Tables

**Extended Data Table 1:**
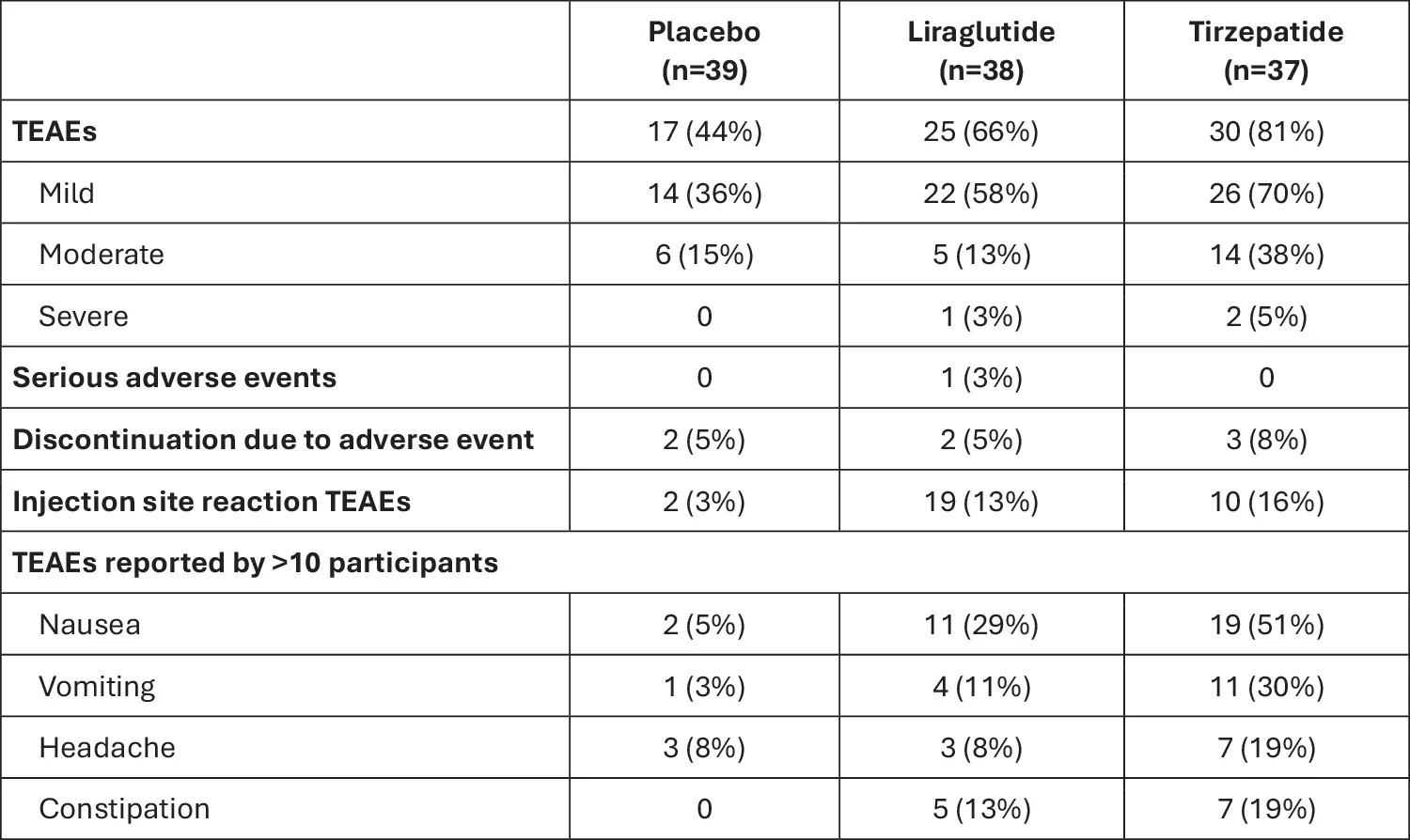
Summary of adverse events

Data are number of participants (%) with adverse events in all randomized participants who received ≥1 dose of study treatment. TEAE=treatment-emergent adverse event.

## Data Availability

Data from the analyses in this study cannot be shared publicly due to the sponsor’s (Eli Lilly and Company) contractual obligations. Eli Lilly and Company provides access to all individual participant data collected during the trial, after anonymization, except for pharmacokinetic or genetic data. Data are available to request 6 months after the indication studied has been approved in the United States and European Union and after primary publication acceptance, whichever is later. No expiration date of data requests is currently set once data have been made available. Access is provided after a proposal has been approved by an independent review committee identified for this purpose and after receipt of a signed data-sharing agreement. Data and documents, including the study protocol, statistical analysis plan, clinical study report and blank or annotated case report forms, will be provided in a secure data-sharing environment. For details on submitting a request, see the instructions provided at www.vivli.org. Contact the corresponding author for details on submitting a request. Source data are provided with this paper.

## References

[1] Bray, G. A., Kim, K. K., Wilding, J. P. H. & World Obesity Federation. Obesity: a chronic relapsing progressive disease process. A position statement of the World Obesity Federation. *Obes. Rev.* **18**, 715–723 (2017). 10.1111/obr.12551 28489290

[2] *Obesity and Overweight Key Facts. 2021* (World Health Organization, accessed 16 February 2024); www.who.int/news-room/fact-sheets/detail/obesity-and-overweight

[3] Carnell, S., Gibson, C., Benson, L., Ochner, C. N. & Geliebter, A. Neuroimaging and obesity: current knowledge and future directions. *Obes. Rev.* **13**, 43–56 (2012). 21902800 10.1111/j.1467-789X.2011.00927.x PMC3241905

[4] Chetty, A. K., Rafi, E., Bellini, N. J., Buchholz, N. & Isaacs, D. A review of incretin therapies approved and in late-stage development for overweight and obesity management. *Endocr. Pract.* **30**, 292–303 (2024). 38122931 10.1016/j.eprac.2023.12.010

[5] Bettadapura, S., Dowling, K., Jablon, K., Al-Humadi, A. W. & le Roux, C. W. Changes in food preferences and ingestive behaviors after glucagon-like peptide-1 analog treatment: techniques and opportunities. *Int. J. Obes.* **49**, 418–426 (2025). 10.1038/s41366-024-01500-y PMC11971042 38454010

[6] van Can, J. et al. Effects of the once-daily GLP-1 analog liraglutide on gastric emptying, glycemic parameters, appetite and energy metabolism in obese, non-diabetic adults. *Int. J. Obes.* **38**, 784–793 (2014). 10.1038/ijo.2013.162 PMC4052428 23999198

[7] Blundell, J. et al. Effects of once-weekly semaglutide on appetite, energy intake, control of eating, food preference and body weight in subjects with obesity. *Diabetes Obes. Metab.* **19**, 1242–1251 (2017). 28266779 10.1111/dom.12932 PMC5573908

[8] van Bloemendaal, L. et al. GLP-1 receptor activation modulates appetite- and reward-related brain areas in humans. *Diabetes* **63**, 4186–4196 (2014). 25071023 10.2337/db14-0849

[9] Eldor, R. et al. Discordance between central (brain) and pancreatic action of exenatide in lean and obese subjects. *Diabetes Care* **39**, 1804–1810 (2016). 27489336 10.2337/dc15-2706 PMC5864097

[10] Farr, O. M. et al. GLP-1 receptors exist in the parietal cortex, hypothalamus and medulla of human brains and the GLP-1 analogue liraglutide alters brain activity related to highly desirable food cues in individuals with diabetes: a crossover, randomised, placebo-controlled trial. *Diabetologia* **59**, 954–965 (2016). 26831302 10.1007/s00125-016-3874-y PMC4826792

[11] Ten Kulve, J. S. et al. Liraglutide reduces CNS activation in response to visual food cues only after short-term treatment in patients with type 2 diabetes. *Diabetes Care* **39**, 214–221 (2016). 26283736 10.2337/dc15-0772

[12] Ten Kulve, J. S. et al. Endogenous GLP1 and GLP1 analogue alter CNS responses to palatable food consumption. *J. Endocrinol.* **229**, 1–12 (2016). 26769912 10.1530/JOE-15-0461

[13] Farr, O. M. et al. Longer-term liraglutide administration at the highest dose approved for obesity increases reward-related orbitofrontal cortex activation in response to food cues: implications for plateauing weight loss in response to anti-obesity therapies. *Diabetes Obes. Metab.* **21**, 2459–2464 (2019). 31282006 10.1111/dom.13827 PMC6800581

[14] Coskun, T. et al. LY3298176, a novel dual GIP and GLP-1 receptor agonist for the treatment of type 2 diabetes mellitus: from discovery to clinical proof of concept. *Mol. Metab.* **18**, 3–14 (2018). 30473097 10.1016/j.molmet.2018.09.009 PMC6308032

[15] Jastreboff, A. M. et al. Tirzepatide once weekly for the treatment of obesity. *N. Engl. J. Med.* **387**, 205–216 (2022). 35658024 10.1056/NEJMoa2206038

[16] Martin, C. K. et al. 128-OR: the effect of tirzepatide during weight loss on food intake, appetite, food preference, and food craving in people with obesity. *Diabetes* **72**, 128-OR (2023).

[17] Ravussin, E. et al. 127-OR: the effect of tirzepatide during weight loss on metabolic adaption, fat oxidation, and food intake in people with obesity. *Diabetes* **72**, 127-OR (2023).

[18] Heise, T. et al. Tirzepatide reduces appetite, energy intake, and fat mass in people with type 2 diabetes. *Diabetes Care* **46**, 998–1004 (2023). 36857477 10.2337/dc22-1710 PMC10154650

[19] Chao, A. M. et al. Effects of liraglutide and behavioral weight loss on food cravings, eating behaviors, and eating disorder psychopathology. *Obesity* **27**, 2005–2010 (2019). 31746553 10.1002/oby.22653 PMC6873814

[20] Kadouh, H. et al. GLP-1 analog modulates appetite, taste preference, gut hormones, and regional body fat stores in adults with obesity. *J. Clin. Endocrinol. Metab.* **105**, 1552–1563 (2020). 31665455 10.1210/clinem/dgz140 PMC7105351

[21] Williamson, D. A. et al. Microanalysis of eating behavior of three leptin deficient adults treated with leptin therapy. *Appetite* **45**, 75–80 (2005). 15949871 10.1016/j.appet.2005.01.002

[22] Adriaenssens, A. E. et al. Glucose-dependent insulinotropic polypeptide receptor-expressing cells in the hypothalamus regulate food intake. *Cell Metab.* **30**, 987–996.e6 (2019). 31447324 10.1016/j.cmet.2019.07.013 PMC6838660

[23] Dorling, J. L. et al. Change in self-efficacy, eating behaviors and food cravings during two years of calorie restriction in humans without obesity. *Appetite* **143**, 104397 (2019). 31398376 10.1016/j.appet.2019.104397 PMC6766406

[24] Alabduljabbar, K., Bonanos, E., Miras, A. D. & le Roux, C. W. Mechanisms of action of bariatric surgery on body weight regulation. *Gastroenterol. Clin. North Am.* **52**, 691–705 (2023). 37919021 10.1016/j.gtc.2023.08.002

[25] Bryant, E. J., Malik, M. S., Whitford-Bartle, T. & Waters, G. M. The effects of bariatric surgery on psychological aspects of eating behaviour and food intake in humans. *Appetite* **150**, 104575 (2020). 31875518 10.1016/j.appet.2019.104575

[26] Kober, H. & Boswell, R. G. Potential psychological & neural mechanisms in binge eating disorder: implications for treatment. *Clin. Psychol. Rev.* **60**, 32–44 (2018). 29329692 10.1016/j.cpr.2017.12.004

[27] Schulte, E. M., Grilo, C. M. & Gearhardt, A. N. Shared and unique mechanisms underlying binge eating disorder and addictive disorders. *Clin. Psychol. Rev.* **44**, 125–139 (2016). 26879210 10.1016/j.cpr.2016.02.001 PMC5796407

[28] Gearhardt, A. N. & Schulte, E. M. Is food addictive? A review of the science. *Annu. Rev. Nutr.* **41**, 387–410 (2021). 34152831 10.1146/annurev-nutr-110420-111710

[29] Chuong, V. et al. The glucagon-like peptide-1 (GLP-1) analogue semaglutide reduces alcohol drinking and modulates central GABA neurotransmission. *JCI Insight* **8**, e170671 (2023). 37192005 10.1172/jci.insight.170671 PMC10371247

[30] Klausen, M. K., Thomsen, M., Wortwein, G. & Fink-Jensen, A. The role of glucagon-like peptide 1 (GLP-1) in addictive disorders. *Br. J. Pharmacol.* **179**, 625–641 (2022). 34532853 10.1111/bph.15677 PMC8820218

[31] Wang, W. et al. Association of semaglutide with tobacco use disorder in patients with type 2 diabetes: target trial emulation using real-world data. *Ann. Intern. Med.* **177**, 1016–1027 (2024). 39074369 10.7326/M23-2718 PMC12721465

[32] Wang, W. et al. Association of semaglutide with reduced incidence and relapse of cannabis use disorder in real-world populations: a retrospective cohort study. *Mol. Psychiatry* **29**, 2587–2598 (2024). 38486046 10.1038/s41380-024-02498-5 PMC11412894

[33] Roth, C. L. et al. Impaired brain satiety responses after weight loss in children with obesity. *J. Clin. Endocrinol. Metab.* **107**, 2254–2266 (2022). 35544121 10.1210/clinem/dgac299 PMC9282278

[34] Murdaugh, D. L., Cox, J. E., Cook, E. W. III & Weller, R. E. fMRI reactivity to high-calorie food pictures predicts short- and long-term outcome in a weight-loss program. *Neuroimage* **59**, 2709–2721 (2012). 22332246 10.1016/j.neuroimage.2011.10.071 PMC3287079

[35] Boswell, R. G. & Kober, H. Food cue reactivity and craving predict eating and weight gain: a meta-analytic review. *Obes. Rev.* **17**, 159–177 (2016). 26644270 10.1111/obr.12354 PMC6042864

[36] Thorpe, S. J., Rolls, E. T. & Maddison, S. The orbitofrontal cortex: neuronal activity in the behaving monkey. *Exp. Brain Res.* **49**, 93–115 (1983). 6861938 10.1007/BF00235545

[37] Rolls, E. T., Critchley, H. D., Mason, R. & Wakeman, E. A. Orbitofrontal cortex neurons: role in olfactory and visual association learning. *J. Neurophysiol.* **75**, 1970–1981 (1996). 8734596 10.1152/jn.1996.75.5.1970

[38] Rolls, E. T., Feng, R., Cheng, W. & Feng, J. Orbitofrontal cortex connectivity is associated with food reward and body weight in humans. *Soc. Cogn. Affect. Neurosci.* **18**, nsab083 (2023). 34189586 10.1093/scan/nsab083 PMC10498940

[39] Eiler, W. J. et al. Ventral frontal satiation-mediated responses to food aromas in obese and normal-weight women. *Am. J. Clin. Nutr.* **99**, 1309–1318 (2014). 24695888 10.3945/ajcn.113.080788 PMC4021781

[40] Saruco, E. & Pleger, B. A systematic review of obesity and binge eating associated impairment of the cognitive inhibition system. *Front. Nutr.* **8**, 609012 (2021). 33996871 10.3389/fnut.2021.609012 PMC8116510

[41] Spetter, M. S., de Graaf, C., Viergever, M. A. & Smeets, P. A. Anterior cingulate taste activation predicts ad libitum intake of sweet and savory drinks in healthy, normal-weight men. *J. Nutr.* **142**, 795–802 (2012). 22378331 10.3945/jn.111.153445

[42] Johnson, S. C. et al. Neural correlates of self-reflection. *Brain* **125**, 1808–1814 (2002). 12135971 10.1093/brain/awf181

[43] Johnson, M. K., Nolen-Hoeksema, S., Mitchell, K. J. & Levin, Y. Medial cortex activity, self-reflection and depression. *Soc. Cogn. Affect. Neurosci.* **4**, 313–327 (2009). 19620180 10.1093/scan/nsp022 PMC2799950

[44] Passingham, R. E., Bengtsson, S. L. & Lau, H. C. Medial frontal cortex: from self-generated action to reflection on one’s own performance. *Trends Cogn. Sci.* **14**, 16–21 (2010). 19969501 10.1016/j.tics.2009.11.001 PMC2806969

[45] Moran, J. M., Kelley, W. M. & Heatherton, T. F. What can the organization of the brain’s default mode network tell us about self-knowledge? *Front. Hum. Neurosci.* **7**, 391 (2013). 23882210 10.3389/fnhum.2013.00391 PMC3713343

[46] Kerem, L. et al. Modulation of neural fMRI responses to visual food cues by overeating and fasting interventions: a preliminary study. *Physiol. Rep.* **8**, e14639 (2021). 33369272 10.14814/phy2.14639 PMC7758977

[47] Kahathuduwa, C. N. et al. Effects of 3-week total meal replacement vs. typical food-based diet on human brain functional magnetic resonance imaging food-cue reactivity and functional connectivity in people with obesity. *Appetite* **120**, 431–441 (2018). 28958900 10.1016/j.appet.2017.09.025

[48] Ochner, C. N. et al. Selective reduction in neural responses to high calorie foods following gastric bypass surgery. *Ann. Surg.* **253**, 502–507 (2011). 21169809 10.1097/SLA.0b013e318203a289 PMC3128512

[49] King, J. L. et al. Perceptual characterization of the Macronutrient Picture System (MaPS) for food image fMRI. *Front. Psychol.* **9**, 17 (2018). 29434559 10.3389/fpsyg.2018.00017 PMC5790788

[50] Thanarajah, S. E. et al. Habitual daily intake of a sweet and fatty snack modulates reward processing in humans. *Cell Metab.* **35**, 571–584.e6 (2023). 36958330 10.1016/j.cmet.2023.02.015

[51] Johnson, P. M. & Kenny, P. J. Dopamine D2 receptors in addiction-like reward dysfunction and compulsive eating in obese rats. *Nat. Neurosci.* **13**, 635–641 (2010). 20348917 10.1038/nn.2519 PMC2947358

[52] *Saxenda US, Package Insert* (Novo Nordisk, 2018); www.accessdata.fda.gov/drugsatfda_docs/label/2018/206321s007lbl.pdf

[53] *Wegovy US, Package Insert* (Novo Nordisk, 2021); www.accessdata.fda.gov/drugsatfda_docs/label/2021/215256s000lbl.pdf

[54] Johnson-Mann, C. N. et al. A systematic review on participant diversity in clinical trials—have we made progress for the management of obesity and its metabolic sequelae in diet, drug, and surgical trials. *J. Racial Ethn. Health Disparities* **10**, 3140–3149 (2023). 36536164 10.1007/s40615-022-01487-0 PMC10645628

[55] Blechert, J., Meule, A., Busch, N. A. & Ohla, K. Food-pics: an image database for experimental research on eating and appetite. *Front. Psychol.* **5**, 617 (2014). 25009514 10.3389/fpsyg.2014.00617 PMC4067906

[56] Geiselman, P. J. et al. Reliability and validity of a macronutrient self-selection paradigm and a food preference questionnaire. *Physiol. Behav.* **63**, 919–928 (1998). 9618017 10.1016/s0031-9384(97)00542-8

[57] Tzourio-Mazoyer, N. et al. Automated anatomical labeling of activations in SPM using a macroscopic anatomical parcellation of the MNI MRI single-subject brain. *Neuroimage* **15**, 273–289 (2002). 11771995 10.1006/nimg.2001.0978

