# Supplementary material for: Tirzepatide on ingestive behavior in adults with overweight or obesity: a randomized 6-week phase 1 trial
Source: Nat Med. 2025 Jun 24;31(9):3141–50. doi: 10.1038/s41591-025-03774-9 (PMC12443625; doi:10.1038/s41591-025-03774-9)
Supplement: Supplementary file 1 — Supplementary inclusion and exclusion criteria, methods, Tables 1–17, references, trial protocol and trial statistical analysis plan. [file 41591_2025_3774_MOESM1_ESM.pdf]

nature medicine

Supplementary information https://doi.org/10.1038/s41591-025-03774-9

Tirzepatide on ingestive behavior in adults with overweight or obesity: a randomized 6-week phase 1 trial

In the format provided by the authors and unedited

Supplementary Material

Table of Contents

Inclusion and Exclusion Criteria.......2
Supplementary Methods.......8
Table S1. Pre-lunch fasting VAS subscales.......12
Table S2. Postprandial VAS.......14
Table S3. Fasting retrospective VAS.......17
Table S4. Food Craving Inventory subscales.......19
Table S5. Food Craving Questionnaire-State subscales.......20
Table S6. Power of Food subscales.......22
Table S7. Barratt Impulsiveness Scale subscales.......23
Table S8. BOLD fMRI brain activation for highly palatable foods versus non-food objects.......26
Table S9. BOLD fMRI brain activation for high-fat/high-sugar foods versus non-food objects.......28
Table S10. BOLD fMRI brain activation for high-fat/high-carbohydrate foods versus non-food objects.......30
Table S11. BOLD fMRI brain activation for high-fat/low-carbohydrate foods versus non-food objects.......32
Table S12. BOLD fMRI brain activation for high-sugar/low-fat foods versus non-food objects.......34
Table S13. BOLD fMRI brain activation for low-fat/high-carbohydrate foods versus non-food objects.......36
Table S14. BOLD fMRI brain activation for low-fat/low-carbohydrate/high-protein foods (not highly palatable foods) versus non-food objects.......38
Table S15. Sensitivity analysis of energy intake in participants who experienced and did not experience any nausea or vomiting adverse events during the study.......40
Table S16. Post-hoc analysis of macronutrient intake during the ad libitum lunch meal.......41
Table S17. Number of participants who completed each assessment at each time point.......42
References.......43
Trial Protocol and Statistical Analysis Plan.......44

Inclusion and Exclusion Criteria

Inclusion Criteria

Participants were eligible for inclusion in the study only if they meet all the following criteria at screening:

<u>Subject Characteristics</u>

[1] have overweight/obesity (BMI of 27 kg/m^2 to 50 kg/m^2, inclusive, and can complete MRI scans (see Exclusion Criterion 8), and nondiabetic male or female subjects, as determined by medical history, physical examination, and laboratory assessments at screening;

[2] must be weight stable (no weight change of >4 kg) in the last 1 month;

[3] have safety laboratory test results within normal reference range or with abnormalities deemed clinically insignificant by the investigator;

[4] have venous access sufficient to allow for blood sampling as per the protocol;

[5] male or female subjects between the ages of 18 to 65 years, inclusive;

[5a] male subjects:

- men, regardless of their fertility status, with nonpregnant woman of childbearing potential (WOCBP) partners must agree to either remain abstinent (if this is their preferred and usual lifestyle) or use condoms plus 1 additional highly effective (less than 1% failure rate) method of contraception (such as combination oral contraceptives, implanted contraceptives, or intrauterine device) or effective method of contraception, (such as diaphragms with spermicide or cervical sponge) for the duration of the study and for 5 half-lives of study drug plus 90 days, corresponding to 4 months after the last injection.
    - men and their partners may choose to use a double-barrier method of contraception. (Barrier protection methods without concomitant use of a spermicide are not an effective or acceptable method of contraception. Thus, each barrier method must include use of a spermicide. It should be noted however that the use of male and female condoms as a double-barrier method is not considered acceptable due to the high failure rate when these barrier methods are combined.)
    - periodic abstinence (e.g., calendar, ovulation, symptothermal, postovulation methods), declaration of abstinence just for the duration of a study, and withdrawal are not acceptable methods of contraception.
- men with pregnant partners should use condoms during intercourse for the duration of the study and until the end of estimated relevant potential exposure in WOCBP, which corresponds to 4 months following last injection.
- men must agree to refrain from sperm donation for the duration of the study and until their plasma concentrations are below the level that could result in a relevant potential exposure to a possible fetus, predicted to be 4 months following last injection.

- men who are in exclusively same-sex relationships (as their preferred and usual lifestyle) are not required to use contraception.

[5b] female subjects:

- women of childbearing potential who are abstinent (if this is complete abstinence, as their preferred and usual lifestyle) or in a same-sex relationship (as part of their preferred and usual lifestyle) must agree to either remain abstinent or stay in a same-sex relationship without sexual relationships with males. Periodic abstinence (e.g., calendar, ovulation, symptothermal, post-ovulation methods), declaration of abstinence just for the duration of a study, and withdrawal are not acceptable methods of contraception.
- otherwise, WOCBP participating must agree to use effective contraception, where at least 1 form is highly effective (less than 1% failure rate), for the entirety of the study. Contraception must continue following completion of IP administration for 30 days.
    - women of childbearing potential participating must test negative for pregnancy prior to initiation of treatment as indicated by a negative serum pregnancy test at the screening visit followed by a negative urine pregnancy test within 24 hours prior to exposure
    - two forms of effective contraception, where at least 1 form is highly effective (less than 1% failure rate, such as combination oral contraceptives, implanted contraceptives or intrauterine devices) will be used. Effective contraception (such as male or female condoms with spermicide, diaphragms with spermicide or cervical sponges) may be used as the second therapy. Barrier protection methods without concomitant use of a spermicide are not a reliable or acceptable method. Thus, each barrier method must include use of a spermicide (that is, condom with spermicide, diaphragm with spermicide, female condom with spermicide). It should be noted that the use of male and female condoms as a double barrier method is not considered acceptable due to the high failure rate when these methods are combined
    - Not be breastfeeding
- women who are not of childbearing potential may participate and include those who are:
    - infertile due to surgical sterilization (hysterectomy, bilateral oophorectomy, or tubal ligation), congenital anomaly such as mullerian agenesis, or
    - postmenopausal – defined as either
        i. a woman at least 40 years of age with an intact uterus, not on hormone therapy, who has cessation of menses for at least 1 year without an alternative medical cause, AND a follicle-stimulating hormone (FSH) ≥40 mIU/mL; women in

this category must test negative in pregnancy test prior to study entry

ii. a woman 55 years or older not on hormone therapy, who has had at least 12 months of spontaneous amenorrhea; or

iii. a woman 55 years or older with a diagnosis of menopause prior to starting hormone replacement therapy.

Informed Consent

[6] are reliable and willing to make themselves available for the duration of the study and are willing to follow study procedures;

[7] have given written informed consent approved by Lilly and the institutional review board governing the site.

Exclusion Criteria

Participants will be excluded from study enrollment if they meet any of the following criteria at screening:

Medical Conditions - General

[8] have a maximum body circumference >219 cm (i.e., at the widest point of the body) or a body weight >225 kg, in order to not exceed the body size limitations imposed by the MRI scanner machinery;

[9] have a history or current cardiovascular (e.g., myocardial infarction, congestive heart failure, cerebrovascular accident, venous thromboembolism), respiratory, hepatic, renal, GI, endocrine, hematological (including history of thrombocytopenia), or neurological disorders capable of significantly altering the absorption, metabolism, or elimination of drugs; or constituting a risk when taking the IP; or may interfere with the interpretation of data;

[10] have obesity induced by other endocrinologic disorders (e.g., Cushing syndrome) or diagnosed monogenetic or syndromic forms of obesity (e.g., melanocortin 4 receptor deficiency or Prader-Willi syndrome)

[11] have acute or chronic pancreatitis or a history of acute idiopathic pancreatitis; or have other GI disorders (e.g., relevant esophageal reflux or gall bladder disease) that could be aggravated by GLP-1 analogs;

- Subjects who have had cholecystolithiasis (removal of gall stones) and/or cholecystectomy (removal of gall bladder) in the past, with no long-term complications, are eligible for participation;

[12] have a known clinically significant gastric emptying abnormality (e.g., severe diabetic gastroparesis or gastric outlet obstruction) or chronically take drugs that directly affect GI motility.

[13] have or plan to have endoscopic and/or device-based therapy for obesity or have had device removal within the last 6 months (for example, mucosal ablation, gastric artery embolization, intragastric balloon and duodenal-jejunal bypass sleeve)

[14] have a prior or planned surgical treatment for obesity (excluding liposuction or abdominoplasty if performed >1 year prior to screening)

[15] have an estimated glomerular filtration rate (eGFR) <60 mL/min/1.73 m^2, calculated by Chronic Kidney Disease-Epidemiology at screening.

[16] have an intention to enter into a weight loss program during the study;

[17] have a personal or family history of medullary thyroid carcinoma, have multiple endocrine neoplasia syndrome type 2 (MEN 2), or calcitonin ≥20 pg/mL at screening;

[18] are diagnosed with any form of diabetes prior to entry or have a hemoglobin A1c (HbA1c) value at screening of ≥6.5%;

[19] have findings in the 12-lead ECG at screening that, in the opinion of the investigator, may increase the risks of potentially clinically relevant worsening associated with participation in the study;

[20] have blood pressure of ≥160/90 mmHg and/or pulse rate of <45 or >100 bpm (supine) at screening with or without stable doses [at least 1 month prior to screening] of anti-hypertension medications;

[21] have an active or untreated malignancy or have been in remission from a clinically significant malignancy (other than basal or squamous cell skin cancer, in situ carcinomas of the cervix, or in situ prostate cancer) for <5 years prior to screening;

[22] have evidence of human immunodeficiency virus (HIV) and/or positive HIV antibodies at screening;

[23] have evidence of hepatitis B or positive hepatitis B surface antigen and/or evidence of hepatitis C virus (HCV) or hepatitis C antibody at screening;

- subjects with a previous diagnosis of HCV who have been treated with antiviral therapy and achieved a sustained virological response may be eligible for inclusion in the study, provided they have no detectable HCV RNA on the screening HCV polymerase chain reaction test. A sustained virological response is defined as an undetectable HCV RNA level 24 weeks after completion of a full, documented course of an approved antiviral therapy for HCV.
- subjects who have spontaneously cleared HCV infection, defined as (1): a positive HCV antibody test and (2): a negative HCV RNA test, with no history of anti-HCV treatment, may be eligible for inclusion in the study, provided they have no detectable HCV RNA on screening for this study.

[24] have serum aspartate aminotransferase (AST) or alanine aminotransferase (ALT) >2.0× the upper limit of normal (ULN), total bilirubin (TBL) >1.5× ULN (except for subjects with Gilbert's syndrome, which can be enrolled with TBL <2.0× ULN), or alkaline phosphatase (ALP) >1.5× ULN;

- subjects with non-alcoholic fatty liver disease are eligible for participation;

[25] have had a blood donation of 450 mL or more in the last 3 months or any blood donation within the last month prior to screening;

[26] have a history of drug or alcohol abuse within the last 12 months prior to screening; or a positive drug or alcohol screen; and/or smoke >10 cigarettes per day or the equivalent;

[27] have a history of marijuana use within 3 months of enrollment and unwillingness to abstain from marijuana use during the trial. Subjects should also refrain use of cannabidiol oil for the duration of the study.

[28] have had a blood transfusion or severe blood loss within the last 3 months, or have a hemoglobin value <11 g/dL (males) or <10 g/dL (females),

[29] have an average weekly alcohol intake that exceeds 21 units per week (males) and 14 units per week (females) (1 unit = 12 oz or 360 mL of beer; 5 oz or 150 mL of wine; 1.5 oz or 45 mL of distilled spirits), and/or are unwilling to stop alcohol consumption 12 hours before each CRU outpatient visit;

[30] have any lifetime history of a suicide attempt;

[31] Have a Patient Health Questionnaire-9 (PHQ-9) score of 15 or more at screening;

[32] On the Columbia-Suicide Severity Rating Scale (C-SSRS) at screening:

- a “yes” answer to either Question 4 (Active Suicidal Ideation with Some Intent to Act, Without Specific Plan) or
- a “yes” answer to Question 5 (Active Suicidal Ideation with Specific Plan and Intent) on the “Suicidal Ideation” portion of the C-SSRS, or
- a “yes” answer to any of the suicide-related behaviors (Actual Attempt, Interrupted Attempt, Aborted Attempt, Preparatory Act or Behavior) on the "Suicidal Behavior" portion of the C-SSRS, and
- the ideation or behavior occurred within the past month

[33] have a history of atopy or clinically significant multiple or severe drug allergies, or severe post-treatment hypersensitivity reactions (including, but not limited to, erythema multiforme major, linear immunoglobulin A dermatosis, toxic epidermal necrolysis, or exfoliative dermatitis).

<u>Prior/Concomitant Therapy - General</u>

[34] have current or history of (within 6 months prior to screening or between screening and randomization) treatment with medications that may cause significant weight gain, including but not limited to: tricyclic antidepressants, atypical antipsychotic mood stabilizers for example:

Tofranil® (imipramine), Elavil® (amitriptyline), Remeron® (mirtazapine), Paxil®/Seroxat® (paroxetine), Nardil® (phenelzine), Largactil® (chlorpromazine), Mellaril® (thioridazine), Clozaril®/FazaClo®/Versacloz® (clozapine), Zyprexa® (olanzapine), Depakote®/Convulex® (valproic acid) and its derivatives, and/or Lithobid® (lithium)

Note: selective serotonin reuptake inhibitors other than paroxetine are permitted.

Use of other medications not listed above, may result in subject exclusion at the discretion of the investigator in consultation with the Sponsor.

[35] have taken within 6 months prior to screening or between screening and randomization, medications (prescribed or over-the-counter) or alternative remedies intended to promote weight loss. Examples include, but are not limited to:

Saxenda® (liraglutide 3.0 mg), Xenical®/Alli® (orlistat), Meridia® (sibutramine), Acutrium® (phenylpropanolamine), Sanorex® (mazindol), Adipex® (phentermine), BELVIQ® (lorcaserin), Qsymia® (phentermine/topiramate combination), and/or Contrave® (naltrexone/bupropion).

Note: Use of metformin or any other glucose-lowering medication, whether prescribed for polycystic ovary syndrome or diabetes prevention is not permitted.

Use of other medications not listed above, may result in subject exclusion at the discretion of the investigator in consultation with the Sponsor.

[36] have received chronic (lasting >14 consecutive days) systemic glucocorticoid therapy (excluding topical, intra-articular, and inhaled preparations) within 1 month before screening, or between screening and randomization;

[37] have been treated with any glucose-lowering agent during the last 3 months prior to screening or between screening and randomization;

[38] have received treatment with a drug that has not received regulatory approval for any indication within 3 months or 5 half-lives (whichever is longer) of screening.

Prior/Concurrent Clinical Trial Experience

[39] are persons who have previously completed or withdrawn from this study;

[40] have known allergies to tirzepatide or GLP-1 RAs;

[41] have previous exposure to tirzepatide or exposure to GLP-1 RA in the previous 6 months prior to screening and between screening and randomization;

[42] are currently enrolled in a clinical study involving an IP or any other type of medical research judged not to be scientifically or medically compatible with this study.

Other Exclusions

[43] female subjects who are breastfeeding;

[44] suffer from claustrophobia sufficiently severe that it may preclude completion of MRI scanning;

[45] have contraindications such as cardiac pacemakers, aneurysm clips, or other metallic implants considered unsafe for MRI scanning;

[46] are investigative site personnel directly affiliated with this study and their immediate families. Immediate family is defined as a spouse, parent, child, or sibling, whether biological or legally adopted;

[47] are Eli Lilly and Company employees;

[48] are deemed unsuitable by the investigator for any other reason.

Supplementary Methods

Ad libitum meal test

Participants completed a test lunch at baseline, week 3, and week 6, between approximately 11:30 AM and 12:30 PM. Lunch was a buffet meal consisting of:

- baked potato chips
- grilled chicken bites
- barbeque sauce
- baked tortilla chips
- popcorn chicken bites
- sweet and sour sauce
- corn chips
- cheddar cheese
- M&M'S®
- pretzel twists
- swiss cheese
- crème filled chocolate cake rolls
- buttered popcorn
- salsa
- raisins
- mixed nuts with salt
- fat free ranch dressing
- fruit punch
- baby carrots
- cheese dip
- water

Participants were instructed that they could eat as much or as little as they wished during the test meals and that they should alert a staff member when they finished eating, at which time the participant left the test meal. Staff members were instructed to check on the participants after 25 minutes and terminate the session after 40 minutes if the participant did not end the session.

Food intake was quantified by precisely weighing foods before and after the test meals, as close to the beginning and end of the meal as possible. Energy and nutrient intake was calculated by linking each food to a match in the United States Department of Agriculture Food and Nutrient Database for Dietary Studies Studies[1] or data from the food producer/manufacturer.

Ingestive behavior questionnaires

Ingestive behavior questionnaires were administered at baseline, week 3, and week 6. Attempts were made for questionnaires to be completed in a fasted state shortly after arrival to the study site except for the appetite VAS which was completed immediately before and after the test lunch.

Appetite visual analog scale

Participants completed VAS ratings immediately before and after the test lunch. Participants were asked to rate their feelings of hunger, satiety, fullness, prospective food consumption, and desire for specific foods a 10-cm line using a pen-and-paper VAS. Subjects recorded their rating on the respective lines; ratings ranged from 0 (“not at all”) to 100 (“extremely”) for hunger, satiety, fullness, and prospective food consumption assessment, and from 0 (“Yes, very much”) to 100 (“No, not at all”) for assessment of desiring to eat something fatty, salty, sweet or savory. The ratings included the following 8 questions:[2]

- How hungry do you feel right now? (hunger)
- How satisfied do you feel right now? (satiety)
- How full do you feel right now? (fullness)
- How much food do you think you could eat right now? (prospective food consumption)
- Would you like to eat something sweet?
- Would you like to eat something salty?
- Would you like to eat something savory?
- Would you like to eat something fatty?

Overall appetite score was calculated as the average of the four individual scores for questions 1-4 (satiety + fullness + [100-prospective food consumption] + [100-hunger]/4). A higher overall appetite score indicates less appetite, and a lower score indicates more appetite.

Retrospective appetite visual analog scale

Participants provided retrospective VAS ratings to assess average ratings of appetite that they experienced over the past week. The retrospective method of collecting VAS data has been found to be consistent with daily assessments of satiety.[3] Participants recorded their rating on 10-cm lines; rating ranged from 0 (“not at all”) to 100 (“extremely”). The retrospective VAS assessed 4 appetite-related metrics:

- How hungry did you feel over the past week?
- How satisfied did you feel over the past week?
- How full did you feel over the past week?
- How much food did you think you could eat over the past week?

Overall appetite score was calculated as the average of the four individual scores (satiety + fullness + [100-prospective food consumption] + [100-hunger]/4). A higher overall appetite score indicates less appetite, and a lower score indicates more appetite.

In addition to the appetite metrics noted above, retrospective VAS ratings were also used to quantify feelings of nausea, malaise, and gastrointestinal distress over the previous week. These items were anchored with verbal descriptors ranging from “none at all” to “an extreme amount”. The ratings included the following 3 questions:

- How much nausea did you have over the past week?
- How much malaise did you have over the past week?
- How much gastrointestinal distress did you have over the past week?

Food Craving Inventory

The 33-item FCI was used to measure cravings for specific food groups.[4] The measure consisted of five empirically derived factors:

- high fats
- sweets
- carbohydrates/starches
- fast-food fats
- and fruits and vegetables

The FCI is scaled in a frequency format and assesses the frequency of cravings for particular foods over the past month. All items are scored in the following manner: Never = 1, Rarely = 2, Sometimes = 3, Often = 4, Always = 5. The instructions for the FCI will be altered for week 3 and week 6 to include the following time frame: "Since the last time you completed this questionnaire, how often have you experienced a craving for the food?". This differs from the original instructions, which denotes a 1-month time frame [4] All other instructions on the FCI were the same as the original.

Food Craving Questionnaire-State

The FCQ-S is a 15-item measure that assesses the strength of state food cravings at administration. The FCQ-S has demonstrated good validity and internal consistency in non-clinical populations at the time of provision.[5,6] The FCQ-S assesses the following scores:

- desire
- anticipation positive
- anticipation negative
- lack of control over eating
- hunger

Anticipation positive refers to anticipation of positive reinforcement that may arise from eating, anticipation negative refers to anticipation of relief from negative states and feelings as a result of eating. Greater scores for each subscale denote higher levels of craving.

Eating Inventory

The Eating Inventory is a 51-item validated questionnaire that assesses three eating-related constructs:[7]

- cognitive restraint

- disinhibition
- perceived hunger

Cognitive restraint refers to the intent and ability to restrict energy intake, disinhibition measures the tendency to overeat, and hunger measures susceptibility to feelings of hunger. Scores for restraint, disinhibition, and hunger range from 0 to 21, 0 to 18, and 0 to 14, respectively, and a greater number indicates greater levels of each respective eating component.

Power of Food Scale

The PFS measures appetite for palatable foods in the environment at the following 3 levels of food proximity:[8]

- food readily available in the environment but not physically present (food available)
- food present but not tasted (food present)
- food when first tasted but not consumed (food tasted)

The Power of Food Scale has adequate internal consistency and test-retest reliability.[9]

Barrat Impulsiveness Scale

The Barratt Impulsiveness Scale is one of the most widely used self-report questionnaires for measuring impulsivity.[10,11] The scale is a 30-item self-reported measure describing impulsive or nonimpulsive behaviors and preferences. The scale consists of 6 first-order factors:

- attention; for example, being restless during events when you must be stationary
- motor impulsiveness; for example, doing things without much forethought
- self-control; for example, speaking out of turn
- cognitive complexity; for example, being bored when not intellectually stimulated
- perseverance; for example, changing tasks or jobs frequently
- cognitive instability; for example, experiencing rapid or racing patterns of thought.

The scale also consists of the 3 second-order factors:

- attentional impulsiveness, that is having difficulties with attention and rapid thoughts
- motor impulsiveness, that is having trouble with motor impulsiveness and perseverance
- non-planning impulsiveness, that is having trouble with self-control and cognitive complexity.

The items were rated on a 4-point scale with responses ranging from 1 = rarely or never to 4 = almost always or always. The maximum score of 4 indicates the most impulsive response.

Table S1. Pre-lunch fasting VAS subscales

	Placebo (n=39)	Liraglutide (n=38)	Tirzepatide (n=37)
**Hunger score**			
Baseline	76.76	78.15	78.25
Change at week 3	-3.51 (4.16)	-15.76 (4.28)	-28.73 (4.53)
Vs placebo	..	-12.25 (-23.53 to -0.98), p=0.0336	-25.22 (-36.91 to -13.54), p<0.0001
Vs liraglutide	..	..	-12.97 (-24.65 to -1.30), p=0.0299
Change at week 6	3.11 (4.32)	-15.45 (4.30)	-36.99 (4.57)
Vs placebo	..	-18.56 (-30.09 to -7.04), p=0.0019	-40.10 (-52.04 to -28.16), p<0.0001
Vs liraglutide	..	..	-21.53 (-33.32 to -9.75), p=0.0005
Change from week 3 to week 6 within treatment group	6.62 (-2.20 to 15.43), p=0.1395	0.30 (-8.30 to 8.90), p=0.9442	-8.26 (-17.41 to 0.89), p=0.0762
**Satiety score**			
Baseline	35.36	32.42	37.69
Change at week 3	-5.31 (3.33)	-1.26 (3.43)	9.26 (3.64)
Vs placebo	..	4.05 (-4.96 to 13.07), p=0.3740	14.57 (5.23 to 23.91), p=0.0026
Vs liraglutide	..	..	10.52 (1.18 to 19.86), p=0.0277
Change at week 6	-4.80 (4.34)	0.09 (4.27)	14.26 (4.55)
Vs placebo	..	4.90 (-6.79 to 16.58), p=0.4071	19.07 (6.97 to 31.17), p=0.0024
Vs liraglutide	..	..	14.17 (2.24 to 26.10), p=0.0205
Change from week 3 to week 6 within treatment group	0.50 (-8.63 to 9.64), p=0.9129	1.35 (-7.55 to 10.24), p=0.7639	5.00 (-4.49 to 14.49), p=0.2976
**Prospective food consumption score**			
Baseline	65.85	66.06	66.69
Change at week 3	-4.47 (3.43)	-10.21 (3.54)	-25.45 (3.76)
Vs placebo	..	-5.74 (-14.97 to 3.49), p=0.2196	-20.98 (-30.59 to -11.37), p<0.0001
Vs liraglutide	..	..	-15.24 (-24.82 to -5.66), p=0.0022
Change at week 6	2.73 (3.81)	-12.71 (3.82)	-32.08 (4.07)
Vs placebo	..	-15.44 (-25.62 to -5.26), p=0.0034	-34.81 (-45.39 to -24.22), p<0.0001
Vs liraglutide	..	..	-19.37 (-29.83 to -8.90), p=0.0004
Change from week 3 to week 6 within treatment group	7.20 (0.97 to 13.43), p=0.0241	-2.50 (-8.55 to 3.54), p=0.4121	-6.63 (-13.04 to -0.22), p=0.0428
**Fullness**			
Baseline	12.48	14.00	8.47
Change at week 3	5.91 (4.05)	10.92 (4.17)	28.05 (4.41)
Vs placebo	..	5.01 (-6.05 to 16.07), p=0.3699	22.14 (10.66 to 33.62), p=0.0002
Vs liraglutide	..	..	17.13 (5.60 to 28.65), p=0.0040
Change at week 6	1.37 (4.41)	12.49 (4.36)	30.86 (4.62)
Vs placebo	..	11.13 (-0.70 to 22.95), p=0.0648	29.49 (17.23 to 41.76), p<0.0001
Vs liraglutide	..	..	18.37 (6.24 to 30.49), p=0.0034
Change from week 3 to week 6 within treatment group	-4.54 (-15.24 to 6.16), p=0.4016	1.57 (-8.91 to 12.06), p=0.7664	2.81 (-8.39 to 14.02), p=0.6187
**Desire for sweet foods**			
Baseline	44.48	38.61	51.59
Change at week 3	-3.56 (4.83)	7.30 (4.99)	23.90 (5.31)
Vs placebo	..	10.86 (-2.18 to 23.91), p=0.1015	27.46 (13.92 to 41.00), p<0.0001
Vs liraglutide	..	..	16.59 (2.96 to 30.23), p=0.0177
Change at week 6	-0.79 (5.29)	8.59 (5.30)	25.60 (5.65)
Vs placebo	..	9.38 (-4.78 to 23.53), p=0.1913	26.39 (11.69 to 41.09), p=0.0006
Vs liraglutide	..	..	17.01 (2.38 to 31.64), p=0.0232
Change from week 3 to week 6 within treatment group	2.77 (-6.83 to 12.37), p=0.5673	1.29 (-8.04 to 10.62), p=0.7844	1.70 (-8.20 to 11.61), p=0.7332
**Desire for salty foods**			
Baseline	37.36	38.30	42.97
Change at week 3	0.99 (4.74)	-1.99 (4.87)	15.30 (5.15)
Vs placebo	..	-2.98 (-15.73 to 9.78), p=0.6439	14.31 (0.96 to 27.66), p=0.0359
Vs liraglutide	..	..	17.29 (3.97 to 30.61), p=0.0116
Change at week 6	-2.15 (5.12)	2.51 (5.08)	15.38 (5.39)

Vs placebo	..	4.66 (-8.95 to 18.26), p=0.4980	17.52 (3.30 to 31.75), p=0.0164
Vs liraglutide	..	..	12.87 (-1.15 to 26.89), p=0.0716
Change from week 3 to week 6 within treatment group	-3.13 (-13.47 to 7.20), p=0.5481	4.50 (-5.57 to 14.57), p=0.3772	0.07 (-10.64 to 10.79), p=0.9891
**Desire for savoury foods**			
Baseline	37.36	31.18	35.84
Change at week 3	0.33 (4.91)	-2.28 (5.09)	13.42 (5.35)
Vs placebo	..	-2.61 (-15.96 to 10.74), p=0.6986	13.09 (-0.65 to 26.83), p=0.0616
Vs liraglutide	..	..	15.70 (1.85 to 29.55), p=0.0268
Change at week 6	5.50 (5.54)	4.32 (5.54)	20.23 (5.85)
Vs placebo	..	-1.19 (-16.07 to 13.70), p=0.8746	14.73 (-0.63 to 30.10), p=0.0600
Vs liraglutide	..	..	15.92 (0.64 to 31.19), p=0.0413
Change from week 3 to week 6 within treatment group	5.18 (-5.92 to 16.27), p=0.3563	6.60 (-4.20 to 17.40), p=0.2277	6.81 (-4.68 to 18.31), p=0.2415
**Desire for fatty foods**			
Baseline	49.18	45.94	42.03
Change at week 3	2.89 (4.54)	7.25 (4.66)	17.13 (4.94)
Vs placebo	..	4.36 (-7.97 to 16.70), p=0.4840	14.24 (1.47 to 27.02), p=0.0293
Vs liraglutide	..	..	9.88 (-2.88 to 22.65), p=0.1275
Change at week 6	-4.49 (4.71)	4.60 (4.67)	24.83 (4.96)
Vs placebo	..	9.08 (-3.47 to 21.64), p=0.1539	29.32 (16.33 to 42.32), p<0.0001
Vs liraglutide	..	..	20.24 (7.42 to 33.05), p=0.0023
Change from week 3 to week 6 within treatment group	-7.38 (-17.52 to 2.77), p=0.1519	-2.65 (-12.56 to 7.25), p=0.5958	7.70 (-2.84 to 18.25), p=0.1501

Data are mean of absolute values at baseline, least squares mean (standard error) change from baseline, and difference in least squares mean (95% CI) vs placebo and liraglutide. Higher desire to eat scores indicates less desire. CI=confidence interval; n=number of participants who were randomised and received at least one dose of study treatment; VAS=visual analogue scale.

Table S2. Postprandial VAS

	Placebo (n=39)	Liraglutide (n=38)	Tirzepatide (n=37)
**Overall score**			
Baseline	86.20	87.39	88.21
Change at week 3	-0.59 (2.08)	0.05 (2.12)	1.66 (2.23)
Vs placebo	..	0.64 (-5.02 to 6.31) p=0.8227	2.25 (-3.60 to 8.09), p=0.4469
Vs liraglutide	..	..	1.61 (-4.23 to 7.45), p=0.5861
Change at week 6	-0.15 (1.92)	-0.19 (1.92)	0.29 (2.01)
Vs placebo	..	-0.04 (-5.17 to 5.08), p=0.9869	0.43 (-4.85 to 5.72), p=0.8709
Vs liraglutide	..	..	0.48 (-4.75 to 5.70), p=8568
Change from week 3 to week 6 within treatment group	0.44 (-3.54 to 4.43), p=0.8261	-0.24 (-4.15 to 3.67), p=0.9021	-1.38 (-5.49 to 2.74), p=0.5082
**Hunger score**			
Baseline	6.10	6.32	6.05
Change at week 3	0.78 (2.43)	3.35 (2.46)	-0.91 (2.59)
Vs placebo	..	2.57 (-4.11 to 9.24), p=0.4469	-1.69 (-8.57 to 5.19), p=0.6265
Vs liraglutide	..	..	-4.26 (-11.14 to 2.61), p=0.2217
Change at week 6	-0.56 (1.91)	1.62 (1.89)	0.30 (1.98)
Vs placebo	..	2.17 (-2.90 to 7.25), p=0.3969	0.86 (-4.36 to 6.08), p=0.7440
Vs liraglutide	..	..	-1.31 (-6.46 to 3.84), p=0.6139
Change from week 3 to week 6 within treatment group	-1.33 (-6.67 to 4.00), p=0.6204	-1.73 (-7.00 to 3.54), p=0.5158	1.22 (-4.35 to 6.79), p=0.6646
**Satiety score**			
Baseline	82.72	82.84	85.16
Change at week 3	-3.77 (3.00)	2.60 (3.06)	-0.46 (3.21)
Vs placebo	..	6.37 (-1.78 to 14.53), p=0.1240	3.32 (-5.08 to 11.71), p=0.4351
Vs liraglutide	..	..	-3.06 (-11.46 to 5.34), p=0.4714
Change at week 6	-2.56 (3.01)	2.50 (2.97)	-3.14 (3.13)
Vs placebo	..	5.06 (-2.97 to 13.09), p=0.2142	-0.59 (-8.83 to 7.66), p=0.8883
Vs liraglutide	..	..	-5.64 (-13.80 to 2.52), p=0.1730
Change from week 3 to week 6 within treatment group	1.21 (-5.17 to 7.60), p=0.7070	-0.10 (-6.36 to 6.15), p=0.9738	-2.69 (-9.28 to 3.91), p=0.4206
**Prospective food consumption score**			
Baseline	11.92	10.74	9.11
Change at week 3	-0.33 (2.05)	-1.01 (2.09)	-5.48 (2.19)
Vs placebo	..	-0.68 (-6.27 to 4.91), p=0.8090	-5.15 (-10.91 to 0.62), p=0.0796
Vs liraglutide	..	..	-4.46 (-10.22 to 1.29), p=0.1268
Change at week 6	-2.22 (2.10)	-0.37 (2.07)	-5.10 (2.18)
Vs placebo	..	1.85 (-3.77 to 7.47), p=0.5145	-2.88 (-8.68 to 2.91), p=0.3257
Vs liraglutide	..	..	-4.73 (-10.45 to 0.98), p=0.1031
Change from week 3 to week 6 within treatment group	-1.89 (-6.73 to 2.95), p=0.4395	0.64 (-4.11 to 5.40), p=0.7880	0.38 (-4.64 to 5.39), p=0.8821
**Fullness**			
Baseline	80.10	83.79	82.84
Change at week 3	1.66 (3.19)	0.53 (3.26)	1.09 (3.41)

Vs placebo	..	-1.12 (-9.86 to 7.62), p=0.7991	-0.57 (-9.55 to 8.42), p=0.9008
Vs liraglutide	..	..	0.56 (-8.44 to 9.55), p=0.9023
Change at week 6	0.24 (2.95)	-1.16 (2.93)	-0.13 (3.07)
Vs placebo	..	-1.40 (-9.29 to 6.49), p=0.7256	-0.37 (-8.48 to 7.74), p=0.9282
Vs liraglutide	..	..	1.03 (-6.99 to 9.05), p=0.7992
Change from week 3 to week 6 within treatment group	-1.42 (-8.43 to 5.60), p=0.6897	-1.69 (-8.59 to 5.21), p=0.6275	-1.22 (-8.50 to 6.07), p=0.7403
**Desire for sweet foods**			
Baseline	82.41	78.58	82.22
Change at week 3	1.61 (4.33)	4.32 (4.43)	3.18 (4.65)
Vs placebo	..	2.71 (-9.10 to 14.51), p=0.6498	1.58 (-10.61 to 13.76), p=0.7979
Vs liraglutide	..	..	-1.13 (-13.31 to 11.04), p=0.8538
Change at week 6	7.39 (3.68)	5.98 (3.72)	10.65 (3.89)
Vs placebo	..	-1.41 (-11.19 to 8.38), p=0.7757	3.27 (-6.81 to 13.35), p=0.5215
Vs liraglutide	..	..	4.68 (-5.32 to 14.67), p=0.3554
Change from week 3 to week 6 within treatment group	5.78 (-0.88 to 12.43), p=0.0883	1.66 (-4.87 to 8.19), p=0.6151	7.47 (0.60 to 14.33), p=0.0333
**Desire for salty foods**			
Baseline	85.56	85.29	88.62
Change at week 3	0.79 (3.48)	2.78 (3.54)	-3.09 (3.75)
Vs placebo	..	1.99 (-7.47 to 11.46), p=0.6770	-3.88 (-13.67 to 5.92), p=0.4340
Vs liraglutide	..	..	-5.87 (-15.65 to 3.90), p=0.2362
Change at week 6	1.14 (3.08)	4.60 (3.08)	3.35 (3.25)
Vs placebo	..	3.46 (-4.71 to 11.63), p=0.4028	2.21 (-6.24 to 10.66), p=0.6052
Vs liraglutide	..	..	-1.25 (-9.61 to 7.10), p=0.7668
Change from week 3 to week 6 within treatment group	0.35 (-5.94 to 6.65), p=0.9120	1.82 (-4.36 to 8.00), p=0.5603	6.44 (-0.07 to 12.94), p=0.0524
**Desire for savoury foods**			
Baseline	85.13	81.87	88.62
Change at week 3	1.37 (3.95)	-2.42 (4.02)	-2.99 (4.28)
Vs placebo	..	-3.79 (-14.53 to 6.94), p=0.4846	-4.36 (-15.47 to 6.75), p=0.4379
Vs liraglutide	..	..	-0.56 (-11.71 to 10.58), p=0.9200
Change at week 6	3.95 (3.64)	2.79 (3.63)	0.51 (3.86)
Vs placebo	..	-1.16 (-10.83 to 8.52), p=0.8129	-3.43 (-13.45 to 6.58), p=0.4981
Vs liraglutide	..	..	-2.28 (-12.22 to 7.67), p=0.6507
Change from week 3 to week 6 within treatment group	2.57 (-4.85 to 9.99), p=0.4930	5.21 (-2.06 to 12.49), p=0.1584	3.50 (-4.16 to 11.16), p=0.3668
**Desire for fatty foods**			
Baseline	85.87	88.29	88.24
Change at week 3	2.92 (3.64)	0.94 (3.72)	-1.18 (3.90)
Vs placebo	..	-1.98 (-11.84 to 7.89), p=0.6918	-4.10 (-14.28 to 6.09), p=0.4264
Vs liraglutide	..	..	-2.12 (-12.28 to 8.04), p=0.6794
Change at week 6	1.15 (3.84)	3.79 (3.82)	0.10 (4.02)
Vs placebo	..	2.64 (-7.65 to 12.93), p=0.6121	-1.05 (-11.66 to 9.56), p=0.8447
Vs liraglutide	..	..	-3.69 (-14.16 to 6.78), p=0.4861

Change from week 3 to week 6 within treatment group	-1.77 (-9.21 to 5.68), p=0.6387	2.85 (-4.43 to 10.13), p=0.4387	1.28 (-6.38 to 8.95), p=0.7406

Data are mean of absolute values at baseline, least squares mean (standard error) change from baseline, and difference in least squares mean (95% CI) vs placebo and liraglutide. A higher overall appetite score indicates less appetite. Higher desire to eat scores indicates less desire. CI=confidence interval; n=number of participants who were randomised and received at least one dose of study treatment; VAS=visual analogue scale.

Table S3. Fasting retrospective VAS

	Placebo (n=39)	Liraglutide (n=38)	Tirzepatide (n=37)
**Overall appetite score**			
Baseline	51.06	52.88	52.31
Change at week 3	2.37 (2.52)	14.06 (2.56)	23.19 (2.69)
Vs placebo	..	11.69 (4.86 to 18.52), p=0.0010	20.83 (13.79 to 27.86), p<0.0001
Vs liraglutide	..	..	9.14 (2.12 to 16.15), p=0.0112
Change at week 6	3.33 (2.52)	12.49 (2.51)	20.56 (2.64)
Vs placebo	..	9.16 (2.42 to 15.90), p=0.0083	17.23 (10.29 to 24.18), p<0.0001
Vs liraglutide	..	..	8.07 (1.21 to 14.94), p=0.0217
Change from week 3 to week 6 within treatment group	0.96 (-4.06 to 5.98), p=0.7042	-1.57 (-6.48 to 3.34), p=0.5269	-2.63 (-7.80 to 2.54), p=0.3144
**Average hunger score**			
Baseline	57.51	54.42	58.14
Change at week 3	-4.91 (3.21)	25.02 (3.28)	-40.22 (3.45)
Vs placebo	..	-20.11 (-28.81 to -11.40), p<0.0001	- 35.31 (-44.25 to -26.38), p<0.0001
Vs liraglutide	..	..	-15.21 (-24.21 to -6.20), p=0.0011
Change at week 6	-8.81 (3.43)	-26.94 (3.44)	-37.85 (3.63)
Vs placebo	..	-18.14 (-27.37 to -8.90), p=0.0002	-29.04 (-38.53 to -19.55), p<0.0001
Vs liraglutide	..	..	-10.90 (-20.40 to -1.40), p=0.0249
Change from week 3 to week 6 within treatment group	-3.90 (-10.75 to 2.95), p=0.2615	-1.93 (-8.66 to 4.80), p=0.5709	2.38 (-4.72 to 9.47), p=0.5080
**Nausea**			
Baseline	11.05	5.29	5.89
Change at week 3	2.40 (4.55)	18.85 (4.63)	23.59 (4.85)
Vs placebo	..	16.46 (4.15 to 28.77), p=0.0093	21.20 (8.56 to 33.83), p=0.0012
Vs liraglutide	..	..	4.74 (-7.84 to 17.32), p=0.4566
Change at week 6	2.05 (4.73)	17.69 (4.74)	35.50 (4.97)
Vs placebo	..	15.63 (2.93 to 28.33), p=0.0164	33.44 (20.39 to 46.50), p<0.0001
Vs liraglutide	..	..	17.81 (4.89 to 30.73), p=0.0074
Change from week 3 to week 6 within treatment group	-0.34 (-8.41 to 7.73), p=0.9334	-1.17 (-9.04 to 6.70), p=0.7693	11.91 (3.63 to 20.18), p=0.0053
**Average prospective food consumption score**			
Baseline	57.64	59.37	62.57
Change at week 3	-6.86 (3.00)	-23.34 (3.07)	-29.70 (3.22)
Vs placebo	..	-16.48 (-24.60 to -8.36), p=0.0001	-22.85 (-31.22 to -14.47), p<0.0001
Vs liraglutide	..	..	-6.37 (-14.73 to 2.00), p=0.1341
Change at week 6	-3.45 (3.69)	-15.58 (3.68)	-28.17 (3.88)
Vs placebo	..	-12.13 (-22.11 to -2.15), p=0.0178	-24.72 (-35.02 to -14.43), p<0.0001
Vs liraglutide	..	..	-12.60 (-22.83 to -2.36), p=0.0164
Change from week 3 to week 6 within treatment group	3.41 (-4.13 to 10.94), p=0.3717	7.76 (0.35 to 15.17), p=0.0403	1.53 (-6.29 to 9.35), p=0.6984
**Average fullness**			

Baseline	59.49	63.68	63.95
Change at week 3	-0.93 (3.53)	7.14 (3.57)	15.95 (3.76)
Vs placebo	..	8.07 (-1.50 to 17.64), p=0.0976	16.88 (7.01 to 26.75), p=0.0010
Vs liraglutide	..	..	8.81 (-1.01 to 18.64), p=0.0782
Change at week 6	0.76 (3.41)	9.34 (3.39)	15.89 (3.56)
Vs placebo	..	8.58 (-0.53 to 17.69), p=0.0644	15.13 (5.74 to 24.52), p=0.0019
Vs liraglutide	..	..	6.55 (-2.71 to 15.81), p=0.1636
Change from week 3 to week 6 within treatment group	1.69 (-5.40 to 8.77), p=0.6376	2.20 (-4.74 to 9.14), p=0.5302	-0.06 (-7.37 to 7.25), p=0.9865
**Satiety**			
Baseline	59.90	61.61	66.00
Change at week 3	-1.24 (4.02)	2.73 (4.09)	6.42 (4.31)
Vs placebo	..	3.97 (-6.89 to 14.83), p=0.4701	7.66 (-3.61 to 18.92), p=0.1808
Vs liraglutide	..	..	3.69 (-7.54 to 14.91), p=0.5162
Change at week 6	0.40 (4.31)	-0.37 (4.32)	-0.15 (4.56)
Vs placebo	..	-0.77 (-12.37 to 10.82), p=0.8952	-0.54 (-12.54 to 11.45), p=0.9284
Vs liraglutide	..	..	0.23 (-11.70 to 12.15), p=0.9699
Change from week 3 to week 6 within treatment group	1.64 (-7.24 to 10.52), p=0.7154	-3.10 (-11.83 to 5.62), p=0.4820	-6.56 (-15.76 to 2.64), p=0.1602
**Malaise**			
Baseline	15.15	18.11	25.78
Change at week 3	1.95 (4.37)	6.30 (4.45)	5.59 (4.71)
Vs placebo	..	4.35 (-7.43 to 16.13), p=0.4660	3.64 (-8.70 to 15.98), p=0.5596
Vs liraglutide	..	..	-0.71 (-12.98 to 11.57), p=0.9095
Change at week 6	0.10 (4.42)	6.66 (4.38)	15.53 (4.64)
Vs placebo	..	6.56 (-5.16 to 18.29), p=0.2694	15.43 (3.13 to 27.74), p=0.0145
Vs liraglutide	..	..	8.87 (-3.20 to 20.94), p=0.1479
Change from week 3 to week 6 within treatment group	-1.85 (-10.04 to 6.33), p=0.6541	0.36 (-7.64 to 8.36), p=0.9286	9.94 (1.52 to 18.36), p=0.0212
**Gastrointestinal distress**			
Baseline	13.08	12.47	16.46
Change at week 3	-1.39 (3.79)	6.11 (3.87)	10.01 (4.06)
Vs placebo	..	7.50 (-2.84 to 17.83), p=0.1531	11.40 (0.74 to 22.07), p=0.0364
Vs liraglutide	..	..	3.91 (-6.77 to 14.59), p=0.4696
Change at week 6	-2.26 (3.82)	9.81 (3.79)	13.67 (3.97)
Vs placebo	..	12.06 (1.83 to 22.29), p=0.0213	15.92 (5.37 to 26.48), p=0.0035
Vs liraglutide	..	..	3.86 (-6.58 to 14.30), p=0.4645
Change from week 3 to week 6 within treatment group	-0.86 (-9.69 to 7.97), p=0.8467	3.70 (-4.97 to 12.37), p=0.3988	3.66 (-5.50 to 12.82), p=0.4300

Data are mean of absolute values at baseline, least squares mean (standard error) change from baseline, and difference in least squares mean (95% CI) vs placebo and liraglutide. A higher overall appetite score indicates less appetite. CI=confidence interval; n=number of participants who were randomised and received at least one dose of study treatment; VAS=visual analogue scale.

Table S4. Food Craving Inventory subscales

	Placebo (n=39)	Liraglutide (n=38)	Tirzepatide (n=37)
**High fats**			
Baseline	2.21	2.48	2.11
Change at week 3	-0.24 (0.09)	-0.38 (0.09)	-0.61 (0.10)
Vs placebo	..	-0.14 (-0.38 to 0.11), p=0.2679	-0.38 (-0.63 to -0.12, p=0.0038
Vs liraglutide	..	..	-0.24 (-0.50 to 0.02), p=0.0690
Change at week 6	-0.22 (0.09)	-0.51 (0.09)	-0.76 (0.10)
Vs placebo	..	-0.29 (-0.54 to -0.04), p=0.0233	-0.54 (-0.79 to -0.28), p<0.0001
Vs liraglutide	..	..	-0.24 (-0.51 to 0.02), p=0.0655
Change from week 3 to week 6 within treatment group	0.01 (-0.14 to 0.17), p=0.8501	-0.14 (-0.29 to 0.01), p=0.0708	-0.14 (-0.30 to 0.02), p=0.0766
**Sweets**			
Baseline	2.62	2.74	2.57
Change at week 3	-0.43 (0.11)	-0.59 (0.11)	-1.00 (0.12)
Vs placebo	..	-0.16 (-0.45 to 0.13), p=0.2718	-0.57 (-0.87 to -0.27), p=0.0003
Vs liraglutide	..	..	-0.41 (-0.71 to -0.11), p=0.0080
Change at week 6	-0.39 (0.10)	-0.69 (0.10)	-1.24 (0.11)
Vs placebo	..	-0.30 (-0.57 to -0.03), p=0.0304	-0.85 (-1.13 to -0.57), p<0.0001
Vs liraglutide	..	..	-0.55 (-0.83 to -0.27), p=0.0002
Change from week 3 to week 6 within treatment group	0.03 (-0.14 to 0.21), p=0.7042	-0.10 (-0.28 to 0.07), p=0.2339	-0.24 (-0.42 to -0.06), p=0.0110
**Carbohydrates/starches**			
Baseline	2.44	2.64	2.22
Change at week 3	-0.16 (0.10)	-0.34 (0.11)	-0.61 (0.12)
Vs placebo	..	-0.17 (-0.46 to 0.11); p=0.2299	-0.45 (-0.74 to -0.15), p=0.0032
Vs liraglutide	..	..	-0.28 (-0.58 to 0.02), p=0.0705
Change at week 6	-0.22 (0.10)	-0.56 (0.10)	-0.84 (0.11)
Vs placebo	..	-0.34 (-0.61 to -0.06), p=0.0166	-0.62 (-0.90 to -0.34), p<0.0001
Vs liraglutide	..	..	-0.28 (-0.57 to 0.00), p=0.0517
Change from week 3 to week 6 within treatment group	-0.06 (-0.23 to 0.12), p=0.5123	-0.22 (-0.39 to -0.05), p=0.0115	-0.23 (-0.41 to -0.05), p=0.0139
**Fast food fats**			
Baseline	3.10	3.20	3.02
Change at week 3	-0.38 (0.12)	-0.66 (0.13)	-1.04 (0.14)
Vs placebo	..	-0.28 (-0.61 to 0.05), p=0.1000	-0.65 (-1.00 to -0.31), p=0.0003
Vs liraglutide	..	..	-0.37 (-0.72 to -0.03), p=0.0351
Change at week 6	-0.36 (0.13)	-0.91 (0.13)	-1.26 (0.14)
Vs placebo	..	-0.55 (-0.89 to -0.21), p=0.0018	-0.89 (-1.24 to -0.54), p<0.0001
Vs liraglutide	..	..	-0.34 (-0.69 to 0.01), p=0.0552
Change from week 3 to week 6 within treatment group	0.02 (-0.19 to 0.23), p=0.8481	-0.25 (-0.45 to -0.05), p=0.0162	-0.22 (-0.43 to 0.00), p=0.0465
**Fruits and vegetables**			
Baseline	2.25	2.41	2.09
Change at week 3	-0.04 (0.10)	-0.03 (0.10)	-0.11 (0.10)
Vs placebo	..	0.01 (-0.25 to 0.27), p=0.9371	-0.07 (-0.33 to 0.20), p=0.6250
Vs liraglutide	..	..	-0.08 (-0.35 to 0.19), p=0.5760
Change at week 6	-0.02 (0.10)	-0.22 (0.10)	-0.23 (0.11)
Vs placebo	..	-0.20 (-0.47 to 0.06), p=0.1274	-0.21 (-0.49 to 0.06), p=0.1225
Vs liraglutide	..	..	-0.01 (-0.28 to 0.26), p=0.9488
Change from week 3 to week 6 within treatment group	0.02 (-0.15 to 0.20), p=0.8010	-0.19 (-0.36 to -0.02), p=0.0277	-0.13 (-0.31 to 0.06), p=0.1757

Data are mean of absolute values at baseline, least squares mean (standard error) change from baseline, and difference in least squares mean (95% CI) vs placebo and liraglutide. CI=confidence interval; n=number of participants who were randomised and received at least one dose of study treatment.

Table S5. Food Craving Questionnaire-State subscales

	Placebo (n=39)	Liraglutide (n=38)	Tirzepatide (n=37)
**Physiological state (hunger)**			
Baseline	3.34	3.47	3.25
Change at week 3	-0.01 (0.14)	-0.33 (0.14)	-0.89 (0.15)
Vs placebo	..	-0.31 (-0.69 to 0.06), p=0.1032	-0.87 (-1.26 to -0.48), p<0.0001
Vs liraglutide	..	..	-0.56 (-0.95 to -0.17), p=0.0058
Change at week 6	0.06 (0.14)	-0.50 (0.15)	-1.04 (0.15)
Vs placebo	..	-0.56 (-0.95 to -0.18), p=0.0048	-1.10 (-1.50 to -0.71), p<0.0001
Vs liraglutide	..	..	-0.54 (-0.94 to -0.15), p=0.0080
Change from week 3 to week 6 within treatment group	0.08 (-0.18 to 0.33), p=0.5588	-0.17 (-0.42 to 0.08), p=0.1741	-0.16 (-0.42 to 0.11), p=0.2463
**Lack of control**			
Baseline	2.30	2.31	2.46
Change at week 3	-0.09 (0.13)	-0.43 (0.13)	-0.88 (0.14)
Vs placebo	..	-0.34 (-0.69 to 0.00), p=0.0518	-0.78 (-1.14 to -0.42), p<0.0001
Vs liraglutide	..	..	-0.44 (-0.80 to -0.08), p=0.0166
Change at week 6	-0.09 (0.13)	-0.59 (0.13)	-1.15 (0.14)
Vs placebo	..	-0.50 (-0.85 to -0.16), p=0.0047	-1.06 (-1.41 to -0.70), p<0.0001
Vs liraglutide	..	..	-0.55 (-0.91 to -0.20), p=0.0027
Change from week 3 to week 6 within treatment group	0.00 (-0.21 to 0.21), p=0.9750	-0.16 (-0.36 to 0.05), p=0.1297	-0.27 (-0.49 to -0.05), p=0.0155
**Anticipation of negative reinforcement**			
Baseline	2.97	3.13	2.96
Change at week 3	-0.12 (0.14)	-0.36 (0.15)	-0.87 (0.15)
Vs placebo	..	-0.23 (-0.62 to 0.15), p=0.2358	-0.75 (-1.15 to -0.35), p=0.0003
Vs liraglutide	..	..	-0.51 (-0.91 to -0.11), p=0.0123
Change at week 6	-0.13 (0.15)	-0.67 (0.16)	-0.97 (0.16)
Vs placebo	..	-0.54 (-0.95 to -0.12), p=0.0120	-0.84 (-1.27 to -0.42), p=0.0002
Vs liraglutide	..	..	-0.31 (-0.73 to 0.12), p=0.1553
Change from week 3 to week 6 within treatment group	-0.01 (-0.30 to 0.28), p=0.9650	-0.31 (-0.59 to -0.03), p=0.0325	-0.10 (-0.41 to 0.20), p=0.4956
**Anticipation of positive reinforcement**			
Baseline	2.96	3.07	2.91
Change at week 3	-0.10 (0.14)	-0.47 (0.15)	-1.04 (0.16)
Vs placebo	..	-0.37 (-0.75 to 0.02), p=0.0632	-0.95 (-1.35 to -0.54), p<0.0001
Vs liraglutide	..	..	-0.58 (-0.98 to -0.17), p=0.0054
Change at week 6	-0.17 (0.14)	-0.81 (0.14)	-1.32 (0.15)
Vs placebo	..	-0.64 (-1.02 to -0.25), p=0.0013	-1.15 (-1.55 to -0.76), p<0.0001
Vs liraglutide	..	..	-0.52 (-0.91 to -0.12), p=0.0107
Change from week 3 to week 6 within treatment group	-0.07 (-0.31 to 0.17), p=0.5526	-0.34 (-0.58 to -0.11), p=0.0049	-0.28 (-0.53 to -0.03), p=0.0281
**Desire**			
Baseline	2.97	3.10	2.94
Change at week 3	-0.04 (0.15)	-0.44 (0.15)	-1.17 (0.16)
Vs placebo	..	-0.40 (-0.81 to 0.00), p=0.0522	-1.13 (-1.55 to -0.71), p<0.0001
Vs liraglutide	..	..	-0.73 (-1.15 to -0.31), p=0.0008
Change at week 6	-0.16 (0.15)	-0.74 (0.15)	-1.52 (0.16)
Vs placebo	..	-0.57 (-0.97 to -0.17), p=0.0054	-1.36 (-1.77 to -0.95), p<0.0001
Vs liraglutide	..	..	-0.79 (-1.20 to -0.38), p=0.0002
Change from week 3 to week 6 within treatment group	-0.13 (-0.40 to 0.15), p=0.3574	-0.30 (-0.57 to -0.03), p=0.0310	-0.35 (-0.64 to -0.07), p=0.0162

Data are mean of absolute values at baseline, least squares mean (standard error) change from baseline, and difference in least squares mean (95% CI) vs placebo and liraglutide. CI=confidence interval; n=number of participants who were randomised and received at least one dose of study treatment.

Table S6. Power of Food subscales

	Placebo (n=39)	Liraglutide (n=38)	Tirzepatide (n=37)
**Food available**			
Baseline	2.76	2.57	2.91
Change at week 3	0.00 (0.11)	-0.43 (0.12)	-0.97 (0.12)
Vs placebo	..	-0.43 (-0.74 to -0.13), p=0.0058	-0.97 (-1.29 to -0.66), p<0.0001
Vs liraglutide	..	..	-0.54 (-0.86 to -0.22), p=0.0011
Change at week 6	-0.07 (0.13)	-0.70 (0.13)	-1.11 (0.14)
Vs placebo	..	-0.63 (-0.99 to -0.28), p=0.0006	-1.04 (-1.40 to -0.67), p<0.0001
Vs liraglutide	..	..	-0.40 (-0.77 to -0.04), p=0.0321
Change from week 3 to week 6 within treatment group	-0.07 (-0.26 to 0.11), p=0.4509	-0.27 (-0.45 to -0.09), p=0.0038	-0.14 (-0.33 to 0.06), p=0.1659
**Food present**			
Baseline	3.58	3.35	3.72
Change at week 3	-0.01 (0.15)	-0.37 (0.15)	-1.19 (0.17)
Vs placebo		-0.36 (-0.77 to 0.05), p=0.0833	-1.18 (-1.61 to -0.76), p<0.0001
Vs liraglutide	..	..	-0.82 (-1.25 to -0.39), p=0.0002
Change at week 6	-0.41 (0.15)	-0.74 (0.15)	-1.44 (0.16)
Vs placebo	..	-0.33 (-0.72 to 0.06), p=0.0965	-1.03 (-1.43 to -0.63), p<0.0001
Vs liraglutide	..	..	-0.70 (-1.10 to -0.29), p=0.0009
Change from week 3 to week 6 within treatment group	-0.41 (-0.62 to -0.20), p=0.0002	-0.38 (-0.58 to -0.17), p=0.0005	-0.25 (-0.47 to -0.03), p=0.0269
**Food tasted**			
Baseline	2.95	2.96	3.12
Change at week 3	0.00 (0.10)	-0.17 (0.11)	-0.86 (0.11)
Vs placebo	..	-0.17 (-0.45 to 0.11), p=0.2235	-0.86 (-1.15 to -0.57), p<0.0001
Vs liraglutide	..	..	-0.69 (-0.98 to -0.40), p<0.0001
Change at week 6	-0.08 (0.12)	-0.47 (0.12)	-0.98 (0.12)
Vs placebo	..	-0.39 (-0.70 to -0.08), p=0.0147	-0.90 (-1.23 to -0.58), p<0.0001
Vs liraglutide	..	..	-0.51 (-0.83 to -0.19), p=0.0022
Change from week 3 to week 6 within treatment group	-0.08 (-0.26 to 0.11), p=0.3950	-0.30 (-0.48 to -0.12), p=0.0015	-0.12 (-0.31 to 0.07), p=0.2223

Data are mean of absolute values at baseline, least squares mean (standard error) change from baseline, and difference in least squares mean (95% CI) vs placebo and liraglutide. CI=confidence interval; n=number of participants who were randomised and received at least one dose of study treatment.

Table S7. Barratt Impulsiveness Scale subscales

	Placebo (n=39)	Liraglutide (n=38)	Tirzepatide (n=37)
**First-order effects**			
Attention			
Baseline	2.17	2.15	2.32
Change at Week 3	0.20 (0.05)	0.11 (0.05)	0.03 (0.06)
Vs placebo	..	-0.09 (-0.23 to 0.05), p=0.1936	-0.17 (-0.32 to -0.03), p=0.0213
Vs liraglutide	..	..	-0.08 (-0.23 to 0.07), p=0.2837
Change at Week 6	0.04 (0.06)	0.13 (0.06)	0.03 (0.07)
Vs placebo	..	0.10 (-0.07 to 0.26), p=0.2500	0.00 (-0.17 to 0.17), p=0.9695
Vs liraglutide	..	..	-0.10 (-0.27 to 0.07), p=0.2518
Change from week 3 to week 6 within treatment group	-0.17 (-0.29 to -0.05), p=0.0069	0.02 (-0.10 to 0.14), p=0.7360	0.00 (-0.13 to 0.13, p=1.0000
Cognitive complexity			
Baseline	2.49	2.37	2.57
Change at Week 3	0.08 (0.06)	0.06 (0.06)	0.04 (0.06)
Vs placebo	..	-0.02 (-0.17 to 0.13), p=0.7806	-0.04 (-0.20 to 0.11), p=0.5792
Vs liraglutide	..	..	-0.02 (-0.18 to 0.13), p=0.7772
Change at Week 6	-0.01 (0.05)	0.02 (0.05)	-0.02 (0.06)
Vs placebo	..	0.03 (-0.11 to 0.18); p=0.6524	-0.01 (-0.15 to 0.14); p=0.9285
Vs liraglutide	..	..	-0.04 (-0.19 to 0.11); p=0.6008
Change from week 3 to week 6 within treatment group	-0.09 (-0.20 to 0.02), p =0.0986	-0.04 (-0.15 to 0.07). p=0.4737	-0.06 (-0.17 to 0.06), p=0.3397
Cognitive instability			
Baseline	2.03	2.13	1.96
Change at Week 3	0.20 (0.07)	-0.02 (0.07)	-0.07 (0.08)
Vs placebo	..	-0.22 (-0.41 to -0.03), p=0.0245	-0.27 (-0.47 to -0.07), p=0.0080
Vs liraglutide	..	..	-0.05 (-0.25 to 0.15), p=0.6175
Change at Week 6	0.19 (0.08)	0.03 (0.08)	-0.07 (0.08)
Vs placebo	..	-0.16 (-0.38 to 0.05), p=0.1333	-0.26 (-0.48 to -0.04), p=0.0215
Vs liraglutide	..	..	-0.10 (-0.32 to 0.12), p=0.3886
Change from week 3 to week 6 within treatment group	-0.01 (0.15 to 0.13), p=0.9036	0.05 (-0.09 to 0.18), p=0.4989	0.00 (-0.14 to 0.14), p=1.0000
Self-control			
Baseline	2.82	2.85	2.99
Change at Week 3	0.09 (0.05)	-0.03 (0.05)	0.00 (0.05)
Vs placebo	..	-0.12 (-0.25 to 0.02), p=0.0814	-0.09 (-0.23 to 0.05), p=0.2065
Vs liraglutide	..	..	0.03 (-0.11 to 0.17), p=0.6814
Change at Week 6	0.07 (0.06)	-0.01 (0.06)	0.01 (0.06)
Vs placebo	..	-0.08 (-0.23 to 0.07), p=0.2757	-0.06 (-0.21 to 0.09), p=0.4452
Vs liraglutide	..	..	0.02 (-0.13 to 0.18), p=0.7702
Change from week 3 to week 6 within treatment group	-0.01 (-0.13 to 0.10), p=0.8023	0.02 (-0.09 to 0.14), p=0.7051	0.02 (-0.11 to 0.14), p=0.8000
Motor impulsiveness			
Baseline	2.10	2.13	2.06
Change at Week 3	0.00 (0.05)	0.07 (0.05)	-0.02 (0.05)

Vs placebo	..	0.07 (-0.05 to 0.19), p=0.2594	-0.02 (-0.15 to 0.11), p=0.7825
Vs liraglutide	..	..	-0.09 (-0.22 to 0.04), p=0.1744
Change at Week 6	0.12 (0.06)	0.03 (0.06)	-0.12 (0.06)
Vs placebo	..	-0.09 (-0.25 to 0.07), p=0.2751	-0.24 (-0.40 to -0.07), p=0.0049
Vs liraglutide	..	..	-0.15 (-0.31 to 0.01), p=0.0719
Change from week 3 to week 6 within treatment group	0.12 (0.02 to 0.22), p=0.0143	-0.04 (-0.13 to 0.06), p=0.4449	-0.10 (-0.20 to 0.00), p=0.0547
Perseverance			
Baseline	1.85	1.93	1.84
Change at Week 3	-0.02 (0.04)	-0.02 (0.04)	-0.09 (0.05)
Vs placebo	..	0.00 (-0.11 to 0.12), p=0.9741	-0.07 (-0.19 to 0.05), p=0.2500
Vs liraglutide	..	..	-0.07 (-0.19 to 0.05), p=0.2396
Change at Week 6	-0.02 (0.05)	-0.10 (0.05)	-0.04 (0.05)
Vs placebo	..	-0.08 (-0.21 to 0.04), p=0.1960	-0.02 (-0.15 to 0.11), p=0.7853
Vs liraglutide	..	..	0.07 (-0.07 to 0.20), p=0.3226
Change from week 3 to week 6 within treatment group	0.00 (-0.09 to 0.10), p=0.9614	-0.08 (-0.18 to 0.01), p=0.0823	0.05 (-0.05 to 0.15), p=0.2806
**Second-order effects**			
Attentional impulsiveness			
Baseline	2.12	2.14	2.19
Change at Week 3	0.20 (0.04)	0.07 (0.04)	-0.01 (0.05)
Vs placebo	..	-0.13 (-0.25 to -0.01), p=0.0293	-0.21 (-0.34 to -0.09), p=0.0008
Vs liraglutide	..	..	-0.08 (-0.21 to 0.04), p=0.1841
Change at Week 6	0.09 (0.05)	0.10 (0.05)	-0.01 (0.05)
Vs placebo	..	0.01 (-0.12 to 0.15), p=0.8778	-0.10 (-0.24 to 0.04), p=0.1440
Vs liraglutide	..	..	-0.11 (-0.25 to 0.02), p=0.1061
Change from week 3 to week 6 within treatment group	-0.11 (-0.20 to -0.02), p=0.0175	0.03 (-0.06 to 0.12), p=0.4889	0.00 (-0.09 to 0.09), p=1.0000
Non-planning impulsiveness			
Baseline	2.67	2.63	2.80
Change at Week 3	0.08 (0.04)	0.02 (0.04)	0.01 (0.05)
Vs placebo	..	-0.06 (-0.18 to 0.05), p=0.2668	-0.07 (-0.19 to 0.05), p=0.2651
Vs liraglutide	..	..	0.00 (-0.12 to 0.11), p=0.9549
Change at Week 6	0.03 (0.04)	0.01 (0.04)	-0.01 (0.04)
Vs placebo	..	-0.02 (-0.13 to 0.09), p=0.7214	-0.04 (-0.15 to 0.08), p=0.5485
Vs liraglutide	..	..	-0.02 (-0.13 to 0.10), p=0.7960
Change from week 3 to week 6 within treatment group	-0.05 (-0.14 to 0.04), p=0.2736	-0.01 (-0.09 to 0.08), p=0.9023	-0.02 (-0.11 to 0.08), p=0.7149
Motor-impulsiveness			
Baseline	2.01	2.06	1.98
Change at Week 3	-0.01 (0.03)	0.03 (0.04)	-0.05 (0.04)
Vs placebo	..	0.04 (-0.05 to 0.13), p=0.3740	-0.04 (-0.14 to 0.06), p=0.4164
Vs liraglutide	..	..	-0.08 (-0.18 to 0.02), p=0.0979
Change at Week 6	0.07 (0.04)	-0.02 (0.04)	-0.09 (0.04)
Vs placebo	..	-0.09 (-0.20 to 0.02), p=0.1033	-0.16 (-0.28 to -0.05), p=0.0058
Vs liraglutide	..	..	-0.07 (-0.18 to 0.04), p=0.2226
Change from week 3 to week 6 within treatment group	0.08 (0.01 to 0.15), p=0.0242	-0.05 (-0.12 to 0.01), p=0.1208	-0.04 (-0.11 to 0.03), p=0.2421

Data are mean of absolute values at baseline, least squares mean (standard error) change from baseline, and difference in least squares mean (95% CI) vs placebo and liraglutide. CI=confidence interval; n=number of participants who were randomised and received at least one dose of study treatment.

Table S8. BOLD fMRI brain activation for highly palatable foods versus non-food objects

	Placebo (n=39)	Liraglutide (n=38)	Tirzepatide (n=37)
**Insula**			
Baseline	0.1754	0.3111	0.2102
Change at week 3	-0.0739 (0.0740)	0.0190 (0.0738)	-0.0301 (0.0771)
Vs placebo	..	0.0930 (-0.0441 to 0.2301), p=0.1806	0.0439 (-0.0994 to 0.1871), p=0.5437
Vs liraglutide	..	..	-0.0491 (-0.1846 to 0.0863), p=0.4720
Change at week 6	-0.0541 (0.0644)	-0.0604 (0.0699)	-0.1245 (0.0727)
Vs placebo	..	-0.0063 (-0.1174 to 0.1049), p=0.9110	-0.0704 (-0.1845 to 0.0437), p=0.2226
Vs liraglutide	..	..	-0.0641 (-0.1767 to 0.0485), p=0.2599
Change from week 3 to week 6 within treatment group	0.0198 (-0.0985 to 0.1382), p=0.7399	-0.0794 (-0.1870 to 0.0281), p=0.1455	-0.0944 (-0.2121 to 0.0233), p=0.1142
**Medial frontal gyrus**			
Baseline	0.2210	0.4219	0.2456
Change at week 3	0.0504 (0.0649)	0.0581 (0.0658)	0.0494 (0.0680)
Vs placebo	..	0.0077 (-0.0949 to 0.1102), p=0.8822	-0.0011 (-0.1079 to 0.1058), p=0.9842
Vs liraglutide	..	..	-0.0087 (-0.1111 to 0.0937), p=0.8657
Change at week 6	0.0139 (0.0625)	0.0787 (0.0681)	-0.0040 (0.0707)
Vs placebo	..	0.0647 (-0.0460 to 0.1755), p=0.2477	-0.0180 (-0.1318 to 0.0959), p=0.7541
Vs liraglutide	..	..	-0.0827 (-0.1953 to 0.0299), p=0.1476
Change from week 3 to week 6 within treatment group	-0.0365 (-0.1352 to 0.0622), p=0.4635	0.0206 (-0.0714 to 0.1125), p=0.6568	-0.0534 (-0.1534 to 0.0466), p=0.2901
**Superior temporal gyrus**			
Baseline	0.1922	0.3008	0.1851
Change at week 3	0.0132 (0.0838)	0.0621 (0.0839)	0.0174 (0.0881)
Vs placebo	..	0.0489 (-0.1129 to 0.2107), p=0.5490	0.0043 (-0.1650 to 0.1736), p=0.9599
Vs liraglutide	..	..	-0.0446 (-0.2068 to 0.1176), p=0.5854
Change at week 6	0.0102 (0.0706)	0.0132 (0.0768)	-0.0109 (0.0801)
Vs placebo	..	0.0030 (-0.1184 to 0.1244), p=0.9606	-0.0211 (-0.1457 to 0.1034), p=0.7361
Vs liraglutide	..	..	-0.0241 (-0.1475 to 0.0992), p=0.6974
Change from week 3 to week 6 within treatment group	-0.0030 (-0.1422 to 0.1363), p=0.9664	-0.0488 (-0.1773 to 0.0796), p=0.4510	-0.0284 (-0.1695 to 0.1127), p=0.6894
**Precentral gyrus**			
Baseline	0.1764	0.3015	0.1637
Change at week 3	0.0441 (0.0580)	0.0845 (0.0593)	0.0249 (0.0616)
Vs placebo	..	0.0404 (-0.0617 to 0.1425), p=0.4330	-0.0192 (-0.1259 to 0.0874), p=0.7203
Vs liraglutide	..	..	-0.0596 (-0.1619 to 0.0426), p=0.2488
Change at week 6	0.0437 (0.0532)	0.0470 (0.0583)	0.0256 (0.0604)
Vs placebo	..	0.0033 (-0.0897 to 0.0964), p=0.9432	-0.0181 (-0.1139 to 0.0778), p=0.7077
Vs liraglutide	..	..	-0.0214 (-0.1161 to 0.0733), p=0.6534
Change from week 3 to week 6 within treatment group	-0.0005 (-0.0872 to 0.0863), p=0.9915	-0.0375 (-0.1176 to 0.0426), p=0.3529	0.0007 (-0.0865 to 0.0879), p=0.9871
**Cingulate gyrus**			
Baseline	0.1705	0.3270	0.1781
Change at week 3	0.0283 (0.0615)	0.0967 (0.0623)	0.0214 (0.0643)
Vs placebo	..	0.0684 (-0.0267 to 0.1635), p=0.1563	-0.0070 (-0.1059 to 0.0919), p=0.8886
Vs liraglutide	..	..	-0.0754 (-0.1704 to 0.0196), p=0.1182
Change at week 6	0.0122 (0.0598)	0.0632 (0.0653)	0.0053 (0.0676)
Vs placebo	..	0.0510 (-0.0558 to 0.1577), p=0.3444	-0.0069 (-0.1165 to 0.1026), p=0.8998

Vs liraglutide	..	..	-0.0579 (-0.1664 to 0.0506), p=0.2910
Change from week 3 to week 6 within treatment group	-0.0161 (-0.1111 to 0.0789), p=0.7357	-0.0335 (-0.1222 to 0.0551), p=0.4524	-0.0161 (-0.1126 to 0.0804), p=0.7395
**Hippocampus**			
Baseline	0.1400	0.2811	0.1941
Change at week 3	0.0133 (0.0929)	0.0801 (0.0916)	-0.0438 (0.0967)
Vs placebo	..	0.0668 (-0.1239 to 0.2576), p=0.4875	-0.0571 (-0.2570 to 0.1427), p=0.5707
Vs liraglutide	..	..	-0.1240 (-0.3149 to 0.0669), p=0.1997
Change at week 6	0.0257 (0.0722)	0.0349 (0.0784)	-0.0334 (0.0818)
Vs placebo	..	0.0092 (-0.1133 to 0.1317), p=0.8816	-0.0591 (-0.1851 to 0.0668), p=0.3524
Vs liraglutide	..	..	-0.0683 (-0.1924 to 0.0558), p=0.2762
Change from week 3 to week 6 within treatment group	0.0124 (-0.1494 to 0.1743), p=0.8789	-0.0452 (-0.1945 to 0.1041), p=0.5486	0.0105 (-0.1543 to 0.1752), p=0.8998
**Putamen**			
Baseline	0.0842	0.1331	0.1178
Change at week 3	-0.0063 (0.0457)	0.0517 (0.0450)	0.0009 (0.0467)
Vs placebo	..	0.0581 (-0.0201 to 0.1363), p=0.1432	0.0073 (-0.0747 to 0.0892), p=0.8605
Vs liraglutide	..	..	-0.0508 (-0.1281 to 0.0265), p=0.1942
Change at week 6	0.0326 (0.0410)	-0.0077 (0.0446)	-0.0072 (0.0462)
Vs placebo	..	-0.0402 (-0.1124 to 0.0319), p=0.2702	-0.0398 (-0.1144 to 0.0349), p=0.2919
Vs liraglutide	..	..	0.0005 (-0.0722 to 0.0732), p=0.9898
Change from week 3 to week 6 within treatment group	0.0389 (-0.0394 to 0.1172), p=0.3259	-0.0594 (-0.1311 to 0.0122), p=0.1027	-0.0081 (-0.0869 to 0.0706), p=0.8373
**Orbitofrontal cortex**			
Baseline	0.2084	0.3681	0.2266
Change at week 3	0.0387 (0.0558)	0.0514 (0.0563)	0.0425 (0.0583)
Vs placebo	..	0.0127 (-0.0763 to 0.1017), p=0.7770	0.0038 (-0.0890 to 0.0966), p=0.9353
Vs liraglutide	..	..	-0.0089 (-0.0978 to 0.0800), p=0.8424
Change at week 6	-0.0005 (0.0533)	0.0457 (0.0580)	0.0033 (0.0602)
Vs placebo	..	0.0462 (-0.0480 to 0.1404), p=0.3316	0.0038 (-0.0931 to 0.1007), p=0.9380
Vs liraglutide	..	..	-0.0424 (-0.1383 to 0.0534), p=0.3805
Change from week 3 to week 6 within treatment group	-0.0392 (-0.1260 to 0.0476), p=0.3714	-0.0057 (-0.0866 to 0.0752), p=0.8891	-0.0392 (-0.1274 to 0.0490), p=0.3777

Data are mean of absolute values at baseline, least squares mean (standard error) change from baseline, and difference in least squares mean (95% CI) vs placebo and liraglutide. For each scan and each region, the mean of positive voxels was taken within each of the region separately. No positive voxels (Food > Non-food) were identified for the ventral striatum; therefore, statistical analysis was not done on this region. BOLD=blood oxygenation level dependent; fMRI=functional magnetic resonance imaging; n=number of participants who were randomised and received at least one dose of study treatment.

Table S9. BOLD fMRI brain activation for high-fat/high-sugar foods versus non-food objects

	Placebo (n=39)	Liraglutide (n=38)	Tirzepatide (n=37)
**Insula**			
Baseline	0.1167	0.1661	0.1580
Change at week 3	0.0124 (0.0291)	0.0199 (0.0268)	-0.0397 (0.0298)
Vs placebo	..	0.0076 (-0.0689 to 0.0841), p=0.8442	-0.0521 (-0.1327 to 0.0285), p=0.2018
Vs liraglutide	..	..	-0.0597 (-0.1364 to 0.0170), p=0.1254
Change at week 6	-0.0090 (0.0286)	-0.0097 (0.0281)	-0.0553 (0.0306)
Vs placebo	..	-0.0007 (-0.0781 to 0.0768), p=0.9865	-0.0463 (-0.1269 to 0.0342), p=0.2554
Vs liraglutide	..	..	-0.0457 (-0.1249 to 0.0336), p=0.2545
Change from week 3 to week 6 within treatment group	-0.0214 (-0.0970 to 0.0543), p=0.5753	-0.0296 (-0.1011 to 0.0419), p=0.4116	-0.0156 (-0.0937 to 0.0625), p=0.6911
**Medial frontal gyrus**			
Baseline	0.1404	0.2162	0.1501
Change at week 3	0.0595 (0.0334)	-0.0103 (0.0311)	-0.0408 (0.0344)
Vs placebo	..	-0.0698 (-0.1584 to 0.0188), p=0.1207	-0.1003 (-0.1926 to -0.0080), p=0.0335
Vs liraglutide	..	..	-0.0305 (-0.1192 to 0.0582), p=0.4953
Change at week 6	-0.0032 (0.0304)	0.0219 (0.0300)	-0.0826 (0.0326)
Vs placebo	..	0.0250 (-0.0572 to 0.1073), p=0.5461	-0.0794 (-0.1640 to 0.0052), p=0.0654
Vs liraglutide	..	..	-0.1044 (-0.1884 to -0.0204), p=0.0156
Change from week 3 to week 6 within treatment group	-0.0626 (-0.1397 to 0.0144), p=0.1093	0.0322 (-0.0398 to 0.1042), p=0.3746	-0.0417 (-0.1200 to 0.0366), p=0.2906
**Superior temporal gyrus**			
Baseline	0.1393	0.1956	0.1426
Change at week 3	0.0666 (0.0462)	0.0277 (0.0425)	-0.0495 (0.0472)
Vs placebo	..	-0.0388 (-0.1620 to 0.0843), p=0.5320	-0.1161 (-0.2453 to 0.0132), p=0.0776
Vs liraglutide	..	..	-0.0773 (-0.2010 to 0.0465), p=0.2176
Change at week 6	-0.0132 (0.0300)	-0.0206 (0.0297)	-0.0504 (0.0322)
Vs placebo	..	-0.0074 (-0.0882 to 0.0734), p=0.8558	-0.0372 (-0.1204 to 0.0460), p=0.3757
Vs liraglutide	..	..	-0.0298 (-0.1123 to 0.0526), p=0.4735
Change from week 3 to week 6 within treatment group	-0.0797 (-0.1910 to 0.0316), p=0.1575	-0.0483 (-0.1529 to 0.0563), p=0.3598	-0.0008 (-0.1165 to 0.1149), p=0.9885
**Precentral gyrus**			
Baseline	0.1249	0.2276	0.1412
Change at week 3	0.0304 (0.0411)	-0.0172 (0.0380)	-0.0817 (0.0422)
Vs placebo	..	-0.0476 (-0.1571 to 0.0619), p=0.3899	-0.1120 (-0.2267 to 0.0026), p=0.0552
Vs liraglutide	..	..	-0.0645 (-0.1745 to 0.0456), p=0.2472
Change at week 6	-0.0380 (0.0278)	-0.0441 (0.0276)	-0.0921 (0.0299)
Vs placebo	..	-0.0062 (-0.0810 to 0.0687), p=0.8697	-0.0542 (-0.1307 to 0.0223), p=0.1620
Vs liraglutide	..	..	-0.0480 (-0.1243 to 0.0283), p=0.2131
Change from week 3 to week 6 within treatment group	-0.0684 (-0.1550 to 0.0183), p=0.1196	-0.0269 (-0.1070 to 0.0531), p=0.5020	-0.0105 (-0.0982 to 0.0772), p=0.8109
**Cingulate gyrus**			
Baseline	0.1320	0.2024	0.1337
Change at week 3	0.0391 (0.0284)	0.0051 (0.0264)	-0.0476 (0.0292)
Vs placebo	..	-0.0340 (-0.1090 to 0.0410), p=0.3695	-0.0867 (-0.1650 to -0.0083), p=0.0306
Vs liraglutide	..	..	-0.0526 (-0.1279 to 0.0227), p=0.1679
Change at week 6	-0.0001 (0.0261)	0.0058 (0.0259)	-0.0672 (0.0281)
Vs placebo	..	0.0059 (-0.0649 to 0.0768), p=0.8685	-0.0671 (-0.1400 to 0.0058), p=0.0707

Vs liraglutide	..	..	-0.0730 (-0.1455 to -0.0005), p=0.0486
Change from week 3 to week 6 within treatment group	-0.0392 (-0.1056 to 0.0272), p=0.2431	0.0007 (-0.0614 to 0.0629), p=0.9816	-0.0196 (-0.0873 to 0.0480), p=0.5635
**Hippocampus**			
Baseline	0.0727	0.1354	0.1254
Change at week 3	0.0722 (0.0314)	0.0222 (0.0292)	-0.0297 (0.0325)
Vs placebo	..	-0.0499 (-0.1325 to 0.0326), p=0.2323	-0.1019 (-0.1887 to -0.0150), p=0.0221
Vs liraglutide	..	..	-0.0519 (-0.1344 to 0.0305), p=0.2135
Change at week 6	0.0436 (0.0341)	0.0355 (0.0336)	-0.0190 (0.0367)
Vs placebo	..	-0.0081 (-0.1008 to 0.0847), p=0.8629	-0.0625 (-0.1589 to 0.0339), p=0.2005
Vs liraglutide	..	..	-0.0544 (-0.1493 to 0.0404), p=0.2565
Change from week 3 to week 6 within treatment group	-0.0286 (-0.1040 to 0.0468), p=0.4522	0.0132 (-0.0576 to 0.0841), p=0.7104	0.0107 (-0.0658 to 0.0873), p=0.7806
**Putamen**			
Baseline	0.0623	0.0894	0.1097
Change at week 3	-0.0009 (0.0192)	-0.0091 (0.0177)	-0.0349 (0.0198)
Vs placebo	..	-0.0082 (-0.0588 to 0.0425), p=0.7494	-0.0340 (-0.0875 to 0.0194), p=0.2088
Vs liraglutide	..	..	-0.0259 (-0.0769 to 0.0252), p=0.3158
Change at week 6	-0.0119 (0.0169)	-0.0280 (0.0167)	-0.0312 (0.0183)
Vs placebo	..	-0.0160 (-0.0615 to 0.0294), p=0.4846	-0.0193 (-0.0667 to 0.0281), p=0.4202
Vs liraglutide	..	..	-0.0033 (-0.0501 to 0.0436), p=0.8902
Change from week 3 to week 6 within treatment group	-0.0110 (-0.0573 to 0.0352), p=0.6365	-0.0189 (-0.0623 to 0.0245), p=0.3880	0.0037 (-0.0437 to 0.0512), p=0.8761
**Orbitofrontal cortex**			
Baseline	0.1345	0.1897	0.1408
Change at week 3	0.0547 (0.0293)	-0.0069 (0.0272)	-0.0341 (0.0301)
Vs placebo	..	-0.0616 (-0.1393 to 0.0160), p=0.1179	-0.0889 (-0.1699 to -0.0078), p=0.0321
Vs liraglutide	..	..	-0.0272 (-0.1051 to 0.0506), p=0.4883
Change at week 6	-0.0067 (0.0254)	0.0138 (0.0251)	-0.0685 (0.0272)
Vs placebo	..	0.0205 (-0.0481 to 0.0890), p=0.5528	-0.0618 (-0.1324 to 0.0088), p=0.0852
Vs liraglutide	..	..	-0.0823 (-0.1524 to -0.0123), p=0.0219
Change from week 3 to week 6 within treatment group	-0.0614 (-0.1273 to 0.0046), p=0.0675	0.0207 (-0.0407 to 0.0822), p=0.5018	-0.0343 (-0.1013 to 0.0326), p=0.3079

Data are mean of absolute values at baseline, least squares mean (standard error) change from baseline, and difference in least squares mean (95% CI) vs placebo and liraglutide. For each scan and each region, the mean of positive voxels was taken within each of the regions separately. No positive voxels (Food > Non-food) were identified for the ventral striatum; therefore, statistical analysis was not done on this region. BOLD=blood oxygenation level dependent; CI=confidence interval; fMRI=functional magnetic resonance imaging; n=number of participants who were randomised and received at least one dose of study treatment.

Table S10. BOLD fMRI brain activation for high-fat/high-carbohydrate foods versus non-food objects

	Placebo (n=39)	Liraglutide (n=38)	Tirzepatide (n=37)
**Insula**			
Baseline	0.1519	0.1613	0.1425
Change at week 3	-0.0162 (0.0418)	0.0366 (0.0386)	0.0577 (0.0429)
Vs placebo	..	0.0528 (-0.0580 to 0.1636), p=0.3455	0.0738 (-0.0428 to 0.1905), p=0.2113
Vs liraglutide	..	..	0.0210 (-0.0906 to 0.1326), p=0.7085
Change at week 6	0.0098 (0.0314)	0.0130 (0.0311)	-0.0139 (0.0339)
Vs placebo	..	0.0032 (-0.0809 to 0.0874), p=0.9389	-0.0236 (-0.1109 to 0.0637), p=0.5912
Vs liraglutide	..	..	-0.0269 (-0.1136 to 0.0598), p=0.5388
Change from week 3 to week 6 within treatment group	0.0260 (-0.0731 to 0.1250), p=0.6039	-0.0236 (-0.1164 to 0.0692), p=0.6140	-0.0715 (-0.1735 to 0.0305), p=0.1665
**Medial frontal gyrus**			
Baseline	0.1758	0.2076	0.1635
Change at week 3	0.0192 (0.0334)	-0.0004 (0.0308)	0.0187 (0.0344)
Vs placebo	..	-0.0195 (-0.1074 to 0.0684), p=0.6595	-0.0004 (-0.0930 to 0.0921), p=0.9924
Vs liraglutide	..	..	0.0191 (-0.0694 to 0.1076), p=0.6690
Change at week 6	-0.0014 (0.0336)	0.0154 (0.0332)	-0.0235 (0.0362)
Vs placebo	..	0.0168 (-0.0743 to 0.1078), p=0.7146	-0.0221 (-0.1166 to 0.0724), p=0.6424
Vs liraglutide	..	..	-0.0389 (-0.1328 to 0.0550), p=0.4118
Change from week 3 to week 6 within treatment group	-0.0205 (-0.1112 to 0.0702), p=0.6532	0.0158 (-0.0703 to 0.1018), p=0.7156	-0.0422 (-0.1364 to 0.0520), p=0.3736
**Superior temporal gyrus**			
Baseline	0.1704	0.1612	0.1455
Change at week 3	0.0027 (0.0451)	0.0419 (0.0417)	0.0434 (0.0465)
Vs placebo	..	0.0392 (-0.0799 to 0.1582), p=0.5146	0.0407 (-0.0849 to 0.1662), p=0.5209
Vs liraglutide	..	..	0.0015 (-0.1187 to 0.1217), p=0.9802
Change at week 6	0.0219 (0.0375)	0.0612 (0.0371)	0.0288 (0.0406)
Vs placebo	..	0.0393 (-0.0615 to 0.1400), p=0.4401	0.0069 (-0.0978 to 0.1116), p=0.8961
Vs liraglutide	..	..	-0.0324 (-0.1362 to 0.0715), p=0.5364
Change from week 3 to week 6 within treatment group	0.0192 (-0.0853 to 0.1237), p=0.7153	0.0193 (-0.0783 to 0.1169), p=0.6944	-0.0146 (-0.1212 to 0.0921), p=0.7859
**Precentral gyrus**			
Baseline	0.1576	0.1558	0.1344
Change at week 3	-0.0162 (0.0325)	0.0248 (0.0300)	-0.0023 (0.0333)
Vs placebo	..	0.0410 (-0.0445 to 0.1264), p=0.3430	0.0139 (-0.0760 to 0.1037), p=0.7597
Vs liraglutide	..	..	-0.0271 (-0.1131 to 0.0589), p=0.5319
Change at week 6	-0.0075 (0.0303)	0.0241 (0.0300)	0.0060 (0.0326)
Vs placebo	..	0.0316 (-0.0504 to 0.1136), p=0.4445	0.0136 (-0.0714 to 0.0986), p=0.7512
Vs liraglutide	..	..	-0.0181 (-0.1025 to 0.0663), p=0.6710
Change from week 3 to week 6 within treatment group	0.0086 (-0.0738 to 0.0910), p=0.8357	-0.0007 (-0.0784 to 0.0770), p=0.9855	0.0083 (-0.0766 to 0.0933), p=0.8453
**Cingulate gyrus**			
Baseline	0.1460	0.1956	0.1465
Change at week 3	0.0138 (0.0320)	0.0398 (0.0296)	0.0092 (0.0329)
Vs placebo	..	0.0261 (-0.0582 to 0.1104), p=0.5396	-0.0046 (-0.0930 to 0.0839), p=0.9186
Vs liraglutide	..	..	-0.0306 (-0.1157 to 0.0544), p=0.4753
Change at week 6	0.0022 (0.0346)	0.0195 (0.0342)	0.0188 (0.0371)
Vs placebo	..	0.0173 (-0.0770 to 0.1115), p=0.7158	0.0165 (-0.0809 to 0.1140), p=0.7361

Vs liraglutide	..	..	-0.0007 (-0.0979 to 0.0964), p=0.9879
Change from week 3 to week 6 within treatment group	-0.0115 (-0.1031 to 0.0800), p=0.8026	-0.0203 (-0.1075 to 0.0669), p=0.6434	0.0096 (-0.0859 to 0.1050), p=0.8419
**Hippocampus**			
Baseline	0.1038	0.1198	0.1147
Change at week 3	0.0174 (0.0423)	0.0566 (0.0390)	0.0032 (0.0433)
Vs placebo	..	0.0392 (-0.0728 to 0.1512), p=0.4879	-0.0143 (-0.1321 to 0.1036), p=0.8100
Vs liraglutide	..	..	-0.0535 (-0.1663 to 0.0594), p=0.3482
Change at week 6	0.0395 (0.0333)	0.0385 (0.0328)	0.0492 (0.0358)
Vs placebo	..	-0.0011 (-0.0906 to 0.0885) p=0.9812	0.0097 (-0.0832 to 0.1025) p=0.8361
Vs liraglutide	..	..	0.0107 (-0.0814 to 0.1029) p=0.8170
Change from week 3 to week 6 within treatment group	0.0221 (-0.0848 to 0.1289), p=0.6825	-0.0182 (-0.1191 to 0.0827), p=0.7208	0.0460 (-0.0651 to 0.1571), p=0.4121
**Putamen**			
Baseline	0.0842	0.0715	0.0914
Change at week 3	-0.0279 (0.0237)	0.0174 (0.0220)	0.0047 (0.0243)
Vs placebo	..	0.0452 (-0.0173 to 0.1077), p=0.1536	0.0326 (-0.0330 to 0.0982), p=0.3255
Vs liraglutide	..	..	-0.0126 (-0.0756 to 0.0504), p=0.6909
Change at week 6	0.0115 (0.0250)	-0.0103 (0.0247)	0.0163 (0.0268)
Vs placebo	..	-0.0219 (-0.0901 to 0.0463), p=0.5244	0.0047 (-0.0659 to 0.0753), p=0.8942
Vs liraglutide	..	..	0.0266 (-0.0437 to 0.0969), p-=0.4531
Change from week 3 to week 6 within treatment group	0.0394 (-0.0293 to 0.1082), p=0.2578	-0.0277 (-0.0933 to 0.0379), p=0.4037	0.0115 (-0.0605 to 0.0836), p=0.7506
**Orbitofrontal cortex**			
Baseline	0.1664	0.1875	0.1529
Change at week 3	0.0179 (0.0288)	-0.0017 (0.0266)	0.0169 (0.0297)
Vs placebo	..	-0.0196 (-0.0954 to 0.0562), p=0.6078	-0.0011 (-0.0809 to 0.0788), p=0.9790
Vs liraglutide	..	..	0.0185 (-0.0578 to 0.0949), p=0.6298
Change at week 6	0.0010 (0.0309)	-0.0012 (0.0305)	-0.0169 (0.0333)
Vs placebo	..	-0.0022 (-0.0863 to 0.0819), p=0.9584	-0.0179 (-0.1051 to 0.0694), p=0.6847
Vs liraglutide	..	..	-0.0156 (-0.1023 to 0.0711), p=0.7202
Change from week 3 to week 6 within treatment group	-0.0170 (-0.1003 to 0.0664), p=0.6854	0.0004 (-0.0791 to 0.0799), p=0.9917	-0.0338 (-0.1209 to 0.0533), p=0.4401

Data are mean of absolute values at baseline, least squares mean (standard error) change from baseline, and difference in least squares mean (95% CI) vs placebo and liraglutide. For each scan and each region, the mean of positive voxels was taken within each of the regions separately. No positive voxels (Food > Non-food) were identified for the ventral striatum; therefore, statistical analysis was not done on this region. BOLD=blood oxygenation level dependent; CI=confidence interval; fMRI=functional magnetic resonance imaging; n=number of participants who were randomised and received at least one dose of study treatment.

Table S11. BOLD fMRI brain activation for high-fat/low-carbohydrate foods versus non-food objects

	Placebo (n=39)	Liraglutide (n=38)	Tirzepatide (n=37)
**Insula**			
Baseline	0.1522	0.1756	0.1485
Change at week 3	0.0130 (0.0309)	-0.0031 (0.0286)	-0.0115 (0.0318)
Vs placebo	..	-0.0161 (-0.0972 to 0.0651), p=0.6942	-0.0245 (-0.1097 to 0.0606), p=0.5679
Vs liraglutide	..	..	-0.0085 (-0.0900 to 0.0731), p=0.8369
Change at week 6	0.0000 (0.0329)	-0.0267 (0.0325)	0.0024 (0.0353)
Vs placebo	..	-0.0267 (-0.1160 to 0.0625), p=0.5524	0.0024 (-0.0902 to 0.0950), p=0.9592
Vs liraglutide	..	..	0.0291 (-0.0627 to 0.1210), p=0.5295
Change from week 3 to week 6 within treatment group	-0.0130 (-0.0946 to 0.0686), p=0.7520	-0.0237 (-0.1009 to 0.0536), p=0.5434	0.0139 (-0.0702 to 0.0980), p=0.7426
**Medial frontal gyrus**			
Baseline	0.1717	0.2339	0.1587
Change at week 3	0.0437 (0.0339)	-0.0324 (0.0314)	-0.0205 (0.0350)
Vs placebo	..	-0.0761 (-0.1654 to 0.0133), p=0.0942	-0.0642 (-0.1578 to 0.0295), p=0.1762
Vs liraglutide	..	..	0.0119 (-0.0783 to 0.1020), p=0.7936
Change at week 6	-0.0548 (0.0331)	-0.0018 (0.0327)	-0.0295 (0.0356)
Vs placebo	..	0.0530 (-0.0367 to 0.1428), p=0.2424	0.0253 (-0.0673 to 0.1178), p=0.5875
Vs liraglutide	..	..	-0.0278 (-0.1201 to 0.0646), p=0.5513
Change from week 3 to week 6 within treatment group	-0.0985 (-0.1836 to -0.0134), p=0.0239	0.0306 (-0.0495 to 0.1108), p=0.4487	-0.0090 (-0.0964 to 0.0784), p=0.8375
**Superior temporal gyrus**			
Baseline	0.1395	0.1747	0.1686
Change at week 3	0.0300 (0.0456)	0.0085 (0.0420)	-0.0372 (0.0468)
Vs placebo	..	-0.0215 (-0.1416 to 0.0986), p=0.7226	-0.0672 (-0.1936 to 0.0592), p=0.2929
Vs liraglutide	..	..	-0.0457 (-0.1664 to 0.0750), p=0.4531
Change at week 6	-0.0123 (0.0392)	0.0028 (0.0387)	0.0215 (0.0422)
Vs placebo	..	0.0151 (-0.0904 to 0.1205), p=0.7762	0.0338 (-0.0757 to 0.1433), p=0.5403
Vs liraglutide	..	..	0.0187 (-0.0896 to 0.1270), p=0.7315
Change from week 3 to week 6 within treatment group	-0.0423 (-0.1489 to 0.0642), p=0.4315	-0.0058 (-0.1055 to 0.0940), p=0.9087	0.0587 (-0.0502 to 0.1676), p=0.2862
**Precentral gyrus**			
Baseline	0.1604	0.2009	0.1571
Change at week 3	-0.0106 (0.0367)	-0.0389 (0.0338)	-0.0553 (0.0376)
Vs placebo	..	-0.0283 (-0.1257 to 0.0691), p=0.5651	-0.0447 (-0.1471 to 0.0577), p=0.3877
Vs liraglutide	..	..	-0.0164 (-0.1145 to 0.0817), p=0.7401
Change at week 6	-0.0795 (0.0276)	-0.0312 (0.0273)	-0.0423 (0.0297)
Vs placebo	..	0.0483 (-0.0261 to 0.1227), p=0.1996	0.0372 (-0.0398 to 0.1141), p=0.3385
Vs liraglutide	..	..	-0.0111 (-0.0877 to 0.0654), p=0.7729
Change from week 3 to week 6 within treatment group	-0.0689 (-0.1616 to 0.0238), p=0.1428	0.0077 (-0.0799 to 0.0953), p=0.8610	0.0130 (-0.0837 to 0.1096), p=0.7890
**Cingulate gyrus**			
Baseline	0.1569	0.2032	0.1431
Change at week 3	0.0051 (0.0293)	-0.0035 (0.0271)	-0.0493 (0.0301)
Vs placebo	..	-0.0086 (-0.0861 to 0.0689), p=0.8265	-0.0543 (-0.1357 to 0.0271), p=0.1876
Vs liraglutide	..	..	-0.0458 (-0.1239 to 0.0323), p=0.2467
Change at week 6	-0.0435 (0.0268)	-0.0294 (0.0265)	-0.0251 (0.0288)
Vs placebo	..	0.0141 (-0.0587 to 0.0868), p=0.7009	0.0184 (-0.0569 to 0.0936), p=0.6281

Vs liraglutide	..	..	0.0043 (-0.0706 to 0.0792), p=0.9093
Change from week 3 to week 6 within treatment group	-0.0485 (-0.1285 to 0.0314), p=0.2305	-0.0259 (-0.1020 to 0.0501), p=0.4990	0.0242 (-0.0595 to 0.1078), p=0.5661
**Hippocampus**			
Baseline	0.1187	0.1463	0.1457
Change at week 3	-0.0048 (0.0313)	-0.0401 (0.0286)	-0.0315 (0.0318)
Vs placebo	..	-0.0352 (-0.1170 to 0.0466), p=0.3936	-0.0267 (-0.1128 to 0.0594), p=0.5386
Vs liraglutide	..	..	0.0085 (-0.0729 to 0.0900), p=0.8349
Change at week 6	-0.0058 (0.0309)	-0.0175 (0.0301)	-0.0063 (0.0328)
Vs placebo	..	-0.0117 (-0.0947 to 0.0713), p=0.7795	-0.0005 (-0.0869 to 0.0858), p=0.9901
Vs liraglutide	..	..	0.0112 (-0.0737 to 0.0960), p=0.7939
Change from week 3 to week 6 within treatment group	-0.0009 (-0.0747 to 0.0729), p=0.9802	0.0226 (-0.0452 to 0.0904), p=0.5084	0.0252 (-0.0482 to 0.0987), p=0.4960
**Putamen**			
Baseline	0.0876	0.1054	0.1103
Change at week 3	-0.0192 (0.0184)	-0.0414 (0.0170)	-0.0312 (0.0189)
Vs placebo	..	-0.0223 (-0.0706 to 0.0260), p=0.3617	-0.0120 (-0.0628 to 0.0388), p=0.6395
Vs liraglutide	..	..	0.0103 (-0.0383 to 0.0588), p=0.6753
Change at week 6	-0.0162 (0.0215)	-0.0330 (0.0212)	0.0065 (0.0230)
Vs placebo	..	-0.0168 (-0.0755 to 0.0419), p=0.5705	0.0227 (-0.0382 to 0.0837), p=0.4603
Vs liraglutide	..	..	0.0395 (-0.0210 to 0.1000), p=0.1974
Change from week 3 to week 6 within treatment group	0.0030 (-0.0530 to 0.0590), p=0.9155	0.0085 (-0.0451 to 0.0620), p=0.7538	0.0377 (-0.0209 to 0.0963), p=0.2040
**Orbitofrontal cortex**			
Baseline	0.1611	0.2027	0.1467
Change at week 3	0.0434 (0.0293)	-0.0291 (0.0271)	-0.0193 (0.0302)
Vs placebo	..	-0.0725 (-0.1498 to 0.0048), p=0.0658	-0.0626 (-0.1438 to 0.0185), p=0.1283
Vs liraglutide	..	..	0.0098 (-0.0682 to 0.0878), p=0.8028
Change at week 6	-0.0486 (0.0277)	-0.0084 (0.0274)	-0.0230 (0.0298)
Vs placebo	..	0.0402 (-0.0347 to 0.1152), p=0.2881	0.0256 (-0.0519 to 0.1030), p=0.5123
Vs liraglutide	..	..	-0.0146 (-0.0919 to 0.0626), p=0.7065
Change from week 3 to week 6 within treatment group	-0.0920 (-0.1656 to -0.0183), p=0.0150	0.0207 (-0.0486 to 0.0901), p=0.5529	-0.0037 (-0.0795 to 0.0720), p=0.9218

Data are mean of absolute values at baseline, least squares mean (standard error) change from baseline, and difference in least squares mean (95% CI) vs placebo and liraglutide. For each scan and each region, the mean of positive voxels was taken within each of the regions separately. No positive voxels (Food > Non-food) were identified for the ventral striatum; therefore, statistical analysis was not done on this region. BOLD=blood oxygenation level dependent; CI=confidence interval; fMRI=functional magnetic resonance imaging; n=number of participants who were randomised and received at least one dose of study treatment.

Table S12. BOLD fMRI brain activation for high-sugar/low-fat foods versus non-food objects

	Placebo (n=39)	Liraglutide (n=38)	Tirzepatide (n=37)
**Insula**			
Baseline	0.1377	0.1846	0.1767
Change at week 3	-0.0232 (0.0312)	-0.0537 (0.0287)	-0.0259 (0.0319)
Vs placebo	..	-0.0305 (-0.1119 to 0.0509), p=0.4575	-0.0028 (-0.0882 to 0.0827), p=0.9489
Vs liraglutide	..	..	0.0278 (-0.0537 to 0.1092), p=0.4992
Change at week 6	0.0077 (0.0416)	-0.0060 (0.0409)	-0.0383 (0.0444)
Vs placebo	..	-0.0137 (-0.1277 to 0.1002), p=0.8111	-0.0459 (-0.1640 to 0.0722), p=0.4408
Vs liraglutide	..	..	-0.0322 (-0.1492 to 0.0848), p=0.5848
Change from week 3 to week 6 within treatment group	0.0308 (-0.0684 to 0.1300), p=0.5383	0.0476 (-0.0475 to 0.1428), p=0.3218	-0.0124 (-0.1159 to 0.0912), p=0.8127
**Medial frontal gyrus**			
Baseline	0.1600	0.2284	0.1824
Change at week 3	0.0044 (0.0300)	-0.0655 (0.0277)	-0.0562 (0.0307)
Vs placebo	..	-0.0699 (-0.1489 to 0.0091), p=0.0821	-0.0606 (-0.1432 to 0.0219), p=0.1476
Vs liraglutide	..	..	0.0093 (-0.0698 to 0.0883), p=0.8160
Change at week 6	-0.0028 (0.0343)	0.0060 (0.0338)	-0.0835 (0.0366)
Vs placebo	..	0.0089 (-0.0848 to 0.1025), p=0.8507	-0.0807 (-0.1774 to 0.0161), p=0.1010
Vs liraglutide	..	..	-0.0895 (-0.1856 to 0.0065), p=0.0672
Change from week 3 to week 6 within treatment group	-0.0073 (-0.0945 to 0.0800), p=0.8691	0.0715 (-0.0116 to 0.1547), p=0.0908	-0.0273 (-0.1181 to 0.0636), p=0.5515
**Superior temporal gyrus**			
Baseline	0.1505	0.2217	0.1936
Change at week 3	-0.0107 (0.0361)	-0.0436 (0.0331)	-0.0600 (0.0368)
Vs placebo	..	-0.0329 (-0.1271 to 0.0614), p=0.4897	-0.0493 (-0.1480 to 0.0495), p=0.3235
Vs liraglutide	..	..	-0.0164 (-0.1106 to 0.0778), p=0.7297
Change at week 6	-0.0029 (0.0453)	0.0016 (0.0446)	-0.0452 (0.0483)
Vs placebo	..	0.0045 (-0.1194 to 0.1283), p=0.9431	-0.0424 (-0.1707 to 0.0859), p=0.5124
Vs liraglutide	..	..	-0.0468 (-0.1740 to 0.0803), p=0.4653
Change from week 3 to week 6 within treatment group	0.0079 (-0.1024 to 0.1181), p=0.8874	0.0147 (-0.1003 to 0.1298), p=0.7991	0.0147 (-0.1003 to 0.1298), p=0.7991
**Precentral gyrus**			
Baseline	0.1470	0.1913	0.1731
Change at week 3	-0.0124 (0.0287)	-0.0359 (0.0264)	-0.0680 (0.0293)
Vs placebo	..	-0.0234 (-0.0985 to 0.0516), p=0.5360	-0.0556 (-0.1344 to 0.0232), p=0.1638
Vs liraglutide	..	..	-0.0322 (-0.1074 to 0.0430), p=0.3969
Change at week 6	-0.0177 (0.0333)	-0.0143 (0.0328)	-0.0562 (0.0356)
Vs placebo	..	0.0034 (-0.0874 to 0.0942), p=0.9410	-0.0385 (-0.1326 to 0.0555), p=0.4170
Vs liraglutide	..	..	-0.0419 (-0.1352 to 0.0514), p=0.3735
Change from week 3 to week 6 within treatment group	-0.0052 (-0.0885 to 0.0780), p=0.9008	0.0216 (-0.0578 to 0.1010), p=0.5894	0.0118 (-0.0748 to 0.0985), p=0.7857
**Cingulate gyrus**			
Baseline	0.1561	0.2125	0.1707
Change at week 3	-0.0193 (0.0261)	-0.0384 (0.0241)	-0.0692 (0.0268)
Vs placebo	..	-0.0190 (-0.0873 to 0.0492), p=0.5802	-0.0499 (-0.1213 to 0.0215), p=0.1677
Vs liraglutide	..	..	-0.0309 (-0.0992 to 0.0374), p=0.3709
Change at week 6	0.0175 (0.0365)	-0.0128 (0.0360)	-0.0539 (0.0390)
Vs placebo	..	-0.0303 (-0.1304 to 0.0699), p=0.5489	-0.0714 (-0.1752 to 0.0323), p=0.1742

Vs liraglutide	..	..	-0.0411 (-0.1441 to 0.0618), p=0.4285
Change from week 3 to week 6 within treatment group	0.0368 (-0.0465 to 0.1201), p=0.3817	0.0256 (-0.0542 to 0.1054), p=0.5250	0.0153 (-0.0713 to 0.1019), p=0.7257
**Hippocampus**			
Baseline	0.0933	0.1143	0.1366
Change at week 3	0.0207 (0.0301)	-0.0065 (0.0277)	0.0021 (0.0309)
Vs placebo	..	-0.0272 (-0.1054 to 0.0510), p=0.4911	-0.0186 (-0.1011 to 0.0639), p=0.6544
Vs liraglutide	..	..	0.0085 (-0.0700 to 0.0871), p=0.8289
Change at week 6	0.0235 (0.0372)	0.0183 (0.0366)	0.0124 (0.0397)
Vs placebo	..	-0.0051 (-0.1062 to 0.0960), p=0.9197	-0.0111 (-0.1163 to 0.0942), p=0.8346
Vs liraglutide	..	..	-0.0059 (-0.1100 to 0.0981), p=0.9098
Change from week 3 to week 6 within treatment group	0.0027 (-0.0786 to 0.0841), p=0.9465	0.0248 (-0.0524 to 0.1019), p=0.5235	0.0103 (-0.0731 to 0.0937), p=0.8060
**Putamen**			
Baseline	0.0735	0.0906	0.1186
Change at week 3	-0.0191 (0.0184)	-0.0400 (0.0170)	-0.0240 (0.0190)
Vs placebo	..	-0.0209 (-0.0689 to 0.0271), p=0.3885	-0.0049 (-0.0558 to 0.0459), p=0.8468
Vs liraglutide	..	..	0.0160 (-0.0323 to 0.0643), p=0.5127
Change at week 6	0.0036 (0.0271)	-0.0094 (0.0267)	0.0214 (0.0290)
Vs placebo	..	-0.0130 (-0.0874 to 0.0614), p=0.7285	0.0178 (-0.0596 to 0.0953), p=0.6478
Vs liraglutide	..	..	0.0308 (-0.0458 to 0.1075), p=0.4252
Change from week 3 to week 6 within treatment group	0.0226 (-0.0413 to 0.0865), p=0.4828	0.0305 (-0.0310 to 0.0921), p=0.3260	0.0454 (-0.0216 to 0.1124), p=0.1807
**Orbitofrontal cortex**			
Baseline	0.1529	0.2021	0.1729
Change at week 3	0.0064 (0.0262)	-0.0597 (0.0241)	-0.0523 (0.0268)
Vs placebo	..	-0.0661 (-0.1348 to 0.0026), p= 0.0591	-0.0587 (-0.1306 to 0.0132), p=0.1081
Vs liraglutide	..	..	0.0074 (-0.0613 to 0.0761), p=0.8309
Change at week 6	-0.0018 (0.0308)	0.0011 (0.0303)	-0.0702 (0.0329)
Vs placebo	..	0.0029 (-0.0811 to 0.0870), p=0.9446	-0.0683 (-0.1553 to 0.0187), p=0.1219
Vs liraglutide	..	..	-0.0713 (-0.1575 to 0.0150), p=0.1040
Change from week 3 to week 6 within treatment group	-0.0083 (-0.0855 to 0.0690), p=0.8323	0.0608 (-0.0130 to 0.1345), p=0.1051	-0.0179 (-0.0984 to 0.0626), p=0.6592

Data are mean of absolute values at baseline, least squares mean (standard error) change from baseline, and difference in least squares mean (95% CI) vs placebo and liraglutide. For each scan and each region, the mean of positive voxels was taken within each of the regions separately. No positive voxels (Food > Non-food) were identified for the ventral striatum; therefore, statistical analysis was not done on this region. BOLD=blood oxygenation level dependent; CI=confidence interval; fMRI=functional magnetic resonance imaging; n=number of participants who were randomised and received at least one dose of study treatment.

Table S13. BOLD fMRI brain activation for low-fat/high-carbohydrate foods versus non-food objects

	Placebo (n=39)	Liraglutide (n=38)	Tirzepatide (n=37)
**Insula**			
Baseline	0.1531	0.1747	0.1954
Change at week 3	-0.0480 (0.0231)	-0.0534 (0.0213)	-0.0644 (0.0237)
Vs placebo	..	-0.0054 (-0.0654 to 0.0546), p=0.8578	-0.0165 (-0.0798 to 0.0469), p=0.6065
Vs liraglutide	..	..	-0.0110 (-0.0712 to 0.0491), p=0.7160
Change at week 6	0.0066 (0.0398)	-0.0355 (0.0391)	-0.0118 (0.0424)
Vs placebo	..	-0.0421 (-0.1517 to 0.0675), p=0.4467	-0.0183 (-0.1324 to 0.0957), p=0.7494
Vs liraglutide	..	..	0.0237 (-0.0892 to 0.1367), p=0.6768
Change from week 3 to week 6 within treatment group	0.0545 (-0.0355 to 0.1446), p=0.2317	0.0179 (-0.0693 to 0.1050), p=0.6840	0.0526 (-0.0421 to 0.1473), p=0.2714
**Medial frontal gyrus**			
Baseline	0.1837	0.1856	0.1946
Change at week 3	0.0085 (0.0331)	-0.0329 (0.0306)	-0.0511 (0.0340)
Vs placebo	..	-0.0414 (-0.1279 to 0.0450), p=0.3429	-0.0596 (-0.1505 to 0.0312), p=0.1950
Vs liraglutide	..	..	-0.0182 (-0.1051 to 0.0686), p=0.6770
Change at week 6	0.0118 (0.0415)	-0.0331 (0.0409)	-0.0667 (0.0444)
Vs placebo	..	-0.0449 (-0.1581 to 0.0683), p=0.4321	-0.0784 (-0.1959 to 0.0390), p=0.1874
Vs liraglutide	..	..	-0.0336 (-0.1501 to 0.0830), p=0.5680
Change from week 3 to week 6 within treatment group	0.0033 (-0.0942 to 0.1007), p=0.9469	-0.0002 (-0.0931 to 0.0927), p=0.9964	-0.0155 (-0.1164 to 0.0853), p=0.7581
**Superior temporal gyrus**			
Baseline	0.1970	0.1814	0.2151
Change at week 3	-0.0117 (0.0331)	-0.0457 (0.0306)	-0.0905 (0.0340)
Vs placebo	..	-0.0339 (-0.1204 to 0.0525), p=0.4370	-0.0788 (-0.1698 to 0.0123), p=0.0889
Vs liraglutide	..	..	-0.0448 (-0.1317 to 0.0421), p=0.3073
Change at week 6	0.0055 (0.0405)	-0.0278 (0.0400)	-0.0081 (0.0434)
Vs placebo	..	-0.0333 (-0.1439 to 0.0772), p=0.5501	-0.0136 (-0.1284 to 0.1012), p=0.8142
Vs liraglutide	..	..	0.0197 (-0.0941 to 0.1336), p=0.7310
Change from week 3 to week 6 within treatment group	0.0172 (-0.0789 to 0.1134), p=0.7223	0.0178 (-0.0738 to 0.1095), p=0.6991	0.0824 (-0.0172 to 0.1820), p=0.1035
**Precentral gyrus**			
Baseline	0.1300	0.1737	0.1708
Change at week 3	-0.0300 (0.0274)	-0.0046 (0.0253)	-0.0736 (0.0281)
Vs placebo	..	0.0254 (-0.0460 to 0.0969), p=0.4800	-0.0435 (-0.1187 to 0.0316), p=0.2517
Vs liraglutide	..	..	-0.0690 (-0.1405 to 0.0026), p=0.0586
Change at week 6	0.0108 (0.0341)	-0.0210 (0.0335)	-0.0483 (0.0364)
Vs placebo	..	-0.0317 (-0.1247 to 0.0612), p=0.4981	-0.0591 (-0.1556 to 0.0375), p=0.2264
Vs liraglutide	..	..	-0.0273 (-0.1228 to 0.0682), p=0.5700
Change from week 3 to week 6 within treatment group	0.0408 (-0.0348 to 0.1164), p=0.2849	-0.0164 (-0.0881 to 0.0554), p=0.6496	0.0253 (-0.0524 to 0.1029), p=0.5164
**Cingulate gyrus**			
Baseline	0.1607	0.1790	0.1947
Change at week 3	-0.0114 (0.0269)	-0.0422 (0.0248)	-0.0765 (0.0275)
Vs placebo	..	-0.0308 (-0.1009 to 0.0394), p=0.3847	-0.0651 (-0.1388 to 0.0087), p=0.0828
Vs liraglutide	..	..	-0.0343 (-0.1046 to 0.0360), p=0.3337
Change at week 6	0.0307 (0.0447)	-0.0255 (0.0440)	-0.0409 (0.0476)
Vs placebo	..	-0.0562 (-0.1792 to 0.0669), p=0.3658	-0.0716 (-0.1993 to 0.0561), p=0.2675

Vs liraglutide	..	..	-0.0154 (-0.1421 to 0.1113), p=0.8091
Change from week 3 to week 6 within treatment group	0.0421 (-0.0632 to 0.1473), p=0.4274	0.0167 (-0.0852 to 0.1185), p=0.7444	0.0356 (-0.0755 to 0.1466), p=0.5235
**Hippocampus**			
Baseline	0.1234	0.1388	0.1505
Change at week 3	0.0336 (0.0223)	-0.0503 (0.0206)	-0.0568 (0.0229)
Vs placebo	..	-0.0839 (-0.1425 to -0.0253), p=0.0056	-0.0904 (-0.1521 to -0.0287), p=0.0046
Vs liraglutide	..	..	-0.0065 (-0.0654 to 0.0524), p=0.8266
Change at week 6	0.0009 (0.0291)	-0.0229 (0.0287)	0.0226 (0.0311)
Vs placebo	..	-0.0238 (-0.1035 to 0.0560), p=0.5541	0.0217 (-0.0612 to 0.1045), p=0.6036
Vs liraglutide	..	..	0.0455 (-0.0367 to 0.1276), p=0.2739
Change from week 3 to week 6 within treatment group	-0.0327 (-0.1081 to 0.0427), p=0.3911	0.0274 (-0.0452 to 0.1001), p=0.4550	0.0794 (-0.0003 to 0.1591), p=0.0509
**Putamen**			
Baseline	0.0780	0.0999	0.1235
Change at week 3	-0.0308 (0.0167)	-0.0496 (0.0154)	-0.0428 (0.0172)
Vs placebo	..	-0.0187 (-0.0624 to 0.0249), p=0.3944	-0.0120 (-0.0581 to 0.0341), p=0.6063
Vs liraglutide	..	..	0.0068 (-0.0370 to 0.0506), p=0.7586
Change at week 6	0.0242 (0.0291)	-0.0190 (0.0287)	0.0085 (0.0311)
Vs placebo	..	-0.0432 (-0.1235 to 0.0372), p=0.2878	-0.0157 (-0.0994 to 0.0679), p=0.7087
Vs liraglutide	..	..	0.0274 (-0.0554 to 0.1103), p=0.5114
Change from week 3 to week 6 within treatment group	0.0550 (-0.0131 to 0.1232), p=0.1116	0.0306 (-0.0354 to 0.0967), p=0.3582	0.0513 (-0.0208 to 0.1233), p=0.1599
**Orbitofrontal cortex**			
Baseline	0.1681	0.1653	0.1793
Change at week 3	0.0117 (0.0304)	-0.0292 (0.0280)	-0.0326 (0.0311)
Vs placebo	..	-0.0410 (-0.1204 to 0.0385), p=0.3074	-0.0444 (-0.1279 to 0.0391), p=0.2932
Vs liraglutide	..	..	-0.0034 (-0.0832 to 0.0764), p=0.9328
Change at week 6	0.0150 (0.0380)	-0.0304 (0.0375)	-0.0579 (0.0406)
Vs placebo	..	-0.0454 (-0.1492 to 0.0584), p=0.3864	-0.0729 (-0.1805 to 0.0347), p=0.1812
Vs liraglutide	..	..	-0.0275 (-0.1344 to 0.0794), p=0.6097
Change from week 3 to week 6 within treatment group	0.0033 (-0.0911 to 0.0976), p=0.9451	-0.0012 (-0.0915 to 0.0892), p=0.9796	-0.0253 (-0.1238 to 0.0732), p=0.6073

Data are mean of absolute values at baseline, least squares mean (standard error) change from baseline, and difference in least squares mean (95% CI) vs placebo and liraglutide. For each scan and each region, the mean of positive voxels was taken within each of the regions separately. No positive voxels (Food > Non-food) were identified for the ventral striatum; therefore, statistical analysis was not done on this region. BOLD=blood oxygenation level dependent; CI=confidence interval; fMRI=functional magnetic resonance imaging; n=number of participants who were randomised and received at least one dose of study treatment.

Table S14. BOLD fMRI brain activation for low-fat/low-carbohydrate/high-protein foods (not highly palatable foods) versus non-food objects

	Placebo (n=39)	Liraglutide (n=38)	Tirzepatide (n=37)
**Insula**			
Baseline	0.1402	0.1647	0.1870
Change at week 3	0.0222 (0.0355)	-0.0085 (0.0329)	0.0076 (0.0366)
Vs placebo	..	-0.0307 (-0.1240 to 0.0626), p=0.5139	-0.0147 (-0.1131 to 0.0838), p=0.7673
Vs liraglutide	..	..	0.0160 (-0.0780 to 0.1101), p=0.7350
Change at week 6	-0.0170 (0.0337)	0.0041 (0.0333)	-0.0310 (0.0364)
Vs placebo	..	0.0212 (-0.0699 to 0.1122), p=0.6449	-0.0140 (-0.1089 to 0.0809), p=0.7700
Vs liraglutide	..	..	-0.0351 (-0.1289 to 0.0586), p=0.4578
Change from week 3 to week 6 within treatment group	-0.0393 (-0.1247 to 0.0461), p=0.3635	0.0126 (-0.0675 to 0.0928), p=0.7548	-0.0385 (-0.1258 to 0.0487), p=0.3816
**Medial frontal gyrus**			
Baseline	0.1691	0.2023	0.2018
Change at week 3	0.0681 (0.0419)	0.0076 (0.0387)	-0.0242 (0.0430)
Vs placebo	..	-0.0605 (-0.1713 to 0.0502), p=0.2800	-0.0923 (-0.2089 to 0.0242), p=0.1189
Vs liraglutide	..	..	-0.0318 (-0.1432 to 0.0796), p=0.5715
Change at week 6	-0.0230 (0.0346)	-0.0097 (0.0341)	-0.0459 (0.0372)
Vs placebo	..	0.0133 (-0.0797 to 0.1062), p=0.7767	-0.0229 (-0.1193 to 0.0736), p=0.6377
Vs liraglutide	..	..	-0.0362 (-0.1318 to 0.0595), p=0.4534
Change from week 3 to week 6 within treatment group	-0.0911 (-0.1922 to 0.0100), p=0.0767	-0.0173 (-0.1122 to 0.0776), p=0.7175	-0.0217 (-0.1257 to 0.0823), p=0.6792
**Superior temporal gyrus**			
Baseline	0.1375	0.1503	0.1705
Change at week 3	0.0694 (0.0493)	0.0441 (0.0456)	0.0153 (0.0508)
Vs placebo	..	-0.0253 (-0.1553 to 0.1047), p=0.6990	-0.0541 (-0.1913 to 0.0832), p=0.4351
Vs liraglutide	..	..	-0.0287 (-0.1599 to 0.1024), p=0.6638
Change at week 6	0.0042 (0.0407)	0.0567 (0.0403)	-0.0103 (0.0441)
Vs placebo	..	0.0525 (-0.0569 to 0.1619), p=0.3417	-0.0146 (-0.1285 to 0.0994), p=0.7996
Vs liraglutide	..	..	-0.0671 (-0.1798 to 0.0457), p=0.2395
Change from week 3 to week 6 within treatment group	-0.0652 (-0.1759 to 0.0455), p=0.2447	0.0127 (-0.0904 to 0.1157), p=0.8075	-0.0257 (-0.1382 to 0.0868), p=0.6503
**Precentral gyrus**			
Baseline	0.1624	0.2047	0.1887
Change at week 3	0.0273 (0.0432)	0.0215 (0.0397)	-0.0356 (0.0442)
Vs placebo	..	-0.0057 (-0.1204 to 0.1090), p=0.9210	-0.0628 (-0.1834 to 0.0578), p=0.3028
Vs liraglutide	..	..	-0.0571 (-0.1725 to 0.0583), p=0.3276
Change at week 6	-0.0562 (0.0293)	-0.0302 (0.0290)	-0.0352 (0.0316)
Vs placebo	..	0.0260 (-0.0526 to 0.1046), p=0.5120	0.0210 (-0.0603 to 0.1023), p=0.6077
Vs liraglutide	..		-0.0050 (-0.0857 to 0.0757), p=0.9028
Change from week 3 to week 6 within treatment group	-0.0835 (-0.1843 to 0.0173), p=0.1033	-0.0518 (-0.1461 to 0.0426), p=0.2780	0.0004 (-0.1036 to 0.1043), p=0.9941
**Cingulate gyrus**			
Baseline	0.1499	0.1856	0.1613
Change at week 3	0.0165 (0.0319)	-0.0015 (0.0296)	-0.0437 (0.0329)
Vs placebo	..	-0.0180 (-0.1017 to 0.0658), p=0.6701	-0.0602 (-0.1481 to 0.0277), p=0.1763
Vs liraglutide	..	..	-0.0422 (-0.1264 to 0.0419), p=0.3208
Change at week 6	-0.0170 (0.0344)	0.0081 (0.0340)	-0.0223 (0.0370)
Vs placebo	..	0.0251 (-0.0685 to 0.1186), p=0.5948	-0.0053 (-0.1023 to 0.0917), p=0.9132

Vs liraglutide	..	..	-0.0304 (-0.1266 to 0.0659), p=0.5311
Change from week 3 to week 6 within treatment group	-0.0335 (-0.1143 to 0.0473), p=0.4122	0.0095 (-0.0666 to 0.0857), p=0.8035	0.0214 (-0.0613 to 0.1040), p=0.6073
**Hippocampus**			
Baseline	0.0982	0.0845	0.1622
Change at week 3	0.0230 (0.0379)	0.0242 (0.0351)	0.0147 (0.0393)
Vs placebo	..	0.0012 (-0.0993 to 0.1017), p=0.9811	-0.0082 (-0.1148 to 0.0983), p=0.8781
Vs liraglutide	..	..	-0.0094 (-0.1117 to 0.0928), p=0.8547
Change at week 6	0.0082 (0.0271)	0.0129 (0.0269)	-0.0355 (0.0297)
Vs placebo	..	0.0046 (-0.0677 to 0.0770), p=0.8986	-0.0437 (-0.1197 to 0.0323), p=0.2560
Vs liraglutide	..	..	-0.0483 (-0.1239 to 0.0273), p=0.2068
Change from week 3 to week 6 within treatment group	-0.0148 (-0.0982 to 0.0687), p=0.7260	-0.0113 (-0.0888 to 0.0662), p=0.7722	-0.0502 (-0.1352 to 0.0348), p=0.2431
**Putamen**			
Baseline	0.0773	0.0904	0.1165
Change at week 3	0.0097 (0.0225)	-0.0162 (0.0205)	0.0085 (0.0228)
Vs placebo	..	-0.0259 (-0.0846 to 0.0329), p=0.3836	-0.0012 (-0.0631 to 0.0608), p=0.9701
Vs liraglutide	..	..	0.0247 (-0.0339 to 0.0832), p=0.4037
Change at week 6	-0.0183 (0.0218)	-0.0111 (0.0215)	-0.0310 (0.0235)
Vs placebo	..	0.0072 (-0.0517 to 0.0660), p=0.8092	-0.0127 (-0.0740 to 0.0485), p=0.6800
Vs liraglutide	..	..	-0.0199 (-0.0805 to 0.0407), p=0.5152
Change from week 3 to week 6 within treatment group	-0.0280 (-0.0841 to 0.0281), p=0.3245	0.0050 (-0.0472 to 0.0573), p=0.8486	-0.0396 (-0.0965 to 0.0174), p=0.1705
**Orbitofrontal cortex**			
Baseline	0.1658	0.1731	0.1854
Change at week 3	0.0545 (0.0354)	-0.0020 (0.0326)	-0.0243 (0.0363)
Vs placebo	..	-0.0565 (-0.1501 to 0.0370), p=0.2324	-0.0788 (-0.1773 to 0.0196), p=0.1150
Vs liraglutide	..	..	-0.0223 (-0.1165 to 0.0719), p=0.6388
Change at week 6	-0.0289 (0.0282)	-0.0104 (0.0279)	-0.0448 (0.0303)
Vs placebo	..	0.0185 (-0.0572 to 0.0942), p=0.6271	-0.0159 (-0.0945 to 0.0627), p=0.6877
Vs liraglutide	..	..	-0.0344 (-0.1125 to 0.0436), p=0.3818
Change from week 3 to week 6 within treatment group	-0.0834 (-0.1691 to 0.0023), p=0.0563	-0.0084 (-0.0889 to 0.0722), p=0.8370	-0.0205 (-0.1089 to 0.0679), p=0.6453

Data are mean of absolute values at baseline, least squares mean (standard error) change from baseline, and difference in least squares mean (95% CI) vs placebo and liraglutide. For each scan and each region, the mean of positive voxels was taken within each of the regions separately. No positive voxels (Food > Non-food) were identified for the ventral striatum; therefore, statistical analysis was not done on this region. BOLD=blood oxygenation level dependent; CI=confidence interval; fMRI=functional magnetic resonance imaging; n=number of participants who were randomised and received at least one dose of study treatment.

Table S15. Sensitivity analysis of energy intake in participants who experienced and did not experience any nausea or vomiting adverse events during the study

	Placebo	Liraglutide	Tirzepatide
Energy intake at lunch, kcal			
**In participants who experienced nausea or vomiting**	n=2	n=11	n=23
Baseline	1021.43	956.19	1015.87
Change at week 3	-21.03 (156.56)	-289.19 (63.34)	-553.20 (51.08)
Vs placebo		-268.16 (-609.61, 73.29), p=0.1186	-532.17 (-860.98, -203.36), p=0.0026
Vs liraglutide			-264.01 (-423.54, -104.48), p=0.0022
Change at week 6	-68.67 (181.02)	-268.97 (76.37)	-702.64 (58.98)
Vs placebo		-200.30 (-598.19, 197.60), p=0.3113	-633.98 (-1016.06, -251.89), p=0.0021
Vs liraglutide			-433.68 (-625.53, -241.82), p<.0001
**In participants who did not experience nausea or vomiting**	n=37	n=27	n=14
Baseline	886.17	1080.95	779.61
Change at week 3	-0.54 (49.63)	-310.84 (59.92)	-443.07 (82.00)
Vs placebo		-310.31 (-454.72, -165.89), p<.0001	-442.53 (-622.08, -262.99), p<0.0001
Vs liraglutide			-132.23 (-324.79, 60.34), p=0.1750
Change at week 6	41.82 (56.22)	-338.77 (66.17)	-533.17 (91.65)
Vs placebo		-380.59 (-543.91, -217.26), p<0.0001	-574.99 (-778.77, -371.21), p<0.0001
Vs liraglutide			-194.40 (-410.52, 21.72), p=0.0771

Data are mean at baseline, and least square mean (standard error) change from baseline. n=number of participants in each subgroup.

Table S16. Post-hoc analysis of macronutrient intake during the ad libitum lunch meal

	Placebo (n=39)	Liraglutide (n=38)	Tirzepatide (n=37)
Fat, kcal			
Baseline	391.93	432.98	408.50
Change at week 3	-0.04 (20.50)	-132.67 (21.11)	-240.32 (22.11)
Vs placebo		-132.62 (-187.77, -77.47), p<0.0001	-240.28 (-297.07, -183.48), p<0.0001
Vs liraglutide			-107.66 (-164.66, -50.65), p=0.0003
Change at week 6	26.62 (23.09)	-133.77 (23.32)	-295.29 (24.54)
Vs placebo		-160.39 (-222.54, -98.23), p<.0001	-321.91 (-385.98, -257.84), p<0.0001
Vs liraglutide			-161.52 (-225.41, -97.64), p<0.0001
Change from week 3 to week 6 within treatment group	26.66 (-2.81, 56.14), p=0.0757	-1.10 (-29.75, 27.55), p=0.9395	-54.96 (-85.02, -24.91), p=0.0005
Carbohydrate, kcal			
Baseline	311.88	382.55	321.26
Change at week 3	-0.57 (19.34)	-104.62 (19.93	-184.88 (20.89
Vs placebo		-104.06 (-156.18, -51.93), p=0.0001	-184.31 (-237.76, -130.86), p<0.0001
Vs liraglutide			-80.25 (-134.24, -26.27), p=0.0040
Change at week 6	6.18 (22.83)	-100.74 (23.04)	-229.36 (24.29)
Vs placebo		-106.92 (-168.70, -45.14), p=0.0009	-235.55 (-299.07, -172.03), p<0.0001
Vs liraglutide			-128.63 (-192.22, -65.04), p=0.0001
Change from week 3 to week 6 within treatment group	6.75 (-21.98, 35.48), p=0.6420	3.89 (-24.02, 31.79), p=0.7829	-44.49 (-73.78, -15.19), p=0.0033
Protein, kcal			
Baseline	190.61	228.50	178.62
Change at week 3	-7.29 (10.55)	-62.10 (12.86)	-105.78 (17.21)
Vs placebo		-54.81 (-85.74, -23.87), p=0.0008	-98.49 (-136.52, -60.46), p<0.0001
Vs liraglutide			-43.68 (-84.41, -2.95), p=0.0360
Change at week 6	-4.67 (12.33)	-79.04 (14.48)	-125.44 (19.80)
Vs placebo		-74.36 (-110.25, -38.47), p<0.0001	-120.77 (-165.27, -76.26), p<0.0001
Vs liraglutide			-46.40 (-93.38, 0.57), p=0.0528
Change from week 3 to week 6 within treatment group	2.62 (-14.05, 19.29), p=0.7546	-19.66 (-45.52, 6.21), p=0.1338	-16.94 (-35.49, 1.62), p=0.0729

Data are mean (standard deviation) at baseline, least squares mean (standard error) change from baseline, difference in least squares mean (95% CI) vs placebo and liraglutide, change from week 3 to week 6 (95% CI). n=number of participants who were randomized to each treatment group.

Table S17. Number of participants who completed each assessment at each time point

	Placebo (n=39)	Liraglutide (n=38)	Tirzepatide (n=37)
	Number of participants who completed assessment		
**Energy Intake**			
Baseline	39	38	37
Week 3	36	36	32
Week 6	34	36	32
**Body weight**			
Baseline	39	38	37
Week 3	38	36	32
Week 6	35	36	32
**Ingestive Behavior Assessments**			
Fasting VAS			
Baseline	33	33	32
Week 3	31	31	27
Week 6	29	31	27
Postprandial VAS			
Baseline	39	38	37
Week 3	36	36	32
Week 6	34	36	32
Food Craving Inventory, Food Craving Questionnaire-State, Power of Food, and Barratt Impulsiveness Scale			
Baseline	39	38	37
Week 3	37	37	32
Week 6	35	36	32
Eating Inventory			
Baseline	39	37	37
Week 3	37	36	32
Week 6	35	35	32
**BOLD fMRI Parameters**			
$Food_{HiPal}$ – all ROIs except putamen			
Baseline	33	34	31
Week 3	25	31	25
Week 6	27	28	24
$Food_{HiPal}$ - putamen			
Baseline	33	34	31
Week 3	25	31	25
Week 6	26	28	24
Individual Food Categories – all ROIs			
Baseline	33	34	31
Week 3	26	31	25
Week 6	27	28	24

n=total number of participants randomized to each treatment group; ROI=region of interest.

References

1. United States Department of Agriculture. United States Department of Agriculture, Agricultural Research Service. 2018. USDA Food and Nutrient Database for Dietary Studies 2015-2016.
2. Flint A, Raben A, Blundell JE, Astrup A. Reproducibility, power and validity of visual analogue scales in assessment of appetite sensations in single test meal studies. *Int J Obes Relat Metab Disord* 2000; **24**(1): 38-48.
3. Womble LG, Wadden TA, Chandler JM, Martin AR. Agreement between weekly vs. daily assessment of appetite. *Appetite* 2003; **40**(2): 131-5.
4. White MA, Whisenhunt BL, Williamson DA, Greenway FL, Netemeyer RG. Development and Validation of the Food-Craving Inventory. *Obes Res* 2002; **10**(2): 107-14.
5. Cepeda-Benito A, Gleaves DH, Williams TL, Erath SA. The development and validation of the state and trait food-cravings questionnaires. *Beh Ther* 2000; **31**(1): 151-73.
6. Moreno S, Rodríguez S, Fernandez MC, Tamez J, Cepeda-Benito A. Clinical validation of the trait and state versions of the Food Craving Questionnaire. *Assessment* 2008; **15**(3): 375-87.
7. Stunkard AJ, Messick S. The three-factor eating questionnaire to measure dietary restraint, disinhibition and hunger. *J Psychosom Res* 1985; **29**(1): 71-83.
8. Cappelleri JC, Bushmakin AG, Gerber RA, Leidy NK, Sexton CC, Karlsson J, Lowe MR. Evaluating the Power of Food Scale in obese subjects and a general sample of individuals: development and measurement properties. *Int J Obes* 2009; **33**(8): 913-22.
9. Lowe MR, Butryn ML, Didie ER, et al. The Power of Food Scale. A new measure of the psychological influence of the food environment. *Appetite* 2009; **53**(1): 114-8.
10. Patton JH, Stanford MS, Barratt ES. Factor structure of the Barratt impulsiveness scale. *J Clin Psychol* 1995; **51**(6): 768-74.
11. Reid RC, Cyders MA, Moghaddam JF, Fong TW. Psychometric properties of the Barratt Impulsiveness Scale in patients with gambling disorders, hypersexuality, and methamphetamine dependence. *Addict Behav* 2014; **39**(11): 1640-5.

Protocol I8F-MC-GPHH(f) Effect of Tirzepatide on Energy Intake and Appetite- and Reward-Related Brain Areas in Overweight/Obese Subjects: A Placebo-Controlled 6-Week Study with Functional MRI

Confidential Information

The information contained in this protocol is confidential and is intended for the use of clinical investigators. It is the property of Eli Lilly and Company or its subsidiaries and should not be copied by or distributed to persons not involved in the clinical investigation of tirzepatide (LY3298176), unless such persons are bound by a confidentiality agreement with Eli Lilly and Company or its subsidiaries.

Note to Regulatory Authorities: This document may contain protected personal data and/or commercially confidential information exempt from public disclosure. Eli Lilly and Company requests consultation regarding release/redaction prior to any public release. In the United States, this document is subject to Freedom of Information Act (FOIA) Exemption 4 and may not be reproduced or otherwise disseminated without the written approval of Eli Lilly and Company or its subsidiaries.

Tirzepatide (LY3298176)

Eli Lilly and Company
Indianapolis, Indiana USA 46285

Clinical Pharmacology Protocol Electronically Signed and Approved by Lilly: 31 January 2020
Amendment (a) Electronically Signed and Approved by Lilly: 03 March 2020
Amendment (b) Electronically Signed and Approved by Lilly: 30 April 2020
Amendment (c) Electronically Signed and Approved by Lilly: 02 June 2020
Amendment (d) Electronically Signed and Approved by Lilly: 11 December 2020
Amendment (e) Electronically Signed and Approved by Lilly: 02 February 2021
Amendment (f) Electronically Signed and Approved by Lilly on approval date provided below.

Approval Date: 08-Oct-2021 GMT

Table of Contents

Effect of Tirzepatide on Energy Intake and Appetite- and Reward-Related Brain Areas in Overweight/Obese Subjects: A Placebo-Controlled 6-Week Study with Functional MRI

Section	Page
Protocol I8F-MC-GPHH(f) Effect of Tirzepatide on Energy Intake and Appetite- and Reward-Related Brain Areas in Overweight/Obese Subjects: A Placebo-Controlled 6-Week Study with Functional MRI	1
Table of Contents	2
1. Protocol Synopsis	8
2. Schedule of Activities	10
3. Introduction	18
3.1. Study Rationale	18
3.2. Background	18
3.3. Benefit/Risk Assessment	20
4. Objectives and Endpoints	22
5. Study Design	24
5.1. Overall Design	24
5.2. Number of Participants	27
5.3. End of Study Definition	27
5.4. Scientific Rationale for Study Design	27
5.5. Justification for Dose	28
6. Study Population	30
6.1. Inclusion Criteria	30
6.2. Exclusion Criteria	33
6.3. Lifestyle and Dietary Requirements	37
6.3.1. Meals and Dietary Restrictions	37
6.3.2. Caffeine, Alcohol, and Tobacco	37
6.3.3. Activity	37
6.4. Screen Failures	37
7. Treatment	38
7.1. Treatment Administered	38
7.1.1. Packaging and Labeling	39
7.2. Method of Treatment Assignment	39
7.2.1. Selection and Timing of Doses	39
7.3. Blinding	40

7.4. Dose Modification....................40
7.4.1. Special Treatment Considerations....................40
7.4.1.1. Management of Subjects with Gastrointestinal Symptoms....................40
7.5. Preparation/Handling/Storage/Accountability....................41
7.6. Treatment Compliance....................41
7.7. Concomitant Therapy....................41
7.8. Treatment after the End of the Study....................42
8. Discontinuation Criteria....................43
8.1. Discontinuation from Study Treatment....................43
8.1.1. Permanent Discontinuation from Study Treatment....................43
8.1.2. Discontinuation of Inadvertently Enrolled Subjects....................44
8.2. Discontinuation from the Study....................44
8.3. Subjects Lost to Follow-up....................44
9. Study Assessments and Procedures....................45
9.1. Efficacy Assessments....................45
9.1.1. Appetite and Eating Behavior Assessments....................45
9.1.2. Neuroimaging Assessments....................46
9.1.3. Hormone and Metabolite Assessments....................47
9.2. Adverse Events....................47
9.2.1. Serious Adverse Events....................48
9.2.1.1. Suspected Unexpected Serious Adverse Reactions....................49
9.2.2. Adverse Events of Special Interest....................49
9.2.2.1. Hypoglycemia....................49
9.2.2.2. Pancreatitis....................50
9.2.2.3. Thyroid Malignancies and C-Cell Hyperplasia....................51
9.2.2.4. Hypersensitivity Events....................52
9.2.2.5. Injection-Site Reactions....................52
9.2.2.6. Hepatobiliary Disorders....................53
9.2.2.7. Severe Gastrointestinal Adverse Events....................53
9.2.2.8. Acute Renal Events....................53
9.2.3. Complaint Handling....................53
9.3. Treatment of Overdose....................53
9.4. Safety....................53
9.4.1. Laboratory Tests....................53
9.4.2. Vital Signs....................54
9.4.3. Body Weight and Waist Circumference....................54
9.4.4. Electrocardiograms....................54
9.4.5. Physical Examinations....................55

9.4.6. Suicidal Ideation Assessment 55
9.4.7. Safety Monitoring 55
9.4.7.1. Hepatic Safety 56
9.5. Pharmacokinetics 58
9.5.1. Bioanalysis 58
9.6. Pharmacodynamics 58
9.6.1. Immunogenicity Assessments 58
9.7. Genetics 59
9.8. Biomarkers 59
9.9. Health Economics 60
9.10. Passive Detection of Eating Activity 60
10. Statistical Considerations and Data Analysis 61
10.1. Sample Size Determination 61
10.2. Populations for Analyses 61
10.2.1. Study Participant Disposition 61
10.2.2. Study Participant Characteristics 61
10.2.3. Treatment Compliance 61
10.3. Statistical Analyses 61
10.3.1. Safety Analyses 62
10.3.1.1. Clinical Evaluation of Safety 62
10.3.1.2. Statistical Evaluation of Safety 62
10.3.2. Pharmacokinetic Analyses 62
10.3.2.1. Pharmacokinetic Parameter Estimation 62
10.3.2.2. Pharmacokinetic Statistical Inference 62
10.3.3. Pharmacodynamic Analyses 62
10.3.3.1. Pharmacodynamic Parameter Estimation 62
10.3.3.2. Pharmacodynamic Statistical Inference 63
10.3.4. Evaluation of Immunogenicity 63
10.3.5. Interim Analyses 64
10.3.6. Exploratory Analysis for Motion Sensor Modeling 64
11. References 65
12. Appendices 70

List of Tables

Table **Page**

Table GPHH.1. Objectives and Endpoints 22
Table GPHH.2. Study Treatments and Dose Escalation 38
Table GPHH.3. Details of Buffet Meal 81

List of Figures

Figure **Page**

Figure GPHH.1. Illustration of study design for Protocol I8F-MC-GPHH........................26

List of Appendices

Appendix		Page
Appendix 1.	Abbreviations and Definitions	71
Appendix 2.	Clinical Laboratory Tests	76
Appendix 3.	Study Governance, Regulatory and Ethical Considerations	77
Appendix 4.	Blood Sampling Summary	80
Appendix 5.	Pharmacodynamic Assessments	81
Appendix 6.	Hepatic Monitoring Tests for Treatment-Emergent Abnormality	89
Appendix 7.	Pancreatic Monitoring	91
Appendix 8.	Recommended Laboratory Testing for Hypersensitivity Events	93
Appendix 9.	Protocol Amendment I8F-MC-GPHH(f) Summary - Effect of Tirzepatide on Energy Intake and Appetite- and Reward-Related Brain Areas in Overweight/Obese Subjects: A Placebo-Controlled 6-Week Study with Functional MRI	95

1. Protocol Synopsis

Title of Study:
Effect of Tirzepatide on Energy Intake and Appetite- and Reward-Related Brain Areas in Overweight/Obese Subjects: A Placebo-Controlled 6-Week Study with Functional MRI

Rationale:
Tirzepatide (LY3298176) is a long-acting, dual agonist that binds to the glucose-dependent insulinotropic polypeptide (GIP) receptor (GIPR) and the glucagon-like peptide-1 (GLP-1) receptor (GLP-1R). The available preclinical and clinical data indicate that simultaneous stimulation of these receptors may enhance insulin secretion, improve insulin sensitivity, and reduce body weight beyond the effect of selective GLP-1R stimulation.
Study I8F-MC-GPHH (GPHH) is a Phase 1 study consisting of a 6-week treatment period, designed to examine the effect of tirzepatide on energy intake, and central reward and appetite circuits compared with placebo to further understand the mechanisms involved in body weight reduction.

Objectives/Endpoints:

Objectives	Endpoints
**Primary**<br>To compare the effect of 5-mg tirzepatide versus placebo, at Week 3 on energy intake in a clinical setting.	• Change from baseline in energy intake (kcal) as assessed by ad libitum food intake test
**Secondary**<br>To compare the effect of 5-mg tirzepatide versus placebo, at Week 3 on:<br>• Parameters of central reward and appetite circuits in the fasting state using BOLD fMRI<br>• Parameters of behavioral appetite assessments	• Change from baseline in BOLD fMRI activation to images of highly palatable food (high fat-high sugar and high fat-high carbohydrate) during the fasting state in the brain reward areas (insula, medial frontal gyrus, superior temporal gyrus, precentral gyrus, cingulate gyrus)<br>• Change from baseline in fasting and postprandial appetite VAS, and fasting FCI, FCQ-S, Eating Inventory, Power of Food Scale questionnaires

Abbreviations: BOLD = blood oxygenation level-dependent; FCI = Food Craving Inventory; FCQ-S = Food Craving Questionnaire-State; fMRI = functional magnetic resonance imaging; VAS = visual analog scale.

Summary of Study Design:
Study I8F-MC-GPHH is a Phase 1, multicenter, randomized, partially blinded, placebo-controlled, parallel arm study, with a positive control, liraglutide, in overweight/obese subjects. The primary objective of this study is to compare the effect of tirzepatide and placebo on energy intake, as assessed by ad libitum food intake test, after 3 weeks of treatment at a therapeutic dose of 5 mg (Week 3). Secondary objectives will compare these treatment groups for parameters of central reward and appetite circuits at Week 3. Additional exploratory objectives will assess the effect of 10-mg tirzepatide versus placebo on energy intake and parameters of central reward and appetite at Week 6.

Further exploratory analysis will assess the effect of tirzepatide versus an approved GLP-1 receptor agonist, liraglutide on energy intake and parameters of central reward and appetite signaling at Weeks 3 and/or 6.

Liraglutide will also be used as a positive control for the neuroimaging assessments of appetite- and reward-related brain areas.

Tirzepatide maintenance dose levels of 5, 10, and 15 mg are currently being evaluated in the Phase 3 program in T2DM and chronic weight management indication; both 5 and 10 mg doses are expected to result in weight loss. Tirzepatide 10 mg and liraglutide 3 mg will be attained via step-wise dose escalation to reduce the risk of gastrointestinal adverse events. Eligibility for this study will be assessed at screening (Visit 1) and confirmed at the beginning of the lead-in period (Visit 2).

Treatment Arms and Planned Duration for an Individual Subject:
The study will consist of the following periods: approximately 5-week screening period; 5-day lead-in period; 6-week treatment period, and a 4-week safety follow-up period. Subjects will be randomized in a 1:1:1 ratio to placebo, tirzepatide, or liraglutide. Investigators and subjects will be blinded to tirzepatide and placebo treatment; however, liraglutide treatment will be open-label, therefore the study is considered partially blinded. The randomization will be stratified by baseline BMI (27 to <30 kg/m^2, 30 to <35 kg/m^2, and 35 to 50 kg/m^2).

Tirzepatide once-weekly (QW) dosing will start at 5 mg for 3 weeks followed by a dose escalation to 10 mg for 3 weeks. Subjects will return to the Clinical Research Unit (CRU) each week for the QW administration of tirzepatide or placebo.

Liraglutide once-daily (QD) dosing will start at 0.6 mg for 1 week followed by weekly step-wise dose escalations to 1.2, 1.8, and 2.4 mg, until a 3 mg QD dose is reached. The 3-mg dose of liraglutide will be maintained for 10 days. Subjects will self-administer liraglutide at home in the evening.

Number of Subjects:
Up to 111 subjects (37 subjects per treatment arm) are planned to be randomized so that approximately 93 subjects (31 subjects per treatment arm) complete the study, assuming 15% discontinuation rate.

Statistical Analysis:
Safety analyses will be conducted for all enrolled subjects, whether or not they completed all protocol requirements. Safety parameters that will be assessed include safety lab parameters (including tirzepatide anti-drug antibodies), vital signs, treatment-emergent adverse events (TEAEs; including TEAEs of special interest), and serious adverse events (SAEs). The parameters will be listed and summarized using standard descriptive statistics.

Pharmacodynamic (PD) analyses will be conducted on data from all subjects who receive at least 1 dose of the investigational product and have evaluable data. The primary PD parameter for analysis is energy (kcal) intake, which will be analyzed using an analysis of covariance (ANCOVA) to compare the effect of tirzepatide versus placebo at Week 3 with terms, treatment, baseline BMI stratum, and baseline energy (kcal) intake as a covariate in the model. The response variable in the model will be the change from baseline to Week 3 in energy (kcal) intake, as assessed by ad libitum food intake test. The primary analysis will show least squares means of energy (kcal) intake by treatment and the treatment difference of tirzepatide versus placebo as well as standard error and 95% confidence interval. All tests will be done at the 2-sided 0.05 α level, unless otherwise specified.

Additional exploratory analyses of the data may be conducted as deemed appropriate.

2. Schedule of Activities

Study Schedule Protocol I8F-MC-GPHH: Screening and Lead-in

Procedure	Screening	Lead-in					Comments
**Week of Treatment**	**-5 to -2**	**-1**					
**Visit**	**1**	**2**	**NA**	**NA**	**NA**	**3**	
**Study Day**	**-35 to -5**	**-4**	**-3**	**-2**	**-1**	**1 (predose)**	
**Screening Procedures**							
Informed consent	X						Informed consent will be performed before any other screening procedures.
Lifestyle interview	X						Performed to ensure subjects understand what they will be doing and if they are capable of meeting study demands.
Medical history, menstrual cycle assessment	X						Menstrual cycle assessment performed in females only.
Drug and alcohol screen	X						Procedures may be repeated throughout the study as deemed necessary by the investigator.
Physical examination/medical assessment	X	X				X	Full physical examination at screening. After screening, medical assessment is performed to include medical review and targeted examination, as appropriate.
Height, weight, and BMI	X	X				X	Height and BMI at screening only.
Waist circumference	X					X	
Vital signs (BP/PR/body temperature)	X	X				X	See Section 9.4.2 for details. Vital sign measurements whose nominal times are not listed in the schedule should be taken before PK samples scheduled on the same day.
ECGs (single for safety)	X					X	See Section 9.4.3 for details. Electrocardiograms must be recorded before collecting any blood samples using equipment available at the study site.
Confirm inclusion/exclusion criteria	X	X					
C-SSRS (baseline/ screening form) + SHSF + SHFF	X						The SHFF is required only if triggered by the SHSF, per instructions in the form.
PHQ-9	X						
**Clinical Procedures**							

Procedure	Screening	Lead-in					Comments
**Week of Treatment**	**-5 to -2**	**-1**					
**Visit**	**1**	**2**	**NA**	**NA**	**NA**	**3**	
**Study Day**	**-35 to -5**	**-4**	**-3**	**-2**	**-1**	**1 (predose)**	
Outpatient visit at CRU	X	X				X	Lead-in outpatient visit can occur anytime between Day -4 and Day -1.
Training on signs/symptoms of hypoglycemia and self-treatment		X					Training can be completed anytime during the lead-in period prior to dosing.
Training on self-administration of the subcutaneous liraglutide injection		X					Training can be completed anytime during the lead-in period prior to dosing.
Concomitant medication review	X	X				X	Review of concomitant medication and adverse event can occur anytime between Day -4 and Day -1.
Adverse event review	X	X				X	
Randomization						X	
**Laboratory Tests**							
Safety laboratory tests	X	X					See Appendix 2 for details. Subjects will fast for at least 12 hours before all blood samples are collected.
Pregnancy test	X	X				X	See Appendix 2 for details. Performed and results confirmed prior to MRI.
Hormones and metabolites for appetite and metabolism regulation in the fasting and postprandial states		X					Plasma or serum samples to be taken to measure ghrelin, glucagon, glucose, and triglycerides (fasting), insulin (fasting and postprandial), and PYY, active GIP, active GLP-1, pancreatic polypeptide, amylin, and leptin (postprandial). Postprandial samples will be taken within approximately 60 minutes of completing a test lunch meal.
**Appetite and Eating Behavior**							See Appendix 5 for details.

Procedure	Screening	Lead-in					Comments
Week of Treatment	-5 to -2	-1					
Visit	1	2	NA	NA	NA	3	
Study Day	-35 to -5	-4	-3	-2	-1	1 (predose)	
Ad libitum food intake test at lunch (clinic-based), with appetite VAS		X					Appetite VAS will be completed before and after the lunch test meal. During Lead-in period, ad libitum food intake test at lunch can be done in between Day -4 and Day -1.
FCI, FCQ-S, Eating Inventory, BIS, and Power of Food Scale questionnaires		X					During Lead-in period, questionnaires can be done in between Day -4 and Day -1 in a fasted state shortly after arriving to the study site.
Retrospective appetite VAS		X					
**Neuroimaging**							See Appendix 5 for details.
BOLD fMRI		X					During Lead-in period, fMRI can be done in between Day -4 and Day -1 in a fasted state.
**Diagnostics**							
PK sample						X	PK sample not required for subjects randomized to liraglutide treatment.
Immunogenicity						X	
Pharmacogenetic sample						X	
Nonpharmacogenetic stored samples						X	

Abbreviations: BIS = Barratt Impulsiveness Scale; BMI = body mass index; BOLD = blood oxygenation level dependent; BP = blood pressure; CRU = clinical research unit; C-SSRS = Columbia-Suicide Severity Rating Scale; ECG = electrocardiogram; FCI = Food Craving Inventory; FCQ-S = Food Craving Questionnaire-State; fMRI = functional magnetic resonance imaging; GIP = glucose-dependent insulinotropic polypeptide; GLP-1 = glucagon-like peptide 1; MRI = magnetic resonance imaging; PD = pharmacodynamics; PHQ-9 = Patient Health Questionnaire-9; PK = pharmacokinetics; PR = pulse rate; PYY = peptide YY; SHFF = Self-Harm Follow-Up Form; SHSF = Self-Harm Supplementary Form; VAS = visual analog scale.

Study Schedule Protocol I8F-MC-GPHH: Treatment Period

Procedure	Treatment									
**Week of Treatment**	**1**	**2**	**3**			**4**	**5**	**6**		
**Visit**	**3**	**4**	**5**	**6**	**7**	**8**	**9**	**10**	**11**	**NA**
**Study Day**	**1 [a]**	**8 ± 1**	**15 ± 1**	**16 ± 1**	**17 to 21[g]**	**22 ± 1**	**29 ± 1**	**36 ± 1**	**37 ± 1**	**38-39 ± 1**
**Clinical Procedures**										
Outpatient visit at CRU	X	X	X	X		X	X	X	X	
Weight		P	P			P	P	P		
Waist circumference			P					P		
Vital signs (BP/PR/body temperature)	P	P	P	P		P	P	P	P	
ECGs (single safety)										
Concomitant medication review	X	X	X	X	X	X	X	X	X	
Adverse event review	X	X	X	X	X	X	X	X	X	
**Dose Administration**										
Tirzepatide/placebo [b]	X	X	X			X	X	X		
Liraglutide [c]	Liraglutide will be self-administered every evening from Day 1 through Day 38									
**Laboratory tests**										
Safety laboratory tests								X		
Pregnancy test[h]			X					X		
Hormones and metabolites for appetite and metabolism regulation in the fasting and postprandial states [d]				X					X	
**Appetite and Eating Behavior**										
Ad libitum food intake test at lunch (clinic-based), with appetite VAS [e]				X					X	
FCI, FCQ-S, Eating Inventory, BIS, and Power of Food Scale questionnaires				X					X	
Retrospective appetite VAS				X					X	
**Neuroimaging**										
BOLD fMRI				X					X	
**Diagnostics**										
PK sample [f]			P			P		P		

Procedure	Treatment									
Week of Treatment	1	2	3			4	5	6		
Visit	3	4	5	6	7	8	9	10	11	NA
Study Day	1 [a]	8 ± 1	15 ± 1	16 ± 1	17 to 21[g]	22 ± 1	29 ± 1	36 ± 1	37 ± 1	38-39 ± 1
Immunogenicity			P					P		
Nonpharmacogenetic stored samples			P					P		

Abbreviations: BIS = Barratt Impulsiveness Scale; BMI = body mass index; BOLD = blood oxygenation level-dependent; BP = blood pressure; CRU = clinical research unit; ECG = electrocardiogram; FCI = Food Craving Inventory; FCQ-S = Food Craving Questionnaire-State; fMRI = functional magnetic resonance imaging; GIP = glucose-dependent insulinotropic polypeptide; GLP-1 = glucagon-like peptide 1; MRI = magnetic resonance imaging; NA = not applicable; P = predose; PD = pharmacodynamics; PK = pharmacokinetics; PR = pulse rate; PYY = peptide YY; VAS = visual analog scale.

a Predose assessments on Day 1 are shown in the Screening/Lead-in Schedule of Activities.

b Tirzepatide or placebo will be administered in the CRU only by CRU staff, the morning after an overnight fast of at least 12 hours.

c Liraglutide will be self-administered by the subject in the evenings while at home.

d Plasma or serum samples to be taken to measure ghrelin, glucagon, glucose, and triglycerides (fasting), insulin (fasting and postprandial), and PYY, active GIP, active GLP-1, pancreatic polypeptide, amylin, and leptin (postprandial). Postprandial samples will be taken within approximately 60 minutes of completing a test lunch meal.

e Appetite VAS will be completed before and after the test meal.

f PK sample not required to be collected for subjects randomized to liraglutide treatment.

g Adverse event and concomitant medication review may be remotely assessed any time between Days 17 and 21 if deemed necessary by the investigator.

h Pregnancy test will be conducted prior to fMRI.

Study Schedule Protocol I8F-MC-GPHH: Follow-up Period

Procedure	Follow-up			Early Termination	Comments
**Weeks Post-Final Dose**	**3**		**4**	**NA**	
**Visit**	**12**	**NA**	**13**	**NA**	
**Study Day** (Subjects Randomized to Tirzepatide/Placebo)	**59 ± 1**	**60 - 63 ± 1**	**64 ± 1**	**NA**	
**Study Day** (Subjects Randomized to Liraglutide)	**61 ± 1**	**62 - 65 ± 1**	**66 ± 1**	**NA**	
**Clinical Procedures**					
Outpatient visit at CRU	X		X	X	
At-home assessment		X			
Weight and waist circumference			X	X	
Vital signs (BP/PR/body temperature)			X	X	
ECGs (single safety)			X	X	
Concomitant medication review	X		X	X	
Adverse event review	X		X	X	
Physical examination			X	X	
**Laboratory tests**					
Safety laboratory tests			X	X	See Appendix 2 for details. Subjects will fast for at least 12 hours before all blood samples are collected.
Pregnancy test			X	X	
**Appetite and Eating Behavior**					
Training on use of RFPM and SmartIntake® app training	X				
Ad libitum food intake test at lunch (clinic-based)	X		X		See Appendix 5 for details. Only test meal (lunch) is required to be completed on Visit 13. Subjects must be wearing a wrist-wearable device during the test meals.
Ad libitum food intake assessment (free-living)		X			See Appendix 5 for details. Measured with the RFPM and SmartIntake app. Subjects will complete this assessment at home.

Procedure	Follow-up			Early Termination	Comments
**Weeks Post-Final Dose**	**3**		**4**	**NA**	
**Visit**	**12**	**NA**	**13**	**NA**	
**Study Day**<br>(Subjects Randomized to Tirzepatide/Placebo)	**59 ± 1**	**60 - 63 ± 1**	**64 ± 1**	**NA**	
**Study Day**<br>(Subjects Randomized to Liraglutide)	**61 ± 1**	**62 - 65 ± 1**	**66 ± 1**	**NA**	
**Other Efficacy/PD Assessments**					
Provide subjects with wrist-wearable device and training on its use	X				See Section 9.10 for details.
Passive eating activity data collection via wrist-wearable device	X	X	X		See Section 9.10 for details.
Return wrist-wearable device to CRU staff			X	X	
**Diagnostics**					
PK sample			X	X	PK sample not required for subjects randomized to liraglutide treatment.
Immunogenicity			X	X	

Abbreviations: BP = blood pressure; CRU = clinical research unit; ECG = electrocardiogram; NA = not applicable; PD = pharmacodynamics; PK = pharmacokinetics; PR = pulse rate; RFPM = Remote Food Photography Method©.

3. Introduction

3.1. Study Rationale

Tirzepatide (LY3298176) is a long-acting, dual agonist that binds to the glucose-dependent insulinotropic polypeptide (GIP) receptor (GIPR) and the glucagon-like peptide-1 (GLP-1) receptor (GLP-1R). The available preclinical and clinical data indicate that simultaneous stimulation of these receptors may enhance insulin secretion, improve insulin sensitivity, and reduce body weight beyond the effect of selective GLP-1R stimulation (Frias et al. 2018; Coskun et al. 2018). Study I8F-MC-GPHH (GPHH) is a Phase 1 study consisting of a 6-week treatment period, designed to examine the effect of tirzepatide on energy intake, and central reward and appetite circuits compared with placebo to further understand the mechanism involved in body weight reduction.

3.2. Background

Glucagon-like peptide 1 is synthesized and secreted from enteroendocrine L cells in the small and large intestine and is a well-characterized incretin hormone that potentiates insulin and reduces glucagon secretion in a glucose-dependent manner after meal ingestion. GLP-1 exerts its insulinotropic action through distinct G-protein-coupled receptors highly expressed on islet β cells and in some non-islet cells. For example, GLP-1Rs are expressed throughout the brain, in regions that control glucose homeostasis, gut motility, energy intake, aversive signaling, and cardiovascular function (Campbell and Drucker 2013; Farr et al. 2016). Currently, there are approved GLP-1 receptor agonists (GLP-1RAs) for the treatment of diabetes and obesity.

Glucose-dependent insulinotropic polypeptide is synthesized and secreted by enteroendocrine K cells in the proximal intestine. The GIPR is widely expressed in islets, gut, adipose tissue, and brain. GIP secretion is primarily regulated by nutrients, especially fats. GIP is responsible for the majority of the insulinotropic incretin effect in humans (Calanna et al. 2013; Nauck and Meier 2016; Nauck and Meier 2019). GIP has important additional functions that are distinct from GLP-1. GIP promotes glucagon secretion at low blood glucose levels to augment endogenous glucose production. It stimulates lipolysis and inhibits insulin-induced lipogenesis in human adipocytes. Although GIP signaling on body weight regulation is neutral, co-administration of individual GIP and GLP-1 selective agonists as well as unimolecular co-agonists has shown profound weight-lowering benefits that exceed that of either agent alone (Finan et al. 2016). Despite the observed effects of GIP, currently, no pharmaceutical agents that are based on its structure and function have been developed for treatment of metabolic conditions.

Tirzepatide is a 39-amino-acid synthetic peptide with dual agonistic activity at both the GIP and GLP-1 receptors. Its structure is based on the GIP sequence and includes a C20 fatty di-acid moiety (Coskun et al. 2018). It is administered once weekly (QW) by subcutaneous (SC) injection.

Tirzepatide is being developed as a therapy to improve glycemic control in adults with type 2 diabetes mellitus (T2DM), as an adjunct to diet and exercise. Tirzepatide is also being investigated for chronic weight management and nonalcoholic steatohepatitis.

In a Phase 1 study (Coskun et al. 2018) that included single- and multiple-ascending dose (SAD, MAD) parts, tirzepatide has been administered as a single SC dose up to 8 mg in healthy subjects. In the MAD part, higher doses up to 10 mg were attained in healthy subjects via dose escalation. In the same study, doses up to 15 mg were achieved in patients with T2DM via dose escalation. In this study, gastrointestinal (GI) adverse events (AEs) (nausea, vomiting, diarrhea, abdominal distension) and decreased appetite were the most frequently reported events by both healthy subjects and patients with T2DM and were dose related. Most AEs were mild in severity, a few were moderate, and none were reported as severe. During the SAD study, the high incidence of GI AEs, notably vomiting, were considered to be dose limiting at the 8-mg dose; therefore, the 5-mg dose was considered the maximum tolerated dose (MTD) for a single dose. A dose-dependent increase in heart rate was detected in both healthy subjects and patients with T2DM who received tirzepatide, similar to what was observed with selective GLP-1RAs. A few subjects experienced transient elevations in lipase and/or amylase levels, but these laboratory observations were not associated with any relevant clinical outcomes.

Once-weekly doses of 1, 5, 10, and 15 mg have been further investigated in a Phase 2 study (Frias et al. 2018). Additionally, dose levels of 12 and 15 mg, attained using alternate dose-escalation schemes, were investigated in a 12-week Phase 2 study (Frias et al. 2020). Doses above 5 mg of tirzepatide were attained via stepwise dose escalation. Results from the 2 Phase 2 studies demonstrated that tirzepatide at doses between 5 and 15 mg provided clinically meaningful efficacy in lowering both glucose and body weight. Gastrointestinal-related AEs (nausea, diarrhea, vomiting) were the most frequently reported AEs in Phase 2 studies. The majority of the treatment-emergent adverse events (TEAEs) were mild or moderate in severity. Treatment with tirzepatide was associated with treatment-emergent anti-drug antibodies (TE-ADAs) in 40.7% in the 26-week Phase 2 study. However, the presence of anti-drug antibodies (ADAs) appeared to have no effect on the pharmacokinetics (PK), safety, or efficacy of tirzepatide. There were no other clinically relevant safety observations in the Phase 1 and 2 studies.

Tirzepatide terminal half-life was estimated to be approximately 5 days, thus supporting a QW dosing regimen, with maximum observed drug concentration occurring 24 to 72 hours post-dose.

Glucagon-like peptide-1 receptor agonists have demonstrated efficacy in body weight reduction. Liraglutide (Saxenda®) is the first agent from this class that has been approved for the treatment of chronic weight management, at a dose of 3 mg/day. The following dose escalation to a 3-mg dose is recommended to mitigate the risk of GI side effects; 1 week at 0.6 mg once-daily (QD), 1 week at 1.2 mg QD, 1 week at 1.8 mg QD, 1 week at 2.4 mg QD, followed by 3.0 mg QD onwards (Saxenda package insert 2018). Commonly reported adverse reactions following liraglutide administration include GI disorders (e.g., nausea, diarrhea, constipation, vomiting), metabolism and nutrition disorders (hypoglycemia in patients with T2DM, decreased appetite), and nervous system disorders (e.g., headache) (Saxenda package insert 2018).

Studies that investigated the mechanism of action of liraglutide have shown that liraglutide induces weight loss by reducing appetite and energy intake, with no effect on energy expenditure (van Can et al. 2014; Blundell et al. 2017).

In Phase 1 and Phase 2 development, treatment with tirzepatide has shown clinically meaningful effect on body weight regulation by suppressing appetite and reducing energy intake, but no data are available regarding the effects of tirzepatide on brain activity related to different food cues. In the literature, it is well demonstrated that GLP-1 affects central reward and appetite circuits of the brain in healthy lean and obese subjects as well as subjects with T2DM (van Bloemendaal et al. 2014; Farr et al. 2016; Hayes and Schmidt 2016).

Compared with healthy lean subjects, obese patients with T2DM and non-diabetic obese subjects showed increased brain responses to visual food cues in appetite- and reward-related brain regions (insula and amygdala). Intravenous treatment with a GLP-1 receptor agonist, exenatide, reduced these brain responses which correlated with reduction in food intake (van Bloemendaal et al. 2014). This effect was mediated by the GLP-1 receptor activation, as the exenatide-induced effects were inhibited by infusion of the GLP-1 receptor antagonist, exendin 9-39 (van Bloemendaal et al. 2014). Similarly, treatment with liraglutide has been shown to reduce brain activation in response to highly desirable food images in the parietal cortex (Farr et al. 2016) as well as the insula and putamen (ten Kulve et al. 2016). Recent literature has proposed the reducing effect of liraglutide on brain activation may be short-term; at 5 weeks following dose escalation to 3 mg, treatment with liraglutide did not show differential activations in response to food cues, compared to placebo (Farr et al. 2019). These results warrant further investigations of how dual GIPR/GLP-1R agonism affects brain activation in response to visual food cues.

Study GPHH is designed to assess the effect of tirzepatide versus placebo on energy intake, assessed by ad libitum food intake test, as well as central reward and appetite circuits using functional magnetic resonance imaging (fMRI) under fasting condition. The effect of liraglutide versus tirzepatide and placebo on energy intake will be assessed as an exploratory objective. In addition, liraglutide treatment will be used in the current study as a positive control for neuroimaging assessments. The study is expected to provide important, new information on feeding behavior associated with a dual GIP/GLP-1 receptor agonist treatment.

3.3. Benefit/Risk Assessment

The most common safety issue with administration of tirzepatide was related to frequent reporting of decreased appetite and GI side effects, most commonly nausea, vomiting, and diarrhea. These GI events were generally mild in severity, with few moderate, and no severe events reported in Phase 1 studies. Tirzepatide triggered generation of ADAs in a subset of patients in Phase 2 trials. The presence of tirzepatide ADA did not affect PK or pharmacodynamic (PD) parameters and was not associated with increased incidence of local or systemic hypersensitivity AEs in Phase 1 and Phase 2 studies up to 26 weeks of exposure. Based on these results, tirzepatide ADA represents a low risk in exposed patients, but more data is needed for definitive conclusion on the frequency and severity of possible side effects. No other clinically relevant safety concerns were identified in the dose range up to 15 mg, the highest dose investigated in Phase 1 and Phase 2 studies in T2DM patients, administered QW up to 26 weeks. Findings to date indicate that the safety profile for a dual GIP/GLP-1 receptor agonist is similar to the safety profile of the selective GLP-1RAs. Potential risks, such as GI effects, acute

pancreatitis, increases in heart rate, and hypoglycemic events are consistent with the risks associated with currently available GLP-1RAs, including liraglutide, the positive control in this trial. To mitigate the risk of GI adverse events, the target tirzepatide 10 mg and liraglutide 3 mg doses will be attained via step-wise dose escalation. Based on the results of Phase 2 study (Frias et al. 2018), tirzepatide doses of 5, 10, and 15 mg are expected to lead to meaningful body weight reduction. While 5-mg tirzepatide can safely be administered as a first dose (i.e., the MTD in Study GPGA; Coskun et al. 2018), doses of 10 and 15 mg can be attained only via step wise dose escalation to minimize the potential for GI adverse events.

Details on the management of the potential risks are provided in Section 7.4.1.1 (Management of Subjects with Gastrointestinal Symptoms) and Section 9.2.2 (Adverse Events of Special Interest). The monitoring plan for all AEs of special interest is included in this protocol, as well as treatment measures.

Since Study GPHH will enroll subjects without diabetes, no self-monitoring of plasma glucose (PG) is planned. Consistent with the mechanism of action of incretins, which are non-secretagogues, severe hypoglycemia has not been reported in Phase 1 or Phase 2 trials that included healthy subjects or patients with T2DM. However, to mitigate the risk, the subjects in this study will be trained on signs and symptoms of hypoglycemia and self-treatment measures. Section 9.2.2.1 (Hypoglycemia) describes definitions and criteria when diagnosing and categorizing an episode considered to be related to hypoglycemia, and provides detailed information concerning the management of hypoglycemia.

There are several possible AEs associated with the planned procedures in the study. A magnetic resonance imaging (MRI) scanner will be used to assess brain response patterns to food and control images (see Appendix 5 for more details). Some level of claustrophobia or discomfort may be experienced from staying in the fMRI scanner.

Although there is a possibility of weight loss associated with tirzepatide treatment, subjects in Study GPHH are not expected to have direct health-related benefits due to the short duration of study treatment. The potential risks described above, which are also similarly reported in liraglutide, are considered clinically detectable, transient, manageable, and associated with low long-term health risks. The routine clinical safety monitoring procedures planned in this study is considered sufficient to mitigate the possible risks.

More information about the known and expected benefits, risks, serious adverse events (SAEs) and reasonably anticipated AEs of tirzepatide are to be found in the Investigator's Brochure (IB). Information on AEs expected to be related to the investigational product (IP) can be found in Section 6 (Summary of Data, Development Core Safety Information) of the IB. Information on SAEs that are expected in the study population independent of drug exposure will be assessed by the sponsor in aggregate, periodically during the course of the study, and can be found in Section 5 (Effects in Humans) of the IB.

More detailed information about the known and expected benefits and risks of liraglutide (Saxenda) may be found in the Package Insert or US Prescribing Information (USPI).

4. Objectives and Endpoints

Table GPHH.1 shows the objectives and endpoints of the study.

Table GPHH.1. Objectives and Endpoints

Objectives	Endpoints
**Primary**<br>To compare the effect of 5-mg tirzepatide versus placebo, at Week 3, on energy intake in a clinical setting.	• Change from baseline in energy intake (kcal) as assessed by ad libitum food intake test
**Secondary**<br>To compare the effect of 5-mg tirzepatide versus placebo, at Week 3 on:<br>• Parameters of central reward and appetite circuits in the fasting state using BOLD fMRI<br>• Parameters of behavioral appetite assessments	• Change from baseline in BOLD fMRI activation to images of highly palatable food (high fat-high sugar and high fat-high carbohydrate) during the fasting state in the brain reward areas (insula, medial frontal gyrus, superior temporal gyrus, precentral gyrus, cingulate gyrus)<br>• Change from baseline in fasting and postprandial appetite VAS, and fasting FCI, FCQ-S, Eating Inventory, Power of Food Scale questionnaires
**Exploratory**<br>To compare the effect of 5- or 10-mg tirzepatide versus placebo at Week 3 and/or Week 6 on:<br>• Parameters of central reward and appetite circuits in the fasting state using BOLD fMRI (Week 6/10 mg)<br>• Parameters of behavioral appetite assessments (Week 6/10 mg)<br>• Energy intake in a clinical setting (Week 6/10 mg)	• Change from baseline in BOLD fMRI activation to images of highly palatable food (high fat-high sugar and high fat-high carbohydrate) during the fasting state in the brain reward areas (insula, medial frontal gyrus, superior temporal gyrus, precentral gyrus, cingulate gyrus)<br>• Change from baseline in fasting and postprandial appetite VAS, and fasting FCI, FCQ-S, Eating Inventory, Power of Food Scale questionnaires<br>• Change from baseline in energy intake (kcal) as assessed by ad libitum food intake test
• Parameters of central reward and appetite circuits in the fasting state using BOLD fMRI (Week 3/5 mg and Week 6/10 mg)<br>• Impulsivity (Week 3/5 mg and Week 6/10 mg)	• Change from baseline in BOLD fMRI activation to images of highly palatable food (high fat-high sugar and high fat-high carbohydrate) during fasting state in the hippocampus, putamen, orbitofrontal cortex, and ventral striatum brain regions<br>• Change from baseline in BIS questionnaire

• Appetite and metabolism regulating hormones and metabolites (Week 3/5 mg and Week 6/10 mg)	• Change from baseline in ghrelin, glucagon, glucose, and triglycerides (fasting), insulin (fasting and postprandial), and amylin, GIP, GLP-1, leptin, pancreatic polypeptide, and PYY (postprandial)
To compare the effect of 5- and 10-mg tirzepatide versus liraglutide and liraglutide versus placebo at Week 3 and/or Week 6 on:<br>• Energy intake in a clinical setting (Week 3/5 mg and Week 6/10 mg)<br>• Parameters of behavioral appetite assessments (Week 3/5 mg and Week 6/10 mg)<br>• Parameters of central reward and appetite circuits in the fasting state using BOLD fMRI (Week 3/5 mg and Week 6/10 mg)<br>• Appetite and metabolism regulating hormones and metabolites (Week 3/5 mg and Week 6/10 mg)	• Changes from baseline in energy intake (kcal) as assessed by ad libitum food intake test<br>• Change from baseline in fasting and postprandial appetite VAS, and fasting FCI, FCQ-S, Eating Inventory, Power of Food Scale questionnaires<br>• Change from baseline in BOLD fMRI activation to images of highly palatable food (high fat-high sugar and high fat-high carbohydrate) during the fasting state in the brain reward areas (insula, medial frontal gyrus, superior temporal gyrus, precentral gyrus, cingulate gyrus, hippocampus, putamen, orbitofrontal cortex, and ventral striatum)<br>• Change from baseline in ghrelin, glucagon, glucose, and triglycerides (fasting), insulin (fasting and postprandial), and amylin, GIP, GLP-1, leptin, pancreatic polypeptide, and PYY (postprandial)
To assess the safety and tolerability of tirzepatide.	• Adverse events<br>• Safety laboratory parameters<br>• Frequency of treatment-emergent anti-tirzepatide antibodies

Abbreviations: BIS = Barratt Impulsiveness Scale; BOLD = blood oxygenation level-dependent; FCI = Food Craving Inventory; FCQ-S = Food Craving Questionnaire-State; fMRI = functional magnetic resonance imaging; GIP = glucose-dependent insulinotropic polypeptide; GLP-1 = glucagon-like peptide 1; PYY = peptide YY; VAS = visual analog scale.

5. Study Design

5.1. Overall Design

This is a Phase 1, multicenter, randomized, partially blinded, placebo-controlled, parallel-arm study, with a positive control, liraglutide in overweight/obese subjects. The primary objective of this study is to compare the effect of tirzepatide and placebo on energy intake, as assessed by ad libitum food intake test, after 3 weeks of treatment at a therapeutic dose of 5 mg (Week 3). Secondary objectives will compare these treatment groups for parameters of central reward and appetite circuits at Week 3. Additional exploratory objectives will assess the effect of 10-mg tirzepatide versus placebo on energy intake and parameters of central reward and appetite at Week 6.

Further exploratory analysis will assess the effect of tirzepatide versus liraglutide, and liraglutide versus placebo on energy intake and parameters of central reward and appetite signaling at Weeks 3 and/or 6.

Liraglutide will also be used as a positive control for the neuroimaging assessments of appetite- and reward-related brain areas.

See Section 5.4 for details on study design rationale.

The study will consist of 4 periods (see Section 2, Schedule of Activities):

- approximately 5-week screening period (Visit 1; Days -35 to -5)
- a 5-day lead-in period, including pre-dose assessments on Day 1 (Visits 2 to 3)
    - 3 clinical research unit [CRU] outpatient visits
- a 6-week treatment period (Visits 3 to 11)
    - 9 CRU outpatient visits
- a 4-week safety follow-up period (Visits 12 and 13)
    - 2 CRU outpatient visits

The subject will sign the informed consent form (ICF) before any study procedures are performed. Eligibility will be assessed at screening (Visit 1) and confirmed during the lead-in period (Visit 2), when safety laboratory results will be available.

Subjects will perform baseline assessments in the CRU prior to randomization, according to the Schedule of Activities (Section 2). After the completion of all baseline procedures, subjects will be randomized in a 1:1:1 ratio to tirzepatide, placebo, or liraglutide. Investigators and subjects will be blinded to tirzepatide and placebo treatment; however, liraglutide treatment will be open-label, therefore the study is considered partially blinded (see Section 5.4). The randomization will be stratified by baseline BMI (27 to <30 kg/m^2, 30 to <35 kg/m^2, and 35 to 50 kg/m^2).

Tirzepatide QW dosing will start at 5 mg for 3 weeks followed by a dose escalation to 10 mg for 3 weeks. Subjects will return to the CRU each week for the QW administration of tirzepatide or placebo.

Liraglutide QD dosing will start at 0.6 mg for 1 week followed by weekly step-wise dose escalations to 1.2, 1.8, and 2.4 mg, until a 3 mg QD dose is reached. The 3-mg dose of liraglutide will be maintained for 10 days. Subjects will self-administer liraglutide at home in the evening.

Section 2 (Schedule of Activities) details all planned visits and procedures for this study. After completion of the 6-week treatment period, subjects will attend 2 follow-up outpatient visits at the CRU; approximately 3 and 4 weeks after the last dose of study drug. The final follow-up visit will assess subject safety, subjects will return the wearable device, and subsequently complete each subject’s participation.

All subjects will receive study diaries where they will be instructed to record relevant clinical information, when outside of the CRU (e.g., AEs, concomitant medications, details of drug dosing, hypoglycaemia symptoms).

Figure GPHH.1 illustrates the study design. Study governance considerations are described in detail in Appendix 3.

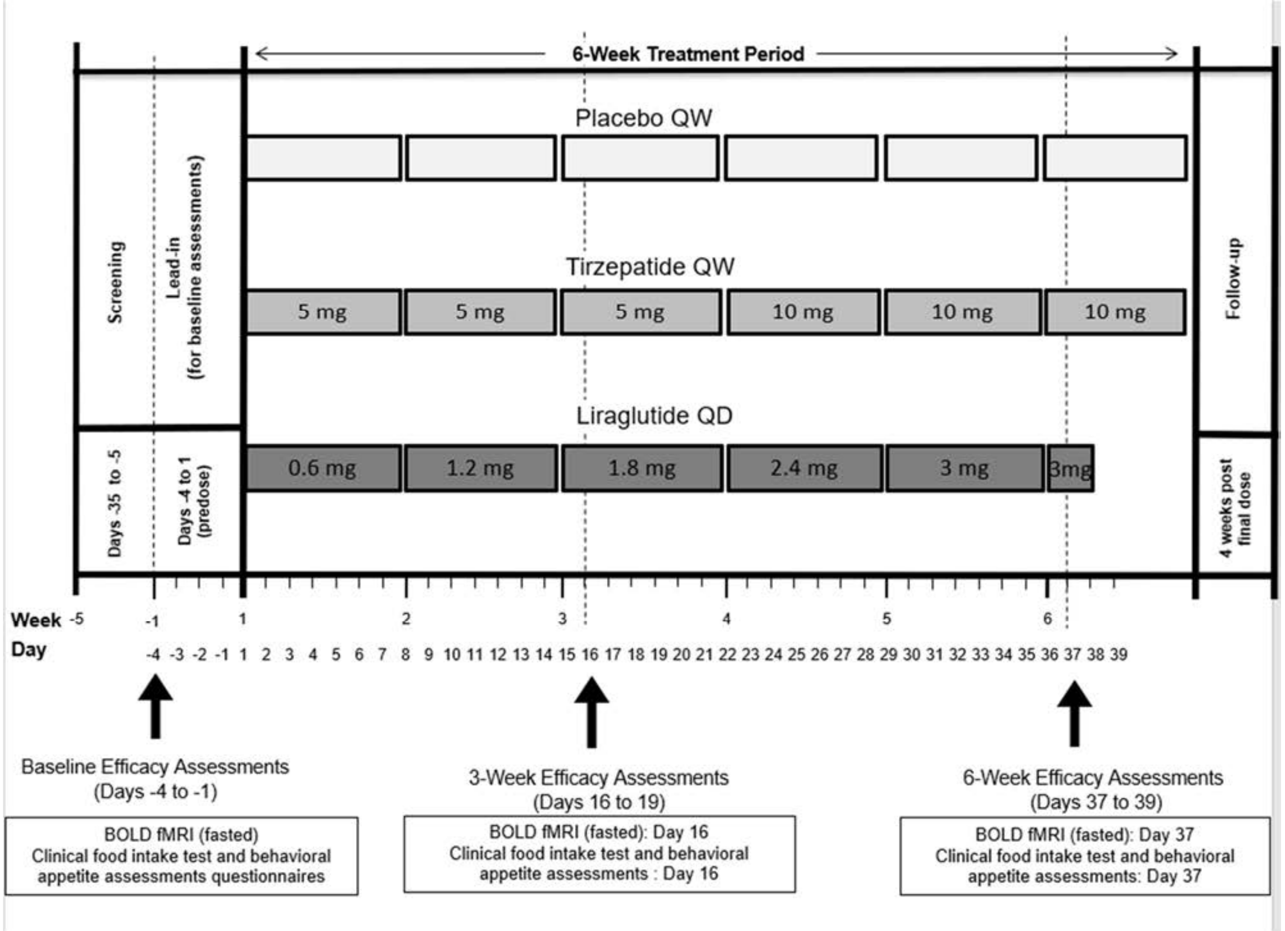

Abbreviations: BOLD = blood oxygenation level-dependent; fMRI = functional MRI; QD = once daily; QW = once weekly.

Figure GPHH.1. Illustration of study design for Protocol I8F-MC-GPHH.

5.2. Number of Participants

Up to 111 subjects (37 subjects per treatment arm) may be enrolled to maximize the probability that approximately 93 subjects (31 subjects per treatment arm) complete the study. For purposes of this study, a subject completes the study when all scheduled procedures shown in the Schedule of Activities have been finished.

5.3. End of Study Definition

End of the study is the date of the last visit or last scheduled procedure shown in the Schedule of Activities (Section 2) for the last subject.

5.4. Scientific Rationale for Study Design

Tirzepatide treatment is currently being investigated for chronic weight management, therefore overweight/obese subjects only will be enrolled as the target population. In addition, overweight/obese subjects are expected to be more susceptible to a potential weight reduction effect by tirzepatide and liraglutide as compared to lean subjects. Previous literature has confirmed obese subjects (body mass index [BMI] ≥30 kg/m^2) had increased brain responses to food cues in central reward- and appetite-related brain regions, compared to lean subjects (BMI <25 kg/m^2) (van Bloemendaal et al. 2014). To avoid the confounding effect of changes in glucose regulation on energy intake, the study will enroll nondiabetic subjects only.

Study GPHH will employ a parallel-group design to avoid the confounding effects of the study treatments expected if a cross-over design is used.

The primary study measure of the ad libitum food intake test was chosen as the most reproducible and clinically relevant measure of energy intake. The secondary study measures, such as functional neuroimaging (blood oxygenation level-dependent [BOLD] MRI), parameters of appetite, and eating attitudes and behaviors, are expected to provide important additional data. All study measures and methods of assessment are well validated and fully established in the medical literature as the parameters and methods relevant for objectives of Study GPHH.

Placebo has been chosen as the primary control treatment to assess whether any observed effects are treatment-related or simply reflect the study conditions. Liraglutide (Saxenda) has been chosen as a neuroimaging positive control for GLP-1 pharmacology. Liraglutide (1.8 mg) has been shown to reduce brain activation in response to highly desirable food images in the parietal cortex (Farr et al. 2016) as well as the insula and putamen (ten Kulve et al. 2016). The dose justification for tirzepatide and liraglutide is provided in Section 5.5 (Justification of Dose).

The timing of endpoint measurements (Week 3 and Week 6) was determined based on the effect of GLP-1 on brain centers and the time needed to escalate doses. In addition, tirzepatide doses of 5-mg and 10-mg have shown to result in clinically meaningful body weight reduction (Frias et al. 2018). Previous literature confirms liraglutide effects on central nervous system activation were observed following 10 to 17 days of treatment, where liraglutide dose was escalated weekly to 1.8 mg (Farr et al. 2016; ten Kulve et al. 2016), supporting efficacy assessments at Week 3 in the current study. Given the possibility that the effect of incretins on brain activation is short

term (Farr et al. 2019), the Week 3 efficacy assessment of 5-mg tirzepatide versus placebo will be the primary objective. The influence of higher tirzepatide doses, such as 10 mg, on brain activity is not yet understood, therefore efficacy assessments in the current study will occur at Week 6 (as exploratory endpoints), due to the need for dose escalation to attain a 10-mg tirzepatide dose.

In addition, efficacy assessments at Week 6 was chosen to observe the effect of tirzepatide and liraglutide after the dose-escalation regimen to reach 10 mg and 3 mg, respectively.

The dosing of tirzepatide will be double blinded and placebo-controlled in order to avoid bias in the collection and evaluation of data during its conduct. Liraglutide dosing will be open label as blinding is not possible due to the QD dosing regimen. In order to achieve the recommended liraglutide dose-escalation scheme, liraglutide doses must be self-administered daily in comparison to the weekly dosing of tirzepatide/placebo in the CRU.

The rationale for the sample size is provided in Section 10.1 (Sample Size Determination).

5.5. Justification for Dose

Tirzepatide maintenance dose levels of 5, 10, and 15 mg are currently being evaluated in the Phase 3 program for T2DM and chronic weight management indication. Results from a Phase 2 study (Frias et al. 2020 have shown that in addition to the highest dose level of 15 mg, tirzepatide dose levels of 5 and 10 mg also result in clinically meaningful body weight reduction.

Weekly doses of tirzepatide ranging from 0.25 to 8 mg have been studied in the single-ascending dose study, with MTD achieved at 5 mg when administered as a single dose. Dose levels of 10 and 15 mg were tolerated when administered via step-wise dose escalation (Frias et al. 2018; Coskun et al. 2018). Tirzepatide 15 mg is predicted to maintain an exposure multiple of 1.6 to 2.4 to the no-observed-adverse-effect level doses in 6-month monkey and rat toxicology studies, respectively. Additional information can be found in the IB.

Tirzepatide doses of 5 mg and 10 mg administered SC QW have been selected for investigation in this study based on current toxicology, and clinical data. Doses of liraglutide QD for 17 days (dose escalated to 1.8-mg) reduced brain activation in response to highly desirable food images (Farr et al. 2016; ten Kulve et al. 2016). However, following 5-weeks of dosing with liraglutide (dose escalated to 3-mg), differential brain activity in response to food cues, compared to placebo, was not observed (Farr et al. 2019). Therefore, doses of 5- and 10-mg tirzepatide have been chosen in the current study as they can be attained in a reasonable duration, while providing an opportunity to observe brain activity signals up to 6 weeks.

The tirzepatide treatment period will consist of a step-wise dose escalation where dosing will start at 5 mg QW for the first 3 weeks followed by escalation to 10 mg QW at Week 4, followed by repeating administration of the 10-mg dose at Week 5 and Week 6. Dosing will be initiated at 5 mg and administered for 3 weeks before escalating to a 10-mg dose. This stepwise increment was selected based on cumulative understanding of safety and GI tolerability from Phase 1 and 2 studies. It is expected to minimize GI tolerability concerns in this overweight/obese population

by permitting adequate time for development of tolerance to GI events. Near steady-state exposures are expected to be attained within 3 weeks of dosing at the 10-mg dose level.

The final liraglutide dose of 3 mg is selected because it is the highest dose approved for the treatment of chronic weight management. Additionally, the liraglutide dose of 1.8 mg at Week 3 should result in sufficient exposure to act as the positive control in the current study (Farr et al. 2016). The dose-escalation schedule of liraglutide to be used in the current trial is consistent with the recommended dosing of marketed liraglutide (Saxenda package insert 2018) and has been chosen to mitigate the risk of GI AEs.

6. Study Population

Eligibility of subjects for the study will be based on the results of screening medical history, physical examination, vital signs, clinical laboratory tests, and electrocardiograms (ECGs).

The nature of any conditions present at the time of the physical examination and any preexisting conditions will be documented.

Screening may occur within approximately 4 weeks prior to lead-in. Eligible subjects will enter lead-in (Visit 2) approximately 4 days prior to randomization. Subjects who are not enrolled within 6 weeks of screening may be subjected to additional medical assessment and/or clinical measurements to confirm their eligibility.

Prospective approval of protocol deviations to recruitment and enrollment criteria, also known as protocol waivers or exemptions, are not permitted.

6.1. Inclusion Criteria

Subjects are eligible for inclusion in the study only if they meet all the following criteria at screening:

Subject Characteristics

[1] are overweight/obese (BMI of 27 kg/m^2 to 50 kg/m^2, inclusive, and can complete MRI scans (see Exclusion Criterion 8), and nondiabetic male or female subjects, as determined by medical history, physical examination, and laboratory assessments at screening;

[2] must be weight stable (no weight change of >4 kg) in the last 1 month;

[3] have safety laboratory test results within normal reference range or with abnormalities deemed clinically insignificant by the investigator;

[4] have venous access sufficient to allow for blood sampling as per the protocol;

[5] male or female subjects between the ages of 18 to 65 years, inclusive;

[5a] male subjects:

- men, regardless of their fertility status, with nonpregnant woman of childbearing potential (WOCBP) partners must agree to either remain abstinent (if this is their preferred and usual lifestyle) or use condoms plus 1 additional highly effective (less than 1% failure rate) method of contraception (such as combination oral contraceptives, implanted contraceptives, or intrauterine device) or effective method of contraception, (such as diaphragms with spermicide or cervical sponge) for the duration of the study and for 5 half-lives of study drug plus 90 days, corresponding to 4 months after the last injection .

- men and their partners may choose to use a double-barrier method of contraception. (Barrier protection methods without concomitant use of a spermicide are not an effective or acceptable method of contraception. Thus, each barrier method must include use of a spermicide. It should be noted however that the use of male and female condoms as a double-barrier method is not considered acceptable due to the high failure rate when these barrier methods are combined.)
- periodic abstinence (e.g., calendar, ovulation, symptothermal, postovulation methods), declaration of abstinence just for the duration of a study, and withdrawal are not acceptable methods of contraception.

- men with pregnant partners should use condoms during intercourse for the duration of the study and until the end of estimated relevant potential exposure in WOCBP, which corresponds to 4 months following last injection.
- men must agree to refrain from sperm donation for the duration of the study and until their plasma concentrations are below the level that could result in a relevant potential exposure to a possible fetus, predicted to be 4 months following last injection.
- men who are in exclusively same-sex relationships (as their preferred and usual lifestyle) are not required to use contraception.

[5b] female subjects:

- women of childbearing potential who are abstinent (if this is complete abstinence, as their preferred and usual lifestyle) or in a same-sex relationship (as part of their preferred and usual lifestyle) must agree to either remain abstinent or stay in a same-sex relationship without sexual relationships with males. Periodic abstinence (e.g., calendar, ovulation, symptothermal, post-ovulation methods), declaration of abstinence just for the duration of a study, and withdrawal are not acceptable methods of contraception.
- otherwise, WOCBP participating must agree to use effective contraception, where at least 1 form is highly effective (less than 1% failure rate), for the entirety of the study. Contraception must continue following completion of IP administration for 30 days.
    - women of childbearing potential participating must test negative for pregnancy prior to initiation of treatment as indicated by a negative serum pregnancy test at the screening visit followed by a negative urine pregnancy test within 24 hours prior to exposure
    - two forms of effective contraception, where at least 1 form is highly effective (less than 1% failure rate, such as combination

oral contraceptives, implanted contraceptives or intrauterine devices) will be used. Effective contraception (such as male or female condoms with spermicide, diaphragms with spermicide or cervical sponges) may be used as the second therapy. Barrier protection methods without concomitant use of a spermicide are not a reliable or acceptable method. Thus, each barrier method must include use of a spermicide (that is, condom with spermicide, diaphragm with spermicide, female condom with spermicide). It should be noted that the use of male and female condoms as a double barrier method is not considered acceptable due to the high failure rate when these methods are combined.

    - Not be breastfeeding

- women who are not of childbearing potential may participate and include those who are:
    - infertile due to surgical sterilization (hysterectomy, bilateral oophorectomy, or tubal ligation), congenital anomaly such as mullerian agenesis, or
    - postmenopausal – defined as either
        i. a woman at least 40 years of age with an intact uterus, not on hormone therapy, who has cessation of menses for at least 1 year without an alternative medical cause, AND a follicle-stimulating hormone (FSH) ≥40 mIU/mL; women in this category must test negative in pregnancy test prior to study entry
        ii. a woman 55 years or older not on hormone therapy, who has had at least 12 months of spontaneous amenorrhea; or
        iii. a woman 55 years or older with a diagnosis of menopause prior to starting hormone replacement therapy.

Informed Consent

[6] are reliable and willing to make themselves available for the duration of the study and are willing to follow study procedures;

[7] have given written informed consent approved by Lilly and the institutional review board (IRB) governing the site.

6.2. Exclusion Criteria

Subjects will be excluded from study enrollment if they meet any of the following criteria at screening:

Medical Conditions - General

[8] have a maximum body circumference >219 cm (i.e., at the widest point of the body) or a body weight >225 kg, in order to not exceed the body size limitations imposed by the MRI scanner machinery;

[9] have a history or current cardiovascular (e.g., myocardial infarction, congestive heart failure, cerebrovascular accident, venous thromboembolism), respiratory, hepatic, renal, GI, endocrine, hematological (including history of thrombocytopenia), or neurological disorders capable of significantly altering the absorption, metabolism, or elimination of drugs; or constituting a risk when taking the IP; or may interfere with the interpretation of data;

[10] have obesity induced by other endocrinologic disorders (e.g., Cushing syndrome) or diagnosed monogenetic or syndromic forms of obesity (e.g., melanocortin 4 receptor deficiency or Prader-Willi syndrome)

[11] have acute or chronic pancreatitis or a history of acute idiopathic pancreatitis; or have other GI disorders (e.g., relevant esophageal reflux or gall bladder disease) that could be aggravated by GLP-1 analogs;

- Subjects who have had cholecystolithiasis (removal of gall stones) and/or cholecystectomy (removal of gall bladder) in the past, with no long-term complications, are eligible for participation;

[12] have a known clinically significant gastric emptying abnormality (e.g., severe diabetic gastroparesis or gastric outlet obstruction) or chronically take drugs that directly affect GI motility.

[13] have or plan to have endoscopic and/or device-based therapy for obesity or have had device removal within the last 6 months (for example, mucosal ablation, gastric artery embolization, intragastric balloon and duodenal-jejunal bypass sleeve)

[14] have a prior or planned surgical treatment for obesity (excluding liposuction or abdominoplasty if performed >1 year prior to screening)

[15] have an estimated glomerular filtration rate (eGFR) <60 mL/min/1.73 m^2, calculated by Chronic Kidney Disease-Epidemiology at screening.

[16] have an intention to enter into a weight loss program during the study;

[17] have a personal or family history of medullary thyroid carcinoma (MTC), have multiple endocrine neoplasia syndrome type 2 (MEN 2), or calcitonin ≥20 pg/mL at screening;

[18] are diagnosed with any form of diabetes prior to entry or have a hemoglobin A1c (HbA1c) value at screening of ≥6.5%;

[19] have findings in the 12-lead ECG at screening that, in the opinion of the investigator, may increase the risks of potentially clinically relevant worsening associated with participation in the study;

[20] have blood pressure of ≥160/90 mmHg and/or pulse rate of <45 or >100 bpm (supine) at screening with or without stable doses [at least 1 month prior to screening] of anti-hypertension medications;

[21] have an active or untreated malignancy or have been in remission from a clinically significant malignancy (other than basal or squamous cell skin cancer, in situ carcinomas of the cervix, or in situ prostate cancer) for <5 years prior to screening;

[22] have evidence of human immunodeficiency virus (HIV) and/or positive HIV antibodies at screening;

[23] have evidence of hepatitis B or positive hepatitis B surface antigen and/or evidence of hepatitis C virus (HCV) or hepatitis C antibody at screening;

- subjects with a previous diagnosis of HCV who have been treated with antiviral therapy and achieved a sustained virological response may be eligible for inclusion in the study, provided they have no detectable HCV RNA on the screening HCV polymerase chain reaction test. A sustained virological response is defined as an undetectable HCV RNA level 24 weeks after completion of a full, documented course of an approved antiviral therapy for HCV.
- subjects who have spontaneously cleared HCV infection, defined as (1): a positive HCV antibody test and (2): a negative HCV RNA test, with no history of anti-HCV treatment, may be eligible for inclusion in the study, provided they have no detectable HCV RNA on screening for this study.

[24] have serum aspartate aminotransferase (AST) or alanine aminotransferase (ALT) >2.0× the upper limit of normal (ULN), total bilirubin (TBL) >1.5× ULN (except for subjects with Gilbert's syndrome, which can be enrolled with TBL <2.0× ULN), or alkaline phosphatase (ALP) >1.5× ULN;

- subjects with non-alcoholic fatty liver disease are eligible for participation;

[25] have had a blood donation of 450 mL or more in the last 3 months or any blood donation within the last month prior to screening;

[26] have a history of drug or alcohol abuse within the last 12 months prior to screening; or a positive drug or alcohol screen; and/or smoke >10 cigarettes per day or the equivalent;

[27] have a history of marijuana use within 3 months of enrollment and unwillingness to abstain from marijuana use during the trial. Subjects should also refrain use of cannabidiol oil for the duration of the study.

[28] have had a blood transfusion or severe blood loss within the last 3 months, or have a hemoglobin value <11 g/dL (males) or <10 g/dL (females),

[29] have an average weekly alcohol intake that exceeds 21 units per week (males) and 14 units per week (females) (1 unit = 12 oz or 360 mL of beer; 5 oz or 150 mL of wine; 1.5 oz or 45 mL of distilled spirits), and/or are unwilling to stop alcohol consumption 12 hours before each CRU outpatient visit;

[30] have any lifetime history of a suicide attempt;

[31] Have a Patient Health Questionnaire-9 (PHQ-9) score of 15 or more at screening;

[32] On the Columbia-Suicide Severity Rating Scale (C-SSRS) at screening:

- a "yes" answer to either Question 4 (Active Suicidal Ideation with Some Intent to Act, Without Specific Plan) or
- a "yes" answer to Question 5 (Active Suicidal Ideation with Specific Plan and Intent) on the "Suicidal Ideation" portion of the C-SSRS, or
- a "yes" answer to any of the suicide-related behaviors (Actual Attempt, Interrupted Attempt, Aborted Attempt, Preparatory Act or Behavior) on the "Suicidal Behavior" portion of the C-SSRS, and
- the ideation or behavior occurred within the past month

[33] have a history of atopy or clinically significant multiple or severe drug allergies, or severe post-treatment hypersensitivity reactions (including, but not limited to, erythema multiforme major, linear immunoglobulin A dermatosis, toxic epidermal necrolysis, or exfoliative dermatitis).

Prior/Concomitant Therapy - General

[34] have current or history of (within 6 months prior to screening or between screening and randomization) treatment with medications that may cause significant weight gain, including but not limited to: tricyclic antidepressants, atypical antipsychotic mood stabilizers for example:
Tofranil® (imipramine), Elavil® (amitriptyline), Remeron® (mirtazapine), Paxil®/Seroxat® (paroxetine), Nardil® (phenelzine), Largactil® (chlorpromazine), Mellaril® (thioridazine), Clozaril®/FazaClo®/Versacloz® (clozapine), Zyprexa® (olanzapine), Depakote®/Convulex® (valproic acid) and its derivatives, and/or Lithobid® (lithium)

Note: selective serotonin reuptake inhibitors other than paroxetine are permitted.

Use of other medications not listed above, may result in subject exclusion at the discretion of the investigator in consultation with the Sponsor.

[35] have taken within 6 months prior to screening or between screening and randomization, medications (prescribed or over-the-counter) or alternative remedies intended to promote weight loss. Examples include, but are not limited to:
Saxenda® (liraglutide 3.0 mg), Xenical®/Alli® (orlistat), Meridia® (sibutramine), Acutrium® (phenylpropanolamine), Sanorex® (mazindol), Adipex® (phentermine), BELVIQ® (lorcaserin), Qsymia® (phentermine/topiramate combination), and/or Contrave® (naltrexone/bupropion).

Note: Use of metformin or any other glucose-lowering medication, whether prescribed for polycystic ovary syndrome or diabetes prevention is not permitted.

Use of other medications not listed above, may result in subject exclusion at the discretion of the investigator in consultation with the Sponsor.

[36] have received chronic (lasting >14 consecutive days) systemic glucocorticoid therapy (excluding topical, intra-articular, and inhaled preparations) within 1 month before screening, or between screening and randomization;

[37] have been treated with any glucose-lowering agent during the last 3 months prior to screening or between screening and randomization;

[38] have received treatment with a drug that has not received regulatory approval for any indication within 3 months or 5 half-lives (whichever is longer) of screening.

Prior/Concurrent Clinical Trial Experience

[39] are persons who have previously completed or withdrawn from this study;

[40] have known allergies to tirzepatide or GLP-1 RAs;

[41] have previous exposure to tirzepatide or exposure to GLP-1 RA in the previous 6 months prior to screening and between screening and randomization;

[42] are currently enrolled in a clinical study involving an IP or any other type of medical research judged not to be scientifically or medically compatible with this study.

Other Exclusions

[43] female subjects who are breastfeeding;

[44] suffer from claustrophobia sufficiently severe that it may preclude completion of MRI scanning;

[45] have contraindications such as cardiac pacemakers, aneurysm clips, or other metallic implants considered unsafe for MRI scanning;

[46] are investigative site personnel directly affiliated with this study and their immediate families. Immediate family is defined as a spouse, parent, child, or sibling, whether biological or legally adopted;

[47] are Eli Lilly and Company employees;

[48] are deemed unsuitable by the investigator for any other reason.

6.3. Lifestyle and Dietary Requirements

Throughout the study, subjects may undergo medical assessments and review of compliance with requirements before continuing in the study.

6.3.1. Meals and Dietary Restrictions

Subjects shall fast for at least 12 hours overnight prior to each outpatient visit where fasting samples are drawn. Subjects will fast for at least 12 hours prior to tirzepatide/placebo dosing, after which subjects will receive a meal. Liraglutide will be dosed without regard to timing of meals. Water can be consumed freely.

Subjects will only consume lunch test meal provided by the CRU during outpatient visits. Further details regarding dietary requirements for the planned assessments in the CRU at baseline, Week 3, and Week 6 of the treatment period, and 3 and 4 weeks post final dose of the follow-up period are described in Appendix 5.

6.3.2. Caffeine, Alcohol, and Tobacco

Regular caffeine drinkers are required to maintain their usual amount of caffeine intake throughout the study including on CRU outpatient visit days.

No alcohol will be allowed at least 12 hours before each CRU outpatient visit. Between CRU outpatient visits, weekly alcohol should not exceed 21 units per week for males and 14 units per week for females (a unit is defined in Section 6.2 Exclusion Criteria).

Subjects will not be allowed to use tobacco products in the CRU.

6.3.3. Activity

Subjects will be advised to maintain their regular levels of physical activity/exercise during the study. No intense physical activity will be allowed for at least 48 hours before each CRU outpatient visit. When certain study procedures are in progress at the CRU, subjects may be required to remain recumbent or sitting.

6.4. Screen Failures

Screening tests such as clinical laboratory tests and vital signs/ECGs may be repeated at the discretion of the investigator. Individuals who do not meet the criteria for participation in this study (screen failure) may be re-screened up to 1 time. The interval between re-screenings should be at least 2 weeks. Each time re-screening is performed, the individual must sign a new ICF and will be assigned a new site screening number. Subject numbers/enrollment numbers are assigned on the morning of Day 1.

7. Treatment

7.1. Treatment Administered

Subjects will receive tirzepatide or placebo administered SC QW for 6 doses or liraglutide administered SC QD for 38 days. Table GPHH.2 shows the treatment regimens.

Table GPHH.2. Study Treatments and Dose Escalation

	Dosing Schemes					
**Treatment**	**Week 1**	**Week 2**	**Week 3**	**Week 4**	**Week 5**	**Week 6**
Tirzepatide	1 × 5-mg PFS (Day 1)	1 × 5-mg PFS (Day 8)	1 × 5-mg PFS (Day 15)	1 × 10-mg PFS (Day 22)	1 × 10-mg PFS (Day 29)	1 × 10-mg PFS (Day 36)
Placebo	1 × PFS (Day 1)	1 × PFS (Day 8)	1 × PFS (Day 15)	1 × PFS (Day 22)	1 × PFS (Day 29)	1 × PFS (Day 36)
Liraglutide	0.6 mg QD (Days 1-7)	1.2 mg QD (Days 8-14)	1.8 mg QD (Days 15-21)	2.4 mg QD (Days 22-28)	3 mg QD (Days 29-35)	3 mg QD (Days 36-38)

Abbreviations: PFS = prefilled syringe; QD = once daily.

Tirzepatide or placebo will be administered QW at the CRU in a blinded fashion.

Liraglutide will be administered QD in an open-label manner and will be self-administered in the evening by the subject at home. Subjects will receive adequate training for liraglutide self-administration from site personnel.

All injections will be administered into the SC tissue of the abdominal wall. Injection sites will be alternated weekly for tirzepatide/placebo and daily for liraglutide, between 4 sites (right and left lower quadrants and right and left upper quadrants) of the abdominal wall.

Dosing will occur at approximately the same time of day in all groups and on the same day for the QW groups. The actual time of dosing will be recorded in the subject's electronic case report form (eCRF).

Whenever possible, IP administration at the CRU should be carried out by the same personnel. Additional details are provided in Section 7.3 (Blinding). The personnel who administer study drugs will be unblinded to liraglutide, but they will remain blinded to subjects receiving tirzepatide or placebo.

The investigator or designee is responsible for:

- explaining the correct use of the IP to the site personnel
- verifying that instructions are followed properly
- maintaining accurate records of IP dispensing and collection
- and returning all unused medication to Lilly or its designee at the end of the study

Note: In some cases, sites may destroy the material if, during the investigative site selection, the evaluator has verified and documented that the site has appropriate facilities and written procedures to dispose of clinical materials.

The site will be instructed to discard used medications according to local regulations.

7.1.1. Packaging and Labeling

Tirzepatide and matching placebo will be supplied by Lilly. Tirzepatide will be provided as prefilled syringes (PFS) containing 0.5 mL solution and provided in individual cartons to be dispensed. Placebo will be provided as matching 0.5-mL PFS.

Liraglutide will be the positive control and the commercially available formulation (Saxenda®) will also be supplied by Lilly.

The IP will be labeled according to the country's regulatory requirements.

7.2. Method of Treatment Assignment

Subjects who meet all criteria for enrollment will be randomized in a 1:1:1 ratio to receive tirzepatide, liraglutide, or placebo. Assignment to treatment will be determined by a randomization table with treatment codes. The randomization will be stratified by baseline BMI (27 to <30 kg/m^2, 30 to <35 kg/m^2, and 35 to 50 kg/m^2).

7.2.1. Selection and Timing of Doses

The doses will be administered QW (tirzepatide or placebo) or QD (liraglutide) according to the randomization schedule, on the same day of the week (QW doses) and at approximately the same time of the day. The actual time of all dose administrations will be recorded in the subject's eCRF.

Tirzepatide and placebo will be administered in the morning of each dosing day following an overnight fast of at least 12 hours. Liraglutide will be administered in the evening of each dosing day without regard to timing of meals.

A visit window (±1 day) may be used for dosing of tirzepatide or placebo only when the subject is unable to visit the CRU on the scheduled day. If a subject is not able to receive his/her tirzepatide or placebo dose on the scheduled day (±1 day), the dose should be administered as soon as possible and at least 72 hours prior to the next scheduled dose. If the remaining time to the next scheduled dose is less than 72 hours, the dose will not be administered and will be considered a missed dose.

Investigator site staff will take reasonable steps to educate and inform study subjects randomized to liraglutide to be compliant to their treatment (e.g., written instructions, reminder calls, verbal reinforcement during clinic visits). If a subject misses their daily dose of liraglutide, the subject will be instructed to administer the missed dose as soon as they remember, then administer the next daily dose as usual on the following day. The subject will be instructed not to administer an extra dose or increase their dose on the following day to make up for the missed dose. If a subject misses their daily dose of liraglutide for 2 days or more, they will be instructed to contact staff at the CRU.

7.3. Blinding

The sponsor, investigators, and subjects will be blinded to tirzepatide and placebo treatment assignments; however, liraglutide will be administered in an open-label manner. Therefore, the study will be considered partially blinded.

Blinding between tirzepatide and placebo will be maintained throughout the conduct of the study as described in the separate Blinding and Unblinding Plan. To preserve the blinding of tirzepatide and placebo allocation, all study site personnel, except those who dispense and administer tirzepatide and placebo, will be blinded to treatment allocation. Tirzepatide, placebo, and liraglutide will be dispensed by the site pharmacy in accordance to the randomization table. The site staff will take the necessary steps to ensure that subjects will remain blinded to tirzepatide and placebo administration.

If a subject's study treatment assignment is unblinded (tirzepatide or placebo only), the subject must be discontinued from the study, unless the investigator obtains specific approval from a Lilly clinical pharmacologist (CP) or clinical research physician (CRP) for the subject to continue in the study. During the study, emergency unblinding should occur only by accessing the subject's emergency code.

In case of an emergency, the investigator has the sole responsibility for determining if unblinding of a subject's treatment assignment (tirzepatide or placebo only) is warranted for medical management of the event. The subject's safety must always be the first consideration in making such a determination. If the investigator decides that unblinding is warranted, it is the responsibility of the investigator to promptly document the decision and rationale and notify Lilly as soon as possible.

Upon completion of the study, all codes must be returned to Lilly or its designee.

7.4. Dose Modification

The subject should follow the planned dosing regimen. In the case of poor tolerability during the study, dosing may need to be terminated following the guidance provided in Section 8.1.1 (Permanent Discontinuation from Study Treatment).

Deviations from the planned tirzepatide dose escalation are generally not permitted; however, at the discretion of the investigator, subjects may dose escalate to 10 mg tirzepatide 1 week later than planned if expected to improve tolerability. A delay in dose escalation of tirzepatide will not impact the planned total treatment duration. Deviations from the planned liraglutide dose escalation are not permitted.

7.4.1. Special Treatment Considerations

7.4.1.1. Management of Subjects with Gastrointestinal Symptoms

The tirzepatide and liraglutide dose-escalation schemes have been designed to minimize the development of intolerable GI symptoms. The dose-escalation period for tirzepatide is considered to be 3 weeks to reach the 10-mg dose. The dose-escalation period for liraglutide is considered to be 4 weeks to reach the 3-mg maintenance dose. During the dose-escalation

period, every effort should be made by the investigator to be able to escalate and maintain subjects on the corresponding study drug dosage.

To mitigate GI symptoms and manage subjects with poorly tolerated GI AEs, the investigator should:

- Advise subjects to eat smaller meals, for example, splitting 3 daily meals into 4 or more smaller meals, and to stop eating when they feel full.
- Prescribe symptomatic medication (for example, anti-emetic or anti-diarrheal medication) per local country availability and individual subject needs.
- Discontinuation from study treatment as per guidance provided in Section 8.1.1 (Permanent Discontinuation from Study Treatment).

7.5. Preparation/Handling/Storage/Accountability

The investigator or designee must confirm appropriate temperature conditions have been maintained, as communicated by sponsor, during transit for all IP received and any discrepancies are reported and resolved before use of the study treatment.

Only participants enrolled in the study may receive IP or study materials, and only authorized site staff may supply or administer IP while in the CRU. Subjects will receive training from only authorized site staff on how to self-administer liraglutide at home.

All IP should be stored in an environmentally controlled and monitored (manual or automated) area in accordance with the labeled storage conditions with access limited to the investigator and authorized site staff.

The investigator, institution, or the head of the medical institution (where applicable) is responsible for study treatment accountability, reconciliation, and record maintenance (such as receipt, reconciliation, and final disposition records).

7.6. Treatment Compliance

Tirzepatide and placebo will be administered at the study site and documentation of treatment administration will occur at the site. Liraglutide will be self-administered at home and compliance will be assessed at each visit by direct questioning and subject diaries. Deviations from the prescribed dosing regimen should be recorded in the eCRF.

Subjects randomized to receive liraglutide who are repeatedly (2 or more episodes) noncompliant with dosing will be reviewed by the investigator and sponsor to determine if the subject should continue treatment or be discontinued from the study.

7.7. Concomitant Therapy

Section 6.2 (Exclusion Criteria) provides a list of excluded medications in this study prior to randomization (e.g., weight-modifying agents, systemic glucocorticoids, glucose-lowering agents). These medications are also not allowed during the treatment period.

Subjects on stable concomitant medication at the time of study entry, other than those that are prohibited, should continue their regular, unchanged dose throughout the study.

In general, concomitant medication should be avoided; however, acetaminophen (1g, maximum 3 g/24 hours) may be administered at the discretion of the investigator for treatment of headaches etc. If the need for concomitant medication (other than acetaminophen) arises, inclusion or continuation of the subject may be at the discretion of the investigator after consultation with a Lilly CP or CRP.

Any medication used during the course of the study must be documented in source documents and entered into the eCRF.

7.8. Treatment after the End of the Study

Not applicable. Tirzepatide and liraglutide will not be made available to subjects after completion of the study.

8. Discontinuation Criteria

Subjects discontinuing from the treatment prematurely for any reason should complete an early termination visit and safety follow-up visit procedures per Section 2 (Schedule of Activities) of this protocol.

8.1. Discontinuation from Study Treatment

8.1.1. Permanent Discontinuation from Study Treatment

Possible reasons leading to permanent discontinuation of IP:

- The subject requests to discontinue IP
- If a subject is inadvertently enrolled and it is determined that continued treatment with IP would not be medically appropriate (Section 8.1.2 Discontinuation of Inadvertently Enrolled Subjects)
- If a subject is diagnosed with acute or chronic pancreatitis after randomization
- If a subject is confirmed to have developed T2DM after randomization
- If a subject is diagnosed with MTC after randomization
- If a subject is diagnosed with an active or untreated malignancy (other than basal or squamous cell skin cancer, in situ carcinomas of the cervix, or in situ prostate cancer) after randomization
- If the investigator, after consultation with the sponsor-designated medical monitor, determines that a systemic hypersensitivity reaction has occurred related to study drug administration
- If a subject is diagnosed with any other TEAE, SAE, or clinically significant laboratory value for which the investigator believes that permanent IP discontinuation is the appropriate measure to be taken
- If female subject becomes pregnant

Discontinuation of the IP for abnormal liver tests **should be considered** by the investigator when a subject meets 1 of the following conditions after consultation with the Lilly-designated medical monitor:

- ALT or AST >8× ULN
- ALT or AST >5× ULN sustained for more than 2 weeks
- ALT or AST >3× ULN **and** (TBL >2× ULN **or** international normalized ratio >1.5)
- ALT or AST >3× ULN, with the appearance of fatigue, nausea, vomiting, right upper quadrant pain or tenderness, fever, rash, and/or eosinophilia (>5%)

Subjects who discontinue IP early will be also discontinued from the study after performing the early termination (ET) visit and safety follow-up visit procedures, as specified in Section 2 (Schedule of Activities).

8.1.2. Discontinuation of Inadvertently Enrolled Subjects

If the sponsor or investigator identifies a subject who did not meet enrollment criteria and was inadvertently enrolled, a discussion must occur between the Lilly CP/CRP and the investigator to determine if the subject may continue in the study. If both agree it is medically appropriate to continue, the investigator must obtain documented approval from the Lilly CP/CRP to allow the inadvertently enrolled subject to continue in the study with or without continued treatment with IP.

8.2. Discontinuation from the Study

In addition to the situations that result in IP discontinuation described in Section 8.1.1 (Permanent Discontinuation from Study Treatment), subjects will be discontinued in the following circumstances:

- Enrollment in any other clinical study involving an investigational product or enrollment in any other type of medical research judged not to be scientifically or medically compatible with this study
- Participation in the study needs to be stopped for medical, safety, regulatory, or other reasons consistent with applicable laws, regulations, and good clinical practice (GCP)
- Investigator Decision
    - the investigator decides that the subject should be discontinued from the study
    - if the subject, for any reason, requires treatment with another therapeutic agent that has been demonstrated to be effective for treatment of the study indication, discontinuation from the study occurs prior to introduction of the new agent
- Subject Decision
    - the subject, or legal representative, requests to be withdrawn from the study.

8.3. Subjects Lost to Follow-up

A subject will be considered lost to follow-up if he or she repeatedly fails to return for scheduled visits and is unable to be contacted by the study site. Site personnel are expected to make diligent attempts to contact subjects who fail to return for a scheduled visit or were otherwise unable to be followed up by the site.

9. Study Assessments and Procedures

Section 2 lists the Schedule of Activities, detailing the study procedures and their timing (including tolerance limits for timing).

Appendix 2 lists the laboratory tests that will be performed for this study.

Appendix 4 provides a summary of the maximum number and volume of invasive samples, for all sampling, during the study.

Appendix 5 details the procedures for efficacy assessments.

The specifications in this protocol for the timings of safety and sample collections are given as targets to be achieved within reasonable limits. Modifications may be made to the time points based upon emerging clinical information. The scheduled time points may be subject to minor alterations; however, the actual time must be recorded correctly in the eCRF. Failure or delays (i.e., outside stipulated time allowances) in performing procedures or obtaining samples due to legitimate clinical issues (e.g., equipment technical problems, venous access difficulty, or subject defaulting or turning up late on an agreed scheduled procedure) will not be considered as protocol deviations but the CRU will still be required to notify the sponsor in writing via a file note.

Unless otherwise stated in subsections below, all samples collected for specified laboratory tests will be destroyed within 60 days of receipt of confirmed test results. Certain samples may be retained for a longer period, if necessary, to comply with applicable laws, regulations, or laboratory certification standards.

9.1. Efficacy Assessments

In this study, the measures used to assess mechanisms of action of study treatments with respect to energy intake, and central reward and appetite circuits are considered efficacy measures. The planned assessments will be performed at baseline, Week 3, and Week 6. Food intake tests will also be completed 3 and 4 weeks following the final dose of study drug in the follow-up period.

All parameter calculations, when applicable, and analyses are described in Section 10.3 (Statistical Analyses) or in the statistical analysis plan (SAP).

9.1.1. Appetite and Eating Behavior Assessments

To characterize the treatment effect on energy intake, clinic-based food intake test will be completed at the time points specified in the Schedule of Activities (Section 2). Energy intake (kcal) will be the primary endpoint.

The treatment effect on attitude towards food will be assessed with the use of several food-related questionnaires, completed at the time points specified in the Schedule of Activities (Section 2). The following questionnaires will be applied:

- appetite visual analog scale (VAS) for hunger, fullness, satiety, prospective food consumption, desire for specified foods, and overall appetite score

- Food Craving Inventory (FCI)
- Food Craving Questionnaire-State (FCQ-S)
- Eating Inventory
- Power of Food Scale

A full description of all appetite and eating behavior assessment methods are detailed in Appendix 5.

9.1.2. Neuroimaging Assessments

To characterize the treatment effect on central reward and appetite circuits in the brain, fMRI scans will be completed at the time points specified in the Schedule of Activities (Section 2).

- BOLD fMRI: Day -4 to Day -1 (baseline), Day 16 (Week 3, Visit 6), and Day 37 (Week 6, Visit 11)

On each day, BOLD fMRI scan will be completed in the fasting state and subjects will perform a food image task during each scan.

The principal brain reward areas to be examined will be the:

- insula
- medial frontal gyrus
- superior temporal gyrus
- precentral gyrus
- cingulate gyrus

In addition, exploratory brain reward areas to be examined will be the:

- hippocampus
- putamen
- orbitofrontal cortex
- ventral striatum

The change from baseline to Week 3 in BOLD fMRI brain activation in response to images of highly palatable foods versus non-food objects during fasting state will be considered as a secondary endpoint in the current study. All other data collected from functional neuroimaging will be considered exploratory.

A full description of the MRI assessment method, including the food image task, is detailed in Appendix 5.

Impulsivity assessments are described in Appendix 5.

9.1.3. *Hormone and Metabolite Assessments*

To explore potential effects of study treatment on hormones and metabolites related to appetite and the regulation of nutrient metabolism, plasma or serum samples will be collected at the time points provided in the Schedule of Activities (Section 2).

The changes from baseline to Week 3 and Week 6 of the following hormones and metabolites will be considered exploratory endpoints:

- amylin
- ghrelin
- GIP
- GLP-1
- leptin
- pancreatic polypeptide
- peptide YY
- insulin
- glucagon

In addition to safety monitoring, fasting plasma glucose and triglycerides will be measured as efficacy endpoints at baseline, Week 3 and Week 6.

9.2. Adverse Events

Investigators are responsible for monitoring the safety of subjects who have entered this study and for alerting Lilly or its designee to any event that seems unusual, even if this event may be considered an unanticipated benefit to the subject.

The investigator is responsible for the appropriate medical care of subjects during the study.

Investigators must document their review of each laboratory safety report.

The investigator remains responsible for following, through an appropriate health care option, AEs that are serious or otherwise medically important, considered related to the investigational product or the study, or that caused the subject to discontinue the investigational product before completing the study. The subject should be followed until the event resolves, stabilizes with appropriate diagnostic evaluation, or is reasonably explained. The frequency of follow-up evaluations of the AE is left to the discretion of the investigator.

The investigator will record all relevant AE and SAE information in the eCRF. After the ICF is signed, study site personnel will record, via eCRF, the occurrence and nature of each subject's preexisting conditions. Additionally, site personnel will record any change in the condition(s) and the occurrence and nature of any AEs.

The investigator will interpret and document whether or not an AE has a reasonable possibility of being related to study treatment, or a study procedure, concomitant treatment or pathologies.

A "reasonable possibility" means that there is a potential cause and effect relationship between the investigational product, study device and/or study procedure and the AE.

Planned surgeries should not be reported as AEs unless the underlying medical condition has worsened during the course of the study.

If a subject's IP is discontinued as a result of an AE, study site personnel must report this to Lilly or its designee via eCRF.

9.2.1. Serious Adverse Events

An SAE is any AE from this study that results in one of the following:

- death
- initial or prolonged inpatient hospitalization
- a life-threatening experience (that is, immediate risk of dying)
- persistent or significant disability/incapacity
- congenital anomaly/birth defect
- important medical events that may not be immediately life-threatening or result in death or hospitalization but may jeopardize the patient or may require intervention to prevent one of the other outcomes listed in the definition above
- when a condition related to the PFS necessitates medical or surgical intervention to preclude either permanent impairment of a body function or permanent damage to a body structure, the serious outcome of "required intervention" will be assigned.

Study site personnel must alert the Lilly CP/CRP, or its designee, of any SAE as soon as practically possible.

Additionally, study site personnel must alert Lilly Global Patient Safety, or its designee, of any SAE within 24 hours of investigator awareness of the event via a sponsor-approved method. If alerts are issued via telephone, they are to be immediately followed with official notification on study-specific SAE forms. This 24-hour notification requirement refers to the initial SAE information and all follow-up SAE information.

Investigators are not obligated to actively seek AEs or SAEs in subjects once they have discontinued from and/or completed the study (the subject summary eCRF has been completed). However, if the investigator learns of any SAE, including a death, at any time after a subject has been discharged from the study, and he/she considers the event reasonably possibly related to the study treatment or study participation, the investigator must promptly notify Lilly.

Pregnancy (maternal or paternal exposure to IP) does not meet the definition of an AE. However, to fulfill regulatory requirements any pregnancy should be reported following the SAE process to collect data on the outcome for both mother and fetus.

9.2.1.1. Suspected Unexpected Serious Adverse Reactions

Suspected unexpected serious adverse reactions (SUSARs) are serious events that are not listed in the IB and that the investigator reports as related to IP or procedure. Lilly has procedures that will be followed for the recording and expedited reporting of SUSARs that are consistent with global regulations and the associated detailed guidance.

9.2.2. Adverse Events of Special Interest

9.2.2.1. Hypoglycemia

Tirzepatide is an incretin which acts on the pancreatic β cell as a non-secretagogue; therefore, the risk of hypoglycemia is very low. Similarly, the risk of hypoglycemia is considered low with liraglutide.

If hypoglycemia occurs, each episode should be treated according to the standards of care by the investigator and additional monitoring of glucose levels may be requested at the investigator's discretion. As a general safety precaution, subjects will be trained during the lead-in period about signs and symptoms of hypoglycemia, how to treat hypoglycemia, and how to collect appropriate information for each episode of hypoglycemia.

Hypoglycemia may be identified by spontaneous reporting of symptoms from participants (whether confirmed or unconfirmed by simultaneous glucose values) or by blood glucose samples collected during study visits.

Subjects may, at the investigator's discretion, be given glucometers to assist in the evaluation of reported symptoms consistent with hypoglycemia. Subjects receiving glucometers will record relevant information (for example, glucose values, symptoms) in a diary.

Investigators should use the following definitions and criteria when diagnosing and categorizing an episode considered to be related to hypoglycemia (the PG values in this section refer to values determined by a laboratory or International Federation of Clinical Chemistry and Laboratory Medicine plasma-equivalent glucose meters and strips) (American Diabetes Association 2019):

Glucose Alert Value (Level 1):

- **Documented symptomatic hypoglycemia** is defined as any time a subject feels that he or she is experiencing symptoms and/or signs associated with hypoglycemia, and has a PG level of ≤70 mg/dL (≤3.9 mmol/L)
- **Documented asymptomatic hypoglycemia** is defined as any event not accompanied by typical symptoms of hypoglycemia, but with a measured PG ≤70 mg/dL (≤3.9 mmol/L)
- **Documented unspecified hypoglycemia** is defined as any event with no information about symptoms of hypoglycemia available, but with a measured PG ≤70 mg/dL (≤3.9 mmol/L)

Clinically Significant Hypoglycemia (Level 2):

- **Documented symptomatic hypoglycemia** is defined as any time a subject feels that he/she is experiencing symptoms and/or signs associated with hypoglycemia, and has a PG level of <54 mg/dL (<3.0 mmol/L)
- **Documented asymptomatic hypoglycemia** is defined as any event not accompanied by typical symptoms of hypoglycemia, but with a measured PG <54 mg/dL (<3.0 mmol/L)
- **Documented unspecified hypoglycemia** is defined as any event with no information about symptoms of hypoglycemia available, but with a measured PG <54 mg/dL (<3.0 mmol/L)

Severe Hypoglycemia (Level 3):

- **Severe hypoglycemia** is defined as an episode with severe cognitive impairment requiring the assistance of another person to actively administer carbohydrate, glucagon, or other resuscitative actions. These episodes may be associated with sufficient neuroglycopenia to induce seizure or coma. Blood glucose measurements may not be available during such an event, but neurological recovery attributable to the restoration of blood glucose to normal is considered sufficient evidence that the event was induced by a low blood glucose concentration.

Other Hypoglycemia Categories:

- **Nocturnal hypoglycemia** is defined as any hypoglycemic event that occurs between bedtime and waking

If a hypoglycemic event meets the criteria of severe, the investigator must record the event as serious on the AE eCRF and report it to Lilly as an SAE.

To avoid duplicate reporting, all consecutive blood glucose values ≤70 mg/dL (3.9 mmol/L) occurring within a 1-hour period may be considered to be a single hypoglycemic event (Weinberg et al. 2010; Danne et al. 2013).

In each case of suspected or confirmed hypoglycemia, it is important that the event be properly categorized, the effect of the intervention be assessed, and the frequency of hypoglycemia be evaluated. The role of dietary changes and physical exercise (or any other contributing factor) in the development of an event should be established. The subject should receive additional education, if deemed appropriate.

9.2.2.2. Pancreatitis

Glucagon-like peptide-1 receptor agonists have been associated with a possible risk of acute pancreatitis. Acute pancreatitis is defined as an AE of interest in all studies with tirzepatide including this study. Acute pancreatitis is an acute inflammatory process of the pancreas that may also involve peripancreatic tissues and/or remote organ systems (Banks and Freeman 2006). The diagnosis of acute pancreatitis requires 2 of the following 3 features:

- abdominal pain, characteristic of acute pancreatitis (generally located in the epigastrium and radiates to the back in approximately half the cases [Banks and Freeman 2006; Koizumi et al. 2006]; the pain is often associated with nausea and vomiting)
- serum amylase (total and/or pancreatic) and/or lipase ≥3× ULN
- characteristic findings of acute pancreatitis on computed tomography (CT) scan or MRI

If acute pancreatitis is suspected, appropriate laboratory tests (including levels of pancreatic amylase and lipase) should be obtained via the local laboratory. Imaging studies, such as abdominal CT scan with or without contrast, MRI, or gallbladder ultrasound, should be performed. If laboratory values and/or abdominal imaging support the diagnosis of acute pancreatitis, the subject must discontinue IP, and will be discontinued from the study after completing all ET and follow-up procedures. A review of the subject's medical data, including concomitant medications, should be conducted to assess potential causes of pancreatitis.

Each case of AE of pancreatitis must be reported. If typical signs and/or symptoms of pancreatitis are present and confirmed by laboratory values (lipase or amylase [total and/or pancreatic]) and imaging studies, the event must be reported as an SAE. For a potential case that does not meet all of these criteria, it is up to the investigator to determine the seriousness of the case (AE or SAE) and the relatedness of the event to IP.

Each subject will have measurements of amylase and lipase, which are part of the safety laboratory tests as shown in Section 2 (Schedule of Activities) to assess the effects of study treatment on pancreatic enzyme levels. Serial measures of pancreatic enzymes have limited clinical value for predicting episodes of acute pancreatitis in asymptomatic subjects (Nauck et al. 2017; Steinberg et al. 2017a; Steinberg et al. 2017b). Thus, further diagnostic follow-up of cases of asymptomatic pancreatic hyperenzymemia (lipase and/or pancreatic amylase ≥3× ULN) is not mandated but may be performed based on the investigator's clinical judgment and assessment of the subject's overall clinical condition. If further diagnostic assessment due to asymptomatic hyperenzymemia is warranted, it should follow Lilly standard algorithm for the monitoring of pancreatic enzymes (Appendix 7).

9.2.2.3. Thyroid Malignancies and C-Cell Hyperplasia

Individuals with personal or family history of MTC and/or MEN 2 will be excluded from the study, as well as those with calcitonin ≥20 pg/mL at screening. Participants who are diagnosed with MTC and/or MEN-2 during the study will have study drug stopped and should continue follow-up with an endocrinologist.

The assessment of thyroid safety during the study will include reporting of any case of thyroid malignancy, including MTC and papillary carcinoma, and measurements of calcitonin at screening. This data will be captured in the specific section of the eCRFs.

9.2.2.4. Hypersensitivity Events

Many drugs, but particularly biologic agents, carry the risk of systemic hypersensitivity reactions. If such a reaction occurs, additional data describing each symptom should be provided to the sponsor in the eCRF.

The sites should have appropriately trained medical staff and appropriate medical equipment available when study participants are receiving study drug. It is recommended that participants who experience a systemic hypersensitivity reaction be treated per the local standard of care.

In the case of generalized urticaria or anaphylaxis, additional blood samples should be collected as described in Appendix 8 (Recommended Laboratory Testing for Hypersensitivity Events). Laboratory results are provided to the sponsor via the central laboratory.

IP should be temporarily interrupted in any individual suspected of having a severe or serious allergic reaction to IP. IP may be restarted when/if it is safe to do so, in the opinion of the investigator. If IP is permanently discontinued, the subject will be discontinued from the study (Section 8.1.1 Permanent Discontinuation from Study Treatment).

9.2.2.5. Injection-Site Reactions

Injection-site assessments for local tolerability will be conducted, when reported as:

- an AE from a subject, or
- a clinical observation from an investigator.

Reported injection-site reactions will be characterized within the following categories:

- edema
- erythema
- induration
- itching
- pain

Injection-site reactions will be collected on the eCRF created for these events. At the time of AE occurrence, unscheduled samples may be collected for measurement of tirzepatide ADA and tirzepatide concentration if the event is suspected to be immune related by the investigator.

All injection-site reactions reported as AEs will be closely monitored until resolution. The report of a clinically significant AE of injection-site reaction may prompt notification of the sponsor, clinical photography, and referral for dermatologic evaluation and consideration of a skin biopsy and laboratory evaluations (ALT, AST, complete blood count with percent eosinophils, and additional immunogenicity testing).

Site staff will be provided with separate instructions/training on how to evaluate injection-site reactions and their severity in a consistent manner. Photographs of injection-site reactions may be taken in a standardized manner for record-keeping purposes; however, the photographs will not be used to evaluate the severity of injection-site reaction.

9.2.2.6. Hepatobiliary Disorders

All events of treatment-emergent biliary colic, cholecystitis, or other suspected events related to gallbladder disease should be evaluated and additional diagnostic tests performed, as needed. In cases of elevated liver markers, hepatic monitoring should be initiated as outlined in Section 9.4.7.1 (Hepatic Safety) and Appendix 6 (Hepatic Monitoring Tests for Treatment-Emergent Abnormality.

9.2.2.7. Severe Gastrointestinal Adverse Events

Tirzepatide or liraglutide may cause severe GI AEs, such as nausea, vomiting, and diarrhea. Information about severe GI AEs as well as antiemetic/antidiarrheal use will be collected in the AE form of the eCRF. For detailed information concerning the management of GI AEs, refer to Section 7.4.1.1 (Management of Subjects with Gastrointestinal Symptoms).

9.2.2.8. Acute Renal Events

Renal safety will be assessed based on laboratory renal functional assessment as well as assessment of AEs suggestive of acute renal failure or worsening of chronic renal failure. Subjects with GI AEs, including nausea, diarrhea, and vomiting are at increased risk of developing dehydration. Dehydration may cause a deterioration in renal function, including acute renal failure. Subjects should be advised to notify investigators in case of severe nausea, frequent vomiting, or symptoms of dehydration.

9.2.3. Complaint Handling

Lilly collects product complaints on IPs and drug delivery systems used in clinical trials in order to ensure the safety of study participants, monitor quality, and to facilitate process and product improvements.

Subjects should be instructed to contact the investigator as soon as possible if he or she has a complaint or problem with the investigational product or drug delivery system so that the situation can be assessed.

9.3. Treatment of Overdose

For the purposes of this study, an overdose of tirzepatide or liraglutide is considered any dose higher than the dose assigned through randomization.

For patients with suspected or confirmed overdose with tirzepatide or liraglutide, there is no specific antidote. The patient should be watched for GI symptoms and hypoglycemia. Treatment is supportive, depending on the subject's symptoms. For detailed information, refer to the IB for tirzepatide and package insert for liraglutide.

9.4. Safety

9.4.1. Laboratory Tests

For each subject, laboratory tests detailed in Appendix 2 should be conducted according to the Schedule of Activities (Section 2).

With the exception of safety laboratory test results that may unblind the study, Lilly or its designee will provide the investigator with the results of laboratory tests analyzed by a central vendor, if a central vendor is used for the study.

9.4.2. Vital Signs

Vital sign measurements should be taken before obtaining an ECG tracing, at visits where required (see Section 2, Schedule of Activities), and before collection of blood samples for laboratory testing. For each parameter, 2 measurements will be taken using the same arm. An appropriately sized cuff (cuff bladder encircling at least 80% of the arm) should be used to ensure the accuracy of blood pressure measurements. The arm used for the blood pressure measurement should be supported at the heart level. The recordings should be taken at least 1 minute apart. Each measurement of sitting heart rate and blood pressure needs to be recorded in the eCRF. Any AE related to changes in blood pressure and heart rate should be reported, per requirements provided in Section 9.2.

Unscheduled orthostatic vital signs should be assessed, if possible, during any AE of dizziness or posture-induced symptoms. Additional vital signs may be measured during each study period if warranted.

9.4.3. Body Weight and Waist Circumference

Weight and waist circumference will be measured according to the schedule provided in the Schedule of Activities (Section 2).

Subjects will be weighed at approximately the same time in the morning, before dosing and after an overnight fast and evacuation of the bowel and bladder, if possible. Weight will be measured twice on each scheduled occasion, with the subject stepping off the scale between measurements. A third measurement will be made if the first 2 measurements are >0.5 kg apart. The 2 closest measurements will be recorded. Wherever possible, the same scale will be used for all weight measurements throughout the study, and the scale will not be moved or recalibrated. Subjects will be weighed in light clothing. Weight measurements will be recorded in the source document and the eCRF.

Waist circumference will be measured at the midpoint between the inferior border of the rib cage and the superior aspect of the iliac crest. The subject will stand in a straight, upright position with feet together and arms at the side. The area measured will be cleared of all clothing other than undergarments. The measurement should be taken at the end of normal expiration. Two separate measures will be taken and steps are repeated until 2 measurements are obtained within 0.5 cm of each other.

9.4.4. Electrocardiograms

For each subject, single 12-lead ECGs should be collected according to the Schedule of Activities (Section 2).

Electrocardiograms must be recorded before collecting any blood samples. Subjects must be supine for approximately 5 to 10 minutes before ECG collection and remain supine but awake

during ECG collection. Electrocardiograms may be obtained at additional times, when deemed clinically necessary. All ECGs recorded should be stored at the investigational site.

Electrocardiograms will be interpreted by a qualified physician (the investigator or qualified designee) at the site as soon after the time of ECG collection as possible, and ideally while the subject is still present, to determine whether the subject meets entry criteria at the relevant visit(s) and for immediate subject management, should any clinically relevant findings be identified.

If a clinically significant finding is identified (including, but not limited to, changes in QT/QTc interval from baseline) after enrollment, the investigator will determine if the subject can continue in the study. The investigator, or qualified designee, is responsible for determining if any change in subject management is needed and must document his/her review of the ECG printed at the time of collection. Any clinically significant findings from ECGs that result in a diagnosis and that occur after the subject receives first dose of the IP should be reported to Lilly, or its designee, as an AE via eCRF.

9.4.5. Physical Examinations

Physical examinations and routine medical assessments will be conducted as specified in the Schedule of Activities (Section 2) and as clinically indicated.

9.4.6. Suicidal Ideation Assessment

Overweight and obese patients are at an increased risk of depression (Luppino et al. 2010). Depression can increase the risk of suicidal ideation and behavior. Therefore, study subjects will be screened at trial entry for depression, suicidal ideation and behavior using the C-SSRS and PHQ-9 as specified in the Schedule of Activities (Section 2). Subjects of higher risk of severe depression and/ or suicidality will be excluded (refer to EC 28-30, Section 6.2)

The C-SSRS (Columbia Lighthouse Project [WWW]) is a scale that captures the occurrence, severity, and frequency of suicidal ideation and/or behavior during the assessment period. The scale includes suggested questions to solicit the type of information needed to determine if suicidal ideation and/or behavior occurred. The tool was developed by the National Institute of Mental Health trial group for the purpose of being a counterpart to the Columbia Classification Algorithm of Suicide Assessment categorization of suicidal events. For this study, the C-SSRS is adapted for the assessment of the ideation and behavior categories only. The Intensity of Ideation and Lethality of Behavior sections are removed

The PHQ-9 is a validated self-report screening tool that assesses the presence and intensity of depressive symptoms. The PHQ-9, which incorporates the 9 Diagnostic and Statistical Manual-IV depression criteria as “0” (not at all) to “3” (nearly every day), was developed for use in primary care settings (Kroenke et al. 2001).

9.4.7. Safety Monitoring

The Lilly CP or CRP/scientist will monitor safety data throughout the course of the study.

Lilly will review SAEs within time frames mandated by company procedures. The Lilly CP or CRP will periodically review the following data:

- trends in safety data
- laboratory analytes including glucose, amylase, and lipase
- serious and nonserious AEs, including AEs of interest (see Section 9.2.2)

When appropriate, the Lilly CP or CRP will consult with the functionally independent Global Patient Safety therapeutic area physician or clinical research scientist.

9.4.7.1. Hepatic Safety

Close hepatic monitoring

Laboratory tests (Appendix 6), including ALT, AST, ALP, TBL, direct bilirubin, gamma-glutamyl transferase (GGT), and creatine kinase (CK), should be repeated within 48 to 72 hours to confirm the abnormality and to determine if it is increasing or decreasing, if 1 or more of these conditions occur:

If a participant with baseline results of ...	develops the following elevations:
ALT or AST <1.5× upper limit of normal (ULN)	ALT or AST ≥3× ULN
ALP <1.5× ULN	ALP ≥2× ULN
TBL <1.5× ULN	TBL ≥2× ULN (except for patients with Gilbert's syndrome)
ALT or AST ≥1.5× ULN	ALT or AST ≥2× baseline
ALP ≥1.5× ULN	ALP ≥2× baseline
TBL ≥1.5× ULN	TBL ≥1.5× baseline (except for patients with Gilbert's syndrome)

If the abnormality persists or worsens, clinical and laboratory monitoring, and evaluation for possible causes of abnormal liver tests should be initiated by the investigator in consultation with the Lilly-designated CP/CRP. At a minimum, this evaluation should include physical examination and a thorough medical history, including symptoms, recent illnesses (for example, heart failure, systemic infection, hypotension, or seizures), recent travel, history of concomitant medications (including over-the-counter), herbal and dietary supplements, history of alcohol drinking and other substance abuse.

Initially, monitoring of symptoms and hepatic biochemical tests should be done at a frequency of 1 to 3 times weekly, based on the participant's clinical condition and hepatic biochemical tests. Subsequently, the frequency of monitoring may be lowered to once every 1 to 2 weeks, if the participant's clinical condition and lab results stabilize. Monitoring of ALT, AST, ALP, and TBL should continue until levels normalize or return to approximate baseline levels.

Comprehensive hepatic evaluation

A comprehensive evaluation should be performed to search for possible causes of liver injury if one or more of these conditions occur:

If a participant with baseline results of...	develops the following elevations:
ALT or AST <1.5× ULN	ALT or AST ≥3× ULN with hepatic signs/symptoms*, or<br>ALT or AST ≥5× ULN
ALP <1.5× ULN	ALP ≥3× ULN
TBL <1.5× ULN	TBL ≥2× ULN (except for patients with Gilbert's syndrome)
ALT or AST ≥1.5× ULN	ALT or AST ≥2× baseline with hepatic signs/symptoms*, or<br>ALT or AST ≥3× baseline
ALP ≥1.5× ULN	ALP ≥2× baseline
TBL ≥1.5× ULN	TBL ≥1.5× baseline (except for patients with Gilbert's syndrome)

* Hepatic signs/symptoms are severe fatigue, nausea, vomiting, right upper quadrant abdominal pain, fever, rash, and/or eosinophilia >5%.

At a minimum, this evaluation should include physical examination and a thorough medical history, as outlined above, as well as tests for prothrombin time-international normalized ratio (PT-INR); tests for viral hepatitis A, B, C, or E; tests for autoimmune hepatitis; and an abdominal imaging study (for example, ultrasound or CT scan).

Based on the patient's history and initial results, further testing should be considered in consultation with the Lilly-designated medical monitor, including tests for hepatitis D virus (HDV), cytomegalovirus (CMV), Epstein-Barr virus (EBV), acetaminophen levels, acetaminophen protein adducts, urine toxicology screen, Wilson's disease, blood alcohol levels, urinary ethyl glucuronide, and blood phosphatidylethanol. Based on the circumstances and the investigator's assessment of the participant's clinical condition, the investigator should consider referring the participant for a hepatologist or gastroenterologist consultation, magnetic resonance cholangiopancreatography (MRCP), endoscopic retrograde cholangiopancreatography (ERCP), cardiac echocardiogram, or a liver biopsy.

Additional safety data collection in hepatic safety eCRF should be performed in study participants who meet 1 or more of the following conditions:

- Elevation of serum ALT to ≥5× ULN on 2 or more consecutive blood tests (if baseline ALT <1.5× ULN)
    - In participants with baseline ALT ≥1.5× ULN, the threshold is ALT ≥3× baseline on 2 or more consecutive tests
- Elevated serum TBL to ≥2× ULN (except for cases of known Gilbert's syndrome)
    - In participants with baseline TBL ≥1.5× ULN, the threshold should be TBL ≥2× baseline
- Elevation of serum ALP to ≥2× ULN on 2 or more consecutive blood tests
    - In participants with baseline ALP ≥1.5× ULN, the threshold is ALP ≥2× baseline on 2 or more consecutive blood tests
- Hepatic event considered to be a SAE
- Discontinuation of study drug due to a hepatic event.

Note: the interval between the 2 consecutive blood tests should be at least 2 days.

9.5. Pharmacokinetics

At the visits and times specified in the Schedule of Activities, venous blood samples of approximately 3 mL each will be collected to determine the plasma concentrations of tirzepatide. A maximum of 3 samples may be collected at additional time points during the study if warranted and agreed upon between both the investigator and sponsor. Instructions for the collection and handling of blood samples will be provided by the sponsor. The actual date and 24-hour clock time of each sampling will be recorded.

Drug concentration information that may unblind the study will not be reported to investigative site or blinded personnel until the study has been unblinded.

9.5.1. Bioanalysis

Samples will be analyzed at a laboratory approved by the sponsor and stored at a facility designated by the sponsor.

Concentrations of tirzepatide will be assayed using a validated liquid chromatography mass spectrometry method. Analyses of samples collected from placebo-treated subjects are not planned.

Bioanalytical samples collected to measure IP concentrations will be retained for a maximum of 1 year following last subject visit for the study. During this time, samples remaining after the bioanalyses may be used for exploratory analyses such as metabolism work, protein binding, and/or bioanalytical method cross-validation.

9.6. Pharmacodynamics

Briefly, PD assessments will include parameters of food intake, and central reward and appetite circuits. A detailed description of PD efficacy measures is provided in Section 9.1 (Efficacy Assessments). A description of procedures for obtaining the PD parameters is detailed in Appendix 5 and the analyses of these parameters is detailed in the SAP.

9.6.1. Immunogenicity Assessments

At the visits and times specified in the Schedule of Activities (Section 2), venous blood samples will be collected to determine antibody production against tirzepatide. To interpret the results of immunogenicity, a venous blood sample will be collected at the same time points to determine the plasma concentrations of tirzepatide.

Immunogenicity will be assessed by a validated assay designed to detect ADAs in the presence of tirzepatide at a laboratory approved by the sponsor. Antibodies may be further characterized and/or evaluated for their ability to neutralize the activity of tirzepatide on GIP and GLP-1 receptors. Positive tirzepatide ADA samples may also be tested for cross-reactivity against native GIP and GLP-1, and, if positive, may then be tested for neutralizing antibodies against native GIP and/or GLP-1.

All subjects will have an ADA sample measured at ET and at the safety follow-up visit. A risk-based approach will be used to monitor subjects who develop TE-ADA, defined in Section 10.3.4 (Evaluation of Immunogenicity).

Every attempt should be made to contact subjects for the follow-up immunogenicity assessment; however, if subjects are unwilling or unable to return for the visit, this is not considered a protocol violation.

Samples will be retained for a maximum of 15 years after the last subject visit, or for a shorter period if local regulations and IRBs allow, at a facility selected by the sponsor. The duration allows the sponsor to respond to future regulatory requests related to tirzepatide. Any samples remaining after 15 years will be destroyed.

9.7. Genetics

A blood sample will be collected for pharmacogenetic analysis as specified in the Schedule of Activities, where local regulations allow.

Samples will not be used to conduct unspecified disease or population genetic research either now or in the future. Samples will be used to investigate variable exposure or response to tirzepatide and to investigate genetic variants thought to play a role in T2DM, obesity, and/or diabetic complications. Assessment of variable response may include evaluation of AEs or differences in efficacy.

All samples will be coded with the subject number. These samples and any data generated can be linked back to the subject only by the investigative site personnel.

Samples will be retained for a maximum of 15 years after the last subject visit, or for a shorter period if local regulations and/or IRBs impose shorter time limits, for the study at a facility selected by Lilly or its designee. This retention period enables use of new technologies, response to regulatory questions, and investigation of variable response that may not be observed until later in the development of tirzepatide or after tirzepatide is commercially available.

Molecular technologies are expected to improve during the 15-year storage period and therefore cannot be specifically named. However, existing approaches include whole genome or exome sequencing, genome wide association studies, multiplex assays, and candidate gene studies. Regardless of technology utilized, data generated will be used only for the specific research scope described in this section.

9.8. Biomarkers

Biomarker research is performed to address questions of relevance to drug disposition, target engagement, pharmacodynamics, mechanism of action, variability of subject response (including safety), and clinical outcome. Sample collection is incorporated into clinical studies to enable examination of these questions through measurement of biomolecules including DNA, RNA, proteins, lipids, and other cellular elements.

Serum and plasma samples for non-pharmacogenetic biomarker research will be collected at the times specified in the Schedule of Activities (Section 2) where local regulations allow.

Samples will be used for research on the drug target, disease process, variable response to tirzepatide, pathways associated with T2DM, obesity, and/or diabetic complications, mechanism of action of tirzepatide, and/or research method, or for validating diagnostic tools or assay(s) related to T2DM, obesity, and/or diabetic complications.

All samples will be coded with the subject number. These samples and any data generated can be linked back to the subject only by the investigative site personnel.

Samples will be retained for a maximum of 15 years after the last subject visit, or for a shorter period if local regulations and/or IRBs impose shorter time limits, at a facility selected by Lilly or its designee. This retention period enables use of new technologies, response to regulatory questions, and investigation of variable response that may not be observed until later in the development of tirzepatide or after tirzepatide is commercially available.

9.9. Health Economics

This section is not applicable for this study.

9.10. Passive Detection of Eating Activity

To evaluate and refine an eating activity pattern recognition model, motion sensor data will be collected passively via a wrist-wearable device during the last week of the follow-up period of the study (Section 2; Schedule of Activities).

At Visit 12 (approximately 3 weeks following the last dose of study drug), subjects will visit the CRU on an outpatient basis, where they will be provided with a wrist-wearable device to be worn on their dominant arm. The device will be worn during an ad libitum food intake test and subjects will then continue to wear the device for a further 4 days at home. The device should always be worn on dominant hand/wrist (24/7) on the days specified.

On all days the subject is wearing the device at home (approximately 4 days), eating events will be detected (food and corresponding utensils, if applicable) in free-living conditions using the Remote Food Photography Method© (RFPM) and SmartIntake® app (Appendix 5). Energy and gram intake will not be calculated with the RFPM/SmartIntake® app, rather, the date and timestamp of the eating events will be evaluated in relation to data from the wrist-worn motion sensor.

At Visit 13 (approximately 4 weeks following the last dose of study drug), subjects will again return to the CRU on an outpatient basis. While the device is still being worn, subjects will complete an ad libitum food intake test. Subjects will then be required to return the wrist-wearable device to the CRU staff.

The start and end time of each ad libitum food intake test during the follow-up period should be recorded in the eCRF.

10. Statistical Considerations and Data Analysis

10.1. Sample Size Determination

The sample size is derived such that the primary endpoint shall be met. In particular, that is to compare the change from baseline treatment difference in ad libitum calorie intake during lunch.

The foundation for making a reasonable assumption for effect size and variability is based on both unblinded data from I8F-MC-GPGC (Study GPGC) and blinded data from I8F-MC-GPGT (Study GPGT). Study GPGC is a Phase 1 study to investigate safety, tolerability, PK, and PD of tirzepatide subcutaneously administered once weekly to Japanese patients with T2DM. Study GPGT is a 28-week Phase 1 study designed to examine α and β cell function, insulin sensitivity, glucose and lipid metabolism, food intake (ad libitum calorie intake during lunch), and energy expenditure in patients with T2DM treated with tirzepatide.

Combining the results from Study GPGC and blinded data from Study GPGT, the estimated variability (standard deviation) is 289 kcal and the estimated treatment difference between placebo and tirzepatide is 212 kcal. Therefore, at least 80% power can be achieved based on 2 sample t-test using 2-sided test at alpha level of 0.05 with a sample size of 31 completers per arm. Assuming 15% discontinuation rate, approximately 111 subjects (37 subjects per treatment arm) are planned to be randomly assigned.

10.2. Populations for Analyses

10.2.1. Study Participant Disposition

A detailed description of subject disposition will be provided at the end of the study. All subjects who discontinue from the study will be identified, and the extent of their participation in the study will be reported. If known, a reason for their discontinuation from study will be given.

10.2.2. Study Participant Characteristics

Demographic and baseline characteristics will be summarized by treatment group. These data will be summarized using standard descriptive statistics.

10.2.3. Treatment Compliance

Treatment compliance will be listed and summarized using standard descriptive statistics.

10.3. Statistical Analyses

Statistical analysis of this study will be the responsibility of Eli Lilly and Company or its designee.

Pharmacodynamic analyses will be conducted on data from all subjects who receive at least 1 dose of tirzepatide, placebo, or liraglutide and have evaluable PD data. Sensitivity analyses may be performed as deemed necessary.

Safety analyses will be conducted for all enrolled subjects, whether or not they completed all protocol requirements.

Additional exploratory analyses of the data may be conducted as deemed appropriate.

10.3.1. Safety Analyses

10.3.1.1. Clinical Evaluation of Safety

The incidence of AEs for each treatment will be presented by severity and by association with IP as perceived by the investigator. Adverse events reported to occur prior to study entry will be distinguished from those reported as new or increased in severity during the study. Each AE will be classified by the most suitable term from the medical regulatory dictionary.

All AEs and SAEs will be reported.

10.3.1.2. Statistical Evaluation of Safety

Safety parameters that will be assessed include safety lab parameters (including tirzepatide ADA), vital signs, TEAEs (including TEAEs of special interest), and SAEs.

Summary statistics will be presented by treatment for the safety measures. Additionally, statistical analysis may be performed if warranted upon review of the data.

The analysis details will be provided in the SAP.

10.3.2. Pharmacokinetic Analyses

10.3.2.1. Pharmacokinetic Parameter Estimation

Sparse PK samples will be collected across the 6-week treatment duration according to Section 2 (Schedule of Activities). Tirzepatide concentrations will be determined to support an understanding of tirzepatide exposure over the treatment duration and compare with expected tirzepatide PK.

10.3.2.2. Pharmacokinetic Statistical Inference

No summaries or analyses of PK parameters are planned. Tirzepatide concentrations may be summarized by week.

10.3.3. Pharmacodynamic Analyses

10.3.3.1. Pharmacodynamic Parameter Estimation

Pharmacodynamic parameters will be measured at baseline, Week 3, and Week 6, to assess the effect of study treatments on energy intake and central reward and appetite circuits in the brain. Parameters are provided in Section 9.1 and additional considerations are described below.

Measures of Appetite and Food Intake

- Energy intake assessed by ad libitum food intake test: The primary endpoint is energy intake (kcal) during lunch in a clinical setting. The lunch consists of a buffet meal, where subjects can eat as much or as little as they wish. Other food intake variables such as grams of carbohydrate, protein, and fat (saturated and unsaturated) consumed, as well as the percent of energy intake (kcal) from carbohydrate, protein, and fat (saturated and unsaturated) may also be computed if deemed necessary.

- Appetite VAS: The VAS scales will be analyzed as continuous variables on the 0-100 scale for individual components. Overall appetite score is calculated as the average of the 4 individual scores (satiety + fullness + [100-prospective food consumption] + [100-hunger]/4) (Flint et al. 2000; van Can et al. 2014). The higher overall appetite score indicates less appetite and the lower score indicates more appetite.

Measures of Central Reward and Appetite Circuits

- BOLD fMRI: Mean percent BOLD signal differences between views of highly palatable food (HF-HS and HF-HCCHO) versus non-food objects (e.g. office supplies, furniture) will be calculated within 5 principal food reward-related regions of interest; distinct locations within the insula, medial frontal gyrus, superior temporal gyrus, precentral gyrus, and cingulate gyrus. Additional analysis will examine the BOLD signal differences in 4 exploratory food reward-related regions of interest; the hippocampus, putamen, orbitofrontal cortex, and ventral striatum.

Additional analyses using other functional contrasts (e.g., Highly Palatable foods versus Not Highly Palatable Foods, not highly palatable foods versus nonfood objects) may be conducted as necessary. Other PD measures and exploratory measures will be described in the SAP.

10.3.3.2. Pharmacodynamic Statistical Inference

The primary endpoint will be analyzed using an analysis of covariance (ANCOVA) to compare the effect of tirzepatide versus placebo at Week 3 on energy intake with terms, treatment, baseline BMI stratum, and baseline energy (kcal) intake as a covariate in the model. The response variable will be the change from baseline to Week 3 in energy (kcal) intake, as assessed by ad libitum food intake test.

The primary analysis will show least squares means of energy (kcal) intake by treatment and the treatment difference of tirzepatide versus placebo as well as standard error and 95% confidence interval. All tests will be done at the 2-sided 0.05 α level, unless otherwise specified.

Analyses for secondary and exploratory endpoints will be specified in SAP.

10.3.4. Evaluation of Immunogenicity

The frequency and percentage of subjects with preexisting ADA and with TE-ADA+ to tirzepatide may be tabulated. Treatment-emergent ADAs are defined as those with a titer 2-fold (1 dilution) greater than the minimum required dilution if no ADAs were detected at baseline (treatment-induced ADA) or those with a 4-fold (2 dilutions) increase in titer compared to baseline if ADAs were detected at baseline (treatment-boosted ADA). For the TE-ADA+ patients the distribution of maximum titers may be described. The frequency of neutralizing antibodies may also be tabulated in TE-ADA+ patients.

The relationship between the presence of antibodies and the PK parameters and PD response including safety and efficacy to tirzepatide may be assessed.

10.3.5. Interim Analyses

No interim analyses are planned for this study. If an unplanned interim analysis is deemed necessary, the Lilly CP, CRP/investigator, or designee will consult with the appropriate medical director or designee to determine if it is necessary to amend the protocol.

10.3.6. Exploratory Analysis for Motion Sensor Modeling

An existing model for eating activity recognition based on data from wrist-wearable device motion sensors (accelerometer and gyroscope) will be applied to classify time segments as involving eating or not. Classification accuracy of this model will be evaluated relative to the ground truth labels collected in this study. Ground truth labels are defined as the meal times obtained by the CRU staff during the follow-up period days, on which ad libitum food intake tests will be performed; a time when the food is weighed before and after the meal will be used as the time of meal start and end, respectively. Ground truth will also be obtained on the days when subjects use the RFPM and SmartIntake® app in free-living conditions, where the timestamps associated with food photographs before and after the meal will be used as the time of meal start and end, respectively.

Classification accuracy will be characterized using sensitivity, specificity, precision, and area under the curve.

The existing model will be refined by re-training it with additional data from this study. Data collected in this study will be split into training, validation, and test datasets to ensure that data used in model re-training will not be used in the assessment of the re-trained model accuracy. Re-trained model accuracy will be assessed using the same metrics as described above.

The analyses specified in this section will be detailed in the digital biomarker analysis plan.

11. References

[ADA] American Diabetes Association. Glycemic targets. Standards of medical care in diabetes—2019. *Diabetes Care*. 2019;42(Suppl. 1):S61-S70.

Bandini LG, Schoeller DA, Cyr HN, Dietz WH. Validity of reported energy intake in obese and nonobese adolescents. *Am J Clin Nutr.* 1990;52(3):421-425.

Banks PA, Freeman ML. Practice guidelines in acute pancreatitis. *Am J Gastroenterol.* 2006;101(10):2379-2400.

Beasley J, Riley WT, Jean-Mary J. Accuracy of a PDA-based dietary assessment program. *Nutrition.* 2005;21(6):672-677.

Beaton GH, Burema J, Retinbaugh C. Errors in the interpretation of dietary assessments. *Am J Clin Nutr.* 1997;65(4 Suppl):11000S-1107S. doi:10.1093/ajcn/65.4.1100S.

Blechert J, Meule A, Busch NA, Ohla K. Food-pics: an image database for experimental research on eating and appetite. *Front Psychol.* 2014;5:617.

Blundell J, Finlayson G, Axelsen M, Flint A, Gibbons C, Kvist T, Hjerpsted JB. Effects of once-weekly semaglutide on appetite, energy intake, control of eating, food preference and body weight in subjects with obesity. *Diabetes Obes Metab*. 2017;19:1242–1251.

Brown BM, Peiffer JJ, Martins RN. Multiple effects of physical activity on molecular and cognitive signs of brain aging: can exercise slow neurodegeneration and delay Alzheimer's disease? *Mol Psychiatry.* 2013;18(8):864-874.

Calanna S, Christensen M, Holst JJ, Laferrère B, Gluud LL, Visbøll T, Knopp FK. Secretion of glucose-dependent insulinotropic polypeptide in patients with type 2 diabetes: a systematic review and meta-analysis of clinical studies. *Diabetes Care.* 2013;36(10):3346-3352.

Campbell JE, Drucker DJ. Pharmacology, physiology, and mechanisms of incretin hormone action. *Cell Metab*. 2013;17(6):818-837.

Cepeda-Benito A, Gleaves DH, Williams TL, Erath SA. The development and validation of the state and trait food-cravings questionnaires. *Behav Ther.* 2000;31(1):151-173.

Coskun T, Sloop K, Loghin C, Alsina-Fernandez J, Urva S, Bokvist KB, Cui X, Briere DA, Cabrera O, Roell WC, Kuchibhotla U, Moyers JS, Benson CT, Gimeno RE, D'Alessio DA, Haupt A. LY3298176, a novel dual GIP and GLP-1 receptor agonist for the treatment of type 2 diabetes mellitus: From discovery to clinical proof of concept. *Molecular metabolism*. 2018;18:3-14.

Columbia Lighthouse Project. Columbia-Suicide Severity Rating Scale (C-SSRS) website. Available at: http://www.cssrs.columbia.edu. Accessed 21 February 2020.

Danne T, Philotheou A, Goldman D, Guo X, Ping L, Cali A, Johnston P. A randomized trial comparing the rate of hypoglycemia – assessed using continuous glucose monitoring – in 125 preschool children with type 1 diabetes treated with insulin glargine or NPH insulin (the PRESCHOOL study). *Pediatr Diabetes*. 2013;14(8):593-601.

Farr OM, Sofopoulos M, Tsoukas MA, Dincer F, Thakkar B, Sahin-Efe A, Filippaios A, Bowers J, Srnka A, Gavrieli A, Ko BJ, Liakou C, Kanyuch N, Tseleni-Balafouta S, Mantzoros CS.

GLP-1 receptors exist in the parietal cortex, hypothalamus and medulla of human brains and the GLP-1 analogue liraglutide alters brain activity related to highly desirable food cues in individuals with diabetes: a crossover, randomised, placebo-controlled trial. *Diabetologia.* 2016;59(5):954-965.

Farr OM, Upadhyay J, Rutagengwa C, DiPrisco B, Ranta Z, Adra A, Bapatla N, Douglas VP, Douglass KAA, Nolen-Doerr E, Matthew H, Mantzoros CS. Longer-term liraglutide administration at the highest dose approved for obesity increases reward-related orbitofrontal cortex activation in response to food cues: Implications for plateauing weight loss in response to anti-obesity therapies. *Diabetes Obes Metab.* 2019;21(11):2459-2464.

Finan B, Müller TD, Clemmensen C, Perez-Tilve D, DiMarchi RD, Tschöp MH. Reappraisal of GIP pharmacology for metabolic disease. *Trends Mol Med*. 2016;22(5):359-376.

Flint A, Raben A, Blundell JE, Astrup A. Reproducibility, power and validity of visual analogue scales in assessment of appetite sensations in single test meal studies. *Int J Obes Relat Metab Disord.* 2000;24:38-48.

Frias JP, Nauck MA, Van J, Kutner ME, Cui X, Benson C, Urva S, Gimeno RE, Milicevic Z, Robins D, Haupt A. Efficacy and safety of LY3298176, a novel dual GIP and GLP-1 receptor agonist, in patients with type 2 diabetes: a randomised, placebo-controlled and active comparator-controlled phase 2 trial. *The Lancet.* 2018;392(10160): 2180-2193.

Frias JP, Nauck MA, Van J, Benson C, Bray R, Cui X, Milicevic Z, Urva S, Haupt A, Robins D. Efficacy and tolerability of tirzepatide, a dual glucose-dependent insulinotropic peptide and glucagon-like peptide-1 receptor agonist in patients with type 2 diabetes: a 12-week, randomized, double-blind, placebo-controlled study to evaluate different dose-escalation regimens. *Diabetes Obes Metab*. 2020; Jan 27. doi: 10.1111/dom.13979.

Geiselman PJ, Anderson AM, Dowdy ML, West DB, Redman SM, Smith SR. Reliability and validity of a macronutrient self-selection paradigm and a food preference questionnaire. *Physiol Behav*. 1998;63(5):919-928.

Hayes MR and Schmidt HD. GLP-1 influences food and drug reward. *Curr Opin Behav Sci.* 2016;9:66-70.

Holland D, Kuperman JM, Dale AM. Efficient correction of inhomogenous static magnetic field-induced distortion in Echo Planar Imaging. *Neuroimage*. 2010;50(1):175-183.

King JL, Fearnbach SN, Ramakrishnapillai S, Shankpal P, Geiselman PJ, Martin CK, Murray KB, Hicks JL, McClernon FJ, Apolzan JW, Carmichael OT. Perceptual Characterization of the Macronutrient Picture System (MaPS) for Food Image fMRI. Front Psychol. 2018;26(9):17.

Kirby KN and Finch JC. The hierarchical structure of self-reported impulsivity. *Pers Individ Dif.* 2010;48(6):704-713.

Kirby KN and Marakovic NN. Delay-discounting probabilistic rewards: Rates decrease as amounts increase. *Psychon Bull Rev.* 1996;3(1):100-104.

Koizumi M, Takada T, Kawarada Y, Hirata K, Mayumi T, Yoshida M, Sekimoto M, Hirota M, Kimura Y, Takeda K, Isaji S, Otsuki M, Matsuno S; JPN. JPN Guidelines for the management of acute pancreatitis: diagnostic criteria for acute pancreatitis. *J Hepatobiliary Pancreat Surg.* 2006;13(1):25-32.

Kroenke K, Spitzer RL, Williams JB. The PHQ-9: validity of a brief depression severity measure. *J Gen Intern Med*. 2001;16(9):606-613.

Lowe MR, Butryn ML, Didie ER, Annuziato RA, Thomas JG, Crerand CE, Ochner CN, Coletta MC, Bellace D, Wallaert M, Halford J. The Power Food Scale. A new measure of the psychological influence of the food environment. *Appetite.* 2009;53(1):114-118.

Luppino FS, de Wit LM, Bouvy PF, Stijnen T, Cuijpers P, Penninx BW, Zitman FG. Overweight, obesity, and depression: a systematic review and meta-analysis of longitudinal studies. *Arch Gen Psychiatry.* 2010;67(3):220-229.

Martin CK, Correa JB, Han H, Allen HR, Rood J, Champagne CM, Gunturk BK, Bray GA. Validity of the Remote Food Photography Method (RFPM) for estimating energy and nutrient intake in near real-time. *Obesity (Silver Spring).* 2012;20(4):891-899.

Martin CK, Han H, Coulon Sm, Allen HR, Champagne CM, Anton SD. A novel method to remotely measure food intake of free-living individuals in real time: the remote food photography method. *Br J Nutr.* 2009;11(3):446-456.

Martin CK, Coulon SM, Markward N, et al. Association between energy intake and viewing television, distractibility, and memory for advertisements. *Am J Clin Nutr.* 2009;89:37–44.

Martin CK, Rednam LM, Zhang J, et al. Lorcaserin, a 5-HT_{2C} receptor agonist, reduces body weight by decreasing energy intake without influencing energy expenditure. *J Clin Endocrinol Metab.* 2011;96:837-845.

Martin CK, O'Neil PM, Pawlow L. Changes in food cravings during low-calorie and very-low-calorie diets. *Obesity.* 2006;14(1):115-121.

Moreno S, Rodriguez S, Fernandez MC, Tamez J, Cepeda-Benito A. Clinical validation of the trait and state versions of the Food Craving Questionnaire. *Assessment.* 2008;15(3):375-387.

Nauck MA, Frossard J-L, Barkin JS, Anglin G, Hensley IE, Harper KD, Milicevic Z. Assessment of pancreas safety in the development program of once-weekly GLP-1 receptor agonist dulaglutide. *Diabetes Care*. 2017;40(5):647-654.

Nauck MA, Meier JJ. GIP and GLP-1: Stepsiblings Rather Than Monozygotic Twins Within the Incretin Family. *Diabetes.* 2019;68(5):897-900.

Nauck MA, Meier JJ. The incretin effect in healthy individuals and those with type 2 diabetes: physiology, pathophysiology, and response to therapeutic interventions. *Lancet Diabetes Endocrinol.* 2016;4(6):525-536.

Patton JH, Stanford MS, Barratt ES. Factor structure of the Barratt impulsiveness scale. *J Clin Psychol.* 1995;51(6):768-774.

Reid RC, Cyders MA, Moghaddam JF, Fong TW. Psychometric properties of the Barratt impulsiveness scale in patients with gambling disorders, hypersexuality, and methamphetamine dependence. *Addict Behav*. 2014;39(11):1640-1645.

Saxenda [package insert]. Plainsboro, NJ: Novo Nordisk, 2018.

Steinberg WM, Buse JB, Ghorbani MLM, Ørsted DD, Nauck MA; LEADER Steering Committee; LEADER Trial Investigators. Amylase, lipase, and acute pancreatitis in people with type 2 diabetes treated with liraglutide: results from the LEADER Randomized Trial. *Diabetes Care.* 2017a;40(7):966-972.

Steinberg WM, Rosenstock J, Wadden TA, Donsmark M, Jensen CB, DeVries JH. Impact of liraglutide on amylase, lipase, and acute pancreatitis in participants with overweight/obesity and normoglycemia, prediabetes, or type 2 diabetes: secondary analyses of pooled data from the SCALE clinical development program. *Diabetes Care.* 2017b;40(7):839-848.

Stunkard AJ and Messick S. Eating Inventory Manual (The Psychological Corporation). 1988. San Antonio, TX: Harcourt Brace & Company.

Stunkard AJ and Messick S. The three-factor eating questionnaire eto measure dietary restraint, disinhibition and hunger. *J Psychosom Res*. 1985;29(1):71-83.

Tang DW, Fellows LK, Small DM, Dagher A. Food and drug cues activate similar brain regions: a meta-analysis of functional MRI studies. *Physiol Behav.* 2012;106(3):317-324.

ten Kulve JS, Veltman DJ, van Bloemendaal L, Barkhof F, Drent ML, Diamant M, Ijezerman RG. Liraglutide Reduces CNS Activation in Response to Visual Food Cues Only After Short-term Treatment in Patients with Type 2 Diabetes. *Diabetes Care.* 2016;39(2):214-224.

Tran KM, Johnson RK, Soultanakis RP, Matthews DE. In-person vs telephone-administered multiple-pass 24-hour recalls in women: validation with doubly labeled water. *J Am Diet Assoc.* 2000;100(7):777-783.

Schoeller DA, Bandini LG, Dietz WH. Inaccuracies in self-reported intake identified by comparison with the doubly labelled water method. *Can J Physiol Pharmacol.* 1990;68(7):941-949.

Stone AA, Shiffman S. Ecological momentary assessment (EMA) in behavioral medicine. Ann *Behav Med*. 1994;16(3):199-202.

[USDA] United States Department of Agriculture. United States Department of Agriculture, Agricultural Research Service. 2018. USDA Food and Nutrient Database for Dietary Studies 2015-2016.

van Bloemendaal L, IJzerman RG, ten Kulve JS, Barkhof F, Konrad RJ, Drent ML, Veltman DJ, Diamant M. GLP-1 receptor activation modulates appetite- and reward-related brain areas in humans. *Diabetes.* 2014;63(12):4186-4196.

van Can J, Sloth B, Jensen CB, Flint A, Blaak EE, Saris WH. Effects of the once-daily GLP-1 analog liraglutide on gastric emptying, glycemic parameters, appetite and energy metabolism in obese, non-diabetic adults. *Int J Obes*. 2014;38(6):784-793.

Weinberg ME, Bacchetti P, Rushakoff RJ. Frequently repeated glucose measurements overestimate the incidence of inpatient hypoglycemia and severe hyperglycemia. *J Diabetes Sci Technol*. 2010;4(3):577-582.

White MA, Whisenhunt BL, Williamson DA, Greenway FL, Netemeyer RG. Development and validation of the food-craving inventory. *Obes Res.* 2002;10(2):107-114.

Williamson DA. Digital photography: A new method for estimating food intake in cafeteria settings. *Eat Weight Disord.* 2004;9(1):24-28.

Williamson DA, Allen HR, Martin PD, Alfonso AJ, Gerald B, Hunt A. Comparison of digital photography to weighed and visual estimation of portion sizes. *J Am Diet Assoc*. 2003;103(9):1139-1145.

Womble LG, Wadden TA, Chandler JM, Martin AR. Agreement between weekly vs. daily assessment of appetite. *Appetite.* 2003;40(2):131-135.

12. Appendices

Appendix 1. Abbreviations and Definitions

Term	Definition
**ADA**	anti-drug antibody
**AE**	adverse event: Any untoward medical occurrence in a patient or clinical investigation subject administered a pharmaceutical product that does not necessarily have a causal relationship with this treatment. An AE can therefore be any unfavorable and unintended sign (including an abnormal laboratory finding), symptom, or disease temporally associated with the use of a medicinal (investigational) product, whether or not related to the medicinal (investigational) product.
**ALP**	alkaline phosphatase
**ALT**	alanine aminotransferase
**AST**	aspartate aminotransferase
**BAT**	basophil activation test
**BIS**	Barratt Impulsiveness Scale
**blinding**	A procedure in which one or more parties to the study are kept unaware of the treatment assignments. Unless otherwise specified, blinding will remain in effect until final database lock.<br>A single-blind study is one in which the investigator and/or his staff are aware of the treatment but the subject is not, or vice versa, or when the sponsor is aware of the treatment but the investigator and/his staff and the subject are not. A double-blind study is one in which neither the subject nor any of the investigator or sponsor staff who are involved in the treatment or clinical evaluation of the subjects are aware of the treatment received
**BMI**	body mass index
**BOLD**	blood oxygenation level dependent
**CAT**	computer adaptive test
**CBF**	cerebral blood flow
**complaint**	A complaint is any written, electronic, or oral communication that alleges deficiencies related to the identity, quality, purity, durability, reliability, safety or effectiveness, or performance of a drug or drug delivery system.
**compliance**	Adherence to all the study-related requirements, good clinical practice (GCP) requirements, and the applicable regulatory requirements.
**confirmation**	A process used to confirm that laboratory test results meet the quality requirements defined by the laboratory generating the data and that Lilly is confident that results are accurate. Confirmation will either occur immediately after initial testing or will require that samples be held to be retested at some defined time point, depending on the steps required to obtain confirmed results.
**CP**	Clinical Pharmacologist

**CRP**	Clinical Research Physician: Individual responsible for the medical conduct of the study. Responsibilities of the CRP may be performed by a physician, clinical research scientist, global safety physician or other medical officer.
**CRU**	clinical research unit
**C-SSRS**	Columbia-Suicide Severity Rating Scale
**CT**	computed tomography
**ECG**	electrocardiogram
**eCRF**	electronic case report form
**enroll**	The act of assigning a subject to a treatment. Subjects who are enrolled in the study are those who have been assigned to a treatment.
**ET**	early termination
**FCI**	Food Craving Inventory
**FCQ-S**	Food Craving Questionnaire-State
**fMRI**	functional magnetic resonance imaging
**GCP**	good clinical practice
**GI**	gastrointestinal
**GIP**	glucose-dependent insulinotropic
**GIPR**	glucose-dependent insulinotropic receptor
**GLP-1**	glucagon-like peptide 1
**GLP-1R**	glucagon-like peptide1 receptor
**GLP-1RA**	glucagon-like peptide 1 receptor agonist
**HCV**	hepatitis C virus
**HCCHO**	high carbohydrate
**HF**	high fat
**HIV**	human immunodeficiency virus
**HP**	high protein
**HS**	high sugar
**IB**	Investigator's Brochure

**informed consent/informed consent form (ICF)**	A process by which a subject voluntarily confirms his or her willingness to participate in a particular study, after having been informed of all aspects of the study that are relevant to the subject's decision to participate. Informed consent is documented by means of a written, signed and dated informed consent form.
**ICH**	International Council for Harmonisation
**Ig**	immunoglobulin
**IP**	investigational product: A pharmaceutical form of an active ingredient or placebo being tested or used as a reference in a clinical study, including products already on the market when used or assembled (formulated or packaged) in a way different from the authorized form, or marketed products used for an unauthorized indication, or marketed products used to gain further information about the authorized form.
**investigator**	A person responsible for the conduct of the clinical study at a study site. If a study is conducted by a team of individuals at a study site, the investigator is the responsible leader of the team and may be called the principal investigator.
**IRB**	institutional review board
**Legal Representative**	An individual or judicial or other body authorized under applicable law to consent, on behalf of a prospective subject, to the subject's participation in the clinical study.
**LCCHO**	low carbohydrate
**LF**	low fat
**LP**	low protein
**MAD**	multiple ascending dose
**MEN 2**	multiple endocrine neoplasia syndrome type 2
**MRI**	magnetic resonance imaging
**MTC**	medullary thyroid carcinoma
**NPAL**	not highly palatable
**open label**	A study in which there are no restrictions on knowledge of treatment allocation, therefore the investigator and the study participant are aware of the drug therapy received during the study.
**PAL**	Highly palatable
**PD**	pharmacodynamic
**PHQ-9**	Patient Health Questionnaire-9
**PFS**	pre-filled syringe
**PG**	plasma glucose

**PK**	pharmacokinetic
**QD**	once daily
**QW**	once weekly
**randomize**	the process of assigning subjects/patients to an experimental group on a random basis
**RFPM**	Remote Food Photography Method©
**SAD**	single ascending dose
**SAE**	serious adverse event
**SAP**	statistical analysis plan
**SC**	subcutaneous(ly)
**screen**	The act of determining if an individual meets minimum requirements to become part of a pool of potential candidates for participation in a clinical study.
**SUSAR**	suspected unexpected serious adverse reaction
**T2DM**	type 2 diabetes mellitus
**TBL**	total bilirubin
**TE-ADA**	treatment-emergent anti-drug antibody
**TEAE**	treatment-emergent adverse event: Any untoward medical occurrence that emerges during a defined treatment period, having been absent pretreatment, or worsens relative to the pretreatment state, and does not necessarily have to have a causal relationship with this treatment
**ULN**	upper limit of normal
**VAS**	visual analog scale
**WOCBP**	women of childbearing potential

Appendix 2. Clinical Laboratory Tests

Safety Laboratory Tests

**Hematology**	**Clinical Chemistry**
Hematocrit	Sodium
Hemoglobin	Potassium
Erythrocyte count (RBC)	Bicarbonate
Mean cell volume	Chloride
Mean cell hemoglobin	Calcium
Mean cell hemoglobin concentration	Phosphorus
Leukocytes (WBC)	Glucose (fasting)[a]
Absolute counts of	Blood urea nitrogen
Neutrophils	Uric acid
Lymphocytes	Total protein
Monocytes	Albumin
Eosinophils	Total bilirubin
Basophils	Alkaline phosphatase
Platelets	Alanine aminotransferase
	Aspartate aminotransferase
**Urinalysis**	Creatinine
Specific gravity	Lipase (fasting)
pH	Amylase
Protein	Triglycerides (fasting)[a]
Glucose	Total cholesterol (fasting)
Ketones	Low-density lipoprotein (fasting)
Bilirubin	High-density lipoprotein (fasting)
Urobilinogen	Hemoglobin A1c
Blood	
Leukocytes	**Serology**[e]
Microscopy[b]	Hepatitis B surface antigen
	Hepatitis C antibody, hepatitis C RNA
	HIV antibody
**Endocrine**	
FSH[b, c]	Pregnancy test (urine, serum)[f]
Calcitonin[d]	Drug and alcohol screen[g]

Abbreviations: FSH = follicle-stimulating hormone; HIV = human immunodeficiency virus; RBC = red blood cells; WBC = white blood cells.

a At timepoints when glucose and triglyceride efficacy assessments coincide with safety laboratory tests, glucose and triglycerides will only be analyzed once.

b If clinically indicated, as per investigator's discretion.

c Female subjects only.

d At screening only

e At screening only (unless previously performed within the last 6 months with reports available for review). See exclusion criteria (Section 6.2) for further details.

f Performed for women of childbearing potential only. Serum pregnancy test is done at screening and follow-up and urine pregnancy test is performed on Days -4, 1, 15, and 36.

g Performed at screening. Procedures may be repeated throughout the study as deemed necessary by the investigator.

Appendix 3. Study Governance, Regulatory and Ethical Considerations

Informed Consent

The investigator is responsible for:

- ensuring that the subject understands the nature of the study, the potential risks and benefits of participating in the study, and that their participation is voluntary.
- ensuring that informed consent is given by each subject or legal representative. This includes obtaining the appropriate signatures and dates on the ICF prior to the performance of any protocol procedures and prior to the administration of investigational product.
- answering any questions the subject may have throughout the study and sharing in a timely manner any new information that may be relevant to the subject's willingness to continue his or her participation in the study.
- providing a copy of the ICF to the subject or the subject's legal representative and retaining a copy on file.

Recruitment

Lilly or its designee is responsible for the central recruitment strategy for patients. Individual investigators may have additional local requirements or processes. Study-specific recruitment material should be approved by Lilly.

Ethical Review

The investigator must give assurance that the institutional review board (IRB) was properly constituted and convened as required by ICH guidelines and other applicable laws and regulations.

Documentation of IRB approval of the protocol and the ICF must be provided to Lilly before the study may begin at the investigative site. Lilly or its representatives must approve the ICF before it is used at the investigative site. All ICFs must be compliant with the ICH guideline on GCP.

The study site's IRB should be provided with the following:

- the current IB and updates during the course of the study
- ICF
- relevant curricula vitae

Regulatory Considerations

This study will be conducted in accordance with the protocol and with:

1) consensus ethics principles derived from international ethics guidelines, including the Declaration of Helsinki and Council for International Organizations of Medical Sciences (CIOMS) International Ethical Guidelines
2) applicable ICH GCP Guidelines
3) applicable laws and regulations

Some of the obligations of the sponsor will be assigned to a third-party organization.

Protocol Signatures

The sponsor's responsible medical officer will approve the protocol, confirming that, to the best of his or her knowledge, the protocol accurately describes the planned design and conduct of the study.

After reading the protocol, the principal investigator will sign the protocol signature page and send a copy of the signed page to a Lilly representative.

Final Report Signature

The investigator or designee will sign the clinical study report for this study, indicating agreement with the analyses, results, and conclusions of the report.

The sponsor's responsible medical officer and statistician will sign/approve the final clinical study report for this study, confirming that, to the best of his or her knowledge, the report accurately describes the conduct and results of the study.

Data Quality Assurance

To ensure accurate, complete, and reliable data, Lilly or its representatives will do the following:

- provide instructional material to the study site, as appropriate.
- provide training to instruct the investigators and study coordinators. This training will give instruction on the protocol, the completion of the eCRFs, and study procedures.
- make periodic visits to the study site.
- be available for consultation and stay in contact with the study site personnel by mail, telephone, and/or fax.
- review and evaluate eCRF data and/or use standard computer edits to detect errors in data collection.
- conduct a quality review of the database.

In addition, Lilly or its representatives will periodically check a sample of the subject data recorded against source documents at the study site. The study may be audited by Lilly and/or regulatory agencies at any time. Investigators will be given notice before an audit occurs.

The investigator will keep records of all original source data. This might include laboratory tests, medical records, and clinical notes. If requested, the investigator will provide the sponsor, applicable regulatory agencies, and applicable IRBs with direct access to the original source documents.

Data Collection Tools/Source Data

An electronic data capture system will be used in this study. The site must define and retain all source records and must maintain a record of any data where source data are directly entered into the data capture system.

Data Protection

Data systems used for the study will have controls and requirements in accordance with local data protection law.

The purpose and use of subject personal information collected will be provided in a written document to the subject by the sponsor.

Study and Site Closure

Discontinuation of Study Site

Study site participation may be discontinued if Lilly or its designee, the investigator, or the IRB of the study site judges it necessary for medical, safety, regulatory, or other reasons consistent with applicable laws, regulations, and GCP.

Discontinuation of the Study

The study will be discontinued if Lilly or its designee judges it necessary for medical, safety, regulatory, or other reasons consistent with applicable laws, regulations, and GCP.

Appendix 4. Blood Sampling Summary

This table summarizes the approximate number of venipunctures and estimated blood volumes for all blood sampling (screening, safety laboratories, and bioanalytical assays) during the study.

Protocol I8F-MC-GPHH Sampling Summary

Purpose	Blood Volume per Sample (mL)	Number of Blood Samples	Total Volume (mL)
Screening tests[a,b]	24	1	24
Safety laboratory tests[a,b]	22.5	4	90
Tirzepatide PK	3	5 (+3)	24
Pharmacodynamics			
Ghrelin (fasting)[c]	2	3	6
Glucagon (fasting)[c]	2	3	6
Glucose (fasting) – Weeks 3 and 6[c]	2	2	4
Triglycerides (fasting) – Weeks 3 and 6[c]	2.5	2	5
Insulin (fasting)[c]	2.5	3	7.5
Insulin (postprandial)[c]	2.5	3	7.5
Peptide YY (postprandial)[c]	5	3	15
Pancreatic polypeptide (postprandial)[c]	5	3	15
Leptin (postprandial)[c]	4	3	12
Active GIP and GLP-1 (postprandial)[c]	2	3	6
Amylin (postprandial)[c]	5	3	15
Immunogenicity	10	4 (+3)	70
Pharmacogenetics	10	1	10
Nonpharmacogenetic stored sample[c] (fasted)	11	3	33
Total			350
Total for clinical purposes [rounded up to nearest 10 mL]			350

Abbreviations: GIP = glucose-dependent insulinotropic polypeptide; GLP-1 = glucagon-like peptide 1; PK = pharmacokinetics.

a additional samples may be drawn if needed for safety purposes.

b performed on site or in a local laboratory.

c performed at a central or referral laboratory, including for storage.

Appendix 5. Pharmacodynamic Assessments

5.1. Ad Libitum Food Intake Test

Food intake will be quantified in a clinical setting.

5.1.1. Clinic-Based Food Intake Test

Food intake will be quantified via test meals conducted in the CRU at lunch during the 6-week treatment period (Baseline, Day -4 to Day -1), Day 16 [Week 3, Visit 6], and Day 37 [Week 6, Visit 11]), and during the third and fourth weeks of the follow-up period i.e. [Visit 12] and [Visit 13], respectively. The start time of the lunch test meals will be consistent within subjects across testing occasions. Start and end times of the test meals will be recorded.

Subjects will complete a test lunch that begins between approximately 11:30 AM and 12:30 PM.

Lunch will consist of buffet meal (Table GPHH.3).

Table GPHH.3. Details of Buffet Meal

baked potato chips	grilled chicken bites	barbeque sauce
baked tortilla chips	popcorn chicken bites	sweet and sour sauce
corn chips	cheddar cheese	M&M'S®
pretzel twists	swiss cheese	crème-filled chocolate cake rolls
buttered popcorn	salsa	raisins
mixed nuts with salt	fat-free ranch dressing	fruit punch
baby carrots	cheese dip	water

Subjects will be instructed that they can eat as much or as little as they wish during the test meals and that they should alert a staff member when they are finished eating, at which time the subjects will leave the test meal. Staff members will check on the subjects after 25 minutes and terminate the session after 40 minutes if the subject does not end the session.

Food intake will be quantified by precisely weighing foods before and after the test meals, as close to the beginning and end of the meal as possible. During the follow-up period, the time the food is weighed before and after the test meals will be recorded. Energy and nutrient intake will be calculated by linking each food to a match in the United States Department of Agriculture (USDA) Food and Nutrient Database for Dietary Studies (FNDDS) Studies (USDA 2018) or data from the food producer/manufacturer.

5.1.2. Free-Living Food Intake

The time of meals will be assessed for approximately 4 days during the last week of follow-up period (beginning approximately 3 weeks post-final dose of study drug). During the follow-up period, energy and gram intake will not be calculated, rather, the start and stop times of the meal will be quantified based on the timestamps of the food images. These data will be collected with the Remote Food Photography Method© (RFPM) (Martin et al. 2009, 2013) and SmartIntake®

smartphone application (or app). The RFPM and SmartIntake® app were developed by Corby Martin, Ph.D., H. Raymond Allen, Ph.D., and colleagues at the Pennington Biomedical Research Center. RFPM data are collected with the SmartIntake® app, which is used to streamline data collection and minimize subject burden.

Self-report methods, such as pen-and-paper and dietary recall, rely on the ability of the subject to accurately recall the types and portion sizes of foods consumed, and the accuracy of these methods have been questioned (Beaton et al. 1997; Tran et al. 2000; Schoeller et al. 1990; Bandini et al. 1990). Importantly, ~50% of the error in self-report methods is due to subjects' inability to accurately estimate portion size (Beasley et al. 2005), a limitation that is avoided when using the RFPM and SmartIntake® app.

The RFPM and SmartIntake® app also result in the collection of data in real-time with food image data which can be evaluated via a password protected website for quality and completeness; thus, providing a platform to reduce missing data and facilitate data quality.

When using the SmartIntake® app, subjects place a reference card next to their food and capture images of their food selection and plate waste. The user identifies foods that are not easily identified by wrappers or containers by typing a food description into a text box. Drop-down boxes also allow the user to indicate what meal or snack is being consumed, and also where the food was obtained (e.g., home prepared, restaurant) and where it is being eaten. These data and food images are automatically and wirelessly sent by the app to the server-based Food Photography Application©, which is used to manage the data collection process and analyze the food images to estimate energy and nutrient intake.

To facilitate data quality and completeness, the SmartIntake® app includes Ecological Momentary Assessment (EMA) methodology (Stone et al. 1994) to remind subjects to capture images of the foods and beverages that they consume. These reminders are text messages that are scheduled for delivery at the personalized meal-times of the subjects. The responses to EMAs are tracked in near real time, which allows the research team to quickly identify when data collection problems occur. In such cases, a back-up method is used (e.g., a food record or food recall conducted by phone). The content of the text reminders can be customized by the researcher or clinician.

The SmartIntake® app sends subjects' food images and accompanying food descriptions to a Pennington server where the Pennington team analyzes the images to estimate food intake if needed. The analysis process relies on the Food Photography Application©, which allows the operator to identify a match for each food from the Food and Nutrient Database for Dietary Studies (USDA 2018) and other sources, such as manufacturer's information and Nutrition Fact Panels, to calculate energy and nutrient intake. Additionally, the operator uses the program to estimate portion size by visually comparing subjects' food images to images of foods with a known portion size (i.e., standard portion images). This process relies on existing and validated methodology (Martin et al. 2012, Martin et al. 2009, Williamson et al. 2004; Williamson et al. 2003) to estimate food selected and plate waste, which is used to calculate food intake by difference. The RFPM has been found to accurately measure the energy and nutrient intake of

adults, with error of 3.7% over 6 days in free-living conditions compared to the gold standard, doubly labeled water.

During the current study, subjects will be trained how to use the RFPM and SmartIntake® app during their visit to the center to capture imaging data on Day 59. All food consumption during the observation period should be recorded and subjects will receive regular reminders and extensive review of their data in near real time to foster data quality and completeness. These procedures will facilitate data collection as a formal run-in is not being conducted before data collection begins. Subjects will be instructed to photograph their food as closely as possible to the actual meal start and end.

5.2 Magnetic Resonance Imaging

Functional neuroimaging, in the forms of BOLD fMRI will be used to assess brain response patterns to food and non-food images. The MRI scanning will occur on different scanners such as a 3T General Electric Discovery 750W with 32-channel head coil, a 3T General Electric Discovery 750 with 32-channel head coil, a 3T Siemens Prisma with a 64-channel head coil, or a 3T Philips Achieva with a 32-channel head coil. Functional neuroimaging session will be conducted after participants fast overnight for at least 8 hours (ten Kulve et al. 2016).

Subjects will be required to complete the food image task during the BOLD fMRI scan. The food image task was developed using images from the Food-pic database (Blechert et al. 2014). Briefly, 90 food images across sweet and savory tastes, high- and low-energy density, and varied macronutrient composition, were included along with 15 images of non-food objects (e.g. everyday household objects). This results in 105 images in total. Macronutrient categories (Geiselman et al. 1998) were made by categorizing each food as:

- low versus high fat (LF vs HF, <30% vs >30%)
- low versus high sugar (LS vs. HS, <30% vs. >30%)
- low versus high carbohydrate (LCCHO vs HCCHO, <30% vs >30%)
- low versus high protein (LP vs HP, <13% vs >13%)

This resulted in 6 food categories with 15 photos in each:

1. HF/HS
2. HF/HCCHO
3. HF/LCCHO/HP
4. HS/LF
5. LF/HCCHO
6. LF/LCCHO/HP

Each 600 × 450 color photo shows 1 food on a white background.

Among the 6 food categories, HF-HS and HF-HCCHO will be called highly palatable (PAL) food and LF-LCCHO-HP will be called not highly palatable (NPAL) food.

In each trial of the task, 1 food photo is displayed for 5 seconds followed by 0.5 seconds of a fixation crosshair. Next, the same image scaled to 80% of the original size is shown with the words “How much do you want to eat this?” for 2.5 seconds. A slider bar with “Not at all” and “Want very much” on the left and right ends appears. The subject moves a joystick to select a slider bar position and clicks a joystick button to respond. A fixation crosshair displays for a minimum of 1.5 seconds before the next trial. There are 15 trials (i.e. 15 photos) per block and 14 inter-trial intervals per block: an average of 150 s/block. This is followed by a 30 s inter-block interval before the next block. total of 7 blocks and 6 inter-block intervals: 20.5 minutes of recorded data. Prior to the beginning of the task, subjects will be given time to practice the task in the scanner. Additionally, a high-resolution structural scan will be acquired. The total duration of each MRI session will be around 40 minutes.

For the purposes of evaluating inter-scanner variability to ensure that the fMRI data from all sites can be analyzed collectively at the end of the study, subjects will be required to complete a bilateral hand squeeze motor task during the BOLD fMRI scan, consisting of 4 blocks after the food image task. Each block will consist of 30 seconds of bilateral hand squeezing followed by 30 seconds of rest (around 3 minutes total run time).

To model respiratory and cardiac effects on the BOLD signals, participants will be fitted with a lap belt and pulse oximeter. The respiratory cycle signal and cardiac cycle signal will be regressed out of all time series data.

BOLD fMRI Data Processing

The BOLD fMRI acquisition parameters include repetition time of 3000 ms, echo time of 30 ms, flip angle of 90°, slice thickness of 3.5 mm, and 64×64 image matrix.

As in prior work (King et al. 2018), a T1-weighted magnetization-prepared 180° radio-frequency pulses and rapid gradient-echo (MP RAGE) will be collected as an anatomical reference for functional data.

Preprocessing of fMRI data will be done by an external imaging laboratory services provider.

For the hand squeezing fMRI task, BOLD contrasts of hand squeezing versus resting within bilateral somatomotor ROIs will be calculated. For the food image fMRI task, highly palatable food (HF-HS, HF-HCCHO), not highly palatable foods (LF-LCCHO-HP), and non-food object blocks will be modeled. To assess brain activation related to viewing food pictures, the contrast between highly palatable foods and non-food objects will be computed. Additionally, the contrast between highly palatable foods and not highly palatable foods as well as the contrast between not highly palatable foods and non-food objects may be computed. Contrasts between each specific food category and non-food objects may also be computed if deemed necessary. Average BOLD contrast in each of 9 areas associated to brain reward will be reported (insula, medial frontal gyrus, superior temporal gyrus, precentral gyrus, cingulate gyrus, hippocampus, putamen, orbitofrontal cortex, and ventral striatum).

5.3. Appetite Visual Analog Scales

The aim of the appetite visual analog scale (VAS) is to determine the effects of study treatments on appetite sensations and desire for specific foods. Two types of VAS will be used to assess subjective ratings of appetite and affect; laboratory-based and retrospective.

5.3.1. Appetite Visual Analog Scale

During the laboratory-based test meals on (Baseline, Day -4 to Day -1), Day 16 [Week 3, Visit 6], and Day 37 [Week 6, Visit 11]), subjects will complete VAS ratings immediately before and after the test lunch.

Subjects will be asked to rate their feelings of hunger, satiety, fullness, prospective food consumption, and desire for specific foods on a 10-cm (100-mm) line, or a 100-unit line represented on a piece of paper; anchored by verbal descriptors, usually "not at all" and "extremely." The ratings will include the following 8 questions (Flint et al. 2000):

- How hungry do you feel right now?
- How satisfied do you feel right now?
- How full do you feel right now?
- How much food do you think you could eat right now?
- Would you like to eat something sweet?
- Would you like to eat something salty?
- Would you like to eat something savory?
- Would you like to eat something fatty?

Pen-and-paper VAS will be used and will include a 10-cm line. Subjects will record their rating on the respective lines, ranging from 0 ("not at all") to 100 ("extremely"). Only provided or printed (not copied) paper VAS can be used, as copying them alters the length line.

Overall appetite score is calculated as described in Section 10.3.3.1 (Pharmacodynamic Parameter Estimation).

5.3.2. Retrospective Appetite Visual Analog Scale

Visual analog score will be used to measure average ratings of appetite that subjects experienced over the past week. These ratings will occur on Baseline, Day -4 to Day -1, Day 16 [Week 3, Visit 6], and Day 37 [Week 6, Visit 11]).

The retrospective method of collecting VAS data has been found to be consistent with daily assessments of satiety (Womble et al. 2003). These VAS items will assess 4 appetite-related metrics and the instructions to the subjects will be modified, as detailed here:

- How hungry did you feel over the past week?

- How satisfied did you feel over the past week?
- How full did you feel over the past week?
- How much food did you think you could eat over the past week?

In addition to the 4 appetite metrics noted above, retrospective VAS will also be used to quantify feelings of nausea, malaise, and gastrointestinal distress over the previous week. Specifically, the following items will be administered:

- How much nausea did you have over the past week?
- How much malaise did you have over the past week?
- How much gastrointestinal distress did you have over the past week?

These 3 items will be anchored with verbal descriptors ranging from "none at all" to "an extreme amount".

5.4. Food Intake-Related Questionnaires

Subjects' attitudes towards food will be evaluated with the Food Craving Inventory (FCI), Food Craving Questionnaire-State (FCQ-S), Eating Inventory, and Power of Food Scale. These food-related questionnaires will be administered at Day -4 to Day -1 [baseline], Day 16 [Week 3, Visit 6], and Day 37 [Week 6, Visit 11]). Attempts will be made for questionnaires to be completed in a fasted state shortly after arriving to the study site.

5.4.1. Food Craving Inventory

The 33-item FCI (White et al. 2002) will be used to measure cravings for specific food groups. The measure consists of 5 empirically derived factors:

- high fats
- sweets
- carbohydrates/starches
- fast food fats
- and fruits and vegetables

The FCI is scaled in a frequency format assessing the frequency of cravings for particular foods over the past month. All items are scored in the following manner: Never = 1, Rarely = 2, Sometimes = 3, Often = 4, Always = 5.

The instructions for the FCI will be altered for Days 16 and 37 (Week 3, Visit 6 and Week 6, Visit 11) to include the following time frame: "Since the last time you completed this questionnaire, how often have you experienced a craving for the food?". This differs from the original instructions which denotes a 1-month time frame. All other instructions on the FCI will remain the same as the original.

5.4.2. Food Craving Questionnaire-State

The FCQ-S is a 15-item measure that assesses the strength of state food cravings at the moment of administration. The FCQ-S has demonstrated good validity and internal consistency in non-clinical populations at the moment of provision (Cepeda-Benito et al. 2000; Moreno et al. 2008).

Desire, anticipation positive, anticipation negative, lack of control, and hunger scores are measured. Greater scores for each sub-scale denote higher levels of craving.

5.4.3. Eating Inventory

The Eating Inventory is a 51-item validated questionnaire that assesses 3 eating-related constructs:

- dietary restraint
- disinhibition
- perceived hunger (Stunkard and Messick. 1985; Sunkard and Messick 1988)

Dietary restraint refers to the intent and ability to restrict food intake, disinhibition measures the tendency to overeat, and hunger measures susceptibility to feelings of hunger. Scores for restraint, disinhibition, and hunger range from 0 to 21, 0 to 18, and 0 to 14, respectively, and a greater number indicates greater levels of each respective eating component.

5.4.4. Power of Food Scale

The Power of Food Scale measures appetite for palatable foods in the environment at the following 3 levels of food proximity: food available, food present, and food tasted. The Power of Food Scale has adequate internal consistency and test-retest reliability (Lowe et al. 2009).

5.5. Impulsivity

Impulsivity will be measured with the Barratt Impulsiveness Scale (BIS) on Day -4 to Day -1 [baseline], Day 16 [Week 3, Visit 6], and Day 37 [Week 6, Visit 11]). Attempts will be made for BIS questionnaire to be completed in a fasted state shortly after arriving to the study site.

5.5.1. Barratt Impulsiveness Scale

The BIS is a 30-item self-report measure describing impulsive or non-impulsive behaviors and preferences. The scale consists of 6 first order factors as follows:

- attention
- cognitive stability
- motor
- perseverance
- self-control

- cognitive complexity

The scale also consists of 3 second-order factors: attentional, motor, and non-planning.

The items are rated on a 4-point scale with responses ranging from 1 = rarely/never to 4 = almost always/always. The measure assesses the personality/behavioral construct of impulsiveness and has demonstrated good psychometric properties in multiple populations (Patton et al. 1995; Reid et al. 2014).

Appendix 6. Hepatic Monitoring Tests for Treatment-Emergent Abnormality

Evaluation of subjects with treatment-emergent abnormal hepatic biochemical tests during a clinical trial. Extent and type of work-up may vary by subject's history, severity of liver injury, underlying disease, and geography.

Recommended Evaluation	Competing Causes of Abnormal Liver Tests
**1st Line Testing**	
ALT, AST, ALP, TBL, direct bilirubin, GGT, CK	Routine follow up
Thorough history of symptoms, co-existing medical conditions, concomitant medications, dietary and nutritional supplements, excessive exercise or muscle injury, alcohol consumption, illicit substances.	Systemic infection/ sepsis; ischemic/ congestive hepatic injury; gallstone disease; alcoholic liver disease; muscle injury/ rhabdomyolysis; acetaminophen toxicity; DILI due to another drug, herbal or dietary supplement.
Serum CK	Muscle injury/rhabdomyolysis[a]
Anti-HAV (IgM)	Acute HAV infection
HBsAg<br>Anti-HBc IgG, IgM	Acute hepatitis B; exacerbation of chronic hepatitis B
Anti-HCV<br>HCV RNA (PCR)[b]	Acute hepatitis C[c]; exacerbation of chronic hepatitis C
Anti-HEV (IgG, IgM), HEV RNA[d]	Acute hepatitis E
ANA, ASMA, quantitative immunoglobulins (IgG, IgM, IgA)	Autoimmune hepatitis[e]
Hepatobiliary imaging<br>(ultrasonography, CT scan, MRI, MRCP)[f]	Biliary obstruction; pancreatitis; gallstones; portal-vein/ hepatic vein thrombosis; hepatic metastasis
**2nd Line Testing**	
PT-INR	For patients with elevated TBL or suspected liver failure
Serological tests for EBV, CMV, HSV<br>May need to obtain acute and convalescent serological tests	Hepatic injury caused by CMV, EBV, HSV
EBV-DNA, CMV- DNA, HSV-DNA by PCR.<br>Liver biopsy needed to confirm HSV	Hepatic injury caused by CMV, EBV, HSV
**Additional Tests[g]**	
Bone specific ALP (ALP fractionation)	Differentiate bone from liver origin
LKM-1 antibody	Autoimmune hepatitis
Urinary ethylglucuronide[h],<br>blood phosphatidylethanol[i]	Alcoholic liver disease
Serum acetaminophen level;<br>acetaminophen protein adducts	Acetaminophen toxicity
Review of blood pressure, pulse, electrocardiogram, echocardiogram, cardiology consult	Ischemic or congestive hepatic injury

Urine toxicology screen	Hepatotoxicity due to cocaine, opiates and other illicit substances
Anti-HDV	Hepatitis D
Blood or urine cultures	Systemic infection, sepsis
Serum ceruloplasmin, serum copper Slit lamp eyes examination for Kayser-Fleischer rings, genetic testing	Wilson's disease

Abbreviations: ALP = alkaline phosphatase; ALT = alanine aminotransferase; ANA = antinuclear antibody; anti- = antibody; ASMA = anti-smooth muscle antibody; AST = aspartate aminotransferase; CK = creatinine kinase; CMV = cytomegalovirus; CT = computed tomography; DILI = drug-induced liver injury; EBV = Epstein Bar Virus; GGT = gamma-glutamyl transferase; HAV = hepatitis A virus; HBc = hepatitis B core; HBsAg = hepatitis B surface antigen; HBV = hepatitis B virus; HCV = hepatitis C virus; HDV = hepatitis D virus; HEV = hepatitis E virus; HSV = herpes simplex virus; Ig = immunoglobulin; INR = international normalized ratio; LKM-1 = liver kidney microsomal type 1; MRCP = magnetic resonance cholangiopancreatography; MRI = magnetic resonance imaging; PCR = polymerase chain reaction; PT-INR = prothrombin time-international normalized ratio; RNA = ribonucleic acid; TBL = total bilirubin.

[a] Serum AST typically (although not always) is higher than ALT

[b] If anti-HCV positive, HCV RNA is required to confirm HCV infection

[c] Acute hepatitis C may be anti-HCV negative but HCV RNA positive

[d] If anti-HEV IgM positive, consider confirmation with HEV RNA by nested PCR

[e] A liver biopsy is needed to confirm a diagnosis of autoimmune hepatitis

[f] If cholestatic injury, MRCP may be recommended

[g] Based on medical history and clinical judgment

[h] Alcohol consumption in past 3 to 5 days

[i] Alcohol consumption in past 3 weeks

Appendix 7. Pancreatic Monitoring

Pancreatic Enzymes: Safety Monitoring Algorithm for Subjects/Patients without Symptoms of Pancreatitis[1,2]

Follow this algorithm when the value(s) for serum lipase and/or amylase are ≥ 3X ULN.

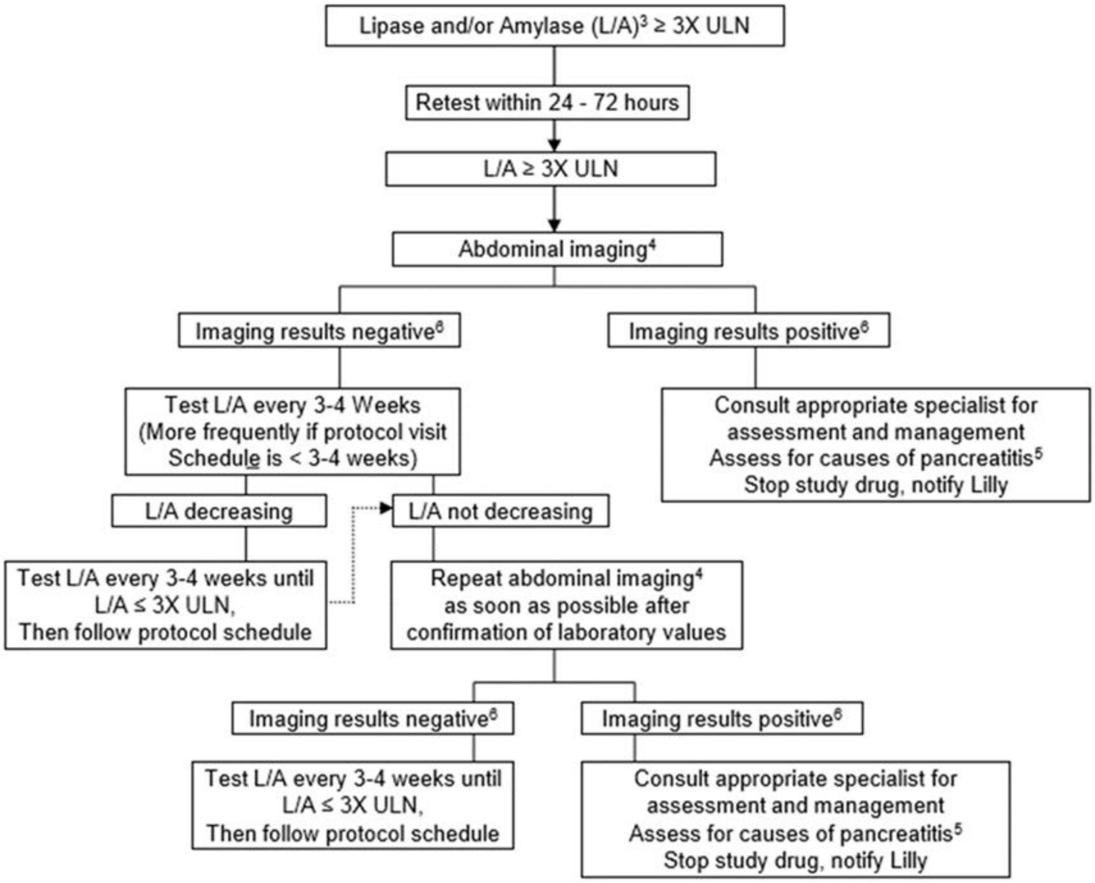

1. Symptomatic – related primarily to abdominal pain consistent with pancreatitis; however, severe nausea, vomiting and other symptoms may be considered by the investigator as symptomatic as well.

2. If, at any time, in the opinion of the investigator, patient/subject has symptoms of acute pancreatitis irrespective of L/A results:
   (a) Consult appropriate specialist for assessment and management
   (b) Assess for causes of pancreatitis
   (c) Stop study drug
   (d) Notify Lilly

3. L/A = Lipase and/or amylase. Either or both enzymes can be measured and either or both can be used to meet the algorithm criteria.

4. Abdominal imaging is most valuable when performed at the time of elevated enzyme values. If in the opinion of the radiologist or investigator, it is safe for the patient/subject to receive contrast, an enhanced abdominal CT is preferred. MRI is also an acceptable imaging modality.

5. As minimum, test hepatic analytes, triglycerides, and calcium, and record all concomitant medications

6. Imaging results positive or negative for signs of acute pancreatitis

Abbreviations: CT = computed tomography; L/A = lipase and/or amylase; MRI = magnetic resonance imaging; ULN = upper limit of normal.

Subjects diagnosed with pancreatitis will be discontinued from the study. Investigators will be responsible for following, through an appropriate healthcare option, these pancreatitis AEs until the events resolve or are explained. Adverse events that meet the diagnostic criteria of acute pancreatitis will be captured as serious adverse events (SAEs). For all other pancreatic AEs (such as idiopathic or asymptomatic pancreatic enzyme abnormalities), the investigator will be responsible for determining the seriousness of the event (AE or SAE) and the relatedness of the event to investigational product.

Appendix 8. Recommended Laboratory Testing for Hypersensitivity Events

Lab testing should be performed at the time of a Systemic Hypersensitivity Event. Important information about why, when, and what to test for are provided below. The management of the adverse event may warrant lab testing beyond that described below and should be performed as clinically indicated.

Laboratory testing during a Systemic Hypersensitivity Event is not performed for diagnostic purposes. Its intent is several fold:

- To help characterize and classify systemic hypersensitivity reactions
- To meet regulatory expectations
- To improve subsequent clinical management by helping to distinguish between the various mechanistic bases of anaphylaxis

When Should Labs Be Obtained?

- In the presence of generalized urticaria or if anaphylaxis is suspected
- After the subject has been stabilized, obtain a sample within 1 to 2 hours of the event; however, samples may be obtained as late as 12 hours after the event as analytes can remain altered for an extended period of time. Record the time at which the sample was collected.
- Obtain a follow-up sample at the next regularly scheduled visit or after 4 weeks, whichever is later.

What Labs* Should Be Obtained?

- Tryptase**
- ADA and tirzepatide concentration (PK)
    - ADA testing should include drug-specific immunoglobulin (Ig)E or the basophil activation test (BAT)[#]. These tests are not routinely available and need to be developed for individual molecules based on their evolving safety profile. Samples are collected, and testing conducted once the assay is available, as appropriate. Please consult an immunologist within GPS for further guidance.
- Complement
    - C3a and C5a

- Cytokines
    - IL-6, IL-1β, IL-10 (or any cytokine panel that includes these 3 cytokines)

* These labs are bundled in the Clinical Laboratory Operations Hypersensitivity Lab Testing Kit.

** If a tryptase sample is obtained more than 2 hours after the event (i.e., within 2 to 12 hours), or is not obtained because more than 12 hours have lapsed since the event, obtain urine for *N*-methylhistamine (NMH) testing. Note that for tryptase serum samples obtained within 2 to 12 hours of the event, urine NMH testing is performed in addition to tryptase testing. Collect the first void urine following the event. Obtain a follow-up urine for NMH testing at the next regularly scheduled visit or after 4 weeks, whichever is later.

[#] The BAT is an in vitro cell based assay that only requires a serum sample. It is a surrogate assay for drug specific IgE but is not specific for IgE.

Appendix 9. Protocol Amendment I8F-MC-GPHH(f) Summary - Effect of Tirzepatide on Energy Intake and Appetite- and Reward-Related Brain Areas in Overweight/Obese Subjects: A Placebo-Controlled 6-Week Study with Functional MRI

Overview

Protocol I8F-MC-GPHH, Effect of Tirzepatide on Energy Intake and Appetite- and Reward-Related Brain Areas in Overweight/Obese Subjects: A Placebo-Controlled 6-Week Study with Functional MRI, has been amended. The new protocol is indicated by Amendment (f) and will be used to conduct the study in place of any preceding version of the protocol.

This amendment is considered a nonsubstantial protocol amendment.

The overall changes and rationale for the changes made to this protocol are as follows:

- Appendix 5 was updated to include language on
  - the types of scanners on which MRI scanning will occur to specify the different scanners used across the different study sites and,
  - the hand squeezing task following the food image task to evaluate inter-scanner variability across the different scanners. The hand squeezing task produces consistent patterns of motor cortex activation in the brain, independent of scanner type.
    - The hand squeezing task will be used to ensure that the fMRI data from all sites can be analyzed collectively at the end of the study.
  - The description of preprocessing of fMRI data was deleted because it will be performed by an external imaging laboratory services provider.

Revised Protocol Sections

**Note:**	All deletions have been identified by ~~strikethroughs~~.<br>All additions have been identified by the use of underscore.

Appendix 5

5.2 Magnetic Resonance Imaging

Functional neuroimaging, in the forms of BOLD fMRI will be used to assess brain response patterns to food and non-food images. The MRI scanning will occur on different scanners such as a 3T General Electric Discovery 750W with 32-channel head coil, a 3T General Electric Discovery 750 with 32-channel head coil, a 3T Siemens Prisma with a 64-channel head coil, or a 3T Philips Achieva with a 32-channel head coil. Functional neuroimaging session will be conducted after participants fast overnight for at least 8 hours (ten Kulve et al. 2016).

Subjects will be required to complete the food image task during the BOLD fMRI scan. The food image task was developed using images from the Food-pic database (Blechert et al. 2014). Briefly, 90 food images across sweet and savory tastes, high- and low-energy density, and varied macronutrient composition, were included along with 15 images of non-food objects (e.g. everyday household objects). This results in 105 images in total.

For the purposes of evaluating inter-scanner variability to ensure that the fMRI data from all sites can be analyzed collectively at the end of the study, subjects will be required to complete a bilateral hand squeeze motor task during the BOLD fMRI scan, consisting of 4 blocks after the food image task. Each block will consist of 30 seconds of bilateral hand squeezing followed by 30 seconds of rest (around 3 minutes total run time).

BOLD fMRI Data Processing

The BOLD fMRI acquisition parameters include repetition time of 3000 ms, echo time of 30 ms, flip angle of 90°, slice thickness of 3.5 mm, and 64×64 image matrix.

As in prior work (King et al. 2018), a T1-weighted magnetization-prepared 180° radio-frequency pulses and rapid gradient-echo (MP RAGE) will be collected as an anatomical reference for functional data.

Preprocessing of fMRI data will be done by an external imaging laboratory services provider. ~~in SPM8 includes slice-timing correction, head-motion correction, smoothing, and warping to a standard coordinate frame. Time points with head rotation greater than 2° or translation greater than 2 mm will be removed. Data will be entered into a first-level voxel-wise analysis with each trial modeled as a boxcar function that covers the period of time when the large image is viewed. The boxcar function is convolved with the canonical hemodynamic response function. Functional scans will be analyzed in the context of the general linear model.~~

For the hand squeezing fMRI task, BOLD contrasts of hand squeezing versus resting within bilateral somatomotor ROIs will be calculated. For the food image fMRI task, h~~H~~ighly palatable food (HF-HS, HF-HCCHO), not highly palatable foods (~~low fat-low carbohydrate-high protein~~LF-LCCHO-HP), and non-food object blocks will be modeled. To assess brain activation related to viewing food pictures, the contrast between highly palatable foods and non-food objects will be computed. Additionally, the contrast between highly palatable foods and not highly palatable foods as well as the contrast between not highly palatable foods and non-food objects may be computed. Contrasts between each specific food category and non-food objects

may also be computed if deemed necessary. Average BOLD contrast in each of 9 areas associated to brain reward will be reported (insula, medial frontal gyrus, superior temporal gyrus, precentral gyrus, cingulate gyrus, hippocampus, putamen, orbitofrontal cortex, and ventral striatum).

Leo Document ID = a44bff92-a78c-493a-b066-c0ef15232beb

Approver: PPD

Approval Date & Time: 08-Oct-2021 12:53:16 GMT

Signature meaning: Approved

Approver: PPD

Approval Date & Time: 08-Oct-2021 15:32:30 GMT

Signature meaning: Approved

1. Statistical Analysis Plan I8F-MC-GPHH: Effect of Tirzepatide on Energy Intake and Appetite- and Reward-Related Brain Areas in Overweight/Obese Subjects: A Placebo-Controlled 6-Week Study with Functional MRI

Confidential Information

The information contained in this document is confidential and the information contained within it may not be reproduced or otherwise disseminated without the approval of Eli Lilly and Company or its subsidiaries.

Note to Regulatory Authorities: this document may contain protected personal data and/or commercially confidential information exempt from public disclosure. Eli Lilly and Company requests consultation regarding release/redaction prior to any public release. In the United States, this document is subject to Freedom of Information Act (FOIA) Exemption 4 and may not be reproduced or otherwise disseminated without the written approval of Eli Lilly and Company or its subsidiaries.

LY3298176 for Overweight/Obesity

Phase 1, multiple-center, randomized, partially blinded, placebo-controlled, parallel-arm study, with a positive control, liraglutide, in overweight/obese subjects to compare tirzepatide to placebo.

Eli Lilly and Company
Indianapolis, Indiana USA 46285
Protocol I8F-MC-GPHH
Phase 1

Document ID: VV-CLIN-109361

2. Table of Contents

Section **Page**

1. Statistical Analysis Plan I8F-MC-GPHH: Effect of Tirzepatide on Energy Intake and Appetite- and Reward-Related Brain Areas in Overweight/Obese Subjects: A Placebo-Controlled 6-Week Study with Functional MRI 1
2. Table of Contents 2
3. List of Abbreviations 7
4. Revision History 9
5. Study Objectives 10
6. Study Design 13
6.1. Study Design and Treatment 13
6.2. Determination of Sample Size 14
7. A Priori Statistical Methods 16
7.1. Populations for Analyses 16
7.2. General Considerations 16
7.3. Study Participant Characteristics 16
7.4. Study Participant Disposition 17
7.5. Concomitant Therapy 17
7.6. Treatment Exposure and Compliance 17
7.6.1. Treatment Exposure 17
7.6.2. Treatment Compliance 17
7.7. Important Protocol Deviations 18
7.8. Pharmacokinetic Analyses 18
7.8.1. Pharmacokinetic Parameter Estimation 18
7.8.2. Pharmacokinetic Statistical Inference 18
7.9. Pharmacodynamic Analyses 18
7.9.1. Pharmacodynamic Parameter Estimation 18
7.9.1.1. Food Intake (Ad-Libitum Food-Intake Test) 19
7.9.1.2. Central Reward and Appetite Circuits using BOLD fMRI 19
7.9.1.3. Appetite Visual Analog Scale and Retrospective Appetite Visual Analog Scale 20
7.9.1.4. Measures of Food Intake-Related and Impulsiveness Questionnaires 20
7.9.1.5. Hormones and Metabolites Related to Appetite Regulation 21
7.9.2. Pharmacodynamic Statistical Inference 22

7.9.2.1. Primary Pharmacodynamic Analysis 22
7.9.2.1.1. Energy Intake via Ad-libitum Food Intake Test 22
7.9.2.2. Secondary Pharmacodynamic Analyses 23
7.9.2.2.1. Central Reward and Appetite Circuits in the Fasting State using BOLD fMRI 23
7.9.2.2.2. Behavioral Appetite Assessments 24
7.9.2.2.2.1. Fasting and Postprandial Appetite VAS (Laboratory-Based) 25
7.9.2.2.2.2. Fasting FCI 26
7.9.2.2.2.3. FCQ-S 27
7.9.2.2.2.4. Eating Inventory through TFEQ 28
7.9.2.2.2.5. Power of Food Scale Questionnaires 29
7.9.2.2.2.6. Barratt Impulsiveness Scale 31
7.9.2.3. Exploratory Pharmacodynamic Analyses 33
7.10. Safety Analyses 36
7.10.1. Adverse Events 37
7.10.2. Treatment-Emergent Adverse Events 37
7.10.3. Serious Adverse Events 37
7.10.4. Adverse Events Leading to Discontinuation 37
7.10.5. Special Safety Topics 37
7.10.5.1. Hypoglycemia 37
7.10.5.2. Pancreatitis 38
7.10.5.3. Thyroid Malignancies and C-Cell Hyperplasia 38
7.10.5.4. Hypersensitivity Events 38
7.10.5.5. Injection-Site Reactions 39
7.10.5.6. Hepatobiliary Disorders 39
7.10.5.7. Severe/Serious Gastrointestinal Adverse Events 39
7.10.5.8. Acute Renal Events 39
7.10.6. Safety Laboratory Parameters 39
7.10.7. Vital Signs 40
7.10.8. Body Weight and Waist Circumference 40
7.10.9. Electrocardiogram 40
7.11. Immunogenicity 40
7.12. Interim Analyses 41
7.13. COVID-19 Impact Assessment 41
7.14. Exploratory Analysis for Motion Sensor Modeling 41
8. Unblinding Plan 42
9. Change from the Protocol-Specified Statistical Analyses 43

10. References 44
11. Appendices 45

Table of Contents

Tables **Page**

Table GPHH.5.1. Objectives and Endpoints....10
Table GPHH.7.1. Analysis Populations and Analysis Datasets....16
Table GPHH.7.2. Study Period and PD Measurements....18
Table GPHH.7.3. FCQ-S Subscore Category and Questions....27
Table GPHH.7.4. Scoring Algorithm for the TFEQ....29
Table GPHH.7.5. Power of Food Scale Subdomains and Questions....30
Table GPHH.7.6. Questions Categorized into First-Order Categories....32
Table GPHH.7.7. Questions Categorized into Second-Order Categories....32
Table GPHH.7.8. Definition of Hypoglycemic Event Categories....38
Table GPHH.7.9. Categorical Criteria for Treatment-Emergent Abnormal Blood Pressure and Pulse Measurements....40

Table of Contents

Figure **Page**

Figure GPHH.6.1. Illustration of study design for Protocol I8F-MC-GPHH. 15

3. List of Abbreviations

Term	Definition
**ADA**	anti-drug antibody
**BIS**	Barratt Impulsiveness Scale
**BMI**	body mass index
**BOLD fMRI**	blood-oxygenation-level-dependent functional magnetic resonance imaging
**AE**	adverse event
**AESI**	adverse event of special interest
**CI**	confidence interval
**COVID-19**	coronavirus disease 2019
**ECG**	electrocardiogram
**FCI**	Food Craving Inventory
**FCQ-S**	Food Craving Questionnaire-State
**GIP**	glucose-dependent insulinotropic polypeptide
**GLP-1**	glucagon-like peptide 1
**IPD**	important protocol deviation
**Lilly**	Eli Lilly and Company
**LSM**	least squares means
**MedDRA**	Medical Dictionary for Regulatory Activities
**MMRM**	mixed effects model of repeated measures
**MRI**	magnetic resonance imaging
**PD**	pharmacodynamic(s)
**PK**	pharmacokinetic(s)
**PT**	Preferred Term
**PYY**	peptide YY
**QD**	once daily
**QL**	quantification limit

Term	Definition
**QW**	once weekly
**ROI**	region of interest
**SAE**	serious adverse event
**SAP**	statistical analysis plan
**SOC**	System Organ Class
**TE-ADA(+)**	treatment-emergent anti-drug antibody (positive)
**TEAE**	treatment-emergent adverse event
**TFEQ**	Three-Factor Eating Questionnaire
**VAS**	visual analog scale

4. Revision History

Statistical analysis plan version 1 was approved on 25 June 2020 prior to the first patient visit.

Statistical analysis plan version 2 was approved on 09 June 2022 prior to database lock.

The major revisions in version 2 include

- modified study procedure and schedule of activities based on Protocol (f)
- updated sample size to approximately 111 subjects (37 subjects per treatment arm) planned to be randomly assigned, and
- reorganized and rephrased the PD analyses section.

Statistical analysis plan version 3 was approved prior to database lock.

Major revisions in version 3 include

- Change of specification of the BOLD fMRI analysis variables. Contrasts between conditions are defined as the mean difference as opposed to mean percent difference. This is because dividing by the non-food contrast was causing falsely large numbers in scans where the subject had low activation to non-food objects (that is, division by number close to 0).

5. Study Objectives

Table GPHH.5.1 shows the objectives and endpoints of the study.

Table GPHH.5.1. Objectives and Endpoints

Objectives	Endpoints
**Primary**<br>To compare the effect of 5-mg tirzepatide versus placebo, at Week 3, on energy intake in a clinical setting	• Change from baseline in energy intake (kcal) as assessed by ad-libitum food-intake test
**Secondary**<br>To compare the effect of 5-mg tirzepatide versus placebo, at Week 3 on	
• Parameters of central reward and appetite circuits in the fasting states using BOLD fMRI	• Change from baseline in BOLD fMRI activation to images of highly palatable food (high fat-high sugar and high fat-high carbohydrate) during the fasting state in the brain reward areas (insula, medial frontal gyrus, superior temporal gyrus, precentral gyrus, and cingulate gyrus)
• Parameters of behavioral appetite assessments	• Change from baseline in fasting and postprandial appetite VAS, and fasting FCI, FCQ-S, Eating Inventory, and Power of Food Scale questionnaires
**Exploratory**<br>To compare the effect of 5- or 10-mg tirzepatide versus placebo at Week 3 and/or Week 6 on	
• Parameters of central reward and appetite circuits in the fasting state using BOLD fMRI (Week 6/10 mg)	• Change from baseline in BOLD fMRI activation to images of highly palatable food (high fat-high sugar and high fat-high carbohydrate) versus non-food objects during the fasting state in the brain reward areas (insula, medial frontal gyrus, superior temporal gyrus, precentral gyrus, cingulate gyrus)
• Parameters of behavioral appetite assessments (Week 6/10 mg)	• Change from baseline in fasting and postprandial appetite VAS, and fasting FCI, FCQ-S, Eating Inventory, and Power of Food Scale questionnaires
• Energy intake in a clinical setting (Week 6/10 mg)	• Change from baseline in energy intake (kcal) as assessed by ad-libitum food-intake test

Objectives	Endpoints
• Parameters of central reward and appetite circuits in the fasting state using BOLD fMRI (Week 3/5 mg and Week 6/10 mg)<br><br>• Impulsivity (Week 3/5 mg and Week 6/10 mg)<br><br>• Appetite and metabolism regulating hormones (Week 3/5 mg and Week 6/10 mg)	• Change from baseline in BOLD fMRI activation to images of highly palatable food (high fat-high sugar and high fat-high carbohydrate) during fasting state in the hippocampus, putamen, orbitofrontal cortex, and ventral striatum brain regions<br><br>• Change from baseline in BIS questionnaire<br><br>• Change from baseline in ghrelin, glucagon, glucose, and triglycerides (fasting), insulin (fasting and postprandial), and amylin, GIP, GLP-1, leptin, pancreatic polypeptide, and PYY (postprandial)
To compare the effect of 5- and 10-mg tirzepatide versus liraglutide and tirzepatide versus placebo at Week 3 and/or Week 6 on<br>• Energy intake in a clinical setting (Week 3/5 mg and Week 6/10 mg)<br><br>• Parameters of behavioral appetite assessments (Week 3/5 mg and Week 6/10 mg)<br><br>• Parameters of central reward and appetite circuits in the fasting states using BOLD fMRI (Week 3/5 mg and Week 6/10 mg)<br><br>• Appetite and metabolism regulating hormones (Week 3/5 mg and Week 6/10 mg)	• Changes from baseline in energy intake (kcal) as assessed by ad-libitum food-intake test<br><br>• Change from baseline in fasting and postprandial appetite VAS, and the fasting FCI, FCQ-S, Eating Inventory, and Power of Food Scale questionnaires<br><br>• Change from baseline in BOLD fMRI activation to images of highly palatable food (high fat-high sugar and high fat-high carbohydrate during the fasting state in the brain reward areas [insula, medial frontal gyrus, superior temporal gyrus, precentral gyrus, cingulate gyrus, hippocampus, putamen, orbitofrontal cortex, and ventral striatum])<br><br>• Change from baseline in ghrelin, glucagon, glucose, and triglycerides (fasting), insulin (fasting and postprandial), and amylin, GIP, GLP-1, leptin, pancreatic polypeptide, and PYY (postprandial)

Objectives	Endpoints
To assess the safety and tolerability of tirzepatide.	• Adverse events<br>• Safety laboratory parameters<br>• Frequency of treatment-emergent anti-tirzepatide antibodies

Abbreviations: BIS = Barratt Impulsiveness Scale; BOLD = blood-oxygenation-level-dependent; FCI = Food Craving Inventory; FCQ-S = Food Craving Questionnaire-State; fMRI = functional MRI; GIP = glucose-dependent insulinotropic polypeptide; GLP-1 = glucagon-like peptide 1; MRI = magnetic resonance imaging; PYY = peptide YY; VAS = visual analog scale.

Approved on 20 Apr 2023 GMT

6. Study Design

6.1. Study Design and Treatment

This is a Phase 1, multicenter, randomized, partially blinded, placebo-controlled, parallel-arm study, with a positive control, liraglutide, in overweight/obese subjects. The primary objective of this study is to compare the effect of tirzepatide versus placebo on energy intake, as assessed by an ad-libitum food-intake test, at Week 3 of treatment at a therapeutic dose of 5 mg. Secondary objectives will compare these treatment groups for parameters of central reward, appetite circuits, and behavioral appetite assessments at Week 3.

Additional exploratory objectives will assess the effect of 10-mg tirzepatide versus placebo on energy intake, parameters of central reward and appetite, and behavioral appetite assessments at Week 6. Further exploratory analysis will assess the effect of tirzepatide versus liraglutide and liraglutide versus placebo on energy intake, parameters of central reward and appetite signaling, and behavioral appetite assessments at Week 3 and/or 6.

The study will consist of the following periods: approximately 5-week screening period, 5-day lead-in period, 6-week treatment period, and a 4-week safety follow-up period. Subjects will be randomized in a 1:1:1 ratio to placebo, tirzepatide, or liraglutide. Investigators and subjects will be blinded to tirzepatide and placebo treatment; however, liraglutide treatment will be open label, so the study is considered partially blinded. The randomization will be stratified by baseline BMI (27 to less than 30 kg/m^2, 30 to less than 35 kg/m^2, and 35 to 50 kg/m^2).

Tirzepatide QW dosing will start at 5 mg for 3 weeks followed by a dose escalation to 10 mg for 3 weeks. Subjects will return to the clinical research unit each week for the QW administration of tirzepatide or placebo.

Liraglutide QD dosing will start at 0.6 mg for 1 week followed by weekly, step-wise dose escalations to 1.2, 1.8, and 2.4 mg, until a 3-mg QD dose is reached. The 3-mg dose of liraglutide will be maintained for 10 days. Subjects will self-administer liraglutide at home in the evening.

Figure GPHH.6.1 illustrates the study design.

6.2. Determination of Sample Size

Up to 111 subjects (37 subjects per treatment arm) are planned to be randomized so that approximately 93 subjects (31 subjects per treatment arm) complete the study, assuming a 15% discontinuation rate.

These 111 subjects will be randomized to tirzepatide, liraglutide, or placebo in a 1:1:1 ratio. The estimated variability (standard deviation) of the change in energy intake from baseline is 289 kcal on the results from GPGC and blinded data from study GPGT Study I8F-MC-based based on an expected treatment difference of 212 kcal, this provides at least 80% power for the comparison of tirzepatide versus placebo based on a 2-sample t-test using a 2-sided test at an α level of 0.05.

Approved on 20 Apr 2023 GMT

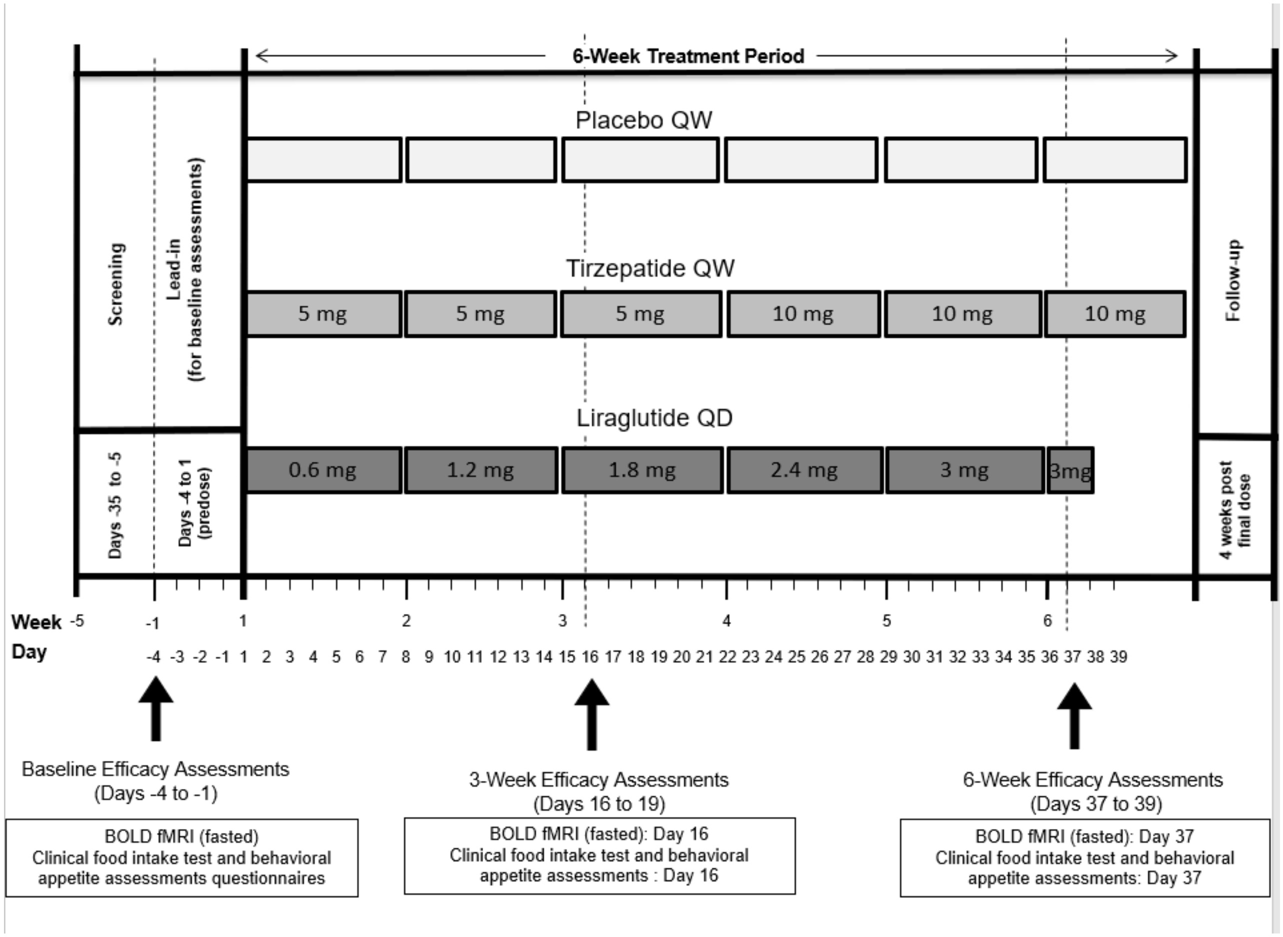

Abbreviations: BOLD = blood-oxygenation-level-dependent; fMRI = functional MRI; MRI = magnetic resonance imaging; QD = once daily; QW = once weekly.

Figure GPHH.6.1. Illustration of study design for Protocol I8F-MC-GPHH.

7. A Priori Statistical Methods

7.1. Populations for Analyses

For the purpose of analysis, Table GPHH.7.1 defines 3 analysis sets.

Table GPHH.7.1. Analysis Populations and Analysis Datasets

**Population/Data Set**	**Description**
Randomized population	All subjects who are randomly assigned to a treatment arm
Safety population	All randomized subjects who receive at least 1 dose of the randomly assigned IP, regardless of whether they completed all protocol requirements
Pharmacodynamic analysis set	All randomized subjects who receive at least 1 dose of the randomly assigned IP and have evaluable data

Abbreviations: IP = investigational product; MRI = magnetic resonance imaging; PD = pharmacodynamic.
Note: Protocol deviations will be considered for their severity/impact and taken into consideration if subjects should be excluded from PD analysis set for MRI data-related analyses.

7.2. General Considerations

Statistical analysis of this study will be the responsibility of Lilly or its designee. Any change to the statistical methods described in the protocol will require a protocol amendment only if it changes a principal feature of the protocol. Any other change to the statistical analyses and the justification for the change will be documented in this SAP.

Unless otherwise specified, safety analyses will be conducted on the safety population (Table GPHH.7.1), and PD analyses will be conducted on the PD analysis set (Table GPHH.7.1).

Unless otherwise specified, the baseline values used for analyses will be the last scheduled, nonmissing value obtained for each subject before first dose.

Unless otherwise noted, all tests of treatment effects will be conducted at a 2-sided α level of 0.05, and the CI will be calculated at 95%, 2-sided.

7.3. Study Participant Characteristics

Subject baseline demographic and clinical characteristics will be summarized for the randomized population by study treatment (including overall). Specifically, the following baseline parameters will be included in (but not limited to) the summary report:

- age (years)
- gender (male and female)
- race
- ethnicity (Hispanic and Non-Hispanic)
- body weight (kg)
- height (cm)
- BMI (kg/m^2)
- BMI (27 to less than 30, 30 to less than 35, and 35 to 50 kg/m^2), and

- waist circumference (cm).

7.4. Study Participant Disposition

A listing and a summary of subject disposition for the randomized population (that is, subjects who have been assigned to a treatment) by actual study treatment will be provided.

A listing of patients who discontinue from the study for any reason for the randomized population will be provided, and the extent of their participation in the study will be reported. The reason for their discontinuation from study will be reported. The listing will also include age, sex, race, treatment, and study period.

7.5. Concomitant Therapy

Concomitant medications will be listed and summarized by treatment and World Health Organization Drug Dictionary Medication Class and PT for the safety population. In addition, concomitant medication taken by 10 or more subjects, regardless of treatment, will also be summarized.

7.6. Treatment Exposure and Compliance

7.6.1. Treatment Exposure

A listing of enrolled subjects who had inadvertently received the incorrect study treatment anytime during the study will be provided, if applicable.

The duration of exposure to study treatment (tirzepatide, liraglutide, or placebo) is defined as

date of last dose of study treatment - date of first dose of study treatment + 7 days

The duration of exposure to study treatment will be summarized by treatment using the safety population.

7.6.2. Treatment Compliance

Percent compliance will be listed for each patient and summarized by treatment on the safety population. Percent compliance and treatment compliance will be summarized by treatment for each period and by treatment only.

Percent compliance will be calculated for each subject as

$$\left(Number\ of\ injections\ administered\ [regardless\ of\ actual\ dose\ in\ mg\ administered] \div total\ number\ of\ injections\ expected\ to\ be\ administered\right) \times 100$$

Patients will be considered treatment compliant if taking 75% or more of their scheduled doses for the 6-week treatment period, considering early termination (specifically excluding the period after early termination).

Approved on 20 Apr 2023 GMT

7.7. Important Protocol Deviations

Important protocol deviations are defined as deviations from the study protocol that may significantly compromise the data integrity and/or patient safety. The details of identification of IPDs are provided in a separate document (that is, Risk and Issue Mitigation and Management Tool [RIMM]). A listing/table of IPDs will be provided by the study manger after database lock.

7.8. Pharmacokinetic Analyses

7.8.1. Pharmacokinetic Parameter Estimation

Sparse PK samples will be collected across the 6-week treatment period. Tirzepatide concentrations will be determined to support an understanding of tirzepatide exposure over the treatment duration and compare with expected tirzepatide PK.

7.8.2. Pharmacokinetic Statistical Inference

No summaries or analyses of PK parameters are planned. Tirzepatide concentrations may be summarized by week.

7.9. Pharmacodynamic Analyses

7.9.1. Pharmacodynamic Parameter Estimation

Pharmacodynamic parameters will be measured at lead-in (baseline), Week 3, and/or Week 6, to assess the effect of study treatments on measures of appetite and food intake, food intake-related and impulsiveness questionnaires, central reward and appetite circuits in the brain, and hormones and metabolites related to appetite regulation. The change from baseline to Week 3 and Week 6 (if applicable) will be calculated for all continuous PD parameters.

Table GPHH.7.2. Study Period and PD Measurements

	Period	Visit (Study Day)	Week	PD Measurements
1	Lead-in (baseline)	2 (between Day -4 and Day -1)	-1	• Ad-libitum food intake test at lunch (clinic-based) with appetite VAS<br>• FCI, FCQ-S, Eating Inventory, BIS and Power of Food Scale questionnaires<br>• Retrospective appetite VAS<br>• BOLD fMRI<br>• Hormones and metabolites for appetite and metabolism regulation in the fasting and postprandial states
2	Treatment	6 (16 ±1)	Week 3	• Ad-libitum food intake test at lunch (clinic-based) with appetite VAS<br>• FCI, FCQ-S, Eating Inventory, BIS and Power of Food Scale questionnaires<br>• Retrospective appetite VAS

	Period	Visit (Study Day)	Week	PD Measurements
				• BOLD fMRI<br>• Hormones and metabolites for appetite and metabolism regulation in the fasting and postprandial states
3	Treatment	11 (37 ±1)	Week 6	• Ad-libitum food intake test at lunch (clinic-based) with appetite VAS<br>• FCI, FCQ-S, Eating Inventory, BIS and Power of Food Scale questionnaires<br>• Retrospective appetite VAS<br>• BOLD fMRI<br>• Hormones and metabolites for appetite and metabolism regulation in the fasting and postprandial states
4	Follow-up	12 (59 ±1 if TZP or placebo and 61 ±1 if liraglutide)	Follow-up Week 3	• Ad-libitum food intake test at lunch (clinic-based)
5	Follow-up	All days between 12 and 13 (~4 days)		• Ad-libitum food intake assessment (free-living)
6	Follow-up	13 (64 ±1 if TZP/ or placebo and 66 ±1 if liraglutide)	Follow-up Week 4	• Ad-libitum food intake test at lunch (clinic-based)

Abbreviations: ~ = approximately; BIS = Barratt Impulsiveness Scale; BOLD = blood-oxygenation-level-dependent; FCI = Food Craving Inventory; FCQ-S = Food Craving Questionnaire-State; fMRI = functional MRI; MRI = magnetic resonance imaging; PD = pharmacodynamics; TZP = tirzepatide; VAS = visual analog scale.

7.9.1.1. Food Intake (Ad-Libitum Food-Intake Test)

Food intake will be quantified through an ad-libitum food-intake test at lunch in a clinical setting and in free-living conditions on baseline (measured in the lead-in period between Day -4 to Day -1), treatment Week 3 (Visit 6, Day 16) and Week 6 (Visit 11, Day 37), and during the follow-up period specified in the above table.

7.9.1.2. Central Reward and Appetite Circuits using BOLD fMRI

To characterize the treatment effect on central reward and appetite circuits in the brain, MRI scans will be completed during the study. In particular, BOLD fMRI will be completed by subjects at lead-in (measured between Day -4 to Day -1) and treatment Week 3 (Visit 6, Day 16) and Week 6 (Visit 11, Day 37), respectively.

On each of the 3 scheduled visits, 1 MRI scan session will be completed in a fasted state. Subjects will perform a food image task during each scan.

The principal brain reward and appetite areas to be examined will be the

- insula

- medial frontal gyrus
- superior temporal gyrus
- precentral gyrus, and
- cingulate gyrus.

In addition, exploratory brain reward and appetite areas to be examined will be the

- hippocampus
- putamen
- orbitofrontal cortex, and
- ventral striatum.

7.9.1.3. Appetite Visual Analog Scale and Retrospective Appetite Visual Analog Scale

The aim of the appetite VAS is to determine the effects of study treatments on appetite sensations and desire for specific foods. Two types of VASs will be used to assess subjective ratings of appetite and affect: laboratory-based and retrospective. The VASs will be analyzed as continuous variables on the 0 to 100 scale for individual components.

Visual analog scale scores will be documented in an electronic clinical report form (eCRF). During the laboratory-based test meals at baseline (that is, lead-in, measured between Day -4 to Day -1), Week 3, and Week 6, subjects will complete pen-and-paper VAS ratings immediately before and after the test lunch and document scores in the eCRF.

7.9.1.4. Measures of Food Intake-Related and Impulsiveness Questionnaires

The FCI, FCQ-S, Eating Inventory (also known as the TFEQ), Power of Food Scale, and BIS questionnaires are administered at baseline (measured in the lead-in period between Day -4 to Day -1), Week 3, and Week 6 postprandially and will be documented in the eCRF.

Food Craving Inventory

The 33-item FCI (White et al. 2002) will be used to measure cravings for specific food groups. The measure consists of 5 empirically derived factors. The FCI will be completed at baseline (measured in the lead-in period between Day -4 to Day -1), Week 3, and Week 6 in a fasted state shortly after arriving to the study site and will be documented in the eCRF.

Food Craving Questionnaire-State

The FCQ-S (Cepeda-Benito et al. 2000; Moreno et al. 2008) is a 15-item measure that assesses the strength of state food cravings at the moment of administration. All items are scored using a 5-point Likert scale in the following manner:

- Strongly disagree - (1).
- Disagree - (2).
- Neutral - (3).
- Agree - (4).
- Strongly agree - (5).

Greater scores for each subscale denote higher levels of craving.

The FCQ-S will be completed at baseline (measured in the lead-in period between Day -4 to Day -1), Week 3, and Week 6 in a fasted state shortly after arriving to the study site and will be documented in the eCRF.

Eating Inventory

The Eating Inventory (Stunkard and Messick 1985; Stunkard and Messick 1988) or the TFEQ is a 51-item, validated questionnaire that assesses 3 eating-related constructs:

- dietary restraint (21 items)
- disinhibition (16 items), and
- perceived hunger (14 items).

Dietary restraint refers to the intent and ability to restrict food intake, disinhibition measures the tendency to overeat, and perceived hunger measures susceptibility to feelings of hunger. Different items have different scales. The TFEQ will be completed at baseline (measured in the lead-in period between Day -4 to Day -1), Week 3, and Week 6 and will be documented in the eCRF.

Power of Food Scale

The 21-item Power of Food Scale (Cappelleri et al. 2009; Lowe et al. 2009) measures appetite for palatable foods. Among 21 items, 15 items are used to calculate the subdomain scores at the following 3 levels of food proximity (3 domain scores):

- food available (6 items)
- food present (4 items), and
- food tasted (5 items).

The Power of Food Scale will be completed at baseline (measured in the lead-in period between Day -4 to Day -1), Week 3, and Week 6 and will be documented in the eCRF.

Barratt Impulsiveness Scale

Impulsivity will be measured with the BIS. The BIS (Patton et al. 1995; Reid et al. 2014) is a 30-item, self-reported measure describing impulsive or nonimpulsive behaviors and preferences.

The BIS will be completed at baseline (measured in the lead-in period between Day -4 to Day -1), Week 3, and Week 6 in a fasted state shortly after arriving to the study site and will be documented in the eCRF.

7.9.1.5. Hormones and Metabolites Related to Appetite Regulation

To explore potential effects of study treatment on hormones related to appetite and the regulation of nutrient metabolism, plasma or serum samples will be collected and assayed. The following hormones measured at baseline (measured in the lead-in period between Day -4 to Day -1), Week 3, and Week 6 at a central laboratory will be exploratory PD parameters of interest and will be only tested once from either plasma or serum:

- amylin (postprandial)
- ghrelin (fasting)
- GIP (postprandial)
- GLP-1 (postprandial)
- leptin (postprandial)
- pancreatic polypeptide (postprandial)
- PYY (postprandial)
- insulin (fasting and postprandial), and
- glucagon (fasting).

In addition to safety monitoring, the following metabolites will also be measured as exploratory endpoints at baseline (Day -4; local laboratory), Week 3 (central laboratory), and Week 6 (central laboratory):

- plasma glucose (fasting), and
- triglycerides (fasting).

Glucose and triglycerides do not have central laboratory measures at baseline, so local laboratory measures will be used for baseline.

7.9.2. Pharmacodynamic Statistical Inference

Pharmacodynamic parameters at baseline (measured in the lead-in period between Day -4 to Day -1), Week 3, and Week 6 (if applicable), as well as change from baseline values at Week 3 and Week 6 and change from Week 3 at Week 6, will be summarized by treatment. Plots of mean PD parameters and mean changes from baseline over time may be provided by treatment and by time (that is, Week) if deemed necessary. The change from baseline to Week 3 and Week 6 (if applicable), calculated for all the continuous PD parameters, will be analyzed.

7.9.2.1. Primary Pharmacodynamic Analysis

7.9.2.1.1. Energy Intake via Ad-libitum Food Intake Test

The primary endpoint in this study is the change from baseline to Week 3 in energy intake (kcal) as assessed by an ad-libitum food-intake test at lunch in a clinical setting for the comparison of 5-mg tirzepatide versus placebo.

For clinical settings, the amount of grams of fat (saturated and unsaturated), carbohydrates, and protein consumed will be documented in the eCRF. These calories will be represented in kcal format using the following conversion: 1 gram of carbohydrate or protein = 4 kcal, 1 gram of saturated fat or unsaturated fat = 9 kcal. The energy intake will be calculated as the total calories (kcal) and recorded in an eCRF as well. The percentage of fat, carbohydrate, and protein in total energy may be derived:

- food intake
    - g (that is, the amount of grams of food consumed)
- fat (saturated and unsaturated)
    - g and kcal

    - percent of energy (that is, fat [kcal]/energy intake [kcal])
- carbohydrate
    - g and kcal
    - percent of energy (that is, carbohydrate [kcal]/energy intake [kcal]), and
- protein
    - g and kcal
    - percent of energy (that is, protein [kcal]/energy intake [kcal]).

The primary endpoint will be analyzed using an analysis of covariance to compare the effect of tirzepatide versus placebo at Week 3 on lunch energy intake (kcal) with treatment as fixed effect, baseline BMI stratum, and baseline energy (kcal) intake as covariates in the model using the PD analysis set.

Inferential statistics will include LSMs (Standard error) of energy (kcal) intake by treatment and the treatment difference of tirzepatide versus placebo in LS Mean difference with 95% CI, and the p value for treatment comparison will be displayed.

7.9.2.2. Secondary Pharmacodynamic Analyses

Secondary PD measures (that is, BOLD fMRI activation; VAS; and the FCI, FCQ-S, TFEQ, and Power of Food Scale questionnaires [see Section 7.10.1 for details]) are scheduled to be measured twice postbaseline at Week 3 and Week 6.

The PD measurements will be analyzed using a MMRM method to compare the effect of tirzepatide versus placebo at Week 3. The response variable is change from baseline in the PD measurement (that is, postbaseline - baseline), where postbaseline measurement is collected at Week 3 and Week 6. The model will include treatment, baseline BMI stratum, week, and treatment-by-week interaction as fixed effects and baseline PD measurement as a covariate; scanner ID will be included as a covariate for modelling the function MRI parameters as well. An unstructured covariance structure will be used to model the within-subject variabilities if deemed appropriate. The restricted-maximum-likelihood approach will be used to obtain model parameter estimates.

For both secondary and exploratory PD parameters, a single model for each parameter will be constructed. The inferential results will be displayed into section for secondary endpoints and section for exploratory endpoints, separately.

For secondary endpoints, inferential statistics will include LSMs (Standard error) of each treatment, the treatment difference with 95% CI at Week 3, along with the p value for comparison.

7.9.2.2.1. Central Reward and Appetite Circuits in the Fasting State using BOLD fMRI

Secondary PD measures to assess the effect of 5-mg tirzepatide versus placebo at Week 3 on parameters of central reward and appetite circuits during fasting and postprandial states using BOLD fMRI are

- change from baseline in mean difference to Week 3 in BOLD fMRI activation to images of highly palatable food (versus nonfood objects) during fasting states in 5 brain reward and appetite areas (insula, medial frontal gyrus, superior temporal gyrus, precentral gyrus, and cingulate gyrus).

The 5 principal brain reward and appetite ROI to be examined and recorded will be

- insula
- medial frontal gyrus
- superior temporal gyrus
- precentral gyrus, and
- cingulate gyrus.

Four additional exploratory brain reward and appetite ROI to be examined and recorded will be

- hippocampus
- putamen
- orbitofrontal cortex, and
- ventral striatum.

Mean differences in BOLD signal between views of highly palatable food (high-fat, high-sugar and high-fat, high-carbohydrate) versus nonfood objects (for example, office supplies and furniture) will be calculated within 5 principal food-reward and appetite-related ROI and 4 exploratory food-reward and appetite-related ROI.

These validated contrast values (that is, mean difference in BOLD signal) (continuous) will be provided by image data processing vendor (Clario) to statisticians/programmers for analyses. Change from baseline in BOLD contrast values at Week3 and at Week6 will be summarized by treatment.

Additional analyses using other functional contrasts (for example, highly palatable foods versus not highly palatable foods [low-fat, low-carbohydrate, high-protein] and not highly palatable foods versus nonfood objects) may be conducted at the 5 principal food-reward and appetite-related ROI and 4 exploratory food-reward and appetite-related ROI if deemed necessary.

7.9.2.2.2. Behavioral Appetite Assessments

Secondary PD measures to assess the effect of 5-mg tirzepatide versus placebo at Week 3 on parameters of behavioral appetite assessments are

- change from baseline to Week 3
  - in fasting and postprandial appetite VAS (laboratory-based), and
  - fasting FCI, FCQ-S, TFEQ, and Power of Food Scale questionnaires.

The parameters will be summarized for each at lead-in, Week 3, and Week 6, and the change from baseline at Week 3 and Week 6 will be also summarized. The comparison of secondary objective parameters will be analyzed using an MMRM model respectively for each parameter.

- Response variable - Change from baseline in a secondary PD parameter.

- Independent variables - Treatment, time, and treatment-by-time interaction as fixed effects; baseline value in secondary parameter as a covariate; and patient as a random effect.

Inferential statistics include LSMs and standard error of each of the secondary objective parameters for tirzepatide and placebo, and the estimated treatment difference (tirzepatide versus placebo) at Week 3, corresponding 2-sided 95% CI, and associated p-values.

7.9.2.2.2.1. Fasting and Postprandial Appetite VAS (Laboratory-Based)

Secondary PD measures to compare the treatment effect of 5-mg tirzepatide versus placebo at Week 3 are

- change from baseline (lead-in) in overall score and individual score of fasting and postprandial appetite VAS in laboratory-based setting, and
- change from baseline (lead-in) in overall score and individual score of retrospective Appetite VAS.

The aim of the VAS is to determine the effects of study treatments on appetite sensations and desire for specific foods. Two types of VASs will be used to assess subjective ratings of appetite and effect: laboratory-based appetite VAS and retrospective appetite VAS. The VAS scales will be analyzed as continuous variables on the 0-to-100 scale for individual components. The VAS scores will be documented in eCRF.

The laboratory-based appetite VAS measures subjects at lead-in, Week 3, Week 6, and immediately before and immediately after the ad-libitum food intake test. The 8 individual questions measure hunger, satiety, fullness, prospective food consumption, desire for sweet food, desire for salty food, desire for savory food, and desire for fatty food. For the first 4 ratings, 0 means “Not at all” and 100 means “Extremely”. For the last 4 ratings, 0 means “Yes, very much” and 100 means “No, not at all”.

The retrospective appetite VAS (Womble et al. 2003) will be used to measure the average ratings of appetite that subjects experienced over the past week. In addition to the 4 appetite metrics (satiety, fullness, prospective food consumption, and hunger) noted above (with instructions modified), retrospective VASs will also be used to quantify feelings of nausea, malaise, and gastrointestinal distress over the previous week. These ratings will occur at baseline, Week 3, and Week 6.

Overall appetite score is calculated as the average of the first 4 individual scores (van Can et al. 2014; Flint et al. 2000):

$$\textit{overall appetite score} = (\textit{satiety} + \textit{fullness} + [100 - \textit{prospective food consumption}] + [100 - \textit{hunger}]) / 4$$

The higher overall appetite score indicates less appetite, and the lower score indicates more appetite.

Individual scores (satiety, fullness, hunger, prospective food consumption, desire for sweet food, desire for salty food, desire for savory food, and desire for fatty food) and overall appetite scores

for the Appetite VAS and individual scores (satiety, fullness, hunger, prospective food consumption, nausea, malaise, and gastrointestinal distress) and overall appetite scores of 4 appetite metrics for the retrospective VAS will be summarized at baseline, Week 3, and Week 6, and the change from baseline at Week 3 and Week 6 will also be summarized.

The secondary objective of comparing the treatment effect of 5-mg tirzepatide versus placebo at Week 3 in the change from baseline (lead-in) in the overall score of fasting and postprandial laboratory appetite VAS and retrospective appetite VAS will be analyzed with MMRM model in a way mentioned in the beginning of this section. The change from baseline (lead-in) in overall score of four 4 appetite metrics for the retrospective VAS will be also analyzed with an MMRM model similarly.

7.9.2.2.2.2. Fasting FCI

A secondary objective to compare the effect of 5-mg tirzepatide versus placebo at Week 3 is change from baseline in fasting FCI

The 33-item FCI (White et al. 2002) is used to measure cravings for specific 5 groups (high fats, sweets, carbohydrates/starches, fast food fats, and fruits and vegetables). It is scaled in a frequency format assessing the frequency of cravings for 33 foods over the past month or since the last time the questionnaire was completed. All items are scored in the following 5-point Likert scale

- never - 1.
- rarely - 2.
- sometimes - 3.
- often - 4.
- always - 5.
    - Greater scores denote higher levels of craving.

Secondary objectives to compare the effect of 5-mg tirzepatide versus placebo at Week 3 for the change from baseline in fasting FCI including 5 subscores (average of nonmissing measurements of individual items in that category) and overall scores (average of nonmissing measurements of all individual items) are

- **Subscores**
    - High fats (8 items) - Fried chicken, gravy, sausage, hot dog, fried fish, corn bread, bacon, and steak.
    - Sweets (8 items) - Cake, cinnamon rolls, ice cream, cookies, chocolate, donuts, candy, and brownies.
    - Carbohydrates/starches (8 items) - Sandwich bread, rice, biscuits, pasta, pancakes or waffles, rolls, cereal, and baked potato.
    - Fast food fats (4 items) - Pizza, french fries, hamburger, and chips.
    - Fruits and vegetables (5 items) - Cooked vegetables, fruit juices, raw vegetables, canned fruit, and raw fruit.
- **Overall score** - All 33 items.

The subscale score for each factor and overall score will be summarized at lead-in, Week 3, and Week 6. Change from baseline (lead-in) will be also summarized for the Week 3 and Week 6 visits. Change from Week 3 at Week 6 will be summarized as well. Mean plot and mean change from baseline plot for each subscore and overall score by visit will be supplied.

The secondary objective of comparing the treatment effect of tirzepatide versus placebo at Week 3 in FCI overall score will be analyzed using an MMRM model in a way mentioned in the beginning of this section.

7.9.2.2.2.3. FCQ-S

A secondary objective to compare the treatment effect 5-mg tirzepatide versus placebo at Week 3 is change from baseline in FCQ-S, including 5 subscores and the overall score in the FCQ-S questionnaire.

The FCQ-S (Cepeda-Benito et al. 2000; Moreno et al. 2008) is a 15-item measure that assesses the strength of state food cravings at the moment of administration. The questionnaire contains 5 subscale scores, including desire, anticipation positive, anticipation negative, lack of control, and hunger.

Table GPHH.7.3. FCQ-S Subscore Category and Questions

Subscore category	Question Number and Description
Desire<br>(3 items)	1. I have an intense desire to eat [one or more specific foods].
	2. I'm craving.
	3. I have an urge for [one or more specific foods].
Anticipation positive<br>(3 items)	4. Eating [one or more specific foods] would make things seem just perfect.
	5. If I were to eat what I am craving, I am sure my mood would improve.
	6. Eating [one or more specific foods] would feel wonderful.
Anticipation negative<br>(3 items)	7. If I ate something, I wouldn't feel so sluggish and lethargic.
	8. Satisfying my craving would make me feel less grouchy and irritable
	9. I would feel more alert if I could satisfy my craving.
Lack of control<br>(3 items)	10. If I had [one or more specific foods], I could not stop eating it.
	11. My desire to eat [one or more specific foods] seems overpowering.
	12. I know I'm going to keep on thinking about [one or more specific foods] until I actually have it.
Hunger<br>(3 items)	13. I am hungry.
	14. If I ate right now, my stomach wouldn't feel as empty.
	15. I feel weak because of not eating.

All items are scored using a 5-point Likert scale in the following manner:

- Strongly disagree - 1.
- Disagree - 2.
- Neutral - 3.
- Agree - 4.
- Strongly agree - 5.
    - Greater scores for each subscale denote higher levels of craving.
- **Subscores -** average score from a nonmissing measurement from an individual question.
    - Desire (average score from questions 1-3)
    - Anticipation positive (average of questions 4-6)
    - Anticipation negative (average of questions 7-9)
    - Lack of control (average of questions 10-12), and
    - Hunger (average of questions 13-15).
- **Overall score - Average of the 5 subscores above.**

Each subscale score and overall score will be summarized for the lead-in, Week 3, and Week 6 visits; change from lead-in will be also summarized for the Week 3 and Week 6 visits. Change from Week 3 at Week 6 will be summarized as well. Mean plot and individual plots for each subscale score by visit will be provided.

The secondary objective of comparing the treatment effect of tirzepatide versus placebo at Week 3 in FCQ-S overall score will be analyzed using an MMRM model in a way mentioned in the beginning of this section.

7.9.2.2.2.4. Eating Inventory through TFEQ

A secondary objective to compare the treatment effect 5-mg tirzepatide versus placebo at Week 3 is change from baseline in the TFEQ, including 3 factors:

- dietary restraint
- disinhibition, and
- perceived hunger.

Of the 51 items of the TFEQ, 36 items are on a binary-point (true or false) response scale, 1 item is on a 6-point response scale, and 14 items are on a 4-point response scale, respectively. Scores from sum of individual items for dietary restraint, disinhibition, and perceived hunger range from 0 to 21, 0 to 16, and 0 to 14, respectively, and a greater number indicates greater levels of each respective eating component:

- Binary point questions (36)-1-36
- 4-point responses questions (14)-37-49
- 6-point response questions (1)-51

Table GPHH.7.4. Scoring Algorithm for the TFEQ

Factor	Binary-Point (True/False) Question No.	6-Point or 4-Point Response Scale Question No.
1 - Dietary restraint (21 items)	4, 6, 10, 14, 18, 21, 23, 28, 30, 32, 33, and 35<br><br>**To score 1 point:**<br>• **Answer true -** 4, 6, 14, 18, 23, 28, 32, 33, 35<br>• **Answer false -** 10, 21, 30	37, 38, 40, 42, 43, 44, 46, 48, and 51<br><br>**To score 1 point for 37, 38, 40, 42, 43, 44, 46, or 48**<br>• Selected the third or fourth choice<br>**To score 1 point for 51**<br>• Selected the fourth, fifth, or sixth choice
2 - Disinhibition (16 items)	1, 2, 7, 9, 11, 13, 15, 16, 20, 25, 27, 31, and 36<br><br>**To score 1 point**<br>• **Answer true** - 1, 2, 7, 9, 11, 13, 15, 20, 27, 36<br>• **Answer false** - 16, 25, 31	45, 49, and 50<br><br>**To score 1 point**<br>• Selected the third or fourth choice
3 - Perceived hunger (14 items)	3, 5, 8, 12, 17, 19, 22, 24, 26, 29, 34<br><br>**To score 1 point**<br>• Answer true - 3, 5, 8, 12, 17, 19, 22, 24, 26, 29, 34	39, 41, and 47<br><br>**To score 1 point for 39 or 41**<br>• Selected the third or fourth choice<br>**To score 1 point for 47**<br>• Selected the first or second choice

Abbreviations: No. = number; TFEQ = Three-Factor Eating Questionnaire.

1) Unless specified above, for any other answer/selection, the patient will score 0 for that question item.

2) For question 51, the scale is 1, 2, 3, 4, 5, and 6 sequentially. If subject selected the fourth choice (scale = 4), the fifth choice(scale = 5), or the sixth choice (scale = 6), the subject will score 1 point from this question.

3) The complete questionnaire will be supplemented in appendix.

The TFEQ will be completed at lead-in, Week 3, and Week 6 in a fasted state shortly after arriving to the study site and will be documented in the eCRF.

Summaries of each factor score will be provided for lead-in, Week 3, and Week 6. Change from lead-in at Week 3 and Week 6, and change from Week 3 at Week 6, in each factor score will be summarized by treatment group. Individual and mean plots will be provided.

The secondary objective of comparing the treatment effect of tirzepatide versus placebo at Week 3 in each TFEQ factor score will be analyzed using an MMRM model in a way mentioned in the beginning of this section.

7.9.2.2.2.5. Power of Food Scale Questionnaires

Secondary objectives of comparing the treatment effect of tirzepatide versus placebo at Week 3 are

- change from baseline in each domain score of the Power of Food Scale Questionnaire
  - Food Available Domain
  - Food Present Domain, and

   - Food Tasted Domain, and
- change from baseline in aggregate score of PFS (mean of the 3 domain scores).

The 21-item Power of Food Scale (Cappelleri et al. 2009; Lowe et al. 2009) measures appetite for palatable foods in the environment at the following 3 levels of food proximity (3 domain scores): food available, food present, and food tasted. The original questionnaire was designed with 21 items, and then 6 items were moved after confirmatory factor analysis. In Study I8F-MC-GPHH, all 21 items will be collected from an eCRF, but only 15 of those will be used to calculate domain scores.

Table GPHH.7.5. Power of Food Scale Subdomains and Questions

Domain	Question Number (based on 21-item)	Question
Food Available Domain (6 items)	1	I find myself thinking about food even when I’m not physically hungry.
	3	I get more pleasure from eating than I do from almost anything else.
	8	It’s scary to think of the power that food has over me.
	16	Sometimes, when I am doing everyday activities, I get an urge to eat “out of the blue” (for no apparent reason).
	17	I think I enjoy eating a lot more than most other people.
	19	It seems like I have food on my mind a lot.
Food Present Domain (4 items)	5	If I see or smell a food I like, I get a powerful urge to have some.
	6	When I’m around fattening food I love, it’s hard to stop myself from at least tasting it.
	10	When I know a delicious food is available, I can’t help myself from thinking about having some.
	11	I love the taste of certain foods so much that I can’t avoid eating them even if they are bad for me.
Food Tasted Domain (5 items)	14	Just before I taste a favorite food, I feel intense anticipation.
	15	When I eat delicious food I focus a lot on how good it tastes.
	18	Hearing someone describe a great meal makes me really want to have something to eat.
	20	It is very important to me that the foods I eat are as delicious as possible.
	21	Before I eat a favorite food my mouth tends to flood with saliva.

The aggregate score is also calculated as the mean of the 3 domain scores. All items are scored on a 5-point Likert scale in the following manner:

- Don’t agree at all - 1.
- Agree a little - 2.
- Agree somewhat - 3.
- Agree - 4.
- Strongly agree - 5.
   - A higher item score indicates a greater responsiveness to the food environment.

The aggregate score and each of the 3 domain scores will be summarized for lead-in, Week 3, and Week 6, and the change from lead-in will be summarized for the Week 3 and Week 6 visits.

The secondary objective of comparing the treatment effect of tirzepatide versus placebo at Week 3 in each domain score and aggregate score of the Power of Food Scale Questionnaire will be analyzed using an MMRM model in a way mentioned in the beginning of this section.

7.9.2.2.2.6. Barratt Impulsiveness Scale

The secondary objective of comparing the treatment effect of Tirzepatide versus Placebo on impulsivity at Week 3 is change from baseline in each of the 6 first-order subscores and each of the 3 second-order subscores in the BIS.

Impulsivity will be measured with the BIS. The BIS (Patton et al. 1995; Reid et al. 2014) is a 30-item, self-reported measure describing impulsive or nonimpulsive behaviors and preferences. The measure assesses the personality/behavioral construct of impulsiveness and has demonstrated good psychometric properties in multiple populations. The items are rated on a 4-point scale with responses of

- rarely/never
- occasionally
- often, and
- almost always/always.

The maximum score of 4 indicates the most impulsive response.

The scale consists of 6 first-order factors, and the subscores are calculated as follows:

- Attention (Factor 1) - Average of 5, 9, 11, 20, and 28.
- Motor impulsiveness (Factor 2) - Average of 2, 3, 4, 17, 19, 22, and 25.
- Self-control (Factor 3) - Average of 1, 7, 8, 12, 13, and 14.
- Cognitive complexity (Factor 4) - Average of 10, 15, 18, 27, and 29.
- Perseverance (Factor 5) - Average of 16, 21, 23, and 30.
- Cognitive instability (Factor 6) - Average of 6, 24, and 26.

The scale also consists of 3 second-order factors:

- Attentional impulsiveness (Factor I) - Average of 5, 6, 9, 11, 20, 24, 26, and 28.
- Motor impulsiveness (Factor II) - Average of 2, 3, 4, 16, 17, 19, 21 22, 23, 25, and 30.
- Non-planning Impulsiveness (Factor III) - Average of 1, 7, 8, 10, 12, 13, 14, 15, 18, 27, and 29.

The subscore for a specific factor, first or second order, is calculated as the average of the corresponding items. A total score is calculated as the average of all items.

Table GPHH.7.6. Questions Categorized into First-Order Categories

First-Order Category	Item Number	Item Description
1 - Attention (5 items)	5	I don't "pay attention".
	9	I concentrate easily.
	11	I "squirm" at plays or lectures.
	20	I am a steady thinker.
	28	I am restless at the theater or lectures.
2 - Motor impulsiveness (7 items)	2	I do things without thinking
	3	I make-up my mind quickly.
	4	I am happy-go-lucky.
	17	I act "on impulse".
	19	I act on the spur of the moment.
	22	I buy things on impulse.
	25	I spend or charge more than I earn.
3 - Self-control (6 items)	1	I plan tasks carefully
	7	I plan trips well ahead of time.
	8	I am self controlled.
	12	I am a careful thinker.
	13	I plan for job security.
	14	I say things without thinking.
4 - Cognitive complexity (5 items)	10	I save regularly.
	15	I like to think about complex problems.
	18	I get easily bored when solving thought problems.
	27	I am more interested in the present than the future.
	29	I like puzzles.
5 - Perseverance (4 items)	16	I change jobs.
	21	I change residences.
	23	I can only think about one thing at a time.
	30	I am future oriented.
6 - Cognitive instability (3 items)	6	I have "racing" thoughts.
	24	I change hobbies.
	26	I often have extraneous thoughts when thinking.

Table GPHH.7.7. Questions Categorized into Second-Order Categories

Second-Order Category	Item Number
I - Attentional impulsiveness (8 items)	5, 6, 9, 11, 20, 24, 26, and 28
II - Motor impulsiveness (11 items)	2, 3, 4, 16, 17, 19, 21 22, 23, 25, and 30
III - Non-planning impulsiveness (11 items)	1, 7, 8, 10, 12, 13, 14, 15, 18, 27, and 29

The BIS will be completed at lead-in, Week 3, and Week 6 in a fasted state shortly after arriving to the study site and will be documented in the eCRF.

Each of the 6 first-order subscores, each of the 3 second-order subscores, and the total score from the BIS questionnaire will be summarized at lead-in, Week 3, and Week 6, and the change from lead-in will be summarized for the Week 3 and Week 6 visits.

The secondary objective of comparing the treatment effect of tirzepatide versus placebo at Week 3 in each of the 6 first-order subscores, each of the 3 second-order subscores, and the total score from the BIS questionnaire will be analyzed using an MMRM model in a way mentioned in the beginning of this section.

7.9.2.3. Exploratory Pharmacodynamic Analyses

The PD measurements will be analyzed using the same MMRM method used for secondary PD parameters to compare the effect of tirzepatide versus placebo at Week 3 and/or Week 6.

Inferential statistics will include LSMs of each treatment, the treatment difference, the standard error, and 95% CI at Week 3 and/or Week 6 for below specified exploratory parameters.

Central reward and appetite circuits in the fasting state using BOLD fMRI

An exploratory PD measure to compare the effect of 10-mg tirzepatide versus placebo at Week 6 on parameters of central reward and appetite circuits during fasting state using BOLD fMRI is

- change from baseline to Week 6 in BOLD fMRI activation to images of highly palatable food (high fat-high sugar and high fat-high carbohydrate) (versus nonfood objects) during the fasting states in **5** brain reward and appetite areas (insula, medial frontal gyrus, superior temporal gyrus, precentral gyrus, and cingulate gyrus).

An exploratory PD measure to compare the effect of 5- and 10-mg tirzepatide versus placebo at Week 3 and Week 6 on parameters of central reward and appetite circuits during fasting state using BOLD fMRI is

- change from baseline to Week 3 and Week 6 in BOLD fMRI activation to images of highly palatable food (high fat-high sugar and high fat-high carbohydrate) (versus nonfood objects) during the fasting states in **4** brain reward and appetite areas (hippocampus, putamen, orbitofrontal cortex, and ventral striatum).

An exploratory PD measure to compare the effect of 5-mg and 10-mg tirzepatide versus liraglutide and liraglutide versus placebo at Week 3 and Week 6 on parameters of central reward and appetite circuits during fasting state using BOLD fMRI is

- change from baseline to Week 3 and Week 6 in BOLD fMRI activation to images of highly palatable food (high fat-high sugar and high fat-high carbohydrate) (versus nonfood objects) during the fasting states in **9** brain reward and appetite areas (insula, medial frontal gyrus, superior temporal gyrus, precentral gyrus, cingulate gyrus, hippocampus, putamen, orbitofrontal cortex, and ventral striatum).

Guided by results, additional analyses on parameters of central reward and appetite circuits using other functional contrasts (for example, highly palatable foods versus not highly palatable foods, not highly palatable foods versus nonfood objects) may be conducted at the 5 principal food-reward and appetite-related ROI and 4 exploratory food-reward and appetite-related ROI as deemed appropriate.

Behavioral appetite assessments

Exploratory PD measures to compare the effect of 10-mg tirzepatide versus placebo at Week 6 on parameters of behavioral appetite assessments are

- change from baseline to Week 6
  - in fasting and postprandial appetite VAS (laboratory-based), and
  - in the fasting FCI, FCQ-S, TFEQ, and Power of Food Scale questionnaires.

Exploratory PD measures to compare the effect of 5-mg and 10-mg tirzepatide versus liraglutide and liraglutide versus placebo at Week 3 and Week 6 on parameters of behavioral appetite assessments are

- change from baseline to Week 3 and Week 6
  - in fasting and postprandial appetite VAS (laboratory-based), and
  - in the fasting FCI, FCQ-S, TFEQ, and Power of Food Scale questionnaires.

Energy intake (ad-libitum food-intake test)

An exploratory PD measure to compare the effect of 10-mg tirzepatide versus placebo at Week 6 on energy intake in a clinic-based setting is

- change from baseline in energy intake (kcal) at lunch to Week 6 as assessed in a clinic-based setting.

An exploratory PD measure to compare the effect of 5-mg and 10-mg tirzepatide versus liraglutide and liraglutide versus placebo at Week 3 and Week 6 on energy intake in a clinic-based setting is

- change from baseline in energy intake (kcal) at lunch to Week 3 and Week 6 as assessed in a clinic-based setting.

Impulsivity

An exploratory PD measure to compare the effect of 5-mg and 10-mg tirzepatide versus placebo at Week 3 and Week 6 on parameters of Impulsivity through BIS questionnaire is

- change from baseline in impulsivity in BIS questionnaire.

Hormones and metabolites related to appetite regulation

To explore potential effects of study treatment on hormones related to appetite and the regulation of nutrient metabolism, plasma or serum samples will be collected and assayed. The following hormones measured at baseline, Week 3, and Week 6 at a central laboratory will be exploratory PD parameters of interest and will be only tested once from either plasma or serum:

- amylin (postprandial)
- ghrelin (fasting)
- GIP (postprandial)
- GLP-1 (postprandial)
- leptin (postprandial)
- pancreatic polypeptide (postprandial)
- PYY (postprandial)
- insulin (fasting and postprandial), and
- glucagon (fasting).

In addition to safety monitoring, the following metabolites will also be measured as exploratory endpoints at lead-in (Day -4; local laboratory), Week 3 (central laboratory), and Week 6 (central laboratory): plasma glucose (fasting) and triglycerides (fasting). Note that glucose and triglycerides do not have central laboratory measures at baseline, so local laboratory measures will be used for baseline.

Exploratory PD measures to compare the effect of 5- and 10-mg tirzepatide versus placebo at Week 3 and Week 6 on hormones and metabolites related to appetite regulation are

- change from baseline to Week 3 and Week 6 in
    - ghrelin (fasting)
    - glucagon (fasting)
    - insulin (fasting and postprandial)
    - amylin (postprandial)
    - GIP (postprandial)
    - GLP-1 (postprandial)
    - leptin (postprandial)
    - pancreatic polypeptide (postprandial), and
    - PYY (postprandial)
- change from baseline to Week 3 and Week 6 in plasma glucose (fasting) and triglycerides (fasting).

Exploratory PD measures to compare the effect of 5-mg and 10-mg tirzepatide versus liraglutide, and liraglutide versus placebo, at Week 3 and Week 6 on hormones and metabolites related to appetite regulation are

- change from baseline to Week 3 and Week 6 in
    - ghrelin (fasting)
    - glucagon (fasting)
    - insulin (fasting and postprandial)
    - amylin (postprandial)
    - GIP (postprandial)
    - GLP-1 (postprandial)
    - leptin (postprandial)
    - pancreatic polypeptide (postprandial), and

  - PYY (postprandial)
- change from baseline to Week 3 and Week 6 in plasma glucose (fasting) and triglycerides (fasting).

Hormone and metabolites analyses are considered as exploratory analyses only. The estimand is pairwise treatment difference in terms of change from baseline to treatment Week 3 and 6. In particular, interest lies in comparing tirzepatide 5 mg with liraglutide, tirzepatide 10 mg with liraglutide, and liraglutide with placebo. All 11 variables described above will be analyzed separately using an MMRM model.

Summary for PD analysis

For PD analyses, when the distribution of response variables significantly deviates from normality, alternative statistical methods will be utilized.

Guided by results, for BOLD fMRI , additional analyses on parameters of central reward and appetite circuits using other functional contrasts (for example, highly palatable foods versus not highly palatable foods, not highly palatable foods versus nonfood objects) may be conducted at the 5 principal food-reward and appetite-related ROI and 4 exploratory food-reward and appetite-related ROI as deemed appropriate.

Inferential statistics will include LSMs of each treatment, the treatment difference, standard error, and 95% CI.

7.10. Safety Analyses

Safety measures include, but are not limited to, AEs, TEAEs, SAEs, AESI, vital signs, and safety laboratory measures.

Safety analyses will be performed on the safety population unless specified otherwise. Safety listings will display values/events during study period. Listings of AEs, deaths, and SAEs may include (but will not be limited to)

- subject identification (ID) number
- age
- sex
- race
- treatment
- dose
- MedDRA SOC and PT
- time of onset from the first dose of study drug
- duration of the AE
- seriousness
- severity
- relatedness to study drug
- action taken, and
- outcome, as deemed appropriate.

Additional safety listings will be provided for safety parameters other than AEs if specified in the sections below.

For safety measurements, summary statistics will be provided by treatment. A summary for AEs with a frequency of 10 or more patients with such events will be provided as well.

7.10.1. Adverse Events

Adverse events for the safety population will be listed, which will include the MedDRA PT.

7.10.2. Treatment-Emergent Adverse Events

A TEAE is defined as an AE that first occurs post first dose or that is present prior to the first dose of study drug and becomes more severe post first dose. The maximum severity for each AE during the baseline period, including ongoing medical history, will be used as baseline severity.

Treatment-emergent AEs will be summarized by treatment, severity, and relationship with study drug.

7.10.3. Serious Adverse Events

A listing of patients with SAEs, including deaths, will be produced.

7.10.4. Adverse Events Leading to Discontinuation

A listing of patients with AEs leading to discontinuation from study by treatment will be presented. A summary will be provided if there are 10 or more subjects with such events.

7.10.5. Special Safety Topics

A listing of patients with all AESI defined in Section 7.10.5 will be provided.

7.10.5.1. Hypoglycemia

A listing of documented, clinically significant hypoglycemia (plasma glucose less than 54 mg/dL) and severe hypoglycemia events will be provided. A listing of patients with hypoglycemic events will be provided. The category of hypoglycemic events will be presented. Hypoglycemic events will be summarized by treatment for each category. The incidence of hypoglycemia will be reported.

Severe/serious hypoglycemia is considered an AESI in this trial.

Approved on 20 Apr 2023 GMT

Table GPHH.7.8. Definition of Hypoglycemic Event Categories

	Symptoms and/or Signs of Hypoglycemia	Plasma Glucose Level
**Glucose alert value**	Yes/no/unknown	≤70 mg/dL (3.9 mmol/L)
Documented symptomatic hypoglycemia	Yes	
Documented asymptomatic hypoglycemia	No	
Documented unspecified hypoglycemia	Unknown	
**Clinically significant hypoglycemia**	Yes/no/unknown	<54 mg/dL (3.0 mmol/L)
Documented symptomatic hypoglycemia	Yes	
Documented asymptomatic hypoglycemia	No	
Documented unspecified hypoglycemia	Unknown	

Severe hypoglycemia is defined as an episode with severe cognitive impairment requiring the assistance of another person to actively administer carbohydrate, glucagon, or other resuscitative actions. Severe hypoglycemia will be reported as an SAE.

Nocturnal hypoglycemia is defined as any hypoglycemia events that occurs between bedtime and waking.

To avoid duplicate reporting, all consecutive glucose values 70 mg/dL (3.9 mmol/L) or less occurring within a 1-hour period may be considered as a single hypoglycemic event.

7.10.5.2. Pancreatitis

A listing of patients with (acute) pancreatic events will be provided.

Treatment-emergent, investigator assessed pancreatitis will be considered as an AESI.

7.10.5.3. Thyroid Malignancies and C-Cell Hyperplasia

A listing of patients with thyroid malignancies and C-cell hyperplasia (search criteria in Appendix 1) will be provided.

Thyroid malignancies and C-cell hyperplasia will be considered as AESI.

7.10.5.4. Hypersensitivity Events

A listing of patients with hypersensitivity reactions (search criteria in Appendix 1) will be provided.

Only the serious/severe cases of hypersensitivity will be considered as AESI.

7.10.5.5. Injection-Site Reactions

Injection-site assessments for local tolerability will be conducted when reported as an AE from a patient or as a clinical observation from an investigator.

A listing of patients with reported injection-site reactions (edema, erythema, induration, itching, and pain) will be provided. A frequency table by treatment of the number of subjects reporting edema, erythema, induration, itching, and pain will be provided. Detailed search criteria can be found in Appendix 1.

Only the severe/serious injection site reactions (for example, abscess, cellulitis, erythema, hematomas/hemorrhage, exfoliation/necrosis, pain, subcutaneous nodules, swelling, indurating, and inflammation) will be considered as AESI.

7.10.5.6. Hepatobiliary Disorders

A listing of subjects with events of biliary colic, cholecystitis, or other suspected events related to gallbladder disease will be provided. Detailed search criteria can be found in Appendix 1.

Severe/serious hepatobiliary disorders will be considered as AESI.

Hepatic monitoring

The subjects' liver disease history and associated data will be listed. Any concomitant medications that have potential for hepatotoxicity, including acetaminophen, will be listed. Results from any hepatic monitoring procedures, such as a magnetic resonance elastography scan, and biopsy assessments will be listed if performed.

Hepatic risk factor assessment data will be listed. Liver-related signs and symptoms data will be summarized by treatment and listed. Alcohol and recreational drug-use data will also be listed.

All hepatic chemistry, hematology, coagulation, and serology data will be listed. Values outside the reference ranges will be flagged on the individual subject data listings.

7.10.5.7. Severe/Serious Gastrointestinal Adverse Events

A listing of patients with severe/serious gastrointestinal AEs (such as nausea, vomiting, and diarrhea) will be provided.

Only the PTs in the gastrointestinal MedDRA SOC with serious/severe cases will be considered as AESI.

7.10.5.8. Acute Renal Events

A listing of patients with acute renal events (search criteria in Appendix 1) will be provided. Serious/severe acute renal events will be considered as AESI.

7.10.6. Safety Laboratory Parameters

All laboratory data will be reported in International System of Units and conventional units. Clinical chemistry, hematology and endocrine (calcitonin) data and their changes from baseline will be summarized at each planned visit by treatment using descriptive statistics.

Additionally, clinical chemistry, hematology, urinalysis and endocrine (calcitonin) data outside the reference ranges will be listed.

If any safety lab measurements are (1) below the QL (0.5 × QL may be used for the calculation of summary statistics); (2) above the QL (1.1 × QL may be used for the calculation of summary statistics, if deemed appropriate).

7.10.7. Vital Signs

Descriptive summaries of vital signs by treatment and by visit will be provided for the lead-in, baseline (Day 1 predose), postbaseline, and change from baseline values. Plots of mean vital signs and mean changes from baseline over time will be provided by treatment.

The treatment-emergent abnormal vital signs will be listed. The criteria for identifying patients with treatment-emergent vital sign abnormalities are stated in Table GPHH.7.9.

Table GPHH.7.9. Categorical Criteria for Treatment-Emergent Abnormal Blood Pressure and Pulse Measurements

Parameter	Low	High
Systolic BP (mmHg)<br>(supine or sitting forearm, at heart level)	≤90 and decrease from baseline ≥20	≥140 and increase from baseline ≥20
Diastolic BP (mmHg)<br>(supine or sitting forearm, at heart level)	≤50 and decrease from baseline ≥10	≥90 and increase from baseline ≥10
Pulse (bpm)<br>(supine or sitting)	<50 and decrease from baseline ≥15	>100 and increase from baseline ≥15

Abbreviation: BP = blood pressure.

7.10.8. Body Weight and Waist Circumference

An average of 2 recordings will be derived in weight and waist circumference for each applicable visit. Absolute value and change from baseline (Day 1 predose) will be summarized by treatment for each visit. For change from baseline, a MMRM based model (similar to the MMRM model used for PD parameters) will be used to model the treatment effect in Body Weight and Waist Circumference at Week 3 and Week 6.

7.10.9. Electrocardiogram

Electrocardiograms will be performed for safety monitoring purposes only and will not be presented. Any clinically significant findings from ECGs will be recorded as AEs.

7.11. Immunogenicity

The baseline immunogenicity sample is collected on Day 1 predose.

The frequency and percentage of subjects with preexisting ADAs and that are treatment-emergent ADA positive (TE-ADA+) to tirzepatide will be tabulated.

Treatment-emergent ADAs are defined as those with a titer 2-fold (1 dilution) greater than the minimum required dilution if no ADAs were detected at baseline (Day 1 predose) (treatment-induced ADA) or those with a 4-fold (2 dilutions) increase in titer compared to baseline if ADAs were detected at baseline (treatment-boosted ADAs). For the TE-ADA+ subjects, the distribution of maximum titers may be described.

If cross reactivity to native GLP-1 and GIP, or neutralizing antibodies against native GLP-1 and GIP, assays are performed, the frequency of each may be reported.

The relationship between the presence of antibodies and PD response, including safety and efficacy to tirzepatide, may be assessed. In particular, listings will be provided to show the relationship between ADA response and hypersensitivity and injection site reaction.

Cases of TE-ADAs that are associated with AEs of either severe/serious hypersensitivity or severe/serious injection site reaction will be classified as AESI.

7.12. Interim Analyses

No interim analyses are planned for this study. If an unplanned interim analysis is deemed necessary, the Lilly clinical pharmacologist, clinical research scientist/clinical research physician, investigator, or designee will consult with the appropriate medical director or designee to determine if it is necessary to amend the SAP.

7.13. COVID-19 Impact Assessment

A listing of patients with AEs related to COVID-19, including death due to COVID-19, serious COVID-19 AEs, and COVID-19 AEs may be provided. Protocol deviations that related to COVID-19 may be described in the clinical study report.

7.14. Exploratory Analysis for Motion Sensor Modeling

To evaluate and refine an eating-activity-pattern recognition model, passive detection of eating-activity data will be collected passively via a wearable wrist device during the last week of the follow-up period of the study.

The analyses of the wearable device data will be detailed in a separate document (that is, the digital biomarker analysis plan).

8. Unblinding Plan

The blinding/unblinding plan is in a separate document.

9. Change from the Protocol-Specified Statistical Analyses

For BOLD fMRI, the ***percent*** BOLD signal differences between views of highly palatable food (HF-HS and HF-HCCHO) versus non-food objects (for example, office supplies, furniture) was defined as the analysis variable. In data review in a blinded manner, quite a few extreme outliers were found, as there were a significant number of observations close to zero for non-food objects (the denominator in the percent change calculation). Therefore, the percent BOLD contrast was replaced with *absolute* BOLD contrast as the analysis variable.

10. References

Cappelleri JC, Bushmakin AG, Gerber RA, et al. Evaluating the Power of Food Scale in obese subjects and a general sample of individuals: development and measurement properties. *Int J Obes (Lond)*. 2009;33(8):913-922. https://doi.org/10.1038/ijo.2009.107

Cepeda-Benito A, Gleaves DH, Williams TL, Erath SA. The development and validation of the State and Trait Food-Cravings Questionnaires. *Behav Ther.* 2000;31(1):151-173. https://doi.org/10.1016/S0005-7894(00)80009-X

Flint A, Raben A, Blundell JE, Astrup A. Reproducibility, power and validity of visual analogue scales in assessment of appetite sensations in single test meal studies. *Int J Obes Relat Metab Disord.* 2000;24(1):38-48. https://doi.org/10.1038/sj.ijo.0801083

Lowe MR, Butryn ML, Didie ER, et al. The Power Food Scale. A new measure of the psychological influence of the food environment. *Appetite.* 2009;53(1):114-118. https://doi.org/10.1016/j.appet.2009.05.016

Moreno S, Rodríguez S, Fernandez MC, et al. Clinical validation of the trait and state versions of the Food Craving Questionnaire. *Assessment*. 2008;15(3):375-387. https://doi.org/10.1177/1073191107312651

Patton JH, Stanford MS, Barratt ES. Factor structure of the Barratt Impulsiveness Scale. *J Clin Psychol.* 1995;51(6):768-774. https://doi.org/10.1002/1097-4679(199511)51:6<768::aid-jclp2270510607>3.0.co;2-1

Reid RC, Cyders MA, Moghaddam JF, Fong TW. Psychometric properties of the Barratt Impulsiveness Scale in patients with gambling disorders, hypersexuality, and methamphetamine dependence. *Addict Behav*. 2014;39(11):1640-1645. https://doi.org/10.1016/j.addbeh.2013.11.008

Stunkard AJ, Messick S. *Eating Inventory Manual*. Psychological Corporation, Harcourt Brace Jovanovich; 1988.

Stunkard AJ, Messick S. The three-factor eating questionnaire to measure dietary restraint, disinhibition and hunger. *J Psychosom Res*. 1985;29(1):71-83. https://doi.org/10.1016/0022-3999(85)90010-8

van Can J, Sloth B, Jensen CB, et al. Effects of the once-daily GLP-1 analog liraglutide on gastric emptying, glycemic parameters, appetite and energy metabolism in obese, non-diabetic adults. *Int J Obes (Lond)*. 2014;38(6):784-793. https://doi.org/10.1038/ijo.2013.162

White MA, Whisenhunt BL, Williamson DA, et al. Development and validation of the food-craving inventory. *Obes Res.* 2002;10(2):107-114. https://doi.org/10.1038/oby.2002.17

Womble LG, Wadden TA, Chandler JM, Martin AR. Agreement between weekly vs. daily assessment of appetite. *Appetite.* 2003;40(2):131-135. https://doi.org/10.1016/s0195-6663(02)00170-8

11. Appendices

Appendix 1

The search criteria for AEs of special safety topics and AESI are stored in the location below.

CLUWE:\\STATSCLSTR\LILLYCE\QA\LY3298176\common\AESI_Lab\Search criteria AESIs_TZP.xlsx

Three-Factor Eating Questionnaire

TFEQ	For items 1-36, please answer either "True" or "False".		
		TRUE	FALSE
1.	When I smell a sizzling steak or see a juicy piece of meat, I find it very difficult to keep from eating, even if I have just finished a meal.	☐	☐
2.	I usually eat too much at social occasions, like parties and picnics.	☐	☐
3.	I am usually so hungry that I eat more than three times a day.	☐	☐
4.	When I have eaten my quota of calories, I am usually good about not eating anymore.	☐	☐
5.	Dieting is so hard for me because I just get too hungry.	☐	☐
6.	I deliberately take small helpings as a means of controlling my weight.	☐	☐
7.	Sometimes things just taste so good that I keep on eating even when I am no longer hungry.	☐	☐
8.	Since I am often hungry, I sometimes wish that while I am eating, an expert would tell me that I have had enough or that I can have something more to eat.	☐	☐
9.	When I feel anxious, I find myself eating.	☐	☐
10.	Life is too short to worry about dieting.	☐	☐
11.	Since my weight goes up and down, I have gone on reducing diets more than once.	☐	☐
12.	I often feel so hungry that I just have to eat something	☐	☐
13.	When I am with someone who is overeating, I usually overeat too.	☐	☐
14.	I have a pretty good idea of the number of calories in common foods.	☐	☐
15.	Sometimes when I start eating, I just can't seem to stop.	☐	☐
16.	It is not difficult for me to leave something on my plate.	☐	☐

17. At certain times of the day, I get hungry because I have gotten used to eating then.	☐	☐
18. While on a diet, if I eat food that is not allowed, I consciously eat less for a period of time to make up for it.	☐	☐
19. Being with someone who is eating often makes me hungry enough to eat also.	☐	☐
20. When I feel blue, I often overeat.	☐	☐
21. I enjoy eating too much to spoil it by counting calories or watching my weight.	☐	☐
22. When I see a real delicacy, I often get so hungry that I have to eat right away.	☐	☐
23. I often stop eating when I am not really full as a conscious means of limiting the amount that I eat.	☐	☐
24. I get so hungry that my stomach often seems like a bottomless pit.	☐	☐
25. My weight has hardly changed at all in the last ten years.	☐	☐
26. I am always hungry so it is hard for me to stop eating before I finish the food on my plate.	☐	☐
27. When I feel lonely, I console myself by eating.	☐	☐
28. I consciously hold back at meals in order not to gain weight.	☐	☐
29. I sometimes get very hungry late in the evening or at night.	☐	☐
30. I eat anything I want, any time I want.	☐	☐
31. Without even thinking about it, I take a long time to eat.	☐	☐
32. I count calories as a conscious means of controlling my weight.	☐	☐
33. I do not eat some foods because they make me fat.	☐	☐
34. I am always hungry enough to eat at any time.	☐	☐

35. I pay a great deal of attention to changes in my figure. ☐ ☐

36. While on a diet, if I eat a food that is not allowed, I often then splurge and eat other high calorie foods. ☐ ☐

Please answer the following questions by filling in the circle on your answer sheet corresponding to the letter of the response that is appropriate to you.

37. How often are you dieting in a conscious effort to control your weight?

☐	☐	☐	☐
rarely	sometimes	usually	always

38. Would a weight fluctuation of 5 lbs affect the way you live your life?

☐	☐	☐	☐
not at all	slightly	moderately	very much

39. How often do you feel hungry?

☐	☐	☐	☐
only at meal times	sometimes between meals	often between meals	almost always

40. Do your feelings of guilt about overeating help you to control your food intake?

☐	☐	☐	☐
never	rarely	often	always

41. How difficult would it be for you to stop eating halfway through dinner and not eat for the next four hours?

☐	☐	☐	☐
easy	slightly difficult	moderately difficult	very difficult

42. How conscious are you of what you are eating?

☐	☐	☐	☐
not at all	slightly	moderately	extremely

43. How frequently do you *avoid* “stocking up” on tempting foods?

☐	☐	☐	☐
almost never	seldom	usually	almost always

44. How likely are you to shop for low calorie foods?

☐	☐	☐	☐
unlikely	slightly likely	moderately likely	very likely

45. Do you eat sensibly in front of others and splurge alone?

☐	☐	☐	☐
never	rarely	often	always

46. How likely are you to consciously eat slowly in order to cut down on how much you eat?

☐	☐	☐	☐
unlikely	slightly likely	moderately likely	very likely

47. How frequently do you skip dessert because you are no longer hungry?

☐	☐	☐	☐
almost never	seldom	at least once a week	almost every day

48. How likely are you to consciously eat less than you want?

☐	☐	☐	☐
unlikely	slightly likely	moderately likely	very likely

49. Do you go on eating binges even though you are not hungry?

☐	☐	☐	☐
never	rarely	sometimes	at least once a week

50. To what extent does this statement describe your eating behavior?

"I start dieting in the morning, but because of any number of things that happen during the day, by evening I have given up and eat what I want, promising myself to start dieting again tomorrow."

☐	☐	☐	☐
not like me	little like me	pretty good description of me	describes me perfectly

51. On a scale of 1 to 6, where 1 means no restraint in eating (eat whatever you want, whenever you want it) and 6 means total restraint (constantly limiting food intake and never "giving in"), what number would you give yourself?

☐	☐	☐	☐	☐	☐
1	2	3	4	5	6

Signature Page for VV-CLIN-109361 v1.0

Approval	PPD<br>20-Apr-2023 16:04:32 GMT+0000

Signature Page for VV-CLIN-109361 v1.0
