# Supplementary material for: Tirzepatide on ingestive behavior in adults with overweight or obesity: a randomized 6-week phase 1 trial
Source: Nat Med. 2025 Jun 24;31(9):3141–50. doi: 10.1038/s41591-025-03774-9 (PMC12443625; doi:10.1038/s41591-025-03774-9)
Supplement: Supplementary file 2 — Reporting Summary [file 41591_2025_3774_MOESM2_ESM.pdf]

nature portfolio

Corresponding author(s): Tamer Coskun

Last updated by author(s): May 12, 2025

Reporting Summary

Nature Portfolio wishes to improve the reproducibility of the work that we publish. This form provides structure for consistency and transparency in reporting. For further information on Nature Portfolio policies, see our Editorial Policies and the Editorial Policy Checklist.

Statistics

For all statistical analyses, confirm that the following items are present in the figure legend, table legend, main text, or Methods section.

n/a	Confirmed	
☐	☒	The exact sample size (*n*) for each experimental group/condition, given as a discrete number and unit of measurement
☒	☐	A statement on whether measurements were taken from distinct samples or whether the same sample was measured repeatedly
☐	☒	The statistical test(s) used AND whether they are one- or two-sided<br>*Only common tests should be described solely by name; describe more complex techniques in the Methods section.*
☐	☒	A description of all covariates tested
☐	☒	A description of any assumptions or corrections, such as tests of normality and adjustment for multiple comparisons
☐	☒	A full description of the statistical parameters including central tendency (e.g. means) or other basic estimates (e.g. regression coefficient) AND variation (e.g. standard deviation) or associated estimates of uncertainty (e.g. confidence intervals)
☐	☒	For null hypothesis testing, the test statistic (e.g. *F*, *t*, *r*) with confidence intervals, effect sizes, degrees of freedom and *P* value noted<br>*Give P values as exact values whenever suitable.*
☒	☐	For Bayesian analysis, information on the choice of priors and Markov chain Monte Carlo settings
☒	☐	For hierarchical and complex designs, identification of the appropriate level for tests and full reporting of outcomes
☒	☐	Estimates of effect sizes (e.g. Cohen's *d*, Pearson's *r*), indicating how they were calculated

Our web collection on statistics for biologists contains articles on many of the points above.

Software and code

Policy information about availability of computer code

Data collection	Data for energy intake and questionnaire were collected using electronic case report forms. fMRI data were collected on four different 3T MRI scanners (Siemens Prisma Fit at Indiana School of Medicine, Siemens Prisma at Purdue University, GE Discovery 750w at Pennington Biomedical Research Center, and Philips Ingenia Elition X at Johns Hopkins University).
Data analysis	Statistical analyses were performed using SAS Enterprise Guide Version 8.<br>fMRI data were processed and summarized into regional endpoints by Clario using an in house pipeline based in SPM12.

For manuscripts utilizing custom algorithms or software that are central to the research but not yet described in published literature, software must be made available to editors and reviewers. We strongly encourage code deposition in a community repository (e.g. GitHub). See the Nature Portfolio guidelines for submitting code & software for further information.

Data

Policy information about availability of data

All manuscripts must include a data availability statement. This statement should provide the following information, where applicable:

- Accession codes, unique identifiers, or web links for publicly available datasets
- A description of any restrictions on data availability
- For clinical datasets or third party data, please ensure that the statement adheres to our policy

Data from the analyses in this study cannot be shared publicly due to the sponsor's (Eli Lilly and Company) contractual obligations. Eli Lilly and

Company provides access to all individual participants data collected during the trial, after anonymization, except for pharmacokinetic or genetic data. Data are available to request 6 months after the indication studied has been approved in the USA and European Union and after primary publication acceptance, whichever is later. No expiration date of data requests is currently set once data have been made available. Access is provided after a proposal has been approved by an independednt review commiittee identified for this purpose and after receipt of a signed data-sharing agreement, Data and documents, including the study protocol, statistical analysis plan, clinical study report and blank or annotated case report forms, will be provided in a secure data-sharing environment. For details on submitting a request, see the instructions provided at www.vivli.org. Requestors can also contact the corresponding author for details on submitting a request.

Research involving human participants, their data, or biological material

Policy information about studies with human participants or human data. See also policy information about sex, gender (identity/presentation), and sexual orientation and race, ethnicity and racism.

Reporting on sex and gender	Participants were screened and enrolled irrrespective of their sex. Sex was self reported by participants. Data are provided in Table 1.
Reporting on race, ethnicity, or other socially relevant groupings	Participants were screened and enrolled irrespective of their race and ethnicity. Race and ethnicity were self-reported by participants. Data are provided in Table 1.
Population characteristics	Patient characteristics are provided in Table 1. BMI was included as a covariate in analyses.
Recruitment	Participants were recruited at three study centers in the United States (Pennington Biomedical Research Center, Indiana University School of Medicine, and Johns Hopkins University School of Medicine) by investigators. The participants were enrolled using the predefined inclusion/exclusion criteria, which are listed in the supplementary information. The first participant was enrolled 9 November, 2020 and the last, 14 October, 2022. Randomization determined using a randomization table with treatment codes, was used to reduce bias.
Ethics oversight	The study was approved by institutional review boards at each site (Pennington Biomedical Research Center Institutional Review Board, Human Research Protection Program Office of Research Compliance Indiana University, Johns Hopkins Institutional Review Board). The study was conducted in accordance with the Declaration of Helsinki and International Conference on Harmonization Good Clinical Practice guidelines. All participants provided written informed consent.

Note that full information on the approval of the study protocol must also be provided in the manuscript.

Field-specific reporting

Please select the one below that is the best fit for your research. If you are not sure, read the appropriate sections before making your selection.

☒ Life sciences ☐ Behavioural & social sciences ☐ Ecological, evolutionary & environmental sciences

For a reference copy of the document with all sections, see nature.com/documents/nr-reporting-summary-flat.pdf

Life sciences study design

All studies must disclose on these points even when the disclosure is negative.

Sample size	Approximately 111 participants were planned to be randomized so that 93 participants (31 per treatment group) would complete the study. This sample size provides at least 80% power for the comparison of tirzepatide versus placebo based on a 2-sample t-test at an alpha level of 0.05, given an expected treatment difference of 212 kcal and an assumed common standard deviation (SD) of 289 kcal for the change in energy intake from baseline.
Data exclusions	Pre-specified analyses plan included all randomized subjects who receive at least 1 dose of the randomly assigned study drug and had evaluable data. In this study, all participants received at least 1 dose of study drug.
Replication	As this was a clinical study, measurements were not replicated for each patient. In addition, patients were not permitted to have received previous treatment, which preclude repeated measurements. Instead, sufficient number of participants the were included to obtain appropriate population means.
Randomization	Participants were randomized 1:1:1 to receive tirzepatide, liraglutide, or placebo, using a randomization table with treatment codes.
Blinding	The sponsor, investigators, and participants were blinded to tirzepatide and placebo treatment, but liraglutide treatment was open-label. Therefore, the study was considered partially blinded.

Reporting for specific materials, systems and methods

We require information from authors about some types of materials, experimental systems and methods used in many studies. Here, indicate whether each material, system or method listed is relevant to your study. If you are not sure if a list item applies to your research, read the appropriate section before selecting a response.

Materials & experimental systems

n/a	Involved in the study
☒	☐ Antibodies
☒	☐ Eukaryotic cell lines
☒	☐ Palaeontology and archaeology
☒	☐ Animals and other organisms
☐	☒ Clinical data
☒	☐ Dual use research of concern
☒	☐ Plants

Methods

n/a	Involved in the study
☒	☐ ChIP-seq
☒	☐ Flow cytometry
☐	☒ MRI-based neuroimaging

Clinical data

Policy information about clinical studies

All manuscripts should comply with the ICMJE guidelines for publication of clinical research and a completed CONSORT checklist must be included with all submissions.

Clinical trial registration	This trial is registered with ClinicalTrials.gov, NCT04311411.
Study protocol	Provided in the manuscript Supplement.
Data collection	Data was collected at individual sites in the United States (Pennington Biomedical Research Center, Indiana University School of Medicine, and Johns Hopkins University School of Medicine) by investigators. The participants were enrolled using the predefined inclusion/exclusion criteria, which are listed in the supplementary information. The first participant was enrolled 9 November, 2020 and the last, 14 October, 2022.
Outcomes	Objective and endpoints were prespecified in the protocol and statistical analysis plan. Primary objective was to compare change from baseline to week 3 in energy intake during ad libitum test meals for tirzepatide and placebo. Secondary objectives were change from baseline to week 3 for tirzepatide vs placebo in fasting and postprandial appetite VAS ratings, Food Cravings Inventory, Food Craving Questionnaire-State, Eating Inventory, Power of Food Scale questionnaire ratings, and BOLD activation to photos of FoodHiPal (FoodHiF/HiS and FoodHiF/HiC) relative to non-food during the fasting state in the insula, medial frontal gyrus, superior temporal gyrus, precentral gyrus, and cingulate gyrus. Analyses were conducted using a mixed-model repeated measures in all randomized participants using all available evaluable data.

Plants

Seed stocks	*Report on the source of all seed stocks or other plant material used. If applicable, state the seed stock centre and catalogue number. If plant specimens were collected from the field, describe the collection location, date and sampling procedures.*
Novel plant genotypes	*Describe the methods by which all novel plant genotypes were produced. This includes those generated by transgenic approaches, gene editing, chemical/radiation-based mutagenesis and hybridization. For transgenic lines, describe the transformation method, the number of independent lines analyzed and the generation upon which experiments were performed. For gene-edited lines, describe the editor used, the endogenous sequence targeted for editing, the targeting guide RNA sequence (if applicable) and how the editor was applied.*
Authentication	*Describe any authentication procedures for each seed stock used or novel genotype generated. Describe any experiments used to assess the effect of a mutation and, where applicable, how potential secondary effects (e.g. second site T-DNA insertions, mosiacism, off-target gene editing) were examined.*

Magnetic resonance imaging

Experimental design

Design type	BOLD functional magnetic resonance imaging plus food cue task.
Design specifications	BOLD imaging in response to task acquired in fasting state at baseline, 3 & 6 weeks post treatment.
Behavioral performance measures	Positive BOLD Contrast (activation) to various food groups relative to non-food objects in pre-specified regions.

Acquisition

Imaging type(s)	2D AxialBOLD fMRI. 3DT1
Field strength	3 Tesla
Sequence & imaging parameters	fMRI: 3.5mm slice thickness, 42 slices, FOV 42mm, Matrix 64x64, in plane res 3.44x3.44, TR=3s, TE=30ms, flip angle 90 degrees
Area of acquisition	Brain

Diffusion MRI ☐ Used ☒ Not used

Preprocessing

Preprocessing software	SPM (statistical parametric mapping)
Normalization	BOLD fMRI slice-time corrected and realigned to the mean; 3DT1 structural MRI is coregistered to the mean. 3DT1 is segmented and deformation field to MNI is generated. Deformation fields are applied to normalize the 3DT1 and fMRI into MNI space at 1x1x3 mm and 3x3x3 mm resolution respectively. The fMRI time series is then smoothed using a 6 mm isotropic FWHM.
Normalization template	MNI
Noise and artifact removal	-
Volume censoring	Head motion and outlier time point regressors are derived from the raw fMRI time series using thresholds based on translation, rotation and global mean activation.

Statistical modeling & inference

Model type and settings	Mixed Model Repeated Measures (MMRM) to test for treatment effect on change from baseline in BOLD contrast. An unstructured covariance structure will be used to model the within-subject errors if deemed appropriate. The restricted-maximum-likelihood (REML) approach will be used to obtain model parameter estimates. The Kenward-Roger approximation will be used to estimate the denominator degrees of freedom.
Effect(s) tested	The response variable is change from baseline in BOLD contrast (that is, postbaseline - baseline), where postbaseline measurement is collected at Week 3 and Week 6. The MMRM model included treatment, baseline BMI stratum, Week, scanner, and treatment-by-week interaction as fixed effects and baseline fMRI BOLD contrast measurement as a covariate

Specify type of analysis: ☐ Whole brain ☒ ROI-based ☐ Both

Anatomical location(s)	BOLD activation was measured in nine a priori-defined brain ROIs based on the AAL atlas. The insula, medial frontal gyrus, superior temporal gyrus, precentral gyrus, and cingulate gyrus were assessed as the principal brain reward ROIs and the hippocampus, putamen, orbitofrontal cortex, and ventral striatum were assessed as exploratory ROIs
Statistic type for inference (See Eklund et al. 2016)	Inferential statistics will include least squares means at each visit, the standard error and 95% CI.
Correction	None

Models & analysis

n/a	Involved in the study
☒	☐ Functional and/or effective connectivity
☒	☐ Graph analysis
☐	☒ Multivariate modeling or predictive analysis

Multivariate modeling and predictive analysis	Task condition time series and multiple regressor time series are entered into a GLM with the smoothed BOLD fMRI time series to estimate beta coefficient images for each condition. Contrast images are generated by comparing each condition of interest against the control condition (non-food or not highly palatable food).
